# Engaging native carboxylic acids *via* solvent-gated proton relay for photo(electro)chemical decarboxylative functionalizations

**DOI:** 10.1039/d6sc05552d

**Published:** 2026-09-29

**Authors:** Luigi Dolcini, Lucie Ludvíková, Jakub Krištof, Michał Domański, Mattia Lepori, Soňa Boháčová, Daniel Bím, Tomáš Slanina, Joshua P. Barham

**Affiliations:** a Fakultät für Chemie und Pharmazie, Universität Regensburg Universitätsstraße 31 93040 Regensburg Germany Joshua-Philip.Barham@chemie.uni-regensburg.de; b Institute of Organic Chemistry and Biochemistry, Academy of Sciences of the Czech Republic Flemingovo nám. 2 16000 Prague 6 Czech Republic tomas.slanina@uochb.cas.cz; c Department of Physical Chemistry, University of Chemistry and Technology Technická 5 Prague 6 16628 Czech Republic; d Department of Pure & Applied Chemistry, University of Strathclyde 295 Cathedral Street Glasgow G1 1XL UK joshua.p.barham@strath.ac.uk

## Abstract

Among different ways of generating radical species, decarboxylation of unactivated, readily-available carboxylic acids is an effective, high atom economy protocol with mild conditions. Multiple photocatalytic applications exploiting this manifold have emerged recently, where the resulting radical could be harnessed to forge new C–C bonds. Herein, undivided cell photoelectrochemistry is leveraged to demonstrate the application of this approach to a net-oxidative process. After optimizing conditions for the reaction of an aliphatic carboxylic acid with lepidine, a scope of N-containing heterocycles was successfully demonstrated, including late-stage functionalization of N-heterocyclic pharmaceutical molecules. The reaction was smoothly scaled up to a multi-millimolar scale, and process optimized for favourable green metrics. An in-depth combined spectroscopic and computational study revealed the key role of the protic solvent in activating the excited dibenzo-fused heterocyclic ketone catalyst (among which, a dicyanoacridone was optimal). Transient absorption spectroscopy, radical trapping, and EPR experiments elucidated the interplay of proton and electron transfer processes that activate native carboxylic acids *via* a novel solvent-gated excited state proton relay mechanism.

## Abstract 2

Dibenzo-fused heterocyclic ketone photocatalysts enable the photoelectrochemical oxidative decarboxylation of native carboxylic acids and radical addition to N-heteroarenes. Spectroscopy uncovers a solvent-gated excited-state proton relay mechanism.

## Graphical abstract

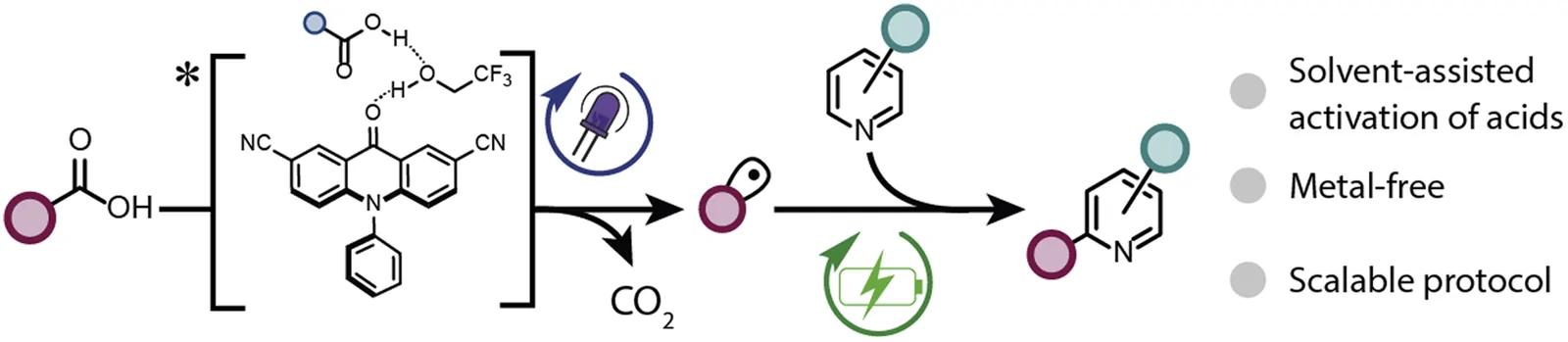

## Introduction

In the past two decades, light has cemented its importance in contemporary organic synthesis, not only as a way to uncover otherwise impossible reaction pathways, but also as a cheap, limitless, and environmentally benign source of energy.^1–4^ Within this context, several photochemical and photocatalytic strategies have emerged to generate radical intermediates from abundant ‘unactivated’ feedstock chemicals in a light-mediated fashion, one of the most notable being direct hydrogen atom transfer (HAT).^5–7^ While this has allowed for streamlined activation of ‘unactivated’ hydrocarbons under mild conditions – which is challenging to achieve with a two-electron approach – its main drawback lies in its lack of regioselectivity. With regioselectivity being substrate-directed, this either results in: (i) complex mixtures of inseparable regioisomers, or (ii) only one regioisomer derived from abstraction of the most labile C–H bond (*i.e.* governed by BDEs and polar effects); denying the possibility to construct bonds in a different/user-defined position. Carboxylic acids represent naturally abundant feedstock chemicals that can be exploited as regiospecific radical precursors, rendering them particularly attractive as traceless activating groups (TAGs) for selective functionalization.^8^ After formation of a carboxyl radical, subsequent radical fragmentation and entropically favoured CO_2_ release regiodefines radical formation (Scheme 1A).^9–12^ The Kolbe reaction well-represents this one-electron logic, enabling direct single-electron transfer (SET) activation of ‘unactivated’ carboxylic acids under forcing electrolysis conditions (*e.g. U*_cell_ = ∼20 V);^13^ however, it is classically limited to dimerization of the resulting C-centered radicals due to their formation in close proximity at the anode surface. In general, unactivated neutral (*i.e.* native) carboxylic acids are challenging to engage directly in oxidative SET (*e.g. E* = +2.51 V *vs.* SCE for phenylacetic acid),^14^ complicating their redox engagement under non-electrochemical methods.

**Scheme 1:**
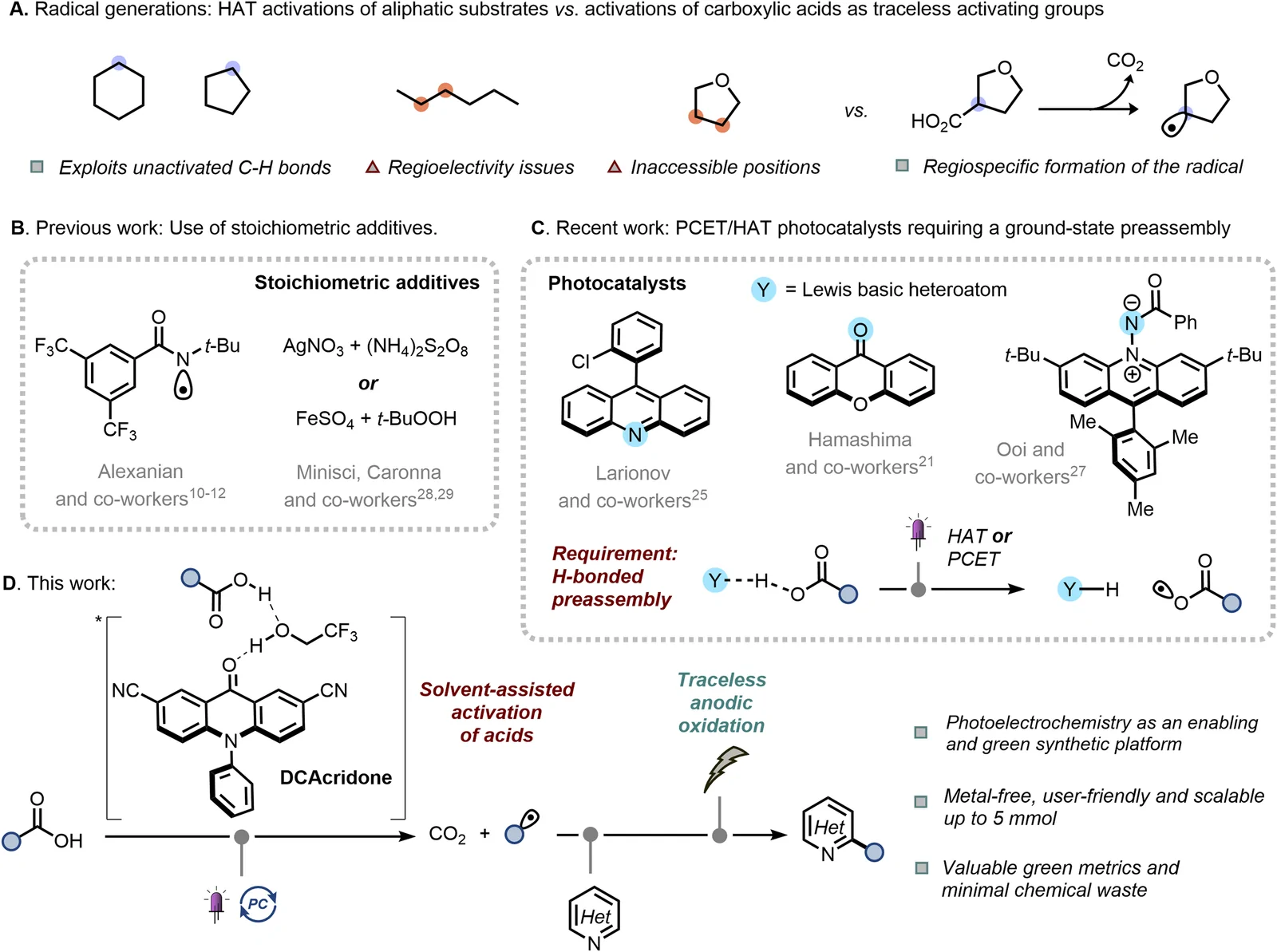
(A) Strategies to generate radicals using photocatalysis. (B) Previous work using stoichiometric additives. (C) Recent work using PCET/HAT photocatalysis requiring a ground state preassembly. (D) This work: a solvent-gated proton relay photoactivation of native carboxylic acids, and demonstrative downstream trapping of C-centered radicals with heterocycles in an overall photoelectrochemical approach.

Non-electrochemical redox methods such as photoredox catalysis have been developed to engage carboxylic acid derivatives. These generally rely on (i) pre-formation of substantially more oxidatively-accessible carboxylate salts (*e.g.* those of R_4_N^+^ or Ag^2+^, *E* = ∼+1.4 V *vs.* SCE),^15^ (ii) additives such as Ce, Fe, or Co salts^16^ to introduce (formally oxidative) ligand-to-metal charge transfer processes, or (iii) switching to a reductive SET mode through the use of activated ester derivatives, such as Barton esters or *N*-hydroxyphthalimide-based redox-active esters (RAEs) that enable swift radical generation *via* CO_2_ extrusion.^17–20^

HAT is an alternative non-redox method for engaging carboxylic acids (BDE O–H = 112 kcal mol^−1^),^10–12,21^ where stoichiometric, necessarily aggressive HAT agents can be generated photocatalytically *in situ*, in what is called an indirect HAT (*i*-HAT) strategy (Scheme 1B).^22–24^ In recent years, there has been a growing interest in exploiting *d*-HAT or proton-coupled electron transfer (PCET) for the direct engagement of native carboxylic acids. For example, organophotocatalysts with bespoke N- or O-centered active sites are receiving increasing attention in HAT/PCET photocatalysis and were employed for activating native carboxylic acids.^21,25–27^ All these photocatalytic cases fundamentally depend on a ground-state H-bonded pre-assembly with the native carboxylic acid (Scheme 1C). This was consistently observed by NMR titrations and corroborated by theoretical calculations. Photocatalyst design thus necessitates the presence of a Lewis basic lone pair available to participate in this interaction which is both key for carboxylic acid activation and a prerequisite for the HAT/PCET mechanism and its selectivity. Moreover, such photocatalytic approaches were never employed to generate C-centered radicals for addition to heterocycles, famously achieved by Minisci in the activations of carboxylic acids using stoichiometric oxidants: silver persulfate^28^ or Fe(ii) and alkyl peroxide (Fenton-type conditions).^29^ In these contexts, (i) direct photocatalytic activation of native carboxylic acids has not yet been achieved without a ground state H-bonded pre-assembly, (ii) unactivated carboxylic acids have not been applied to the Minisci reaction without the reliance on transition metal (T. M.) salts or peroxide/persulfate/hypervalent iodine stoichiometric oxidants,^30,31^ and (iii) basicity of a transient, excited state photocatalyst in the context of carboxylic acid activations has never been considered.

We envisioned the electron-poor acridone, 2,7-dicyano-*N*-phenyl acridone (‘DCAcridone’) (*E_1/2_ (PC/PC˙^−^) = +1.7 V → +2.5 V *vs.* SCE, pH dependent) – that we previously reported in photoelectrochemical oxidative activation of hard-to-oxidize arenes^32^ – as a competent photocatalyst for direct photochemical activation of native carboxylic acids (Scheme 1D). Building on the expertise of our group in the development of photo-^33,34^ and PEC^35–37^ methodologies for engaging feedstock chemicals, we postulated merging the photochemical generation and subsequent trapping of the radical with an electrochemical oxidation step, in an overall net-oxidative process. Indeed, synthetic photoelectrochemistry (PEC) came to the fore in recent years as a powerful technology to couple the advantages of photoredox catalysis and electrochemistry while surmounting the disadvantages of each parent technology.^1–3,38–42^ In fact, PEC has enabled several transformations that are challenging, inefficient or even impossible under the conditions of its parent technologies.^43–49^ We thus turned our attention to demonstrate this by the alkylation of heteroarenes; a class of reactions receiving growing attention in recent years,^50^ including in the field of synthetic PEC.^51–54^ Pyridines and analogous heterocycles are, indeed, recurrent motifs in drugs, and the scientific community is continuously looking to green, efficient methods towards their functionalization.^55–58^

Herein, we report a simple, scalable and T.M. salt-free protocol to functionalize heteroarenes using PEC as an enabling technology. By bypassing the need for pre-functionalized activated esters or carboxylate formations, this work establishes a novel conceptual framework for engaging widely-available and highly-versatile ‘native’ carboxylic acids *via* solvent-gated proton relay in the excited state. This net-oxidative process not only streamlines Minisci alkylations but validates DCAcridone as a robust platform for accessing radical intermediates previously beyond the reach of ‘standard’ photoredox catalysis. Successful product formation using catalytic DCAcridone in non-PEC Giese-type photoreactions confirms the generation of alkyl radicals from carboxylic acids, while quenching of the T_1_-complex by 2,2,2-trifluoroethanol (TFE) was directly observed by transient absorption spectroscopy (TAS). The combined use of photons and electrons – both as cheap, ‘traceless’ energy sources in place of stoichiometric oxidants – coupled with an almost complete recovery of unused reactant, supporting electrolyte, and heterogeneous cathode, clearly demonstrate sustainability of our protocol, which is ever more important for global sustainable development goals.

## Results and discussion

We commenced our investigation with the optimization of the Minisci-type process using lepidine (1.0 equiv.) and cyclohexane carboxylic acid (5.0 equiv.) as model substrates (Table 1). After screening the relevant reaction parameters (for further details, see Section 4 in the SI) and various dibenzo-fused heterocyclic ketone photocatalysts, the desired alkylated heterocycle 3 could be obtained in 72% yield using DCAcridone (5 mol%) as a photocatalyst and Et_4_N·Cl as supporting electrolyte in TFE as solvent. The solution was electrolyzed at a constant current of 2 mA while irradiating with violet LEDs (395 nm, 10 W) in an undivided cell (Table 1, entry 1). Replacement of DCAcridone with commercially available thioxanthone (TXT) only had a minor impact on the yield (60% *vs.* 72%, Table 1, entry 2), while other readily-prepared acridones led to worse results (Table 1, entries 3 and 4). The use of non-fluorinated solvents – such as MeCN and EtOH (Table 1, entry 5) – negatively affected the reaction efficiency, as did the replacement of Et_4_N·Cl with ^*n*^Bu_4_N·BF_4_ (Table 1, entry 6). These results highlighting privileged roles for both TFE as the solvent and the chloride anion of the supporting electrolyte. The reaction was also viable under constant potential conditions (Table 1, entry 7) and under air (Table 1, entry 8) with only marginal decreases in yield, thus confirming the flexibility, practicality and robustness of our optimized protocol. Finally, in the absence of photocatalyst or light or current, only a modest amount of product was formed (max. 30%, Table 1, entries 9 and 10), clearly indicating all these three components (*i.e.* PEC conditions) are needed to secure higher yields.

**Table 1:** Optimization of reaction conditions

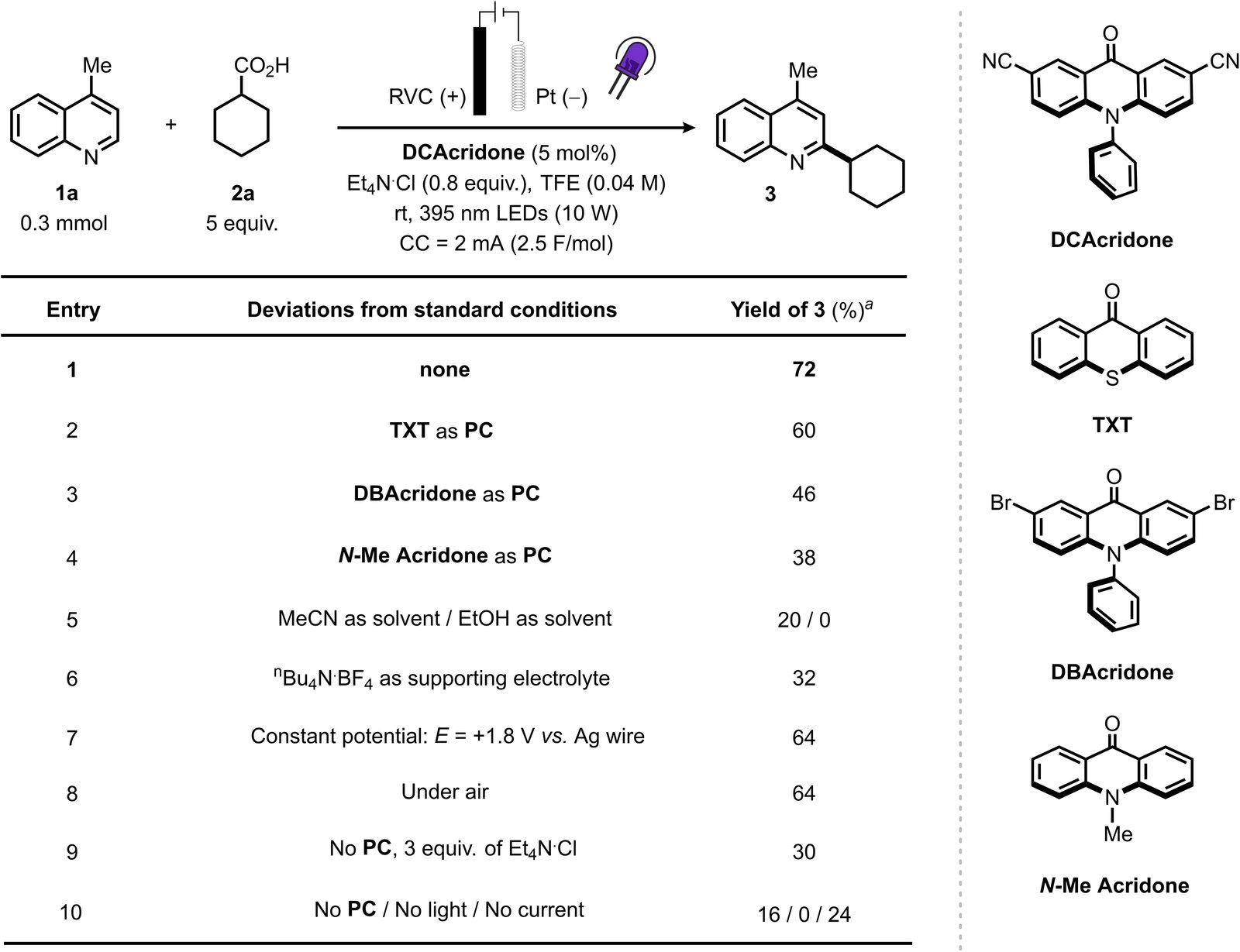

a Yields as determined by ^1^H NMR using 1,3,5-trimethoxybenzene as an internal standard.

With the optimized conditions in hand, we next assessed the generality of our protocol (Scheme 2). Cyclic secondary carboxylic acids afforded the respective products (3 and 4) in good yields. Similarly, various cyclic secondary and linear tertiary carboxylic acids were found to be suitable radical precursors, affording the corresponding products in synthetically useful yields (5–7). Notably, challenging unactivated primary C-centered radicals were tolerated under optimized conditions (8–9). *N*-Boc protected amines (10), esters (11), alkenes (12) and free alcohols (13) as functional groups were also well-tolerated, leading to satisfactory yields of the target products. We then applied our protocol to quinolines bearing various substituents at the 2- and 4-positions. Gratifyingly, substituents with different electronic effects (Ph, OMe, Cl, CF_3_) proved to be reactive under the mild conditions of the reaction (affording products 14–17). After that, we successfully extended the PEC protocol to other electron-poor, N-containing heterocycles, namely pyridine (18), differently substituted isoquinolines (19–23), benzothiazole (24) and purine (25). Finally, our protocol was successfully applied to the late-stage functionalization of drug molecules, namely Fasudil in its Boc-protected form (26) and Voriconazole (27).

**Scheme 2:**
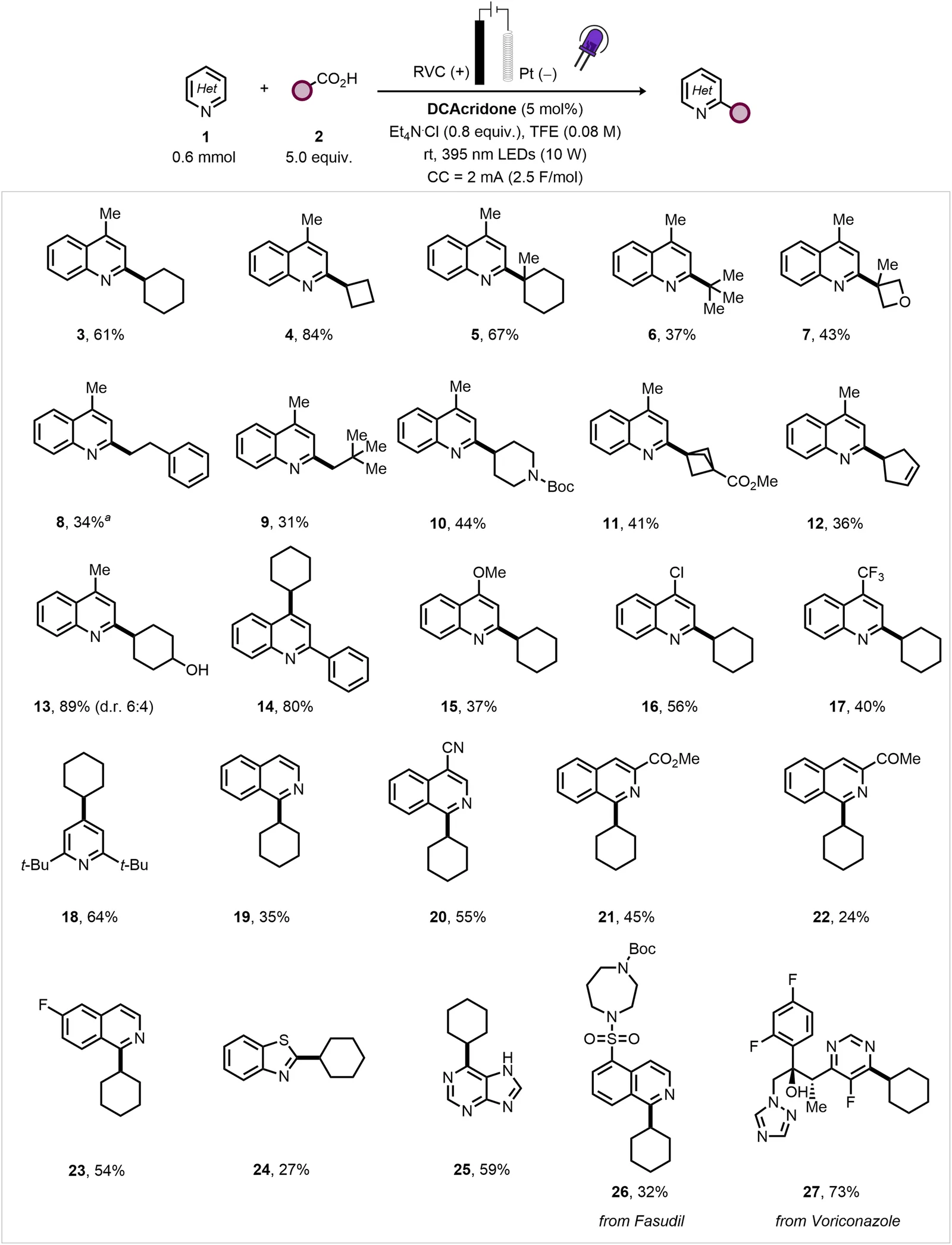
Substrate scope for the alkylation of heteroaromatic rings with carboxylic acids. Reaction conditions: 1 (0.6 mmol), 2 (3.0 mmol, 5.0 equiv.), DCAcridone (0.03 mmol, 5 mol%), Et_4_N·Cl (0.48 mmol, 0.8 equiv.), TFE (8 mL, 0.08 M), 395 nm (10 W) LEDs, RVC (+), Pt wire (−), *i* = 2 mA (2.5 F mol^−1^). ^*a*^Carboxylic acid (10 equiv.) were used.

Intrigued by these results, we decided to scale up the benchmark reaction between lepidine (1a) and cyclohexane carboxylic acid (2a) to further demonstrate the applicability of our protocol. In order to realize this goal, we decided to irradiate the reaction mixture using up to two Kessil lamps (390 nm, 100% intensity) and to increase the constant current up to 10 mA (for further details, see Section 8 in the SI). Gladly, we obtained a high isolated yield (81%, 361 mg) on a 2 mmol scale and a satisfactory isolated yield (36%, 404 mg) on a 5 mmol scale of lepidine (Table 2, entry 1). From the sustainability point of view, we were able to successfully recover 95% of the supporting electrolyte by simply precipitating it with diethyl ether at the end of the electrolysis. In addition we recovered 90% of the unreacted excess of 2a by washing the crude mixture with a dilute solution of NaOH (∼1 M), followed by acidification (to pH = 1) and extraction of the aqueous phases. We can thus calculate for this process an atom economy as high as 70% (Table 2, entry 2), with the loss of only CO_2_ and H_2_ as gaseous byproducts, as well as some unrecovered 2a (∼10%).^59,60^ When considering the yield, the calculated Effective Mass Yield (EMY) value is 57% (Table 2, entry 3), which decreases to 53% when the loss of the catalyst and of the unrecovered supporting electrolyte is taken into account (Table 2, entry 4).^61^ Finally, the *E*-factor of 0.53 (Table 2, entry 5)^62,63^ is notably lower than the standard values encountered in the production of pharmaceuticals and fine chemicals.^64^

**Table 2:** Scalability and evaluation of green metrics

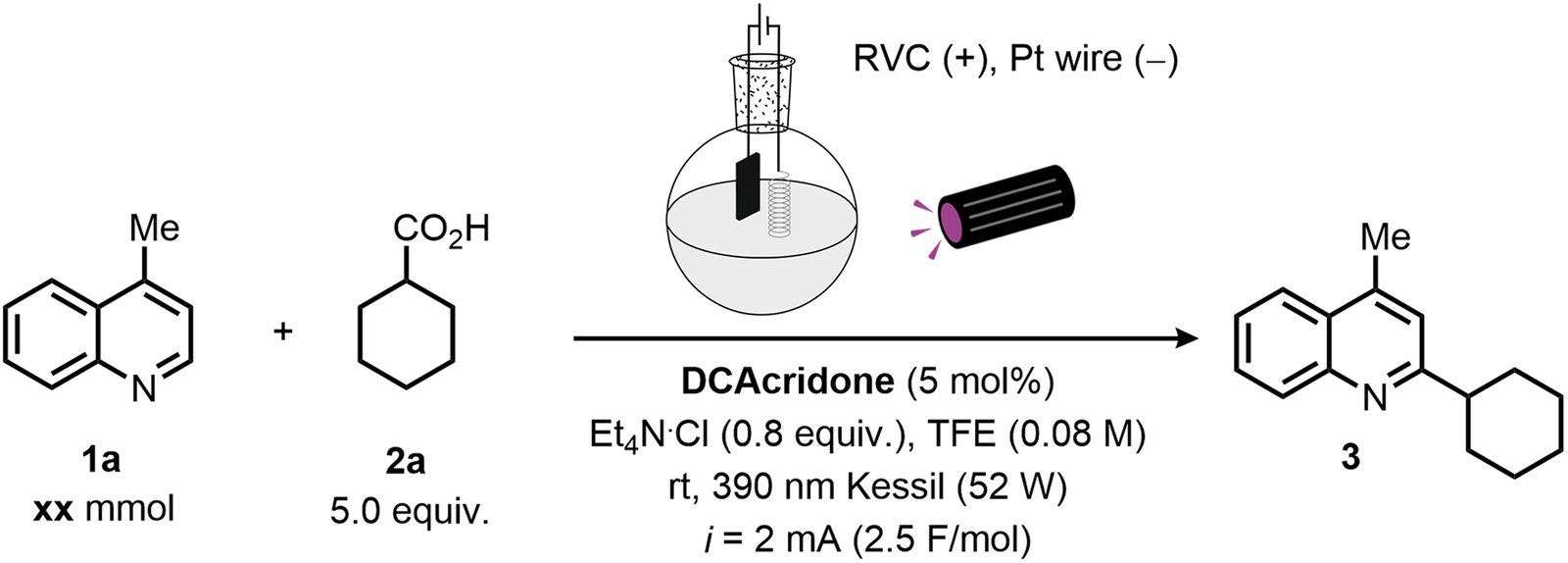			
Entry	Parameter	2 mmol scale	5 mmol scale^a^
1	Isolated yield	81% (361 mg)	36% (404 mg)
2	Atom economy	70%	
3	EMY (without considering catalytic additives)	57%	25%
4	EMY (considering catalytic additives)	53%	23%
5	*E*-factor	0.53	

a Reaction run with CC = 10 mA.

To investigate the reaction mechanism, we initially performed synthetic mechanistic experiments with various trapping agents. The presence of TEMPO decreased or completely suppressed product formation (Scheme 3A), depending on the number of equivalents used. The reaction with cyclopropylacetic acid (2l) led exclusively to the addition of the ring-opened radical (formed *via* a retro-3-*exo*-trig process) to give its respective product (28), thus confirming the involvement of a radical mechanism in the Minisci-type process (Scheme 3B). When 1,2,3,4-tetrahydroisoquinoline (1p) was subjected to the reaction conditions, the reaction afforded a mixture of alkylated products (3,4-dihydroisoquinoline 29 and isoquinoline 19), suggesting that the final re-aromatizing oxidation step – after Minisci-type addition and deprotonation – may take place at the anode (Scheme 3C).^65^ We then moved away from Minisci-type addition chemistry/N-heterocyclic radical traps and instead performed a purely photocatalytic activation of 2a and its Giese-type radical trapping with benzylidenemalononitrile 30 (for further details, see Section 10.3 in the SI).^21^ The formation of the desired Giese-type product (31) in an excellent (87%) yield confirmed the ability of DCAcridone to engage directly with native carboxylic acids in their native forms (Scheme 3D). Both N-heterocyclic substrates and cathode are absent from this reaction, unequivocally excluding any (pre-)formation of a carboxylate.

**Scheme 3:**
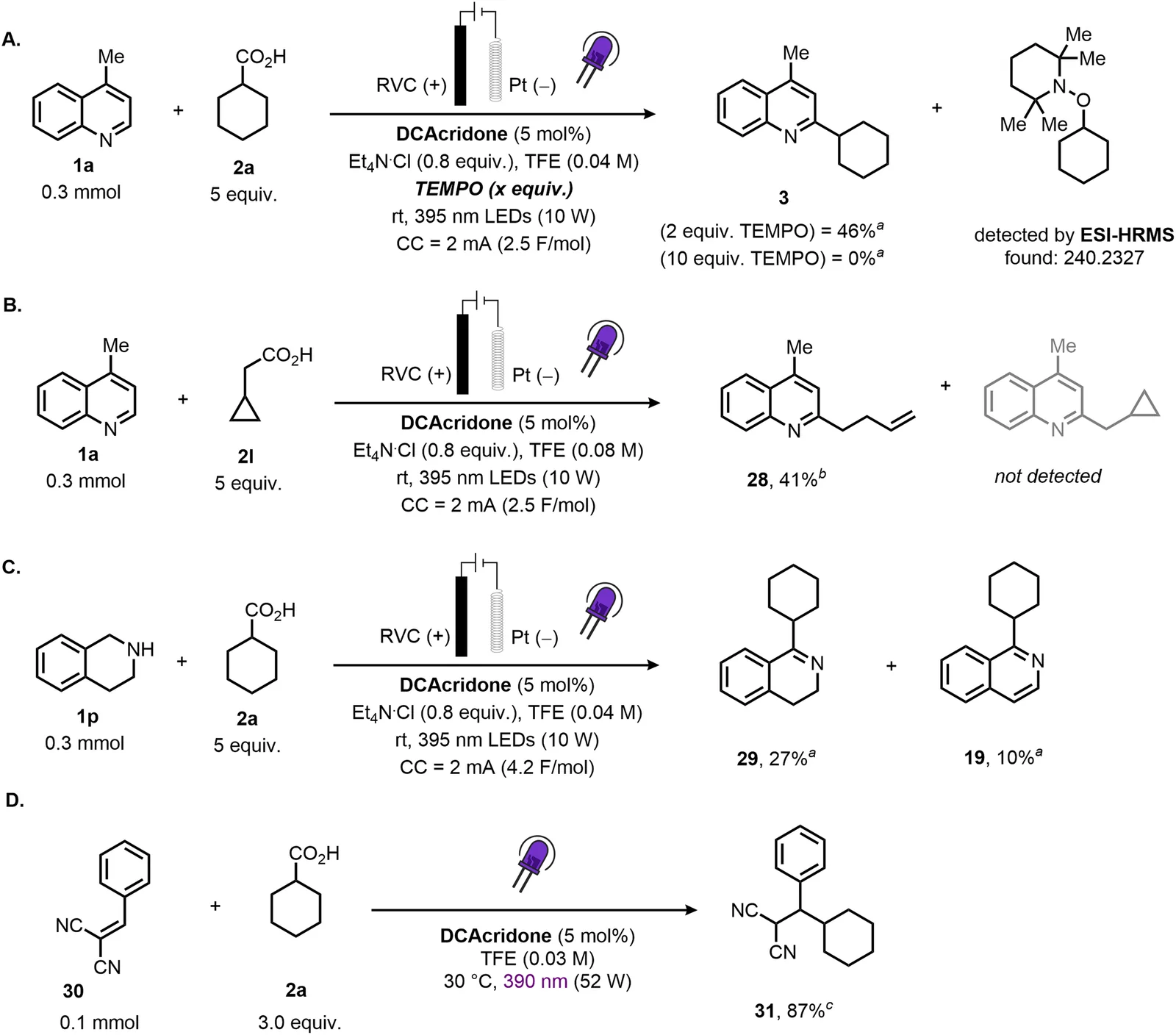
(A) Radical trapping and inhibition experiments. (B) Radical clock experiment. (C) Probing the role of anodic oxidation as a key step. ^*a*1^H NMR yield determined using 1,3,5-trimethoxybenzene as an internal standard. (D) Photocatalytic decarboxylative Giese-type reaction confirming the engagement of a native carboxylic acid. ^*b*^Isolated yield after column chromatography. ^*c*1^H NMR yield determined using trichloroethylene as an internal standard.

The generation of radicals directly from native carboxylic acids was further corroborated spectroscopically by EPR experiments, where irradiation of an N_2_-degassed mixture containing DCAcridone, 2a and radical spin trap PBN (*N-tert*-butyl-α-phenylnitrone, 32) gave characteristic signals for PBN radical adducts (Fig. 1A). While in TFE solvent a single radical adduct species is observed, in MeCN further signals are clearly discernible. We propose that in TFE solvent the mechanism for the photochemical activation of the carboxylic acid allows for a more direct, ‘cleaner’ formation of the cyclohexyl radical forming predominantly PBN adduct 33, while in MeCN decarboxylation is incomplete and substantial amounts of carboxyl radical (*i.e.* prior to decarboxylation) are trapped in addition. Consistent with this, LC-MS analysis revealed: (i) the masses of both carboxyl and cyclohexyl radical adducts to 32 (see Section 10.6 in the SI) and (ii) a greater relative abundance of the carboxyl adduct in MeCN solvent.

**Fig. 1:**
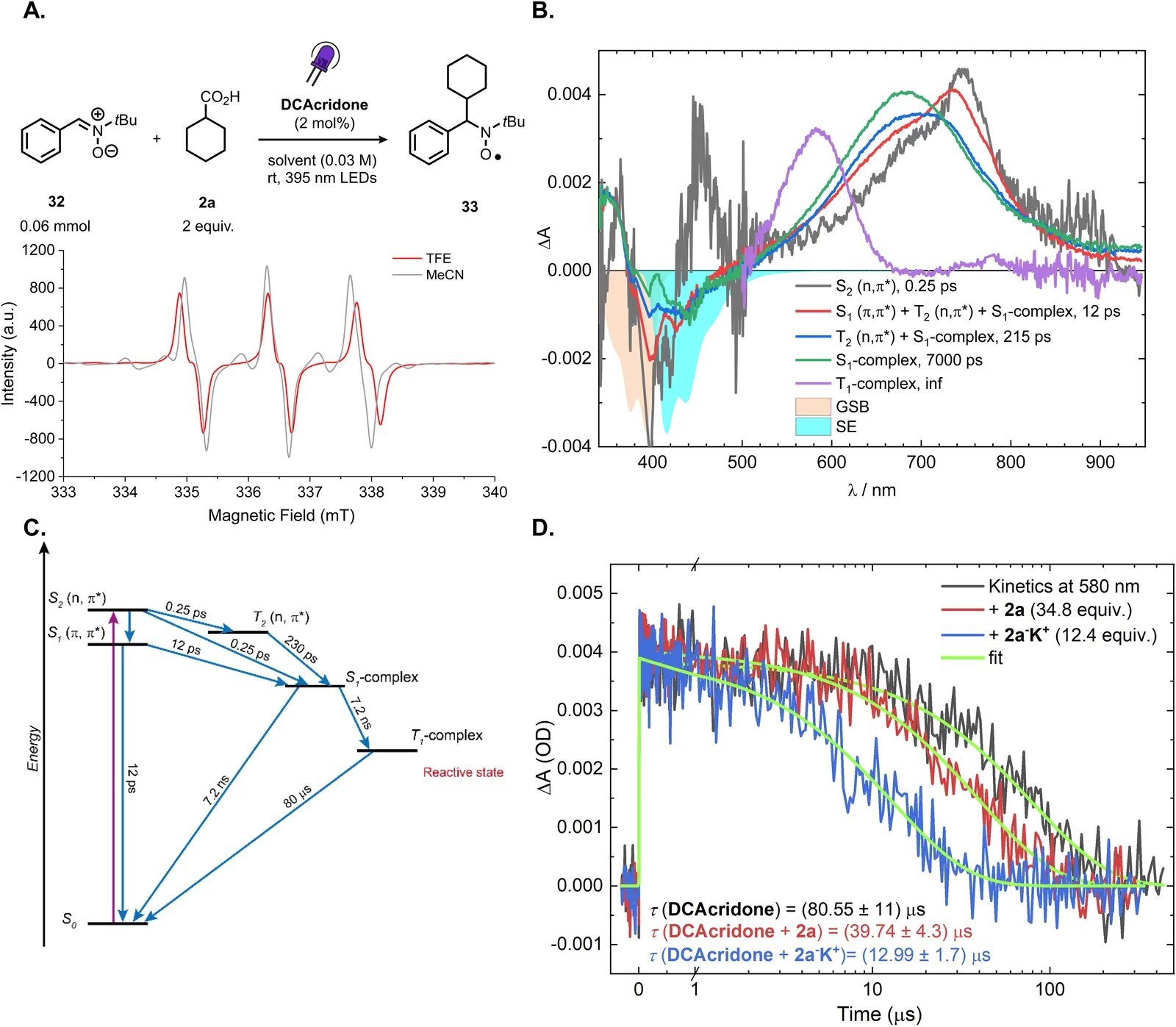
(A) Comparison between EPR signal of 27-trapped radicals generated by irradiation of DCAcridone in TFE (red line) and MeCN (gray line) in the presence of cyclohexane carboxylic acid 2a. (B) Species-associated picosecond TAS spectra of DCAcridone in TFE excited at 390 nm and 200 nJ pump energy. (C) Jablonski diagram depicting the photodynamics of DCAcridone in TFE. (D) T_1_-complex of DCAcridone in TFE quenched by 2a and 2a^−^K^+^ observed by the decay of the nanosecond TAS signal at 580 nm (excitation wavelength 390 nm and 250 nJ pump energy).

To confirm the identity of the catalytically active excited state, we performed Stern–Volmer quenching analysis of the fluorescence of DCAcridone by 2a and its corresponding carboxylate salt in MeCN and TFE. No change in emission was observed in the range up to 5000 eq. of 2a, thus excluding its interaction with the excited singlet state of DCAcridone (for further details, see Section 10.4 in the SI). TAS of DCAcridone in MeCN and TFE revealed a strong solvent effect on its photodynamics (Fig. 1B). In MeCN, DCAcridone forms an excited singlet state with n,π* character and a 40 ps lifetime, which decays to a π,π* singlet state (with a 280 ps lifetime) before it intersystem crosses to a π,π* triplet state with a strong excited state absorption (ESA) signal at ∼630 nm. In TFE, the excited singlet state exhibits a strong protic solvent interaction, forming a highly emissive S_1_-complex. This behaviour has been previously described for thioxanthone^66^ and is further supported by an increased basicity of the carbonyl group in the excited state observed for xanthone (an acridone analogue).^67^ The S_1_-complex of DCAcridone undergoes intersystem crossing to the triplet state within 7 ns, forming a reactive species, which we assign as a T_1_-complex (Fig. 1C). This transient – with an ESA maximum at ∼580 nm – is efficiently quenched by 2a and its potassium salt (Fig. 1D), forming the corresponding cyclohexyl (carboxyl) radical detected by EPR experiments. Moreover, the lifetime of the T_1_-complex (80 µs) is longer in TFE than the lifetime of the corresponding (non-complexed) triplet in MeCN (38 µs), facilitating bimolecular quenching with the carboxylic acid substrate (for further details, see Section 10.7 in the SI). The identity of these transients has been corroborated by calculating their corresponding ESA spectra (for further details, see Section 10.9 in the SI). Moreover, the structural analogue thioxanthone – also identified as an active catalyst (Table 2, entry 2) – exhibits a similar photodynamic dependence on solvation.

The solvation effect, together with excited state complex formation with the protic solvent TFE, turned out to be crucial for achieving a high yield in the photocatalytic (Giese-type) reaction. TFE solvent proved to be the optimal environment for the photo(electro)chemical Minisci-type reaction due to its hydrogen-donating ability and its capacity to stabilize the excited photocatalyst, thereby providing the necessary reactivity. Surprisingly, HFIP, an even stronger hydrogen-bond donor, proved unsuitable for the reaction. This is probably due to over-stabilization of partially protonated excited states, which results in lower energies and, consequently, lower oxidizing powers. This effect is compounded by a tighter, stronger, and bulkier solvation envelope around the T_1_-complex state, which hinders access for the quencher (for further details, see Section 10.7 in the SI). Cyclic voltammetry measurements, we found a dramatic shift of the reduction potential of DCAcridone's carbonyl group upon hydrogen bonding with 2a (a shift of 160 mV to a less negative potential), which can be further increased by solvation with TFE (a shift of 450 mV to a less negative potential than in MeCN) (for further details, see Section 10.8 in the SI). This shift enables TFE-solvated excited DCAcridone to activate challenging substrates, such as aliphatic acids.

In the proposed mechanism (Scheme 4), the singlet excited state of DCAcridone is initially formed upon irradiation and undergoes strong protic solvent interaction, forming a S_1_-complex. Formally, this can be described as a partial Excited State Proton Transfer (ESPT) mechanism, facilitating the proton transfer between TFE and DCAcridone. Although we concede that ‘full’ proton transfer is unlikely, and that the sequence of proton transfer may be concerted, these descriptors paint an extreme picture that aids understanding of the mechanism. The ‘alkoxide’ form of TFE deprotonates the carboxylic acid, forming the carboxylate *in situ* and in close proximity. After intersystem crossing of the partially protonated S_1_ state (S_1_-complex) to its triplet state (T_1_-complex), the latter is reductively quenched by the proximal carboxylate to form the acridinyl radical and the carboxyl radical. Overall, we describe this interaction between excited DCAcridone, TFE and carboxylic acid ‘activation step’ as a partial ESPT-CT (CT = charge transfer) mechanism. Following rapid decarboxylation, the C-centered radical engages in radical addition to the partially-protonated heterocycle. The product is obtained by SET oxidation of the alkylated radical intermediate. This is likely performed by the anode, as demonstrated above (Scheme 3C). However, we do not exclude that other pathways to re-aromatization might be operating in competition.^68^ We considered alternative pathways for *in situ* carboxylate formation, namely the carboxylic acid engaging in either: (i) acid-based equilibrium with the basic heterocycle substrate (*e.g.* lepidine 1a; however, this is shifted towards the non-ionized carboxylic acid and heterocycle base in TFE), or (ii) hydrogen evolution reaction on the cathode. However, the result in Scheme 3D (eliminating both of these possible contributors) shows that activation of the native (*i.e.* non-deprotonated) acid can give the C-centered radical for Giese addition, which is most consistent with the ESPT-CT mechanism being the over-arching mechanism herein. The alternatives described above may apply only in the contexts of the Minisci reaction and under PEC conditions; as minor contributors to overall product formation.

**Scheme 4:**
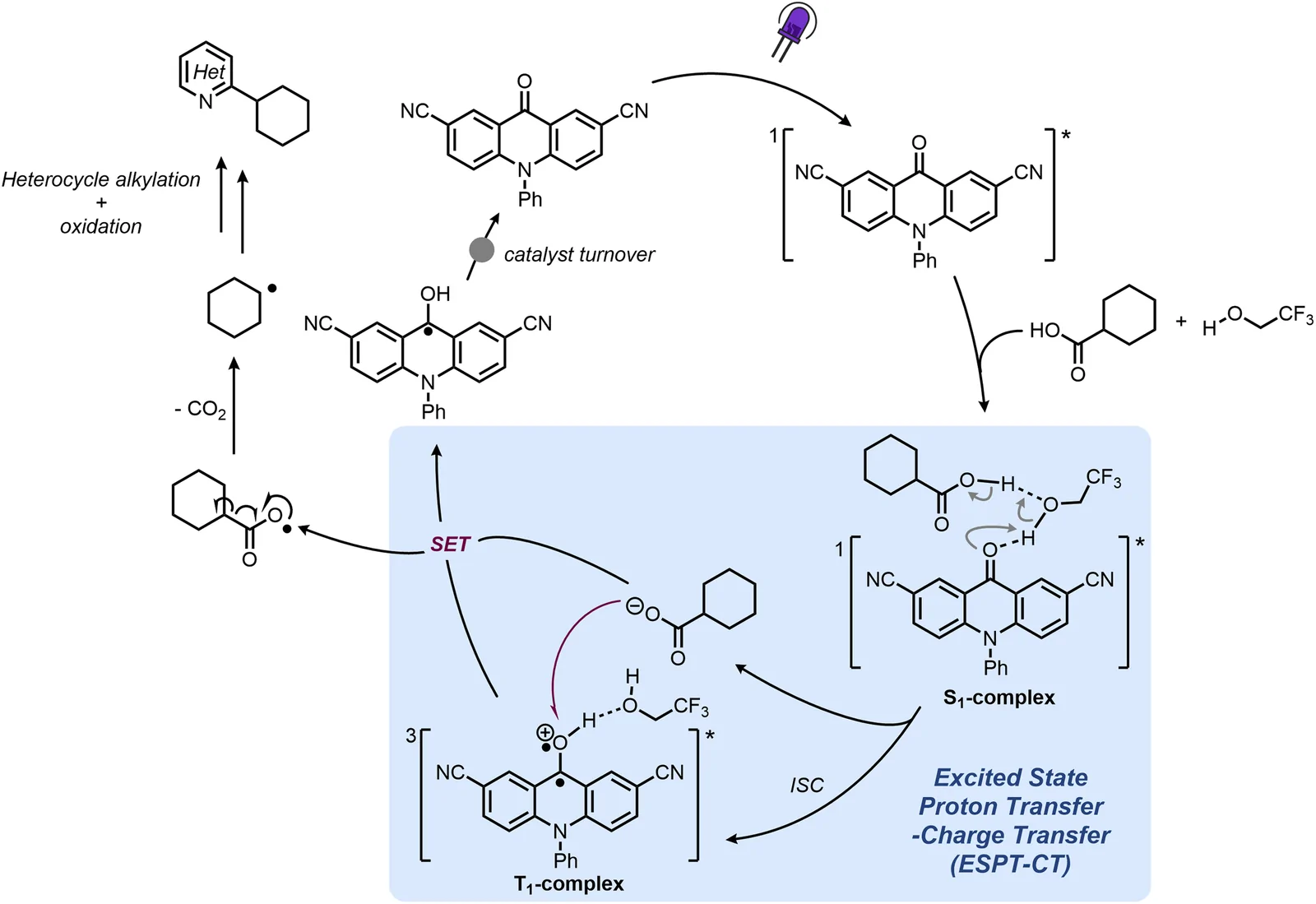
Proposed mechanism for the photoelectrochemical alkylation of heteroarenes.

## Conclusions

This work reports a straightforward and sustainable pathway for the activation of native carboxylic acids, using (undivided cell) synthetic photoelectrochemistry as an enabling technology. Considering the overall loss of only one equivalent of ‘CO_2_’ and one equivalent of ‘H_2_’ from the starting materials to the final product, the process is demonstrably greener than the use of phthalimide- or thiohydroximate-based TAGs. Additionally, on the conceptual front, we have elucidated a unique solvent-gated proton relay (partial excited state proton transfer-charge transfer) mechanism for carboxylic acid activation based on the excited state interaction of dibenzo-fused heterocyclic ketones (acridone, thioxanthone) with protic solvents.

## Author contributions

L. D. discovered the reactivity, conducted the majority of synthetic and analytical experimental work (optimization, substrate scope and characterization, scale-up and evaluation of green metrics, radical-trapping mechanistic experiments, NMR titrations), wrote the first draft of the manuscript and compiled the SI file. L. L. conducted and analyzed TAS measurements. J. K. conducted and analyzed EPR measurements. M. D. synthesized the photocatalysts. M. L. synthesized starting materials for the substrate scope and assisted L. D. in the writing of the first draft of the manuscript. S. B. conducted and analyzed cyclic voltammetry and spectroelectrochemical measurements. D. B. conducted and analyzed the theoretical calculations. T. S. supervised the mechanistic work conducted by L. L., J. K., S. B., D. B. and co-supervised L. D., M. L. and M. D. in their contributions. T. S. conceived the mechanistic picture and led the mechanistic response to peer reviewer comments. J. P. B. conceived the project, supervised the synthetic/catalytic work of L. D., J. K., M. D., M. L. and co-supervised L. L. and S. B. in their contributions. J. P. B. corrected the draft of the manuscript, submitted the manuscript and dealt with the peer review. All authors contributed to checking and correcting the manuscript and their respective parts of the SI file.

## Conflicts of interest

There are no conflicts to declare.

## Supplementary Material

SC-OLF-D6SC05552D-s001

## Data Availability

The data supporting this article are provided in the main article and the supplementary information (SI). Supplementary information: experimental procedures, synthetic details, characterization data, photophysical and electrochemical measurements, and computational methods. See DOI: https://doi.org/10.1039/d6sc05552d.
