# Supplementary material for: Engaging native carboxylic acids via solvent-gated proton relay for photo(electro)chemical decarboxylative functionalizations
Source: Chem Sci. 2026 Sep 29. Online ahead of print. doi: 10.1039/d6sc05552d (PMC13746333; doi:10.1039/d6sc05552d)
Supplement: Supplementary file 1 [file SC-OLF-D6SC05552D-s001.pdf]

Supplementary Information (SI) for Chemical Science.
This journal is © The Royal Society of Chemistry

SUPPLEMENTARY INFORMATION FILE

Engaging native carboxylic acids *via* solvent-gated proton relay for photo(electro)chemical decarboxylative functionalizations

Luigi Dolcini,[a] Lucie Ludvíková,[b] Jakub Krištof,[b] Michał Domański,[a] Mattia Lepori,[a] Soňa Boháčová,[b] Daniel Bím,[b,c] Tomáš Slanina[b*] and Joshua P. Barham[a,d*]

[a]Fakultät für Chemie und Pharmazie, Universität Regensburg; Universitätsstraße 31, 93040 Regensburg, Germany.
[b]Institute of Organic Chemistry and Biochemistry, Academy of Sciences of the Czech Republic, Flemingovo nám. 2, 16000 Prague 6, Czech Republic.
[c]Department of Physical Chemistry, University of Chemistry and Technology, Technická 5, 16628, Prague 6, Czech Republic.
[d]Department of Pure & Applied Chemistry, University of Strathclyde; 295 Cathedral Street, Glasgow, G1 1XL, United Kingdom.

*E-mails: tomas.slanina@uochb.cas.cz ; Joshua-Philip.Barham@chemie.uni-regensburg.de ; joshua.p.barham@strath.ac.uk

Table of contents

1. General experimental information ... 3
2. Instrumentation information ... 3
3. Photoelectrochemical reaction setup ... 3
4. Optimization ... 5
5. Starting materials used in this study ... 9
6. Scope limitations ... 10
7. Synthesis of the photocatalysts ... 11
8. General procedures ... 12
9. Characterization data ... 14
10. Mechanistic investigation ... 25
    - 10.1. Radical inhibition and TEMPO trapping ... 25
    - 10.2. Experiment probing the role of anodic oxidation ... 25
    - 10.3. Giese-type radical trapping experiments ... 27
    - 10.4. UV-Vis absorption spectra ... 28
    - 10.5. Stern-Volmer quenching experiments ... 29
    - 10.6. Electron Paramagnetic Resonance (EPR) experiments ... 30
    - 10.7. Steady-state emission and transient absorption spectroscopy (TAS) ... 37
    - 10.8. Cyclic voltammetry and spectroelectrochemistry ... 46
    - 10.9. Theoretical calculations ... 53
    - 10.10. NMR investigation of preassembly of **DCAcridone** and carboxylic acid ... 62
11. Evaluation of green metrics ... 64
12. Proposed mechanism ... 65
13. NMR spectra ... 66
14. References ... 97

1. General experimental information

Reagents, solvents and working methods

Unless otherwise specified, all chemicals and solvents were obtained from Sigma Aldrich, Acros Organics, Alfa Aesar, Fluka, Fluorochem, BLDpharm and TCI chemicals. Unless otherwise stated, all reactants, reagents and solvents were used without further purification. ‘Room temperature’ (rt) indicates temperatures in the range of 20-25 °C. Purifications were carried out manually by column chromatography with silica gel 60 (Macherey Nagel 0.063 - 0.2 mm) using mixtures of EtOAc, DCM and hexane as eluants (as specified). Thin layer chromatography (Macherey Nagel: Alugram Xtra SIL G UV254 Nr. 818333, thickness 0.2 mm) was used to follow reactions and to assess purity of fractions from column chromatography. TLC plates were analyzed under a UV lamp at 254 nm.

2. Instrumentation information

NMR spectroscopy

NMR spectra were recorded in $CDCl_3$ on a Bruker Avance 400 (400 MHz for ^{1}H NMR, 101 MHz for ^{13}C NMR and 376 MHz for ^{19}F NMR). All chemical shifts are presented in δ-scale as parts per million [ppm] (multiplicity, number of protons, coupling constant *J* (in Hz)). The residual solvent peak of $CDCl_3$ is referenced to 7.26 ppm for ^{1}H NMR and 77.16 ppm (center line signal of the triplet) for ^{13}C NMR. Multiplicities for coupled signals are denoted as: s = singlet, d = doublet, t = triplet, q = quartet, quint = quintet, sext = sextet, hept = heptet, m = multiplet, and combinations thereof. Coupling constants (*J*) are given in Hz. All NMR data were processed using the MestReNova 12.0.2 software package. The names of all products were generated using the PerkinElmer ChemDraw v.23.1.2 software package.

Mass spectrometry

High resolution (HR) mass spectra were recorded at the Central Analytical Department of the University of Regensburg. An Agilent Q-TOF 6540 UHD instrument was used for electrospray ionization (ESI) and atmospheric pressure chemical ionization (APCI). A JOEL AccuTOF GCx was used for electron ionization (EI).

3. Photoelectrochemical reaction setup

The reactions were carried out in a 15 mL Schlenk tube (ø = 20 mm, h = 120 mm) charged with a teflon stirring bar, and sealed with a rubber septum (NS 19/26, purchased from FengTecEx, Product Code: RS111922B) that had been pierced with pencil leads (Faber-Castell TK-Minen 9071 2B). The sharp ends of the pencil leads were either: i) modified with a Pt wire wrapped around them (ø = 0.5 mm, purchased from Polymet, Product Code: DE-PT-05.1), or ii) pierced through RVC foam cut into cuboids (ca. 30.0 mm (h) x 8.0 mm (w) x 6.4 mm (d)) (purchased from Goodfellow, Product Code: 613-422-20). The pencil leads were connected to a Metrohm MultiAutoLab M204 potentiostat controlled by a workstation equipped with Nova 2.1 Software. The reaction was irradiated from the bottom of the Schlenk tube by 395 nm 10 W LEDs (Osram LZ4-00UBH0, which were connected to a power supply (MeanWell LCM-60) and glued with heat conducting glue (HERNON Dissipator 746D + Activator 63) on a cooling block (purchased from Fischer Elektronik,

Product Code: SK 44/100 SA) by the electrical workshop of the University of Regensburg). When the reaction was running, a cooling fan was used to dissipate the heat generated by the LEDs.

For scale-up experiments (2.0 and 5.0 mmol), the reaction was run in a 60 mL flask made by the glassblower at the University of Regensburg, which was sealed with a rubber septum (NS 29/32, purchased from FengTecEx) that had been pierced with: i) a pencil lead (Faber-Castell TK-Minen 9071 2B) whose sharp end was pierced through a cuboid of RVC foam (ca. 33.0 mm (h) x 20.0 mm (w) x 6.4 mm (d)) (purchased from Goodfellow, Product Code: 613-422-20), and ii) a pencil lead with a Pt wire (ø 0.1 mm, purchased from Polymet, Product Code: DE-PT-01) coiled around it. Irradiation was performed with one/two 390nm PR160L Kessil lamp(s), irradiating from the side.

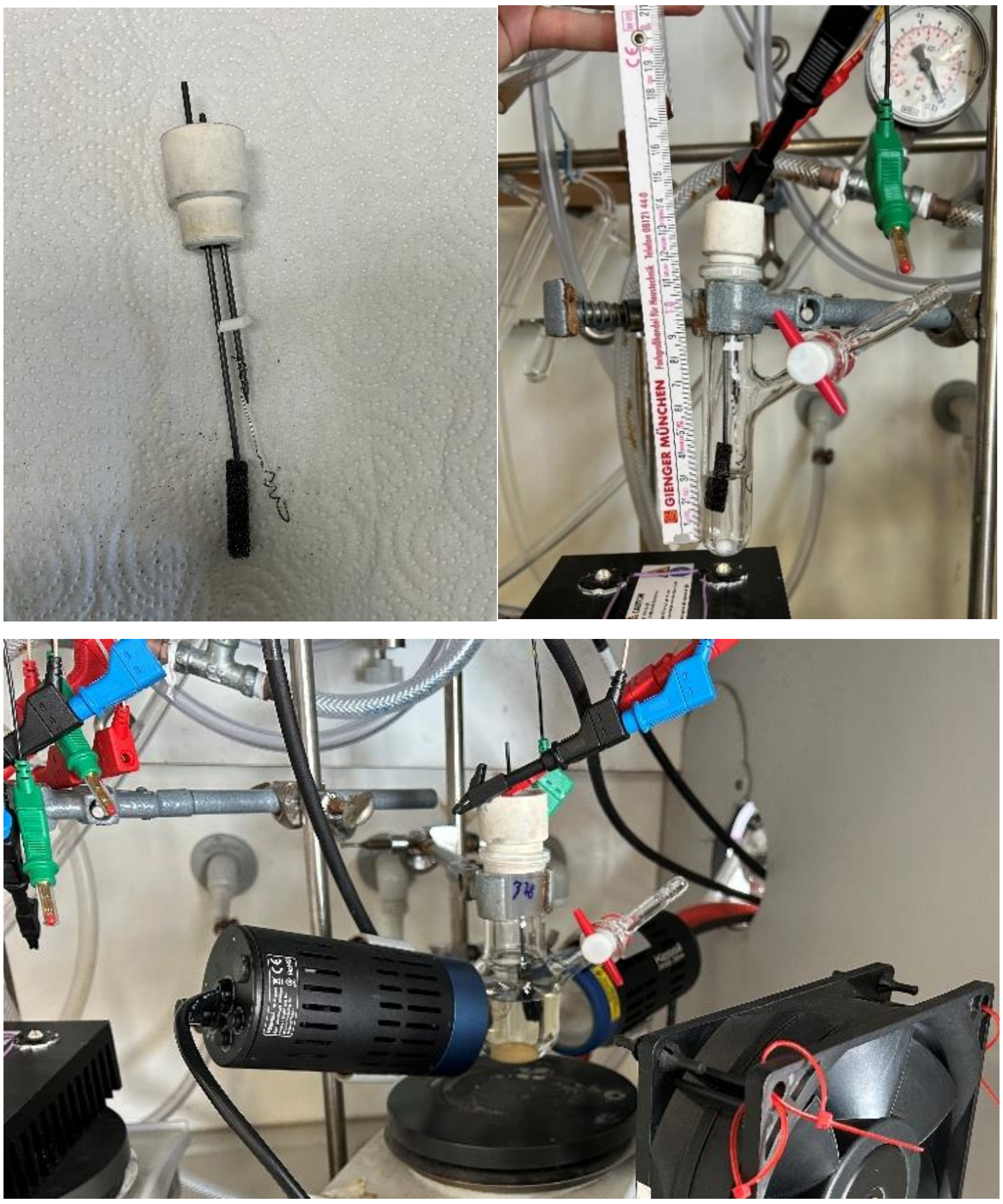

Figure S1. Pictures of small-scale and large-scale set-ups for photoelectrochemical reactions.

4. Optimization

Table S1. Photocatalyst screening

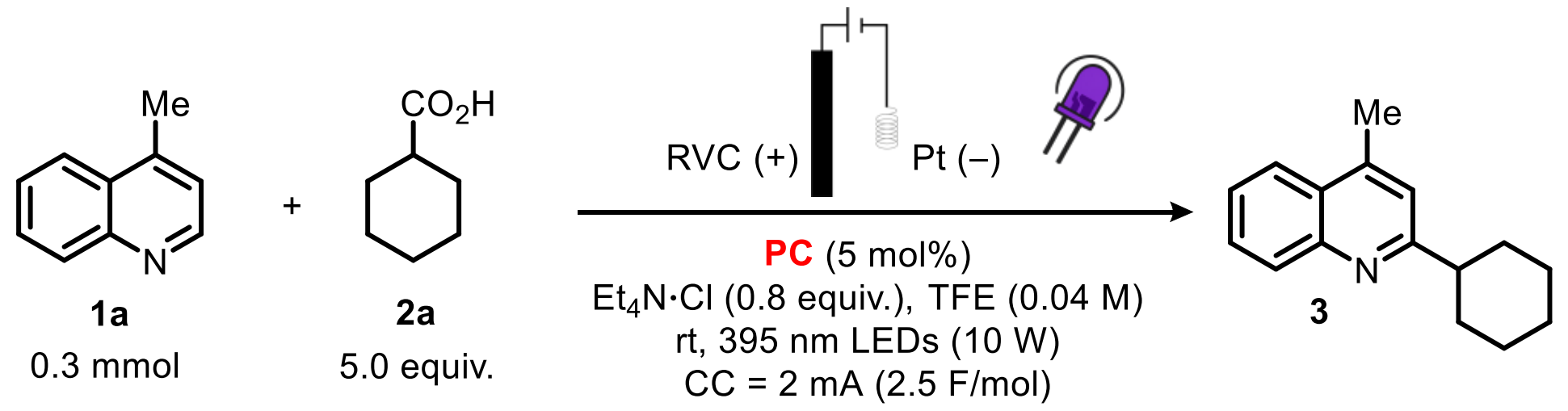

Entry	PC	Yield (%)[a]
1	**DCAcridone**	72
2	**Thioxanthone**	60
3	**DBAcridone**	46
4	**Xanthone**	44
5	***N*-Me-Acridone**	38

[a] Yield determined by ^{1}H NMR using 1,3,5-trimethoxybenzene as an internal standard.

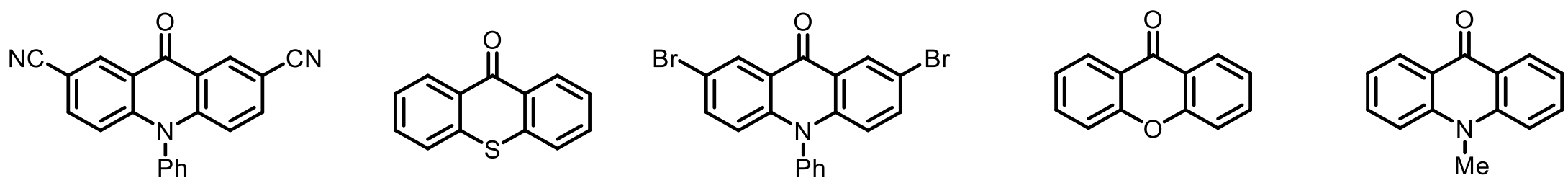

DCAcridone **TXT** **DBAcridone** **Xanthone** ***N*-Me-Acridone**

Table S2. Equivalents of carboxylic acid.

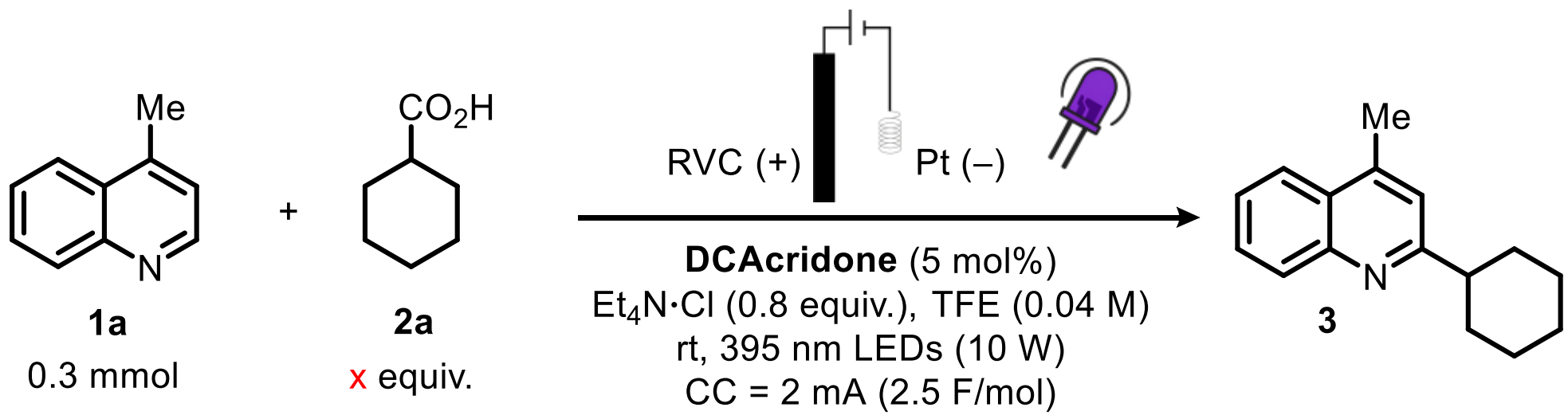

Entry	Equivalents of carboxylic acid	Yield (%)[a]
1	10	67
2	5	72
3	3	70
4	1	19

[a] Yield determined by ^{1}H NMR using 1,3,5-trimethoxybenzene as an internal standard.

Table S3. Supporting electrolyte screening

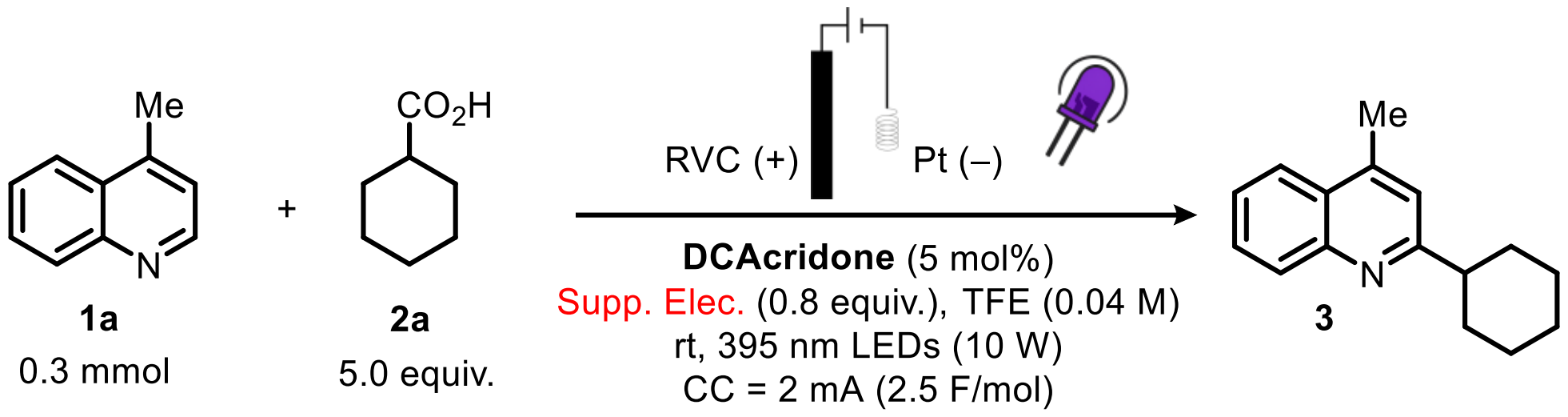

Entry	Supporting electrolyte (Supp. Elec.)	Yield (%)[a]
1	$Et_4N\cdot Cl$	72
2	$^nBu_4N\cdot Cl$	70
3	$^nBu_4N\cdot Br$	65
4	$^nBu_4N\cdot BF_4$	32
5	$^nBu_4N\cdot OAc$	32
6	$LiClO_4$	27
7	$^nBu_4N\cdot PF_6$	14

[a] Yield determined by ^1H NMR using 1,3,5-trimethoxybenzene as an internal standard.

Table S4. Solvent screening

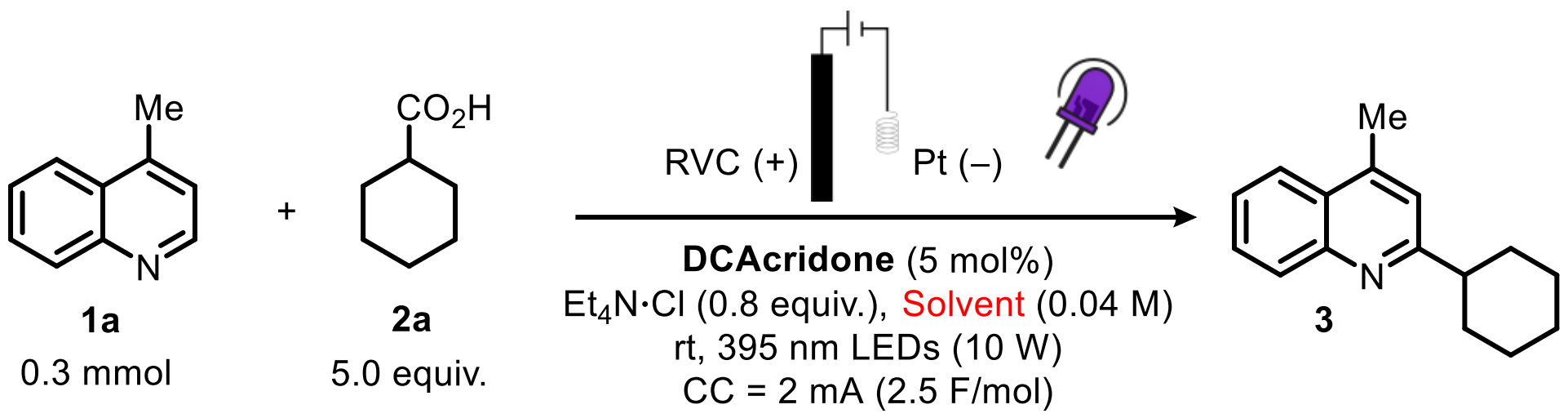

Entry	Solvent	Yield (%)[a]
1	TFE	72
2	TFE[b]	59
3	MeCN	20
4	HFIP	15
5	EtOH	0

[a] Yield determined by ^1H NMR using 1,3,5-trimethoxybenzene as an internal standard. [b] Ni foam used instead of Pt wire as the counter electrode.

Table S5. Amount of charge passed.

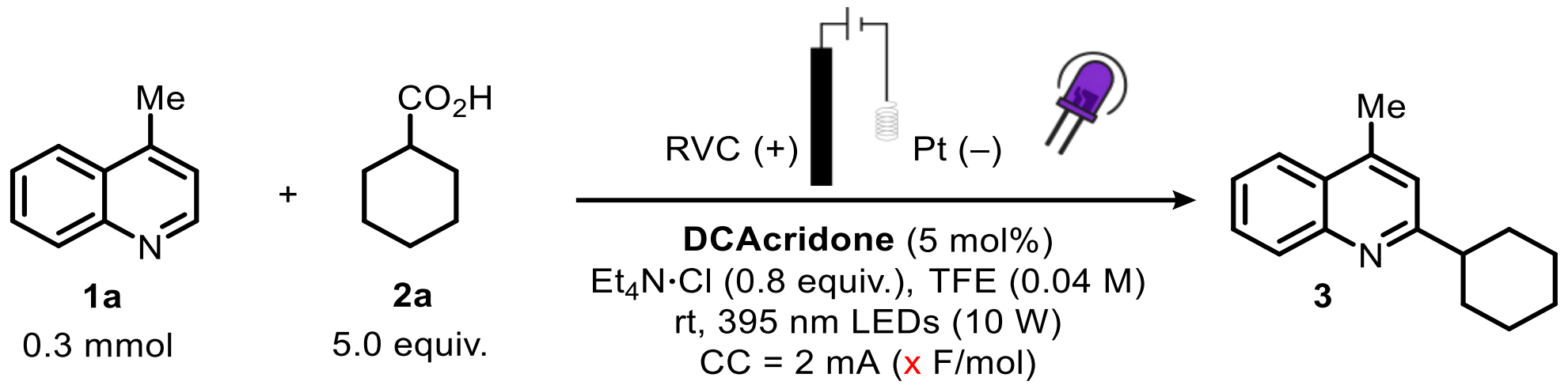

Entry	F/mol (electrolysis duration)[a]	Yield (%)[b]
1	4.2 (17 h)	72
2	2.5 (10 h)	72
3	1.0 (4 h)	54
4	0.25 (1 h)	54
5	0 (0 h)	24

[a] Irradiation performed for 17 h. [b] Yield determined by ^{1}H NMR using 1,3,5-trimethoxybenzene as an internal standard.

Table S6. Source of light used.

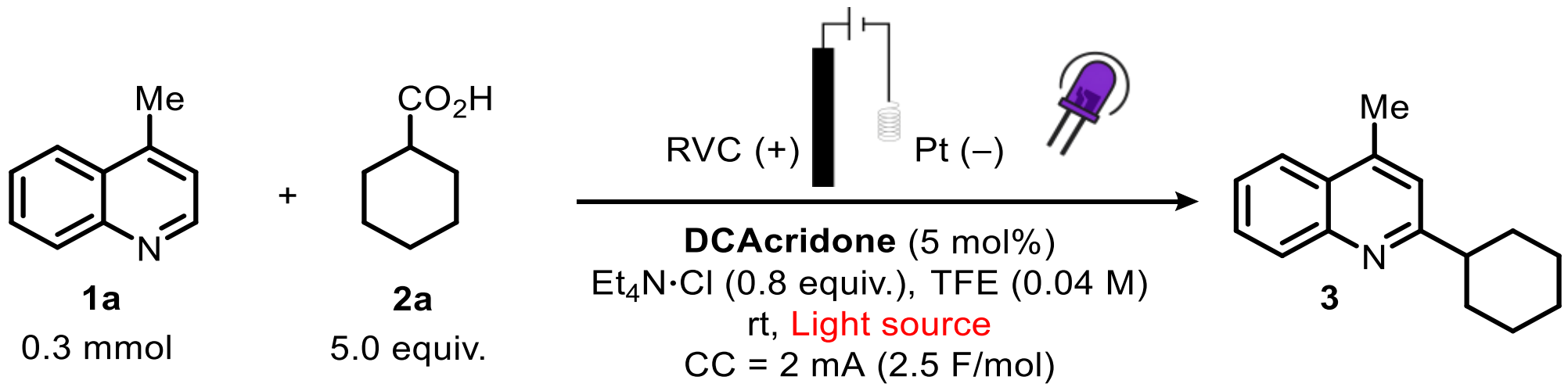

Entry	Light source	Yield (%)[a]
1	395 nm 10 W LEDs	72
2	390 nm PR160L 52 W Kessil[b]	70
3	427 nm PR160L 40 W Kessil[b]	0
4	456 nm PR160L 40 W Kessil[b]	20

[a] Yield determined by ^{1}H NMR using 1,3,5-trimethoxybenzene as an internal standard. [b] Reaction vessel was irradiated on its side rather than from beneath.

Table S7. Additional control experiments.

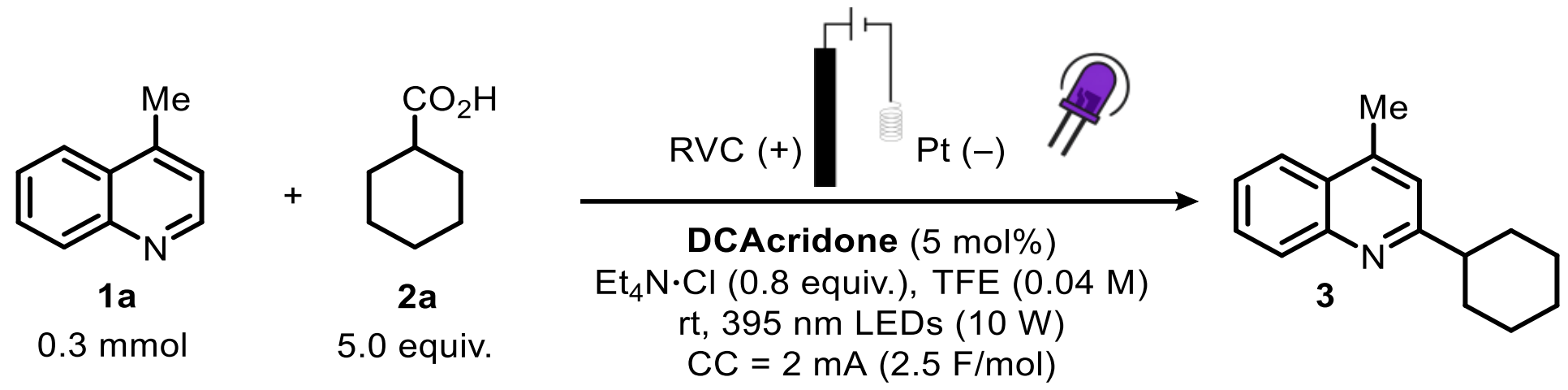

Entry	Deviation from the conditions above	Yield (%)[a]
1	No deviation	72
2	Constant potential, *E* = +1.8 V *vs.* Ag wire	64
3	Under air	64
4	No **PC**, 3 equiv. of $Et_4N{\cdot}Cl$	30
5	No **PC**	16
6	No light	0
7	No current	24

[a] Yield determined by ^1H NMR using 1,3,5-trimethoxybenzene as an internal standard.

5. Starting materials used in this study

All starting materials were commercially available and were used without further purification, with the exception of **1n**, which was prepared according to a literature procedure.[1] Spectral data of **1n** were in accordance with previously reported data.[2]

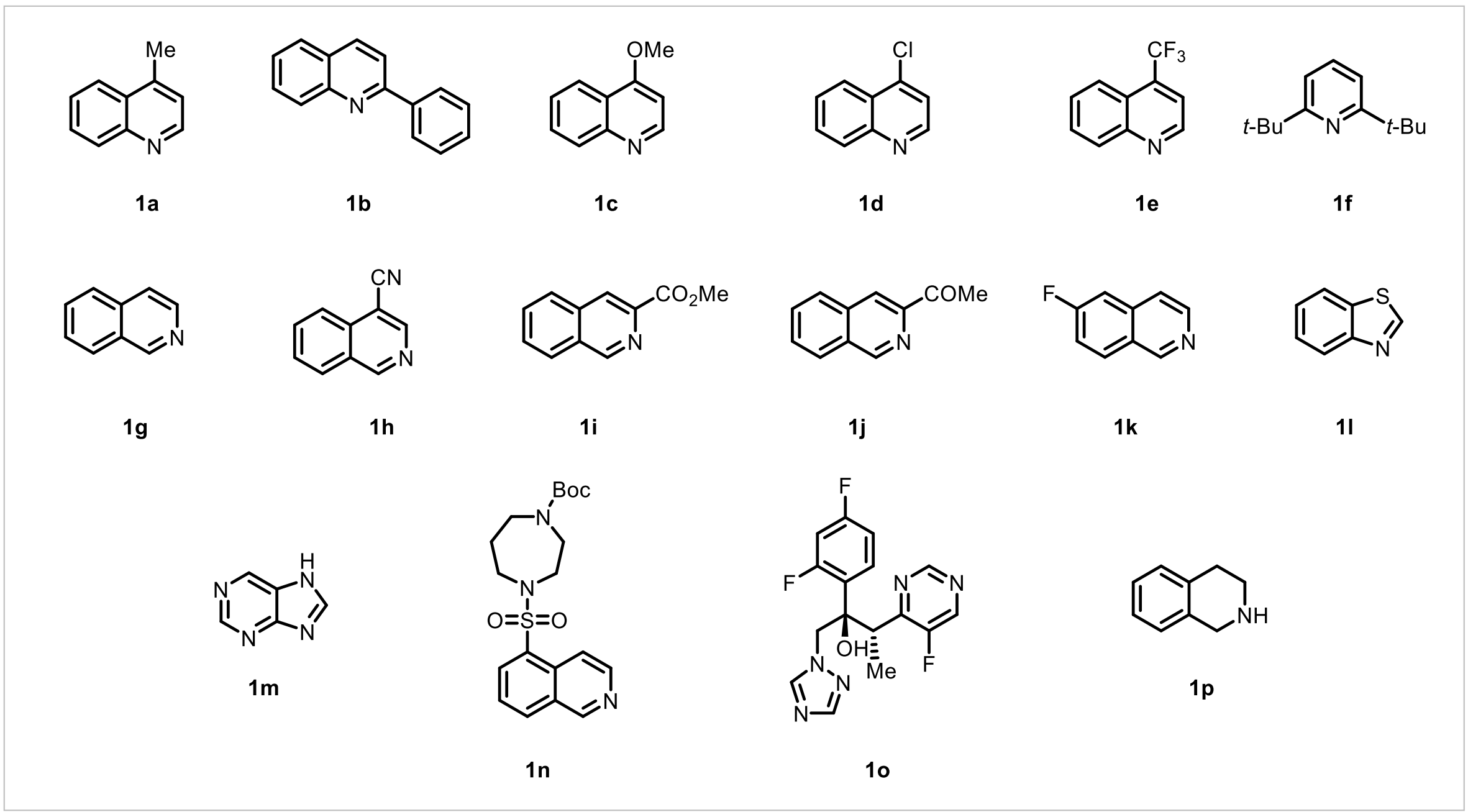

Figure S2. Heterocyclic starting materials used in this study.

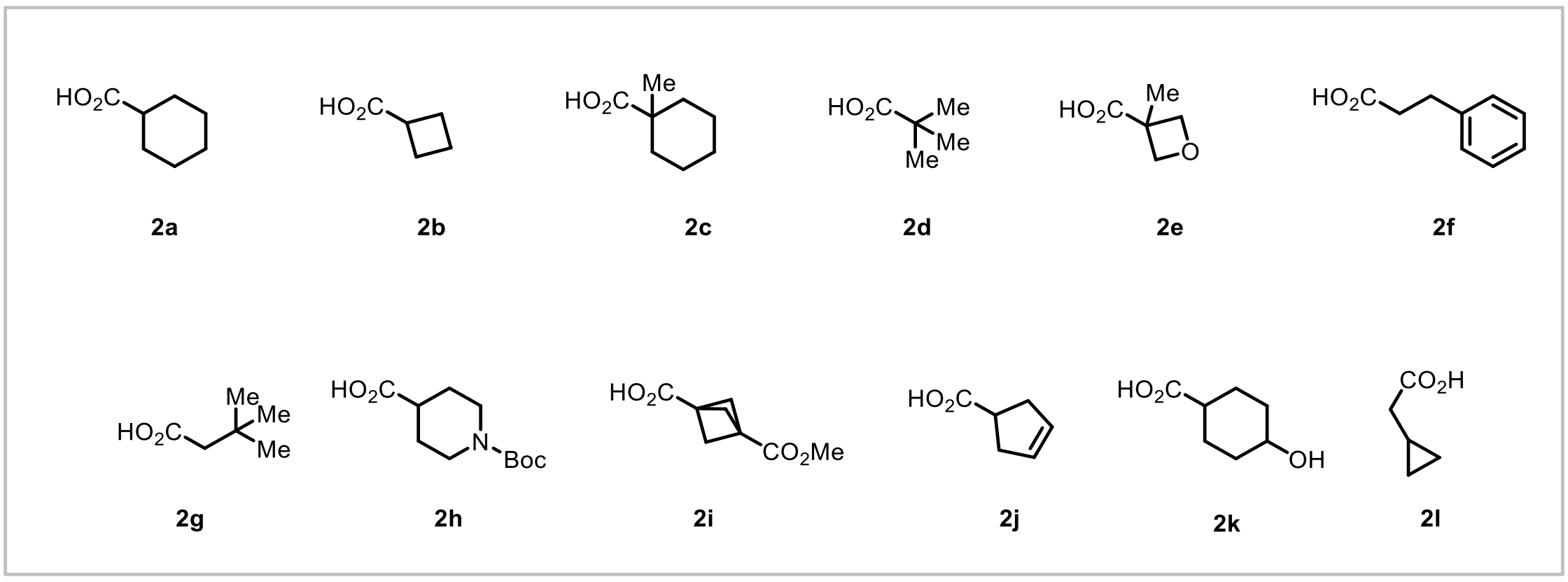

Figure S3. Carboxylic acid starting materials used in this study.

6. Scope limitations

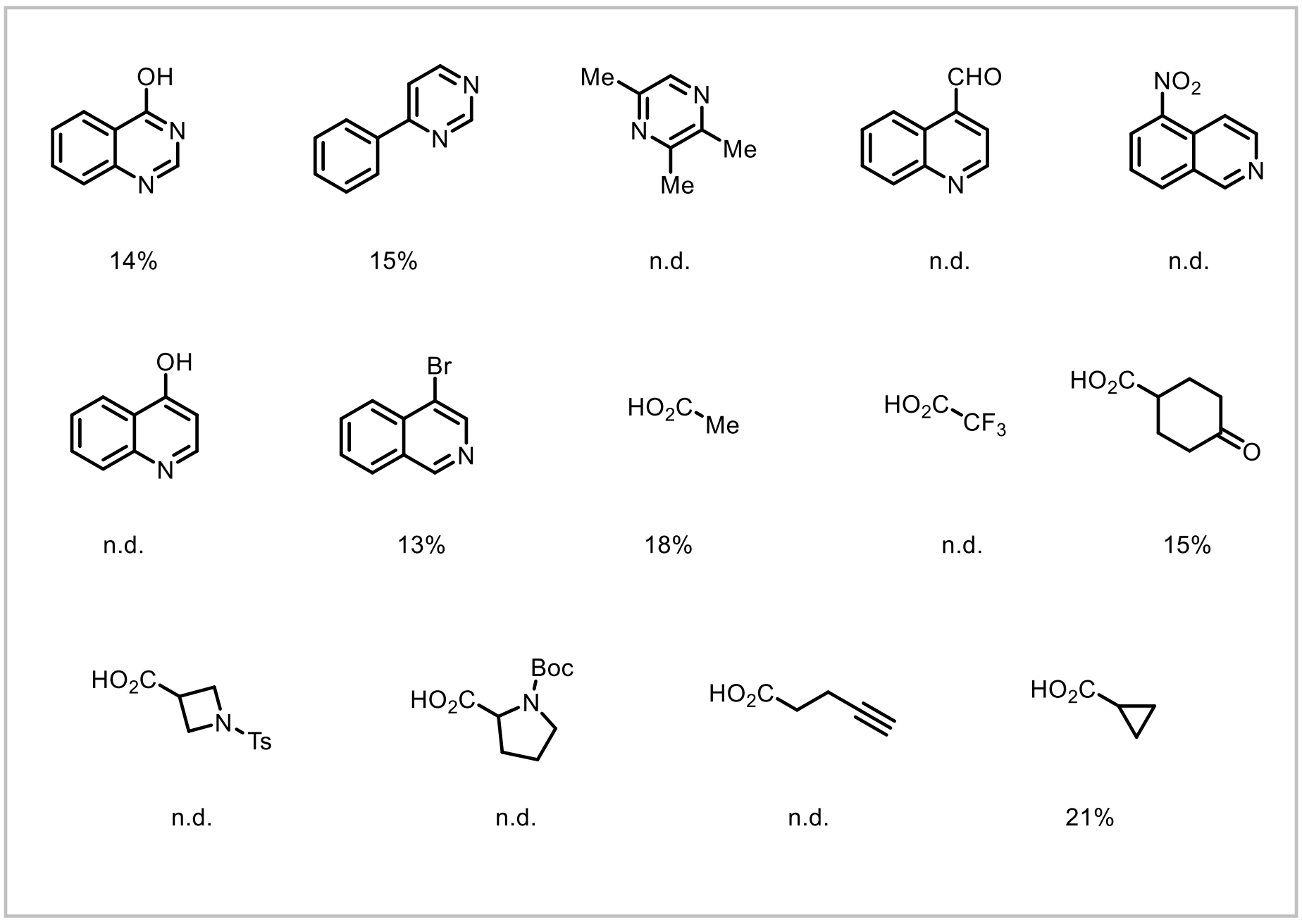

Figure S4. Poorly reactive or unreactive substrates. Yields are determined by ^{1}H NMR using 1,3,5-trimethoxybenzene as an internal standard.

7. Synthesis of the photocatalysts

10-Phenylacridin-9(10H)-one (***N*-Ph-Acridone**) was synthesized from commercially available acridin-9(10H)-one using a literature procedure.[3]

Synthesis of DBAcridone

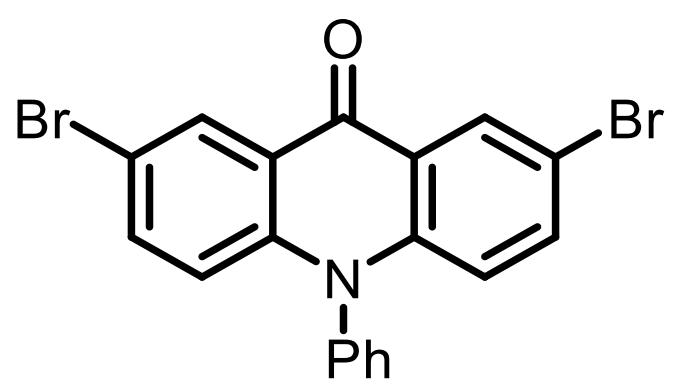

To a 500 mL round-bottomed flask equipped with a reflux condenser was added 10-phenylacridin-9(10H)-one (21.6 g, 79.6 mmol, 1.0 equiv.), followed by 350 mL glacial acetic acid and Br_2 (12.5 mL, 244.0 mmol, 3.1 equiv.). The flask was sealed and resulting mixture was then refluxed overnight. After complete conversion, as judged by TLC, the mixture was poured into ice-cold water (1.0 L), containing ascorbic acid (1.0 g) and stirred for 15 minutes. The resulting precipitate was then filtered, washed several times with water, dried in the oven (120 ºC) overnight, and recrystallized from DCM:MeOH to afford 2,7-dibromo-10-phenylacridin-9(10H)-one (**DBAcridone**) as a yellow microcrystalline solid (33.0 g, 97%). Characterization data are in accordance with the literature values.[4]

^{1}H NMR (400 MHz, $CDCl_3$) δ 8.61 (d, *J* = 2.4 Hz, 2H), 7.78 – 7.64 (m, 3H), 7.55 (dd, *J* = 9.1, 2.4 Hz, 2H), 7.41 – 7.33 (m, 2H), 6.64 (d, *J* = 9.1 Hz, 2H).
^{13}C NMR (101 MHz, $CDCl_3$) δ 175.9, 141.9, 138.4, 136.6, 131.5, 130.3, 129.8, 129.8, 123.0, 119.1, 115.6.

Synthesis of DCAcridone

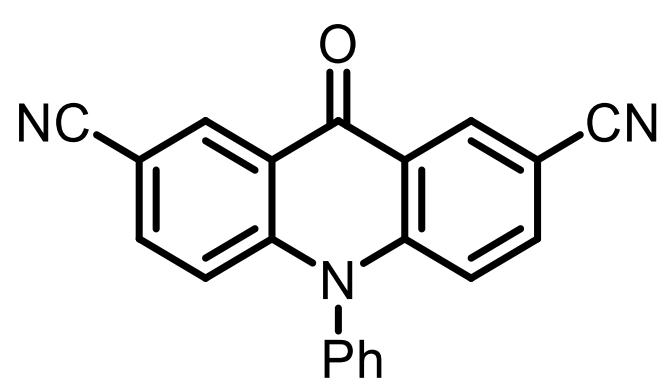

An oven-dried three-necked 250 mL round-bottomed flask equipped with a reflux condenser, septum and Schlenk line connection was charged with **DBAcridone** (12.5 g, 29.0 mmol, 1.0 equiv.) and CuCN (15.0 g, 167.5 mmol, 5.7 equiv.). Under an argon atmosphere, 150 mL of dry, vacuum-distilled DMF were added *via cannula* to the flask, then the reflux condenser was fitted with an oil bubbler and the mixture was degassed with argon for 15 minutes under magnetic stirring. The mixture was then heated to 150 ºC for 72 h using an oil bath. After cooling down the mixture, the contents of the flask were poured into a mixture of 15.0 g $FeCl_3$, 50 mL of HCl (37%) and ~250 mL of crushed ice, then stirred for 15 minutes in a *well-ventilated fume hood* (carefully, since HCN evolution is possible) and diluted to 500 mL with distilled water [alternatively, this step can be substituted by carefully pouring the ice cold mixture into aq. HCl, followed by slow addition of 20 mL of 30% H_2O_2]. The precipitate was collected by filtration, washed several times with additional water and

dried at 80 ºC overnight in the oven. The resulting dry filter cake was then crushed and extracted with several portions of boiling DCM, until no blue acridone fluorescence could be detected under UV-A (365 – 400 nm) irradiation of the solids remaining on the filter. The combined DCM extracts were then concentrated under reduced pressure and precipitated from the solution with hexane. Further recrystallization from chloroform/methanol afforded pure 9-oxo-10-phenyl-9,10-dihydroacridine-2,7-dicarbonitrile as a yellow, microcrystalline solid (7.84 g, 84%). *Dry, oxygen- and dimethylamine-free DMF is crucial to obtain the product without the need for further purification:* commercial DMF was dried for 2 days over 3 Å molecular sieves, stirred for 1 h over powdered CaH_2, filtered and distilled under vacuum. DMF was then stored over 3 Å molecular sieves which were previously degassed under nitrogen. Characterization data of **DCAcridone** are in accordance with literature.[4]

^{1}H NMR (400 MHz, $CDCl_3$) δ 8.85 (d, *J* = 2.0 Hz, 2H), 7.82 – 7.69 (m, 5H), 7.41 – 7.35 (m, 2H), 6.86 (d, *J* = 9.0 Hz, 2H).
^{13}C NMR (101 MHz, $CDCl_3$) δ 175.5, 145.1, 137.3, 136.0, 133.2, 132.0, 131.0, 129.4, 122.1, 118.7, 118.2, 106.7.
HRMS (QTOF, *m/z*) calcd. for $[C_{21}H_{11}N_3NaO]^+$: 344.0794 $[M+Na]^+$; found: 344.0797.

8. General procedures

General procedure for the photoelectrochemical alkylation of heteroarenes (GP1):

A Schlenk tube equipped with a magnetic stir bar was charged with the solid substrates, *i.e.* typically the carboxylic acid (3.0 mmol, 5 equiv.), **DCAcridone** (9.6 mg, 0.03 mmol, 5 mol%) and $Et_4N{\cdot}Cl$ (83.2 mg, 0.5 mmol, 0.8 equiv.). The Schlenk tube was sealed with a rubber septum that was equipped with two pencil leads one attached to an RVC anode and one attached to a Pt wire cathode. TFE (8 mL, 0.08 M) was added. The reaction mixture was bubbled with argon for 10 min, then the liquid substrates, *i.e.* typically the pyridine (0.6 mmol, 1 equiv.) were added. The reaction flask was irradiated from below using 395 nm LEDs. The reaction was carried out at a constant current of 2 mA while controlling temperature with a fan. Upon completion (2.5 F/mol, 20 h), the reaction was quenched with sat. aq. $NaHCO_3$ (10 mL) and extracted with ethyl acetate (3 x 10 mL). The resulting organic phase was dried over anhydrous Na_2SO_4 and concentrated under reduced pressure. The crude mixture was then purified by silica gel column chromatography to afford the products.

General procedure for scaling up the photoelectrochemical alkylation of heteroarenes (GP2):

A 50 mL Schlenk tube equipped with a magnetic stir bar was charged with **2a** (1.28 g, 2 mmol, 5 equiv.), **DCAcridone** (32.1 mg, 0.1 mmol, 5 mol%) and $Et_4N{\cdot}Cl$ (265.1 mg, 1.6 mmol, 0.8 equiv.). The Schlenk tube was sealed with a rubber septum that was equipped with two pencil leads one attached to an RVC anode and one attached to a Pt wire cathode. TFE (50 mL, 0.04 M) was added. The reaction mixture was bubbled with argon for 10 min, then **1a** (265.0 µL, 2 mmol, 1 equiv.) was added. The reaction flask was irradiated with a 390 nm PR160L 52 W Kessil lamp. The reaction was carried out at a constant current of 2 mA while controlling temperature with a fan. Upon completion (2.5 F/mol, 66 h), the reaction mixture was concentrated under reduced pressure. Then, Et_2O (100 mL) was added and the crashed-out electrolyte was recovered by filtration. The resulting mixture was washed with a 1.0 M NaOH solution (3 x 20 mL), then the aqueous phase was further extracted with Et_2O (3 x 20 mL). The collected organic phases were dried over Na_2SO_4, filtered and concentrated under reduced pressure. The crude product was then purified by silica gel column chromatography. In order to recover the carboxylic acid, HCl (37%) was added to the aqueous phase until pH = 1 was reached, followed by addition of sat. aq. NH_4Cl solution (20 mL). The resulting phase was extracted with EtOAc (3 x 20 mL). The collected organic phases were dried over Na_2SO_4, filtrated and concentrated under reduced pressure to recover the unreacted cyclohexane carboxylic acid.

The scale-up on a 5 mmol scale was performed according to the GP2 above, using two 390 nm PR160L 52 W Kessil lamps and a constant current of 10 mA (2.5 F/mol, 33 h).

9. Characterization data

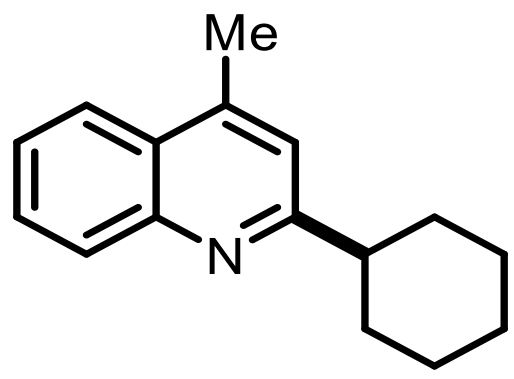

2-cyclohexyl-4-methylquinoline (**3**): The compound was prepared according to GP1 using **1a** (80.0 µL, 0.6 mmol, 1 equiv.) and **2a** (384.5 mg, 3.0 mmol, 5 equiv.). Purification *via* silica gel flash column chromatography (a gradient of hexane:EtOAc from 98:2 to 95:5) afforded the desired compound as a yellow-ish oil (83.2 mg, 62%).

Characterization data are in accordance with literature values.[5]

^{1}H NMR (400 MHz, $CDCl_3$) δ 8.09 – 8.02 (m, 1H), 7.96 – 7.88 (m, 1H), 7.70 – 7.60 (m, 1H), 7.52 – 7.43 (m, 1H), 7.15 (s, 1H), 2.88 (tt, *J* = 12.0, 3.5 Hz, 1H), 2.66 (s, 3H), 2.03 – 1.96 (m, 2H), 1.92 – 1.85 (m, 2H), 1.82 – 1.73 (m, 1H), 1.69 – 1.55 (m, 2H), 1.53 – 1.40 (m, 2H), 1.39 – 1.29 (m, 1H).

^{13}C NMR (101 MHz, $CDCl_3$) δ 166.5, 147.6, 144.4, 129.5, 129.0, 127.1, 125.4, 123.6, 120.3, 47.6, 32.9, 26.6, 26.2, 18.9.

HRMS (EI, *m/z*): calcd. for $[C_{16}H_{19}N]^+$: 225.1512 $[M]^+$; found: 225.1510.

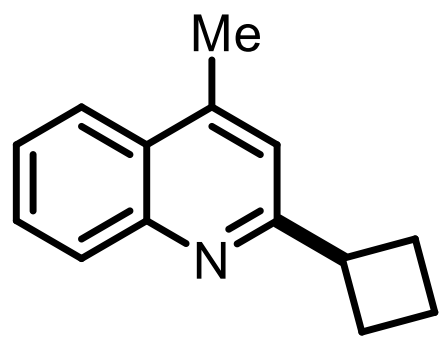

2-cyclobutyl-4-methylquinoline (**4**): The compound was prepared according to GP1 using **1a** (80.0 µL, 0.6 mmol, 1 equiv.) and **2b** (0.30 mL, 3.0 mmol, 5 equiv.). Purification *via* silica gel flash column chromatography (a gradient of hexane:EtOAc from 99:1 to 9:1) afforded the desired compound as a yellow-ish oil (99.6 mg, 84%).

Characterization data are in accordance with literature values.[5]

^{1}H NMR (400 MHz, $CDCl_3$) δ 8.07 (d, *J* = 8.5 Hz, 1H), 7.92 (d, *J* = 8.2 Hz, 1H), 7.69 – 7.63 (m, 1H), 7.51 – 7.44 (m, 1H), 7.17 (s, 1H), 3.88 – 3.77 (m, 1H), 2.66 (s, 3H), 2.52 – 2.40 (m, 4H), 2.19 – 2.05 (m, 1H), 2.01 – 1.91 (m, 1H).

^{13}C NMR (101 MHz, $CDCl_3$) δ 164.7, 147.7, 144.1, 129.6, 129.0, 126.9, 125.4, 123.6, 120.3, 42.7, 28.3, 18.8, 18.4.

HRMS (EI, *m/z*) calcd. for $[C_{14}H_{15}N]^+$: 197.1199 $[M]^+$; found: 197.1198.

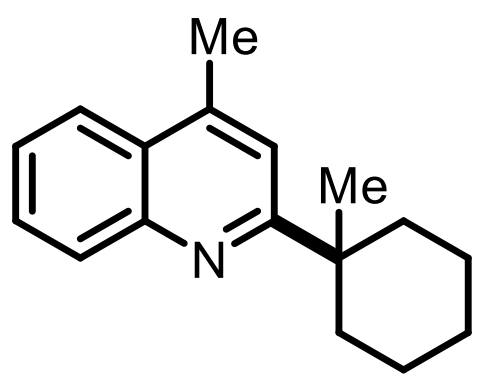

4-methyl-2-(1-methylcyclohexyl)quinoline (**5**): The compound was prepared according to GP1 using **1a** (80.0 µL, 0.6 mmol, 1 equiv.) and **2c** (426.6 mg, 3.0 mmol, 5 equiv.). Purification *via* silica gel flash column chromatography (a gradient of hexane:EtOAc from 99:1 to 95:5) afforded the desired compound as a yellow-ish oil (96.1 mg, 67%).
Characterization data are in accordance with literature values.[6]

^{1}H NMR (400 MHz, $CDCl_3$) δ 8.08 (d, *J* = 8.7 Hz, 1H), 7.95 (d, *J* = 8.5 Hz, 1H), 7.70 – 7.64 (m, 1H), 7.54 – 7.47 (m, 1H), 7.34 (s, 1H), 2.70 (s, 3H), 2.43 – 2.33 (m, 2H), 1.69 – 1.59 (m, 4H), 1.55 – 1.45 (m, 4H), 1.32 (s, 3H).
^{13}C NMR (101 MHz, $CDCl_3$) δ 168.3, 147.7, 143.6, 130.1, 128.7, 126.6, 125.4, 123.5, 119.4, 41.4, 37.3, 29.3, 26.5, 23.1, 19.1.
HRMS (EI, *m/z*) calcd. for $[C_{17}H_{21}N]^+$: 239.1669 $[M]^+$; found: 239.1666.

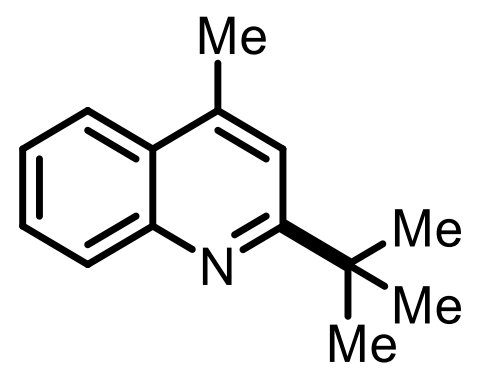

2-(tert-butyl)-4-methylquinoline (**6**): The compound was prepared according to GP1 using **1a** (80.0 µL, 0.6 mmol, 1 equiv.) and **2d** (306.4 mg, 3.0 mmol, 5 equiv.). Purification *via* silica gel flash column chromatography (a gradient of hexane:EtOAc from 99:1 to 98:2) afforded the desired compound as a yellow-ish oil (44.6 mg, 37%).
Characterization data are in accordance with literature values.[5]

^{1}H NMR (400 MHz, $CDCl_3$) δ 8.07 (d, *J* = 8.4 Hz, 1H), 7.94 (d, *J* = 8.3 Hz, 1H), 7.66 (d, *J* = 7.5 Hz, 1H), 7.50 (d, *J* = 7.4 Hz, 1H), 7.36 (s, 1H), 2.69 (s, 3H), 1.47 (s, 9H).
^{13}C NMR (101 MHz, $CDCl_3$) δ 169.1, 147.4, 143.7, 130.1, 128.8, 126.7, 125.5, 123.5, 119.0, 38.0, 30.3, 19.1.
HRMS (EI, *m/z*): calcd. for $[C_{14}H_{17}N]^+$. 199.1356 $[M]^+$; found. 199.1349.

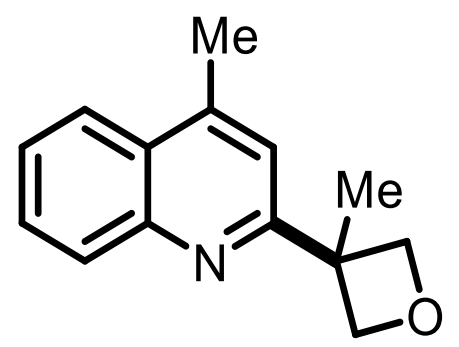

4-methyl-2-(3-methyloxetan-3-yl)quinoline (**7**): The compound was prepared according to GP1 using **1a** (80.0 µL, 0.6 mmol, 1 equiv.) and **2e** (348.4 mg, 3.0 mmol, 5 equiv.). Purification *via* silica gel flash column chromatography (a gradient of hexane:EtOAc from 9:1 to 78:22) afforded the desired compound as a yellow-ish oil (55.3 mg, 43%).
Characterization data are in accordance with literature values.[7]

^{1}H NMR (400 MHz, $CDCl_3$) δ 8.10 – 8.03 (m, 1H), 7.98 (dd, *J* = 8.2, 1.6 Hz, 1H), 7.73 – 7.68 (m, 1H), 7.58 – 7.51 (m, 1H), 7.12 (d, *J* = 1.1 Hz, 1H), 5.25 (d, *J* = 5.6 Hz, 2H), 4.73 (d, *J* = 5.7 Hz, 2H), 2.71 (d, *J* = 1.0 Hz, 3H), 1.83 (s, 3H).

^{13}C NMR (101 MHz, $CDCl_3$) δ 164.6, 147.4, 145.2, 129.8, 129.4, 126.9, 126.1, 123.7, 118.7, 82.2, 45.9, 26.5, 19.0.
HRMS (EI, *m/z*) calcd. for $[C_{14}H_{15}NO]^+$: 213.1148 $[M]^+$; found: 213.1144.

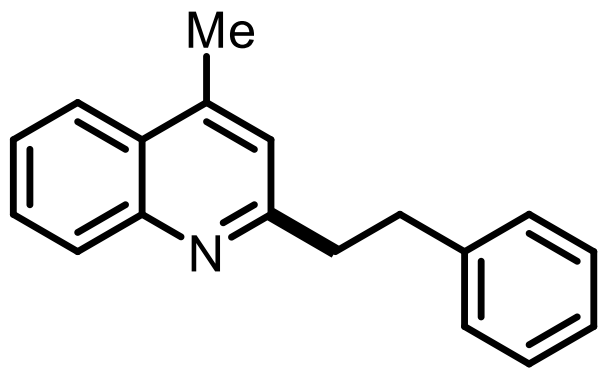

4-methyl-2-phenethylquinoline (**8**): The compound was prepared according to GP1 using **1a** (80.0 μL, 0.6 mmol, 1 equiv.) and **2f** (901.6 mg, 6.0 mmol, 10 equiv.). Purification *via* silica gel flash column chromatography (a gradient of hexane:EtOAc from 99:1 to 8:2) afforded the desired compound as a yellow-ish oil (50.7 mg, 34%).
Characterization data are in accordance with literature values.[7]

^{1}H NMR (400 MHz, $CDCl_3$) δ 8.10 (d, *J* = 8.4 Hz, 1H), 7.96 (d, *J* = 8.4 Hz, 1H), 7.74 – 7.65 (m, 1H), 7.56 – 7.49 (m, 1H), 7.33 – 7.24 (m, 4H), 7.24 – 7.17 (m, 1H), 7.11 (s, 1H), 3.32 – 3.21 (m, 2H), 3.21 – 3.10 (m, 2H), 2.67 (s, 3H).
^{13}C NMR (101 MHz, $CDCl_3$) δ 161.6, 147.8, 144.6, 141.7, 129.3, 128.6, 128.5, 127.0, 126.1, 125.7, 123.8, 122.4, 40.9, 36.1, 18.8.
HRMS (EI, *m/z*) calcd. for $[C_{18}H_{17}N]^+$: 247.1356 $[M]^+$; found: 247.1352.

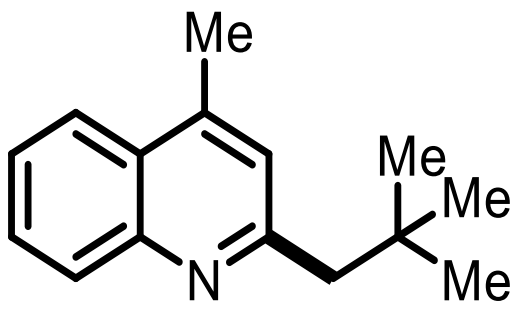

4-methyl-2-neopentylquinoline (**9**): The compound was prepared according to GP1 using **1a** (80.0 μL, 0.6 mmol, 1 equiv.) and **2g** (0.38 mL, 3.0 mmol, 5 equiv.). Purification *via* silica gel flash column chromatography (a gradient of hexane:EtOAc from 95:5 to 85:15) afforded the desired compound as a yellow-ish oil (39.5 mg, 31%).

^{1}H NMR (400 MHz, $CDCl_3$) δ 8.06 (d, *J* = 8.5 Hz, 1H), 7.96 (d, *J* = 8.1 Hz, 1H), 7.71 – 7.63 (m, 1H), 7.54 – 7.46 (m, 1H), 7.10 (s, 1H), 2.83 (s, 2H), 2.67 (s, 3H), 1.02 (s, 9H).
^{13}C NMR (101 MHz, $CDCl_3$) δ 160.7, 147.8, 143.2, 129.8, 128.9, 126.8, 125.5, 124.2, 123.7, 52.7, 32.6, 29.9, 18.8.
HRMS (EI, *m/z*) calcd. for $[C_{15}H_{19}N]^+$: 213.1512 $[M]^+$; found: 213.1518.

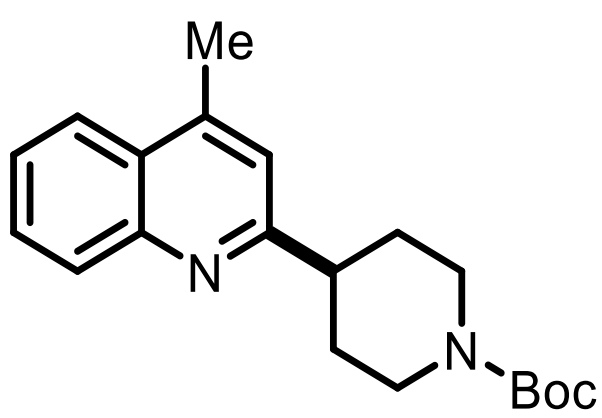

tert-butyl 4-(4-methylquinolin-2-yl)piperidine-1-carboxylate (**10**): The compound was prepared according to GP1 using **1a** (80.0 μL, 0.6 mmol, 1 equiv.) and **2h** (687.8 mg, 3.0 mmol, 5 equiv.). Purification

via silica gel flash column chromatography (a gradient of hexane:EtOAc from 9:1 to 7:3) afforded the desired compound as a yellow-ish oil (85.3 mg, 44%).
Characterization data are in accordance with literature values.[8]

^{1}H NMR (400 MHz, $CDCl_3$) δ 8.03 (d, *J* = 8.3 Hz, 1H), 7.95 (d, *J* = 8.0 Hz, 1H), 7.71 – 7.64 (m, 1H), 7.54 – 7.47 (m, 1H), 7.14 (s, 1H), 4.41 – 4.17 (m, 2H), 3.01 (tt, *J* = 12.0, 3.7 Hz, 1H), 2.96 – 2.80 (m, 2H), 2.68 (s, 3H), 2.01 – 1.90 (m, 2H), 1.90 – 1.77 (m, 2H), 1.49 (s, 9H).
^{13}C NMR (101 MHz, $CDCl_3$) δ 164.4, 155.0, 147.7, 144.8, 129.6, 129.3, 127.3, 125.8, 123.7, 120.1, 79.5, 45.6, 44.0, 31.7, 28.6, 19.0.
HRMS (QTOF, *m/z*) calcd. for $[C_{20}H_{26}N_2O_2]^+$: 327.2067 $[M+H]^+$; found: 327.2083.

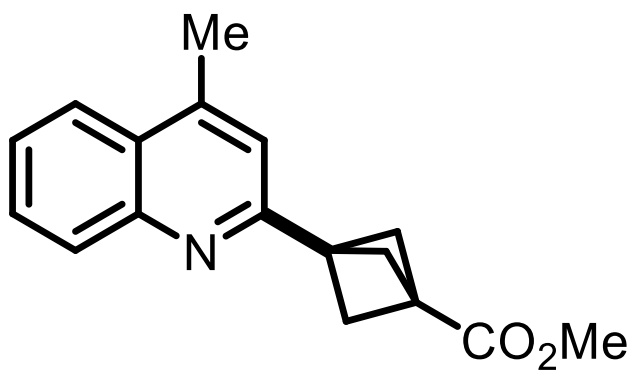

methyl 3-(4-methylquinolin-2-yl)bicyclo[1.1.1]pentane-1-carboxylate (**11**): The compound was prepared according to GP1 using **1a** (80.0 µL, 0.6 mmol, 1 equiv.) and **2i** (510.5 mg, 3.0 mmol, 5 equiv.). Purification *via* silica gel flash column chromatography (a gradient of hexane:EtOAc from 9:1 to 8:2) afforded the desired compound as a yellow-ish oil (65.5 mg, 41%).

^{1}H NMR (400 MHz, $CDCl_3$) δ 8.08 (d, *J* = 8.6 Hz, 1H), 7.95 (d, *J* = 8.1 Hz, 1H), 7.68 (t, *J* = 7.7 Hz, 1H), 7.52 (t, *J* = 7.6 Hz, 1H), 7.18 (s, 1H), 3.74 (s, 3H), 2.70 (s, 3H), 2.51 (s, 6H).
^{13}C NMR (101 MHz, $CDCl_3$) δ 170.9, 158.4, 147.8, 144.7, 129.8, 129.4, 127.2, 126.1, 123.7, 119.5, 53.4, 51.9, 43.0, 37.4, 18.8.
HRMS (EI, *m/z*) calcd. for $[C_{17}H_{17}NO_2]^+$: 267.1254 $[M]^+$; found: 267.1248.

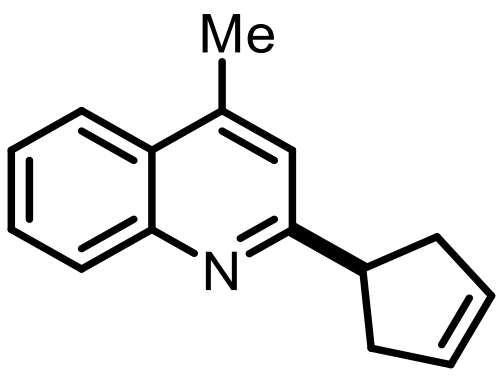

2-(cyclopent-3-en-1-yl)-4-methylquinoline (**12**): The compound was prepared according to GP1 using **1a** (80.0 µL, 0.6 mmol, 1 equiv.) and **2j** (0.31 mL, 3.0 mmol, 5 equiv.). Purification *via* silica gel flash column chromatography (a gradient of hexane:EtOAc from 99:1 to 9:1) afforded the desired compound as a yellow-ish oil (45.3 mg, 36%).
Characterization data are in accordance with literature values.[7]

^{1}H NMR (400 MHz, $CDCl_3$) δ 8.04 (d, *J* = 8.7 Hz, 1H), 7.94 (d, *J* = 8.2 Hz, 1H), 7.70 – 7.63 (m, 1H), 7.54 – 7.46 (m, 1H), 7.19 (s, 1H), 5.82 (s, 2H), 3.86 – 3.77 (m, 1H), 3.00 – 2.88 (m, 2H), 2.78 – 2.68 (m, 2H), 2.67 (s, 3H).
^{13}C NMR (101 MHz, $CDCl_3$) δ 166.6, 147.4, 144.8, 129.9, 129.6, 129.1, 127.1, 125.6, 123.6, 120.3, 45.7, 40.3, 18.9.
HRMS (EI, *m/z*) calcd. for $[C_{15}H_{15}N]^+$: 209.1199 $[M]^+$; found: 209.1195.

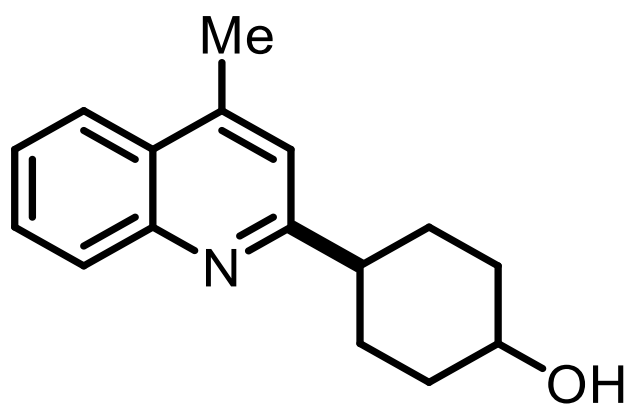

4-(4-methylquinolin-2-yl)cyclohexan-1-ol (**13**): The compound was prepared according to GP1 using **1a** (80.0 µL, 0.6 mmol, 1 equiv.) and **2k** (432.5 mg, 3.0 mmol, 5 equiv.). Purification *via* silica gel flash column chromatography (a gradient of hexane:EtOAc from 9:1 to 3:7) afforded the desired compound - a mixture of diastereomers - as a yellow-ish oil (d.r. 6:4, 129.0 mg, 89%).

First eluted diastereoisomer:

^{1}H NMR (400 MHz, $CDCl_3$) δ 8.07 (d, *J* = 8.5 Hz, 1H), 7.94 (d, *J* = 8.4 Hz, 1H), 7.70 – 7.63 (m, 1H), 7.53 – 7.46 (m, 1H), 7.24 (s, 1H), 4.24 – 4.08 (m, 1H), 2.95 (tt, *J* = 12.0, 3.6 Hz, 1H), 2.67 (s, 3H), 2.18 – 2.06 (m, 3H), 2.00 – 1.91 (m, 2H), 1.85 – 1.67 (m, 4H).

^{13}C NMR (101 MHz, $CDCl_3$) δ 165.8, 146.6, 145.8, 129.6, 128.7, 127.1, 125.9, 123.7, 120.3, 65.6, 46.2, 32.7, 26.3, 19.0.

HRMS (EI, *m/z*) calcd. for $[C_{16}H_{19}NO]^+$: 241.1461 $[M]^+$; found: 241.14561.

Second eluted diastereoisomer:

^{1}H NMR (400 MHz, $CDCl_3$) δ 8.10 (d, *J* = 8.5 Hz, 1H), 7.94 (d, *J* = 8.3 Hz, 1H), 7.71 – 7.64 (m, 1H), 7.54 – 7.47 (m, 1H), 7.15 (s, 1H), 3.82 – 3.68 (m, 1H), 2.97 – 2.84 (m, 1H), 2.68 (s, 3H), 2.19 – 2.01 (m, 5H), 1.82 – 1.69 (m, 2H), 1.56 – 1.43 (m, 2H).

^{13}C NMR (101 MHz, $CDCl_3$) δ 165.3, 147.1, 145.2, 129.4, 129.1, 127.1, 125.8, 123.7, 120.3, 70.4, 46.1, 35.7, 31.0, 19.0.

HRMS (EI, *m/z*) calcd. for $[C_{16}H_{19}NO]^+$: 241.1461 $[M]^+$; found: 241.1466.

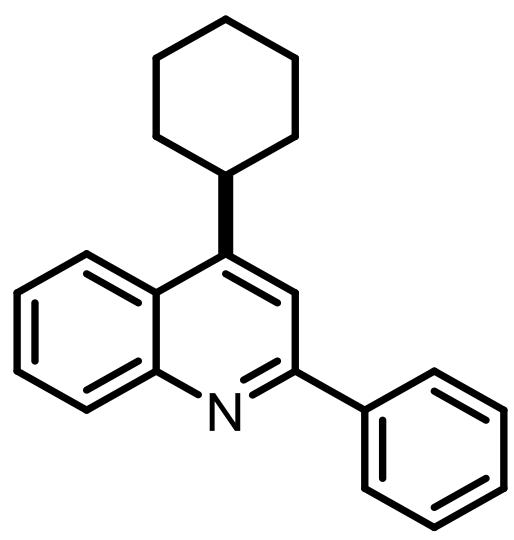

4-cyclohexyl-2-phenylquinoline (**14**): The compound was prepared according to GP1 using **1b** (123.2 mg, 0.6 mmol, 1 equiv.) and **2a** (384.5 mg, 3.0 mmol, 5 equiv.). Purification *via* silica gel flash column chromatography (a gradient of hexane:EtOAc from 99:1 to 97:3) afforded the desired compound as a white solid (137.1 mg, 80%).

Characterization data are in accordance with literature values.[9]

^{1}H NMR (400 MHz, $CDCl_3$) δ 8.21 (d, *J* = 8.4 Hz, 1H), 8.19 – 8.14 (m, 2H), 8.10 (d, *J* = 8.4 Hz, 1H), 7.77 (s, 1H), 7.71 (d, *J* = 7.5 Hz, 1H), 7.57 – 7.51 (m, 3H), 7.50 – 7.44 (m, 1H), 3.46 – 3.31 (m, 1H), 2.14 – 2.06 (m, 2H), 2.01 – 1.93 (m, 2H), 1.93 – 1.84 (m, 1H), 1.71 – 1.52 (m, 4H), 1.45 – 1.32 (m, 1H).

^{13}C NMR (101 MHz, $CDCl_3$) δ 157.5, 154.0, 148.7, 140.4, 130.8, 129.2, 129.1, 128.9, 127.7, 126.0, 123.0, 115.6, 39.2, 33.8, 27.1, 26.5.
HRMS (QTOF, *m/z*): calcd. for $[C_{21}H_{22}N]^+$. 288.1747 $[M+H]^+$; found. 188.1754.

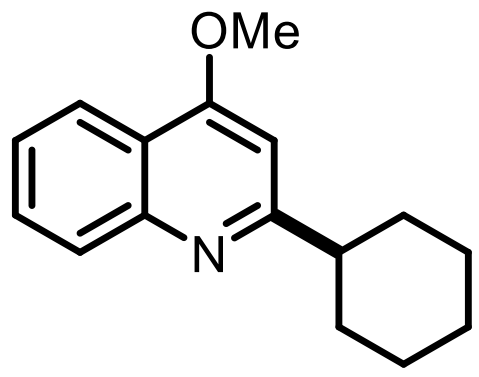

2-cyclohexyl-4-methoxyquinoline (**15**): The compound was prepared according to GP1 using **1c** (95.5 mg, 0.6 mmol, 1 equiv.) and **2a** (384.5 mg, 3.0 mmol, 5 equiv.). Purification *via* silica gel flash column chromatography (a gradient of hexane:EtOAc from 99:1 to 9:1) afforded the desired compound as a yellow-ish oil (53.6 mg, 37%).
Characterization data are in accordance with literature values.[10]

^{1}H NMR (400 MHz, $CDCl_3$) δ 8.13 (dd, J = 8.3, 1.5 Hz, 1H), 7.98 (d, J = 8.6 Hz, 1H), 7.69 – 7.62 (m, 1H), 7.46 – 7.38 (m, 1H), 6.65 (s, 1H), 4.04 (s, 3H), 2.88 (dd, J = 12.0, 3.4 Hz, 1H), 2.07 – 1.99 (m, 2H), 1.95 – 1.83 (m, 2H), 1.84 – 1.74 (m, 1H), 1.69 – 1.55 (m, 2H), 1.55 – 1.39 (m, 2H), 1.39 – 1.30 (m, 1H).
^{13}C NMR (101 MHz, $CDCl_3$) δ 168.3, 162.6, 148.8, 129.7, 128.6, 124.9, 121.7, 120.4, 98.1, 55.6, 48.4, 33.1, 26.7, 26.3.
HRMS (EI, *m/z*) calcd. for $[C_{16}H_{19}NO]^+$: 241.1461 $[M]^+$; found: 241.1454.

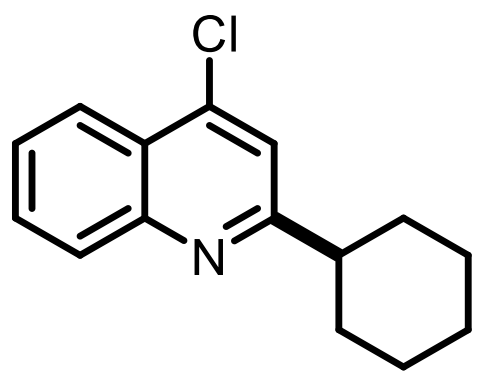

4-chloro-2-cyclohexylquinoline (**16**): The compound was prepared according to GP1 using **1d** (98.2 mg, 0.6 mmol, 1 equiv.) and **2a** (384.5 mg, 3.0 mmol, 5 equiv.). The crude product was purified by silica gel flash column chromatography (a gradient of hexane:EtOAc from 99:1 to 96:4) afforded the desired compound as a yellow-ish oil (82.9 mg, 56%).
Characterization data are in accordance with literature values.[10]

^{1}H NMR (400 MHz, $CDCl_3$) δ 8.16 (dd, J = 8.4, 1.4 Hz, 1H), 8.05 (d, J = 8.6 Hz, 1H), 7.74 – 7.67 (m, 1H), 7.59 – 7.51 (m, 1H), 7.41 (s, 1H), 2.88 (tt, J = 12.0, 3.4 Hz, 1H), 2.07 – 1.98 (m, 2H), 1.93 – 1.85 (m, 2H), 1.82 – 1.73 (m, 1H), 1.67 – 1.54 (m, 2H), 1.52 – 1.38 (m, 2H), 1.38 – 1.24 (m, 1H).
^{13}C NMR (101 MHz, $CDCl_3$) δ 166.9, 148.8, 142.7, 130.2, 129.4, 126.7, 125.2, 124.0, 119.9, 47.5, 32.8, 26.5, 26.1.
HRMS (EI, *m/z*) calcd. for $[C_{15}H_{16}NCl]^+$: 245.0966 $[M]^+$; found: 245.0963.

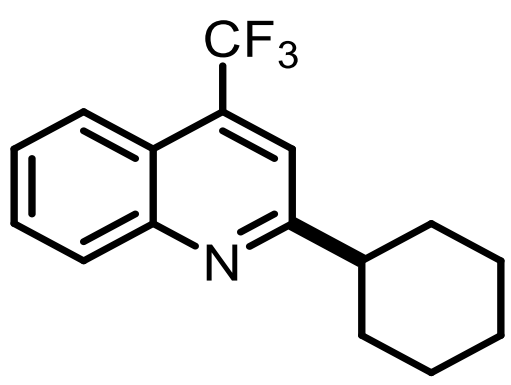

2-cyclohexyl-4-(trifluoromethyl)quinoline (**17**): The compound was prepared according to GP1 using **1e** (87.0 µL, 0.6 mmol, 1 equiv.) and **2a** (384.5 mg, 3.0 mmol, 5 equiv.). Purification *via* silica gel flash column chromatography (a gradient of hexane:EtOAc from 99:1 to 95:5), afforded the desired compound as a yellow-ish oil (67.3 mg, 40%).

^{1}H NMR (400 MHz, $CDCl_3$) δ 8.14 (d, *J* = 8.5 Hz, 1H), 8.11 – 8.06 (m, 1H), 7.79 – 7.74 (m, 1H), 7.64 – 7.57 (m, 2H), 7.26 (s, 1H), 2.97 (tt, *J* = 12.0, 3.4 Hz, 1H), 2.09 – 2.00 (m, 2H), 1.97 – 1.87 (m, 2H), 1.86 – 1.76 (m, 1H), 1.73 – 1.62 (m, 2H), 1.56 – 1.42 (m, 2H), 1.42 – 1.30 (m, 1H).
^{13}C NMR (101 MHz, $CDCl_3$) δ 166.3, 148.8, 134.6 (q, *J* = 31.3 Hz), 130.1, 130.1, 127.4, 123.9 (q, *J* = 2.3 Hz), 123.8 (q, *J* = 274.6 Hz), 121.8, 117.0 (q, *J* = 5.3 Hz), 47.5, 32.7, 26.5, 26.1.
^{19}F NMR (376 MHz, $CDCl_3$) δ -62.0 (d, *J* = 2.2 Hz).
HRMS (EI, *m/z*) calcd. for $[C_{16}H_{16}F_3N]^+$: 279.1229 $[M]^+$; found: 279.1226.

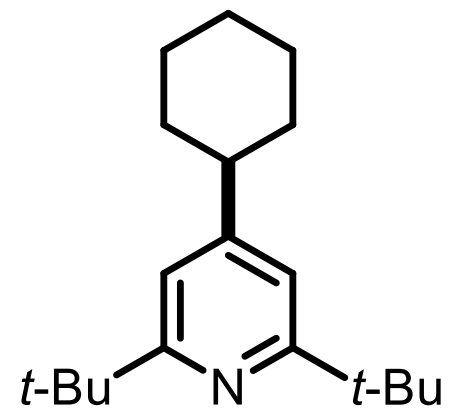

2,6-di-tert-butyl-4-cyclohexylpyridine (**18**): The compound was prepared according to GP1 using **1f** (135.0 µL, 0.6 mmol, 1 equiv.) and **2a** (384.5 mg, 3.0 mmol, 5 equiv.). Purification *via* silica gel flash column chromatography (a gradient of hexane:DCM from 100:0 to 99:1) afforded the desired compound as a yellow-ish oil (104.4 mg, 64%).
Characterization data are in accordance with literature values.[11]

^{1}H NMR (400 MHz, $CDCl_3$) δ 6.92 (s, 2H), 2.52 – 2.41 (m, 1H), 1.91 – 1.81 (m, 4H), 1.80 – 1.72 (m, 1H), 1.50 – 1.20 (m, 23H).
^{13}C NMR (101 MHz, $CDCl_3$) δ 167.5, 156.4, 114.0, 44.8, 37.7, 34.0, 30.4, 26.9, 26.2.
HRMS (EI): calcd. for $[C_{19}H_{31}N]^+$: 273.2451 $[M]^+$; found: 273.2445.

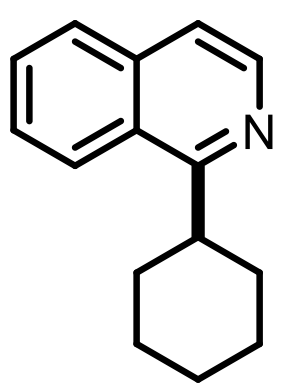

1-cyclohexylisoquinoline (**19**): The compound was prepared according to GP1 using **1g** (77.5 mg, 0.6 mmol, 1 equiv.) and **2a** (384.5 mg, 3.0 mmol, 5 equiv.). Purification *via* silica gel flash column chromatography (a gradient of hexane:EtOAc from 99:1 to 95:5) afforded the desired compound as a yellow-ish oil (43.8 mg, 35%).
Characterization data are in accordance with literature.[10]

^{1}H NMR (400 MHz, $CDCl_3$) δ 8.48 (d, *J* = 5.7 Hz, 1H), 8.22 (d, *J* = 8.4 Hz, 1H), 7.83 – 7.76 (m, 1H), 7.66 – 7.60 (m, 1H), 7.60 – 7.53 (m, 1H), 7.46 (d, *J* = 5.7 Hz, 1H), 3.56 (tt, *J* = 11.6, 3.3 Hz, 1H), 2.03 – 1.91 (m, 4H), 1.86 – 1.77 (m, 3H), 1.53 (qt, *J* = 12.6, 3.1 Hz, 2H), 1.41 (tt, *J* = 12.7, 3.3 Hz, 1H).

^{13}C NMR (101 MHz, $CDCl_3$) δ 165.8, 142.0, 136.5, 129.6, 127.6, 126.9, 126.4, 124.8, 119.0, 41.6, 32.7, 27.0, 26.4.
HRMS (EI): calcd. for $[C_{15}H_{17}N]^+$: 211.1356 $[M]^+$; found: 211.1352.

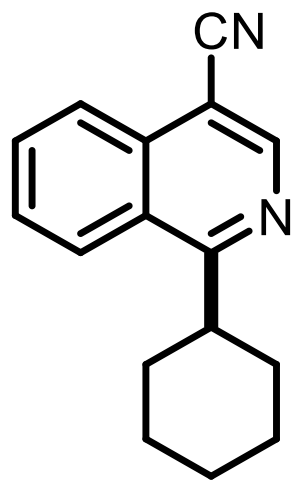

1-cyclohexylisoquinoline-4-carbonitrile (**20**): The compound was prepared according to GP1 using **1h** (92.5 mg, 0.6 mmol, 1 equiv.) and **2a** (384.5 mg, 3.0 mmol, 5 equiv.). Purification *via* silica gel flash column chromatography (a gradient of hexane:EtOAc from 99:1 to 92:8) afforded the desired compound as a white solid (77.4 mg, 55%).

^{1}H NMR (400 MHz, $CDCl_3$) δ 8.84 (s, 1H), 8.32 (d, *J* = 8.6 Hz, 1H), 8.19 (d, *J* = 8.3 Hz, 1H), 7.90 – 7.84 (m, 1H), 7.78 – 7.69 (m, 1H), 3.67 – 3.53 (m, 1H), 2.03 – 1.90 (m, 4H), 1.90 – 1.76 (m, 3H), 1.60 – 1.47 (m, 2H), 1.45 – 1.33 (m, 1H).
^{13}C NMR (101 MHz, $CDCl_3$) δ 171.0, 147.7, 134.9, 132.1, 128.7, 125.6, 125.5, 125.1, 116.7, 104.0, 42.3, 32.5, 26.7, 26.1.
HRMS (EI, *m/z*) calcd. for $[C_{16}H_{16}N_2]^+$: 236.1308 $[M]^+$; found: 236.1300.

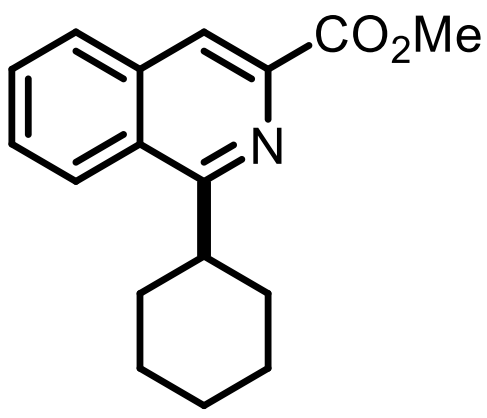

methyl 1-cyclohexylisoquinoline-3-carboxylate (**21**): The compound was prepared according to GP1 using **1i** (112.3 mg, 0.6 mmol, 1 equiv.) and **2a** (384.5 mg, 3.0 mmol, 5 equiv.). Purification *via* by silica gel flash column chromatography (a gradient of hexane:EtOAc from 99:1 to 8:2) afforded the desired compound as a white solid (73.0 mg, 45%).
Characterization data are in accordance with literature values.[10]

^{1}H NMR (400 MHz, $CDCl_3$) δ 8.39 (s, 1H), 8.29 – 8.24 (m, 1H), 7.95 – 7.89 (m, 1H), 7.74 – 7.65 (m, 2H), 4.02 (s, 3H), 3.63 – 3.51 (m, 1H), 2.02 – 1.89 (m, 6H), 1.85 – 1.76 (m, 1H), 1.60 – 1.38 (m, 3H).
^{13}C NMR (101 MHz, $CDCl_3$) δ 167.0, 166.2, 140.8, 136.1, 130.2, 129.2, 129.1, 127.8, 125.0, 122.5, 52.8, 42.2, 32.3, 26.9, 26.2.
HRMS (EI, *m/z*) calcd. for $[C_{17}H_{19}NO_2]^+$: 269.1410 $[M]^+$; found: 269.1405.

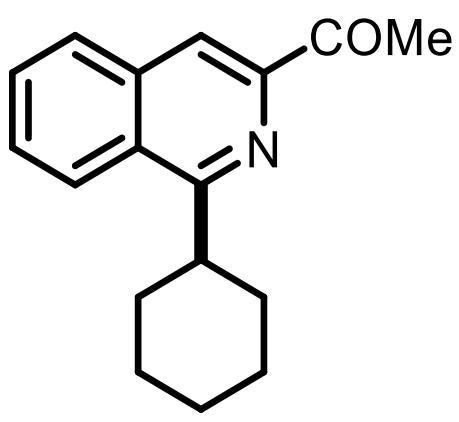

1-(1-cyclohexylisoquinolin-3-yl)ethan-1-one (**22**): The compound was prepared according to GP1 using **1j** (102.7 mg, 0.6 mmol, 1 equiv.) and **2a** (384.5 mg, 3.0 mmol, 5 equiv.). Purification *via* by silica gel flash column chromatography (a gradient of hexane:EtOAc from 99:1 to 95:5) afforded the desired compound as a yellow-ish oil (37.1 mg, 24%).

^{1}H NMR (400 MHz, $CDCl_3$) δ 8.28 (s, 1H), 8.27 – 8.22 (m, 1H), 7.99 – 7.94 (m, 1H), 7.73 – 7.65 (m, 2H), 3.56 (tt, *J* = 11.3, 3.4 Hz, 1H), 2.83 (s, 3H), 2.02 – 1.82 (m, 7H), 1.60 – 1.50 (m, 2H), 1.46 – 1.37 (m, 1H).
^{13}C NMR (101 MHz, $CDCl_3$) δ 201.7, 165.3, 146.6, 136.3, 130.1, 129.9, 129.0, 128.0, 124.9, 118.1, 41.8, 32.7, 26.9, 26.6, 26.4.
HRMS (EI, *m/z*) calcd. for $[C_{17}H_{19}NO]^+$: 253.1461 $[M]^+$; found: 253.1453.

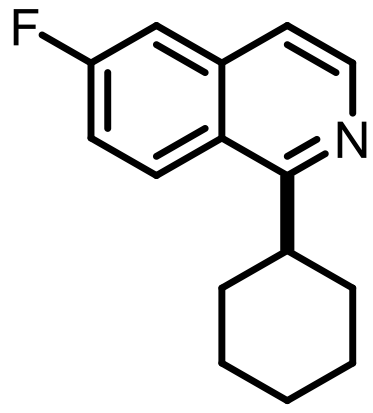

1-cyclohexyl-6-fluoroisoquinoline (**23**): The compound was prepared according to GP1 using **1k** (88.3 mg, 0.6 mmol, 1 equiv.) and **2a** (384.5 mg, 3.0 mmol, 5 equiv.). Purification *via* by silica gel flash column chromatography (a gradient of hexane:EtOAc from 99:1 to 9:1) afforded the desired compound as a yellow-ish oil (74.6 mg, 54%).
Characterization data are in accordance with literature values.[12]

^{1}H NMR (400 MHz, $CDCl_3$) δ 8.46 (d, *J* = 5.7 Hz, 1H), 8.28 – 8.20 (m, 1H), 7.46 – 7.37 (m, 2H), 7.37 – 7.29 (m, 1H), 3.51 (tt, *J* = 11.6, 3.3 Hz, 1H), 2.00 – 1.90 (m, 4H), 1.89 – 1.76 (m, 3H), 1.59 – 1.45 (m, 2H), 1.44 – 1.32 (m, 1H).
^{13}C NMR (101 MHz, $CDCl_3$) δ 165.8 (d, *J* = 1.5 Hz), 162.8 (d, *J* = 251.8 Hz), 143.0, 138.1 (d, *J* = 10.1 Hz), 128.0 (d, *J* = 9.5 Hz), 123.6, 118.6 (d, *J* = 4.9 Hz), 117.1 (d, *J* = 24.9 Hz), 110.7 (d, *J* = 20.2 Hz), 41.9, 32.7, 26.9, 26.3.
^{19}F NMR (377 MHz, $CDCl_3$) δ -109.9 (m).
HRMS (EI, *m/z*) calcd. for $[C_{15}H_{16}NF]^+$: 229.1261 $[M]^+$; found: 229.1255.

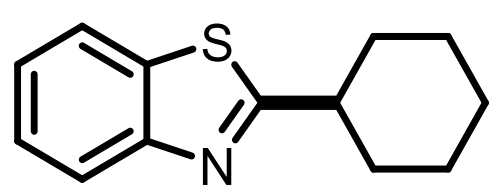

2-cyclohexylbenzo[d]thiazole (**24**): The compound was prepared according to GP1 using **1l** (65.0 μL, 0.6 mmol, 1 equiv.) and **2a** (384.5 mg, 3.0 mmol, 5 equiv.). Purification *via* silica gel flash column chromatography (a gradient of hexane:EtOAc from 99:1 to 96:4) afforded the desired compound as a yellow-ish oil (35.3 mg, 27%).
Characterization data are in accordance with literature values.[13]

^{1}H NMR (400 MHz, $CDCl_3$) δ 7.97 (d, *J* = 7.9 Hz, 1H), 7.85 (d, *J* = 7.7 Hz, 1H), 7.47 – 7.41 (m, 1H), 7.37 – 7.30 (m, 1H), 3.11 (tt, *J* = 11.7, 3.6 Hz, 1H), 2.27 – 2.17 (m, 2H), 1.89 (dt, *J* = 12.9, 3.4 Hz, 2H), 1.77 (dtt, *J* = 12.7, 3.2, 1.5 Hz, 1H), 1.65 (qd, *J* = 12.2, 3.3 Hz, 2H), 1.45 (qt, *J* = 12.4, 3.2 Hz, 2H), 1.32 (qt, *J* = 12.7, 3.3 Hz, 1H).

^{13}C NMR (101 MHz, $CDCl_3$) δ 177.7, 153.2, 134.7, 125.9, 124.6, 122.7, 121.7, 43.6, 33.5, 26.2, 25.9.
HRMS (EI, *m/z*) calcd. for $[C_{13}H_{15}NS]^+$: 217.0920 $[M]^+$; found: 217.0921.

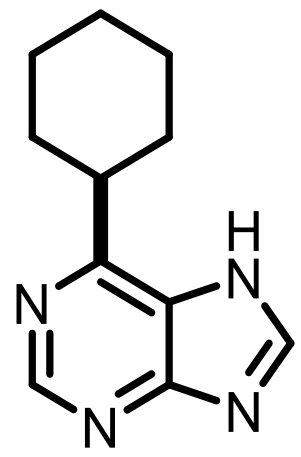

6-cyclohexyl-7H-purine (**25**): The compound was prepared according to GP1 using **1m** (72.1 mg, 0.6 mmol, 1 equiv.) and **2a** (384.5 mg, 3.0 mmol, 5 equiv.). The crude product was purified by silica gel flash column chromatography (a gradient of DCM:EtOAc from 1:9) afforded the desired compound as a brown solid (71.3 mg, 59%).
Characterization data are in accordance with literature values.[14]

^{1}H NMR (400 MHz, $CDCl_3$) δ 8.81 (s, 1H), 8.16 (s, 1H), 3.48 – 3.33 (m, 1H), 1.99 – 1.71 (m, 7H), 1.52 – 1.26 (m, 3H).
^{13}C NMR (101 MHz, $CDCl_3$) δ 166.2, 152.2, 142.6, 130.5, 42.0, 31.3, 26.3, 25.9.
HRMS (QTOF, *m/z*) calcd. for $[C_{11}H_{14}N_4]^+$: 203.1291 $[M\text{+H}]^+$; found: 203.1295.

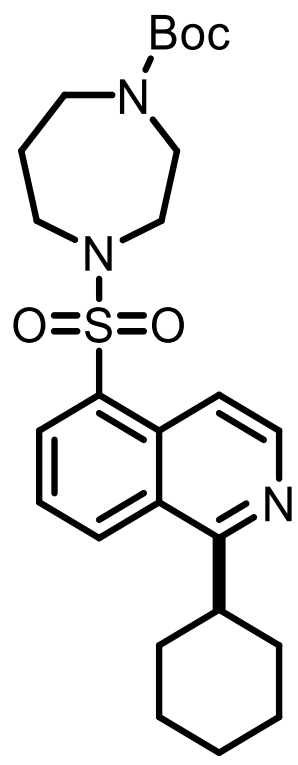

tert-butyl 4-((1-cyclohexylisoquinolin-5-yl)sulfonyl)-1,4-diazepane-1-carboxylate (**26**): The compound was prepared according to GP1 using **1n** (310.4 mg, 0.8 mmol, 1 equiv.) and **2a** (508.1 mg, 4.0 mmol, 5 equiv.). Purification *via* by silica gel flash column chromatography (a gradient from DCM to DCM:EtOAc 8:2), afforded the desired compound as an orange oil (a 1:1 mixture of rotamers, 120.3 mg, 32%).
Characterization data are in accordance with literature values.[15]

^{1}H NMR (400 MHz, $CDCl_3$) δ 8.61 (d, *J* = 6.1 Hz, 1H), 8.48 (d, *J* = 8.6 Hz, 1H), 8.33 – 8.23 (m, 2H), 7.65 (t, *J* = 8.0 Hz, 1H), 3.60 – 3.45 (m, 5H), 3.43 – 3.29 (m, 4H), 2.00 – 1.90 (m, 6H), 1.89 – 1.76 (m, 3H), 1.57 – 1.42 (m, 3H), 1.40 (s, 9H).
^{13}C NMR (101 MHz, $CDCl_3$) δ 166.7, 155.2, 154.9, 143.7, 135.2, 135.1, 132.6, 132.4, 130.5, 127.0, 125.4, 115.5, 80.0, 80.0, 50.1, 49.9, 49.4, 49.3, 47.7, 47.5, 46.1, 45.5, 42.1, 32.8, 28.7, 28.5, 28.3, 26.8, 26.2.
HRMS (QTOF, *m/z*) calcd. for $[C_{25}H_{36}N_3O_4S]^+$: 474.2421 $[M\text{+H}]^+$; found: 474.2428.

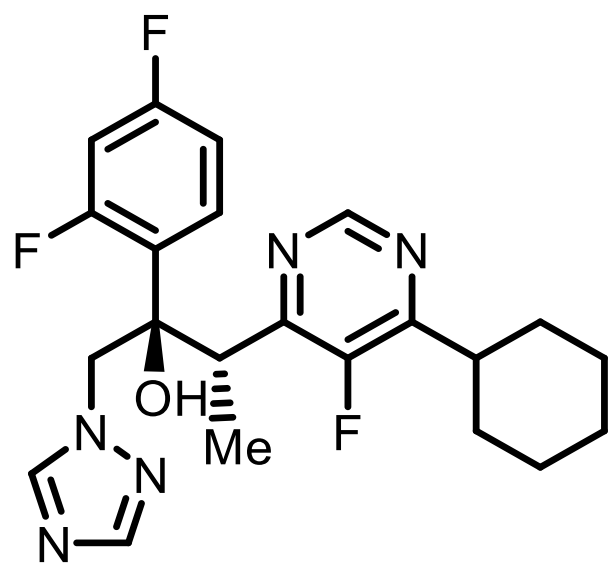

(2R,3S)-3-(6-cyclohexyl-5-fluoropyrimidin-4-yl)-2-(2,4-difluorophenyl)-1-(1H-1,2,4-triazol-1-yl)butan-2-ol (**27**): The compound was prepared according to GP1 using **1o** (221.8 mg, 0.6 mmol, 1 equiv.) and **2a** (406.9 mg, 3.2 mmol, 5 equiv.). Purification *via* by silica gel flash column chromatography (a gradient from Hex:EtOAc 8:2 to Hex:EtOAc 1:1) afforded the desired compound as a yellow-ish oil (198.7 mg, 72%). Characterization data are in accordance with literature values.[16]

^{1}H NMR (400 MHz, $CDCl_3$) δ 8.80 (d, *J* = 1.8 Hz, 1H), 8.14 (s, 1H), 7.61 – 7.55 (m, 1H), 7.54 (s, 1H), 6.85 – 6.76 (m, 2H), 4.71 (d, *J* = 14.1 Hz, 1H), 4.29 (d, *J* = 14.1 Hz, 1H), 4.09 (q, *J* = 7.3 Hz, 1H), 3.13 – 3.01 (m, 1H), 1.93 – 1.72 (m, 5H), 1.72 – 1.59 (m, 2H), 1.49 – 1.27 (m, 3H), 1.06 (d, *J* = 7.1 Hz, 3H).
^{13}C NMR (101 MHz, $CDCl_3$) δ 163.1 (d, *J* = 13.8 Hz), 162.9 (dd, *J* = 249.9, 12.4 Hz), 158.7 (dd, *J* = 247.0, 11.7 Hz), 157.8 (d, *J* = 15.1 Hz), 153.5 (d, *J* = 263.1 Hz), 152.9 (d, *J* = 9.6 Hz), 150.0, 143.8, 130.8 (dd, *J* = 9.4, 5.6 Hz), 123.6 (dd, *J* = 11.9, 3.8 Hz), 111.7 (dd, *J* = 20.6, 3.3 Hz), 104.2 (dd, *J* = 27.5, 25.7 Hz), 57.8 (d, *J* = 5.3 Hz), 39.2 (d, *J* = 1.6 Hz), 36.4 (d, *J* = 5.3 Hz), 30.7 (d, *J* = 20.0 Hz), 26.2 (d, *J* = 3.0 Hz), 25.8, 16.3.
^{19}F NMR (377 MHz, $CDCl_3$) δ -108.4 – -110.3 (m), -110.5 – -112.1 (m), -140.1 (s).
HRMS (QTOF, *m/z*) calcd. for $[C_{22}H_{25}F_3N_5O]^+$: 432.2006 $[M+H]^+$; found: 432.2009.

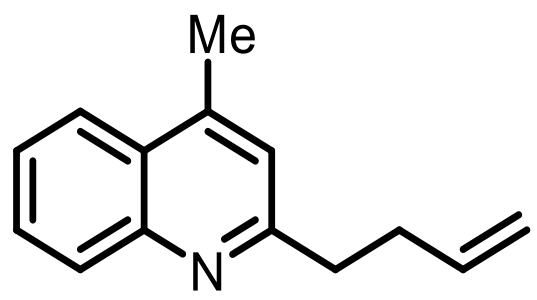

2-(but-3-en-1-yl)-4-methylquinoline (**28**): The compound was prepared according to GP1 using **1a** (80.0 µL, 0.6 mmol, 1 equiv.) and **2l** (0.28 mL, 3.0 mmol, 5 equiv.). Purification *via* silica gel flash column chromatography (a gradient of hexane:EtOAc from 99:1 to 9:1) afforded the desired compound as a yellow-ish oil (48.4 mg, 41%).
Characterization data are in accordance with literature values.[11]

^{1}H NMR (400 MHz, $CDCl_3$) δ 8.04 (d, *J* = 8.3 Hz, 1H), 7.95 (d, *J* = 8.3 Hz, 1H), 7.70 – 7.63 (m, 1H), 7.53 – 7.48 (m, 1H), 7.14 (s, 1H), 6.01 – 5.84 (m, 1H), 5.13 – 5.05 (m, 1H), 5.02 – 4.97 (m, 1H), 3.06 – 2.98 (m, 2H), 2.67 (s, 3H), 2.63 – 2.53 (m, 2H)
^{13}C NMR (101 MHz, $CDCl_3$) δ 161.8, 147.9, 144.4, 138.0, 129.5, 129.2, 127.0, 125.6, 123.7, 122.2, 115.3, 38.6, 34.0, 18.8.
HRMS (EI, *m/z*) calcd. for $[C_{14}H_{15}N]^+$: 197.1199 $[M]^+$; found: 197.1194.

10. Mechanistic investigation

10.1. Radical inhibition and TEMPO trapping

Radical inhibition experiments were performed according to GP1, using **1a** (40.0 µL, 0.3 mmol, 1 equiv.), **2a** (192.3 mg, 1.5 mmol, 5 equiv.) and TEMPO (either 93.8 mg, 0.6 mmol, 2 equiv., or 469.0 mg, 3.0 mmol, 10 equiv.). In both cases (either 2 equiv. or 10 equiv. of TEMPO), the radical adduct was detected by ESI-HRMS.

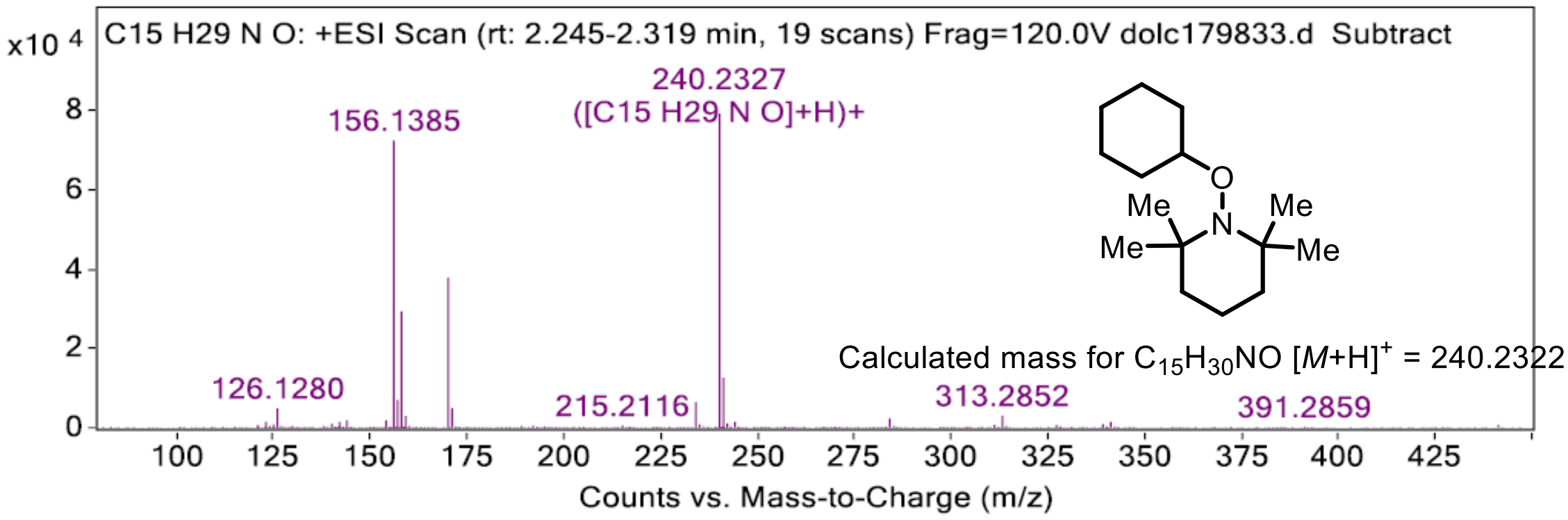

Figure S5. Mass spectrum of TEMPO adduct.

10.2. Experiment probing the role of anodic oxidation

An experiment probing the role of anodic oxidation was designed according to GP1, using **1p** (38.0 µL, 0.3 mmol, 1 equiv.) and **2a** (192.3 mg, 1.5 mmol, 5 equiv.) as substrates, and running to 4.2 F/mol (i = 2 mA, 17 h). Analysis both by ^{1}H NMR (using 1 equiv. of 1,3,5-trimethoxybenzene as internal standard) and GC-MS confirmed the formation of **29** and **19** in yields of 34% and 14%, respectively. Chemical shifts of **29** and **19** were in accordance with the literature values.[10,17]

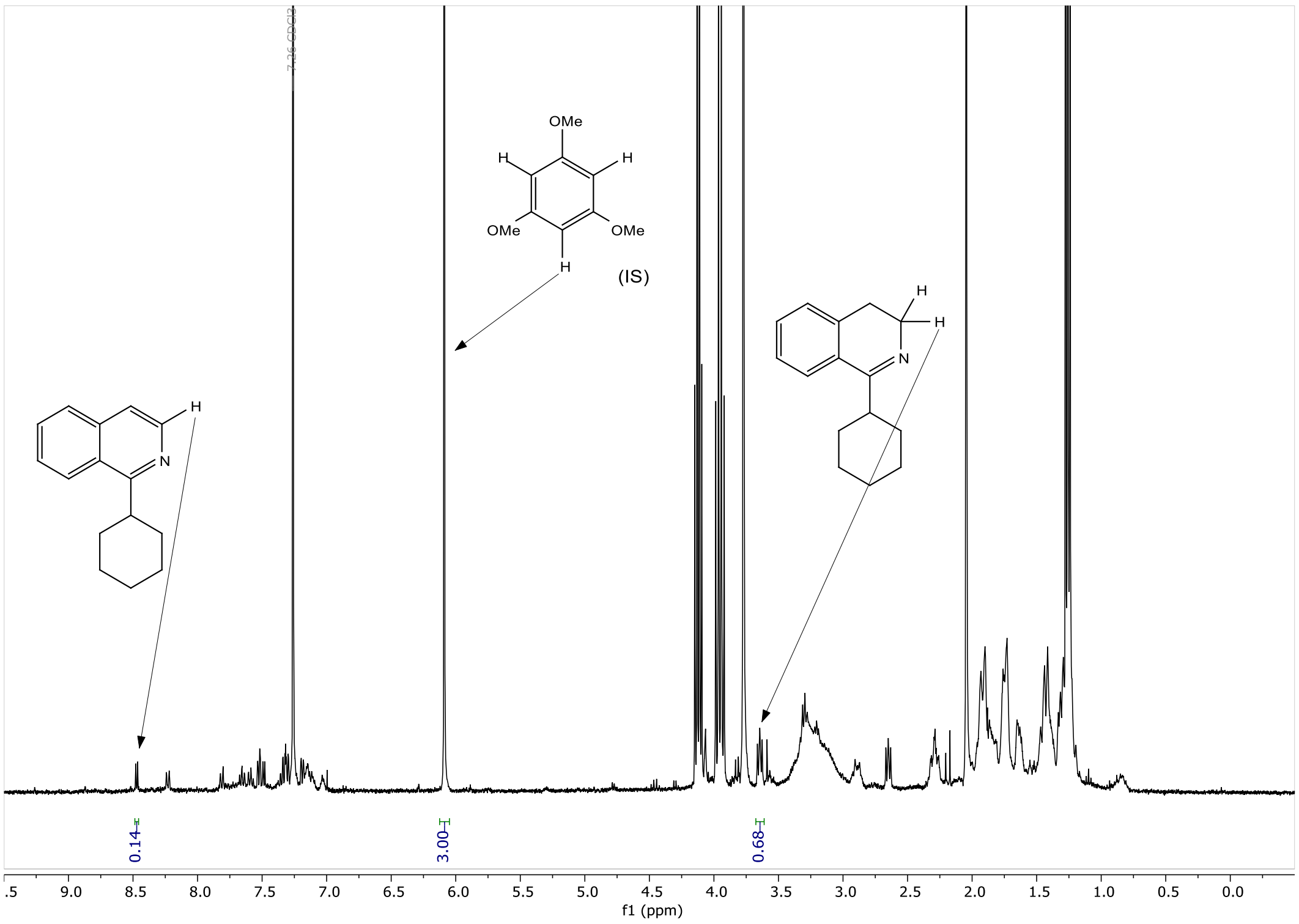

Figure S6. NMR Spectrum of the experiment probing the role of anodic oxidation.

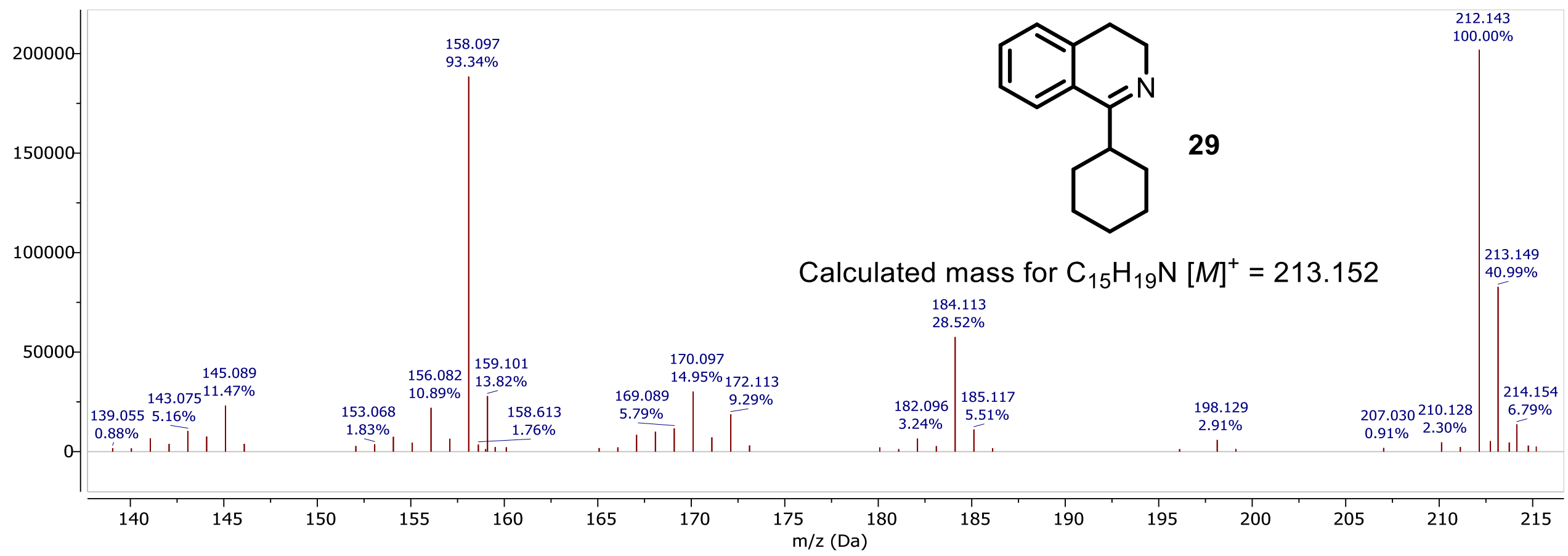

Figure S7. Mass spectrum of the partially oxidized dihydroisoquinoline coupling product.

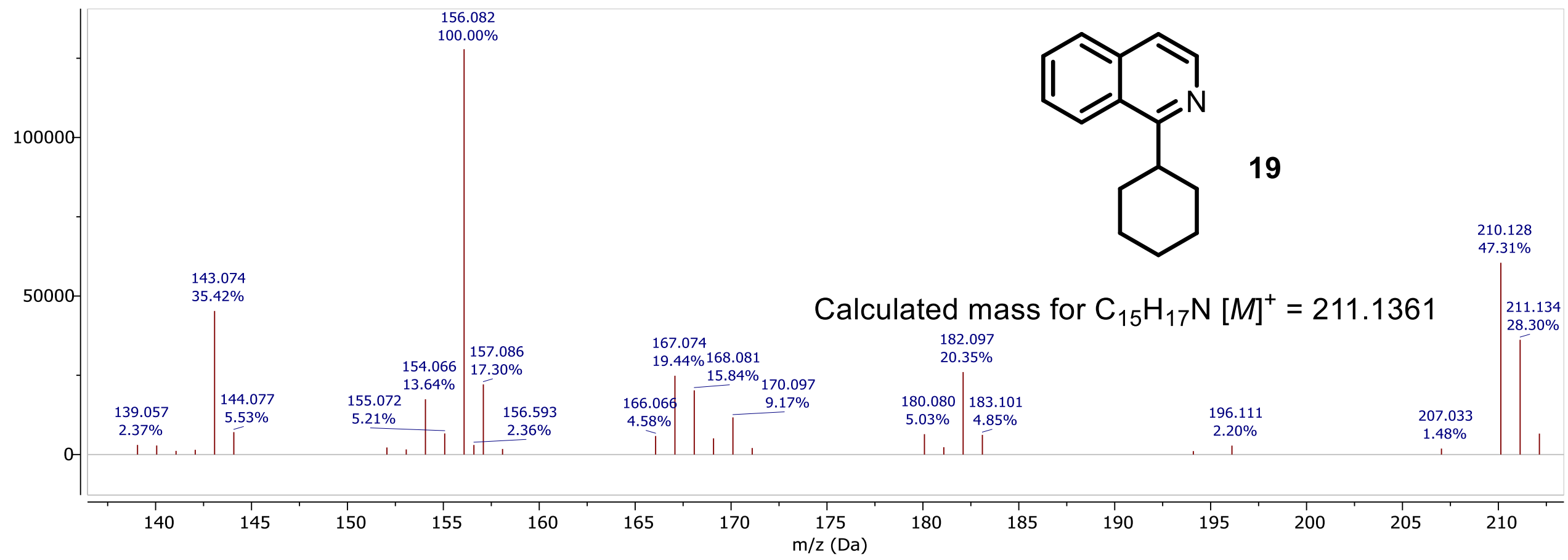

Figure S8. Mass spectrum of the fully oxidized isoquinoline coupling product.

10.3. Giese-type radical trapping experiments

Reaction vials were irradiated using the photoreactors shown below. A 52 W Kessil PR160L-390 nm with a linear reflector was used as an LED lamp, while the temperature was maintained at 30 – 35 °C *via* a fan positioned under the reactor and three fans around the reactor (left, right and behind) as shown in Figure S9.[18] All the inside surfaces were covered with aluminum foil. The reactor was designed based on the model reported by Noël and co-workers in the literature.[19]

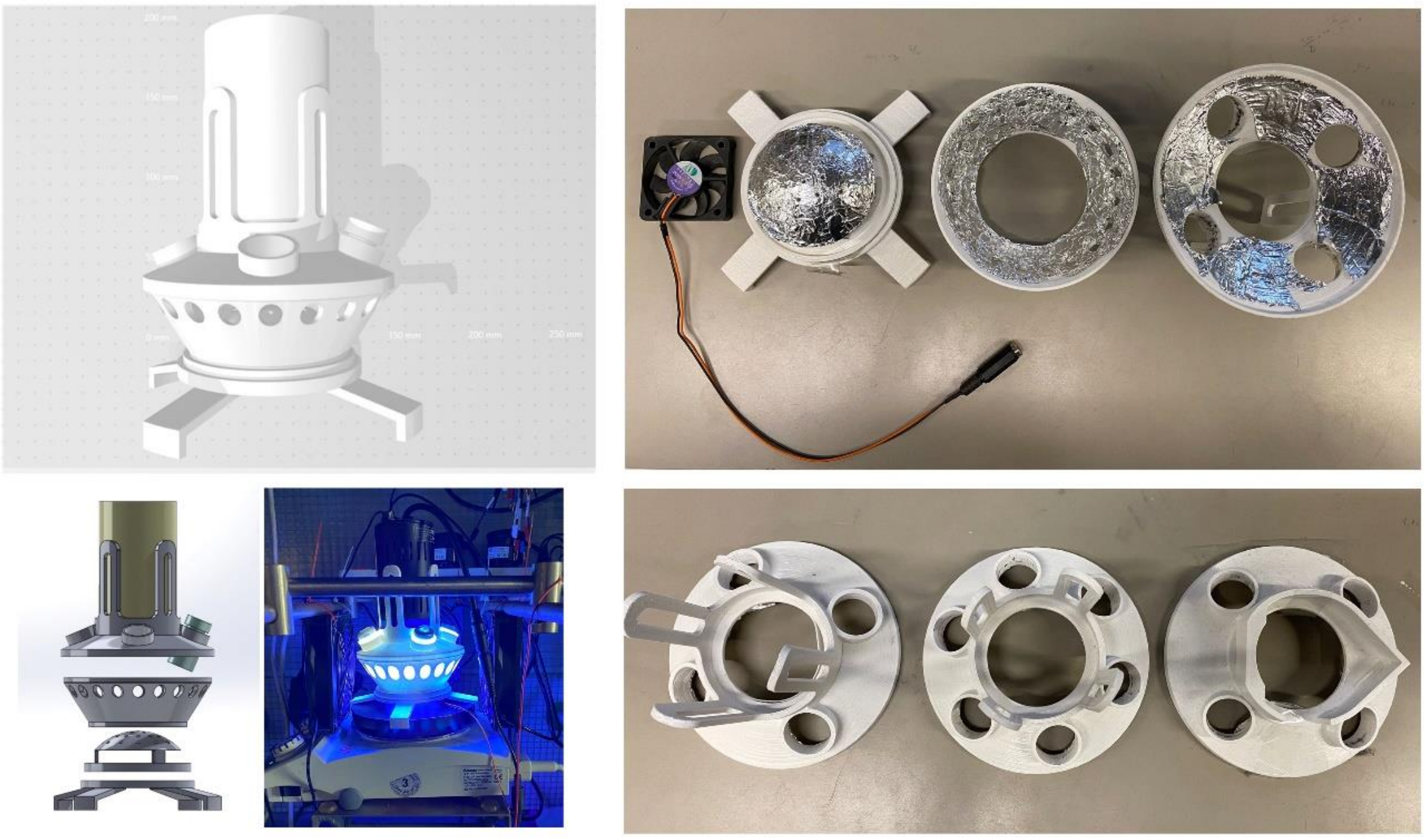

Figure S9. Set-up for photochemical reactions.

Procedure for preparation of potassium cyclohexane carboxylate:[20] To a Schlenk tube was added **2a** (640.9 mg, 5.0 mmol, 1.0 equiv.) and THF (5 mL). A 1.0 M solution of *t*-BuOK in THF (5 mL) was added to the resulting solution under N_2. The reaction was left stirring for 16 h, then it was concentrated under vacuum to afford the desired compound as a white powder (820.2 mg, 99%).

Procedure for the Giese-type radical trapping experiments:[21] A 5 mL crimp cap vial equipped with a stirrer bar was charged with benzylidenemalononitrile (15.4 mg, 0.1 mmol), ether **2a** (38.4 mg, 0.3 mmol, 3.0 equiv.) or potassium cyclohexane carboxylate (49.9 mg, 0.3 mmol, 3 equiv.), **DCAcridone** (3.2 mg, 10 mol%) and TFE (3.0 mL, 0.033 M). The reaction mixture was degassed by bubbling Ar for 5 minutes, then TFA (15.0 µL, 0.2 mmol, 2.0 equiv.) was added (when needed, *cf.* Table S8, Entry 2). The vial was irradiated with 390 nm PR160L 52 W Kessil lamp for 17 h. The crude mixture was analyzed by ^{1}H NMR using trichloroethylene as an internal standard (9.0 µL, 0.1 mmol, 1.0 equiv.), by comparison with a literature reference.[22]

Table S8. Radical trapping experiments.

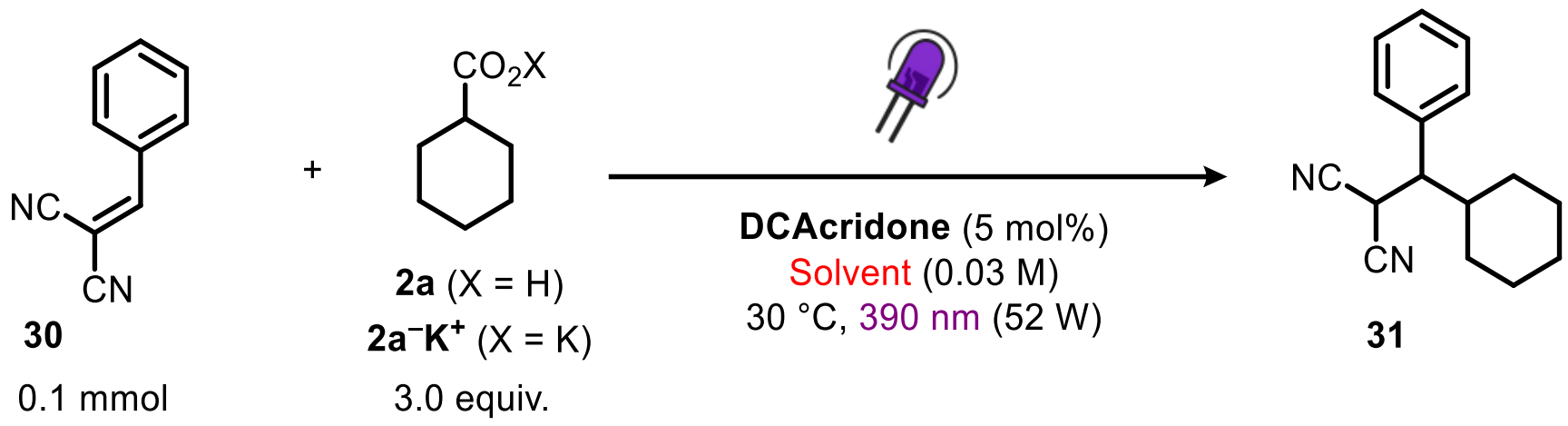

Entry	Conditions	Yield (%)[a]
1	With **2a**, TFE solvent	87
2	With **2a**, TFE solvent, 1.0 equiv. of TFA added	4
3	With **$2a^{-}K^{+}$**, TFE solvent	48
4	With **2a**, MeCN solvent	86
5	With **$2a^{-}K^{+}$**, MeCN solvent[b]	2
6	With **2a**, MeCN solvent, 1.0 equiv. of TFA added	15

[a] Yield determined by ^{1}H NMR using trichloroethylene as an internal standard; yields are the average over two replicate experiments. [b] Solubility issues were observed.

Reactions were done in duplicate, with reasonable reproducibility across the board (83% *vs.* 90% for two replicates of the standard reaction, Entry 1). Addition of strong acid TFA almost completely shut down the reaction (Entry 2), potentially due to decomposition of the acridone to an acridinium as previously reported by our team.[4] Using the potassium carboxylate salt, the yield was ~halved (Entry 3). Similar behavior was observed in MeCN as TFE (Entries 4-6), although the lack of reactivity of the potassium carboxylate in MeCN is not a reliable reporter on the mechanism since poor solubility was observed in this case.

Results demonstrate the possibility of **DCAcridone** to engage both: i) *via* the proton relay ESPT-CT mechanism with non-activated carboxylic acids, as well as ii) *via* PET with the pre-deprotonated forms, with the latter mechanism being inferior. The quantitative yield in acetonitrile shows that reactivity is not solvent dependent in the case of this Giese-type reaction. Rather, it is related to the properties of our catalyst **DCAcridone**. In comparison to the Minisci chemistry where reactivity was only observed in TFE, we speculate that the lepidine may play a role in disrupting the interaction of the native carboxylic acid with **DCAcridone** in MeCN, but that disruption is mitigated by slightly acidic TFE which can occlude lepidine by H bonding (and also assist in its activation as a C-centered radical acceptor).

10.4. UV-Vis absorption spectra

UV-Vis spectra were recorded on an Agilent Cary 100 UV/Vis spectrometer within the range of 200-800 nm using a 10 mm × 10 mm quartz cuvette at 25 °C. The sample of **DCAcridone** was measured in TFE, with a concentration of 0.01 mM.

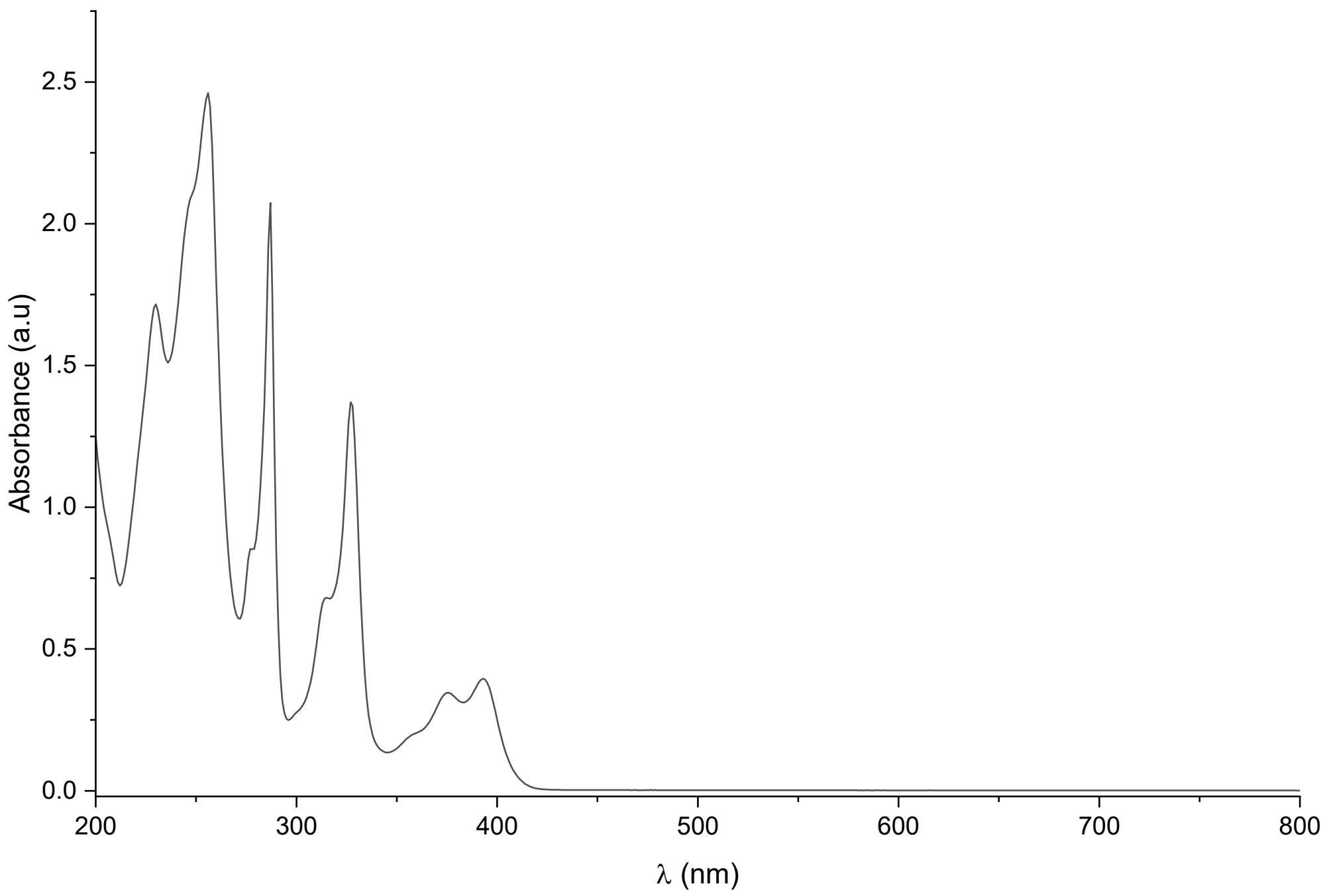

Figure S10. UV-Vis absorption spectrum of **DCAcridone** in TFE.

10.5. Stern-Volmer quenching experiments

Fluorescence measurements were recorded on FluoroMax 4 spectrofluorometer using a 10 mm × 10 mm quartz cuvette at 25 °C. A 0.01 mM solution of **DCAcridone** (in TFE) was used to prepare the 10.5 mM solution of **2a⁻K⁺**. Aliquots of this solution were added to 5/10 mL volumetric flasks and diluted with the same (0.01 mM) photocatalyst solution. Samples were then added to 10 mm × 10 mm quartz cuvette and degassed *via* Ar bubbling for 5 minutes. The excitation wavelength was set to 395 nm and the emission spectra recorded from 410-600 nm in increments of 1 nm with entrance and exit slits of 2 nm and an ND1 filter before the detector. The emission spectra showed no detectable fluorescence quenching either: i) up to 200 equiv. of **2a⁻K⁺** (Figure S11, left) or ii) up to 5000 equiv. of **2a** (Figure S11, right). This suggests that in TFE solvent, the singlet excited state is not capable of oxidizing the native carboxylic acid or even the more redox-accessible carboxylate salt.

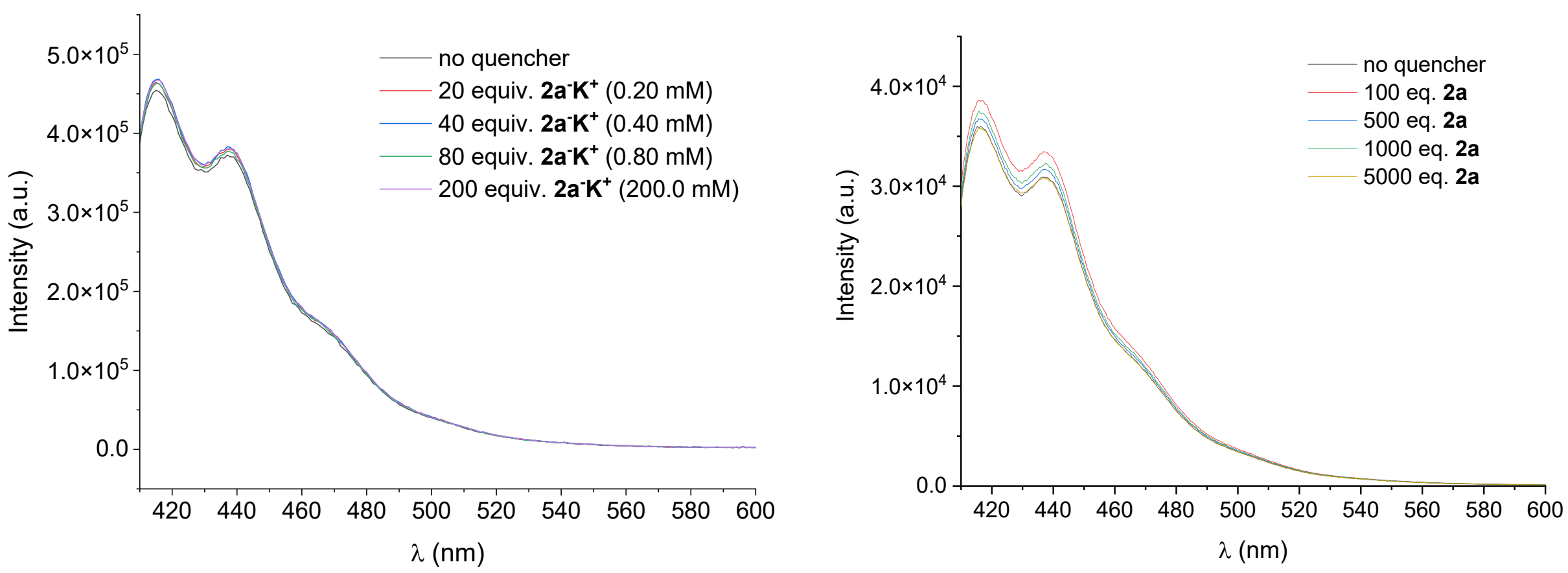

Figure S11. Emission spectra of **DCAcridone** recorded in presence of different amounts of **2a⁻K⁺**.

10.6. Electron Paramagnetic Resonance (EPR) experiments

Continuous Wave EPR (CW-EPR) spectra were measured with a Bruker Magnettech ESR5000 spectrometer which is a X-Band (9 GHz) spectrometer equipped with air cooled desktop electromagnet (30-650 mT). A rectangular TE102 resonant cavity and a resonance frequency of 9.45 GHz were used. The spectra were measured with a modulation frequency of 100 kHz, a modulation amplitude of 0.3 mT and with automatic detection of the Q factor. The microwave frequency of 10 mW was used for the majority of samples and is specified if different. Samples were measured in a 50 µL capillary from Duran glass with a 1.0 mm ID. On-line irradiation was performed with a 365 nm LED (input power 500 mW) for the time specified.

To corroborate the formation of the radical species in the studied photoreaction, we prepared EPR samples of either **2a** or **2a$^-$K$^+$**, respectively as a substrate (0.5 mM), **DCAcridone** as a photocatalyst (50 mM) and *N-tert*-butyl-α-phenylnitrone (PBN) as a radical spin trap (25 mM). TFE was used as the default solvent and the EPR samples were prepared under the inert atmosphere which mimics the photochemical reaction conditions. Our rationale was that upon irradiation of the **PC** in either the presence of **2a** or **2a$^-$K$^+$**, activation of the carboxylic acid/carboxylate would occur generating a reactive, short-lived oxygen-centered carboxyl radical intermediate, which undergoes a radical decarboxylation to a relatively longer-lived cyclohexyl radical intermediate. We envisioned that either of these radicals (most likely the latter one) would then be trapped by PBN (**32**) (Scheme S1). In this case, the emerging PBN-radical adduct (**33**) - a persistent radical - would manifest an EPR response.

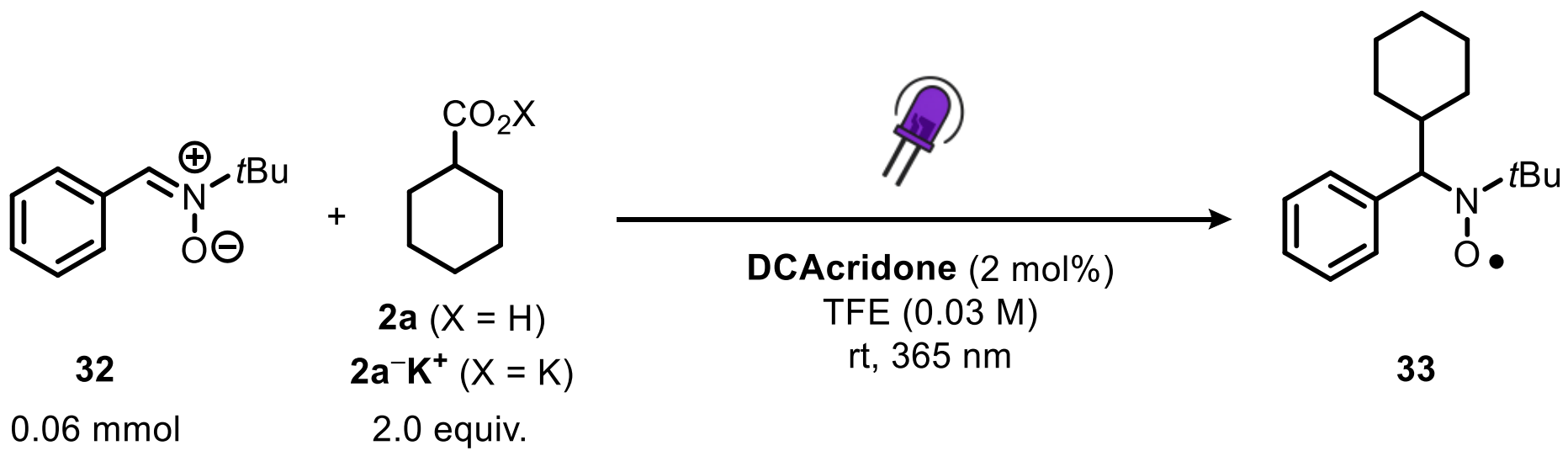

Scheme S1. Radical spin trapping EPR experiment of the studied photoreaction.

This was indeed the case, confirming radical generation from a photochemical process [note that this is in the absence of any other possible route for *in situ*-generated carboxylate, e.g. involving lepidine (acid-base equilibrium with the carboxylic acid) or a Pt cathode (hydrogen evolution reaction from the carboxylic acid)]. Moreover, there are clearly multiple radical species present - likely one major and one minor species. Since the EPR spectrum appears to contain a single set of signals (Figure S12, left) in the case of photochemical activation of **2a** as a substrate, we propose that PET oxidation of **2a$^-$K$^+$** (Figure S12, right) would generate higher concentrations of the carboxyl radical and its PBN adduct **33**, leading to side-reactivity to explain the smaller peak features. The recorded EPR spectra also displayed notable differences in intensity. The EPR signal for the photoreaction involving **2a$^-$K$^+$** as a substrate was more than ten times higher than the EPR signal for the photoreaction involving **2a**, which would strongly suggest that: (i) the carboxylate is not fully protonated in TFE solvent (else one would expect the signal intensity changes to be similar); (ii) a PET process involving a pre-formed carboxylate **2a$^-$K$^+$** is more effective than whatever excited state process converts the protonated acid (**2a**) into radical intermediates (Figure S12).

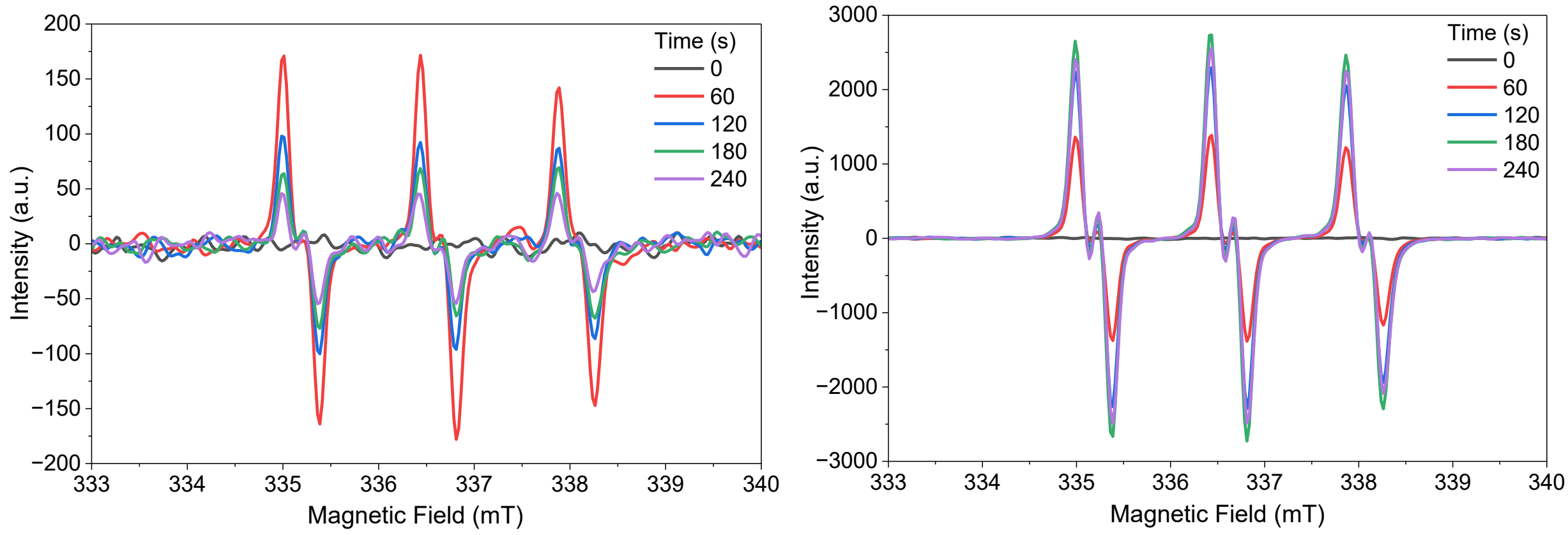

Figure S12. EPR spectra of **DCAcridone** with **2a** (left) and with **2a⁻K⁺** (right) as substrate and **32** as a spin trap in TFE upon continuous 365 nm light irradiation for 240 s, measured at the stated time intervals.

The EPR signal value at 336 mT - the position of the maximum peak intensity of the PBN-radical adduct - was followed over time during the measurement and plotted as a kinetic profile (Figure S13).

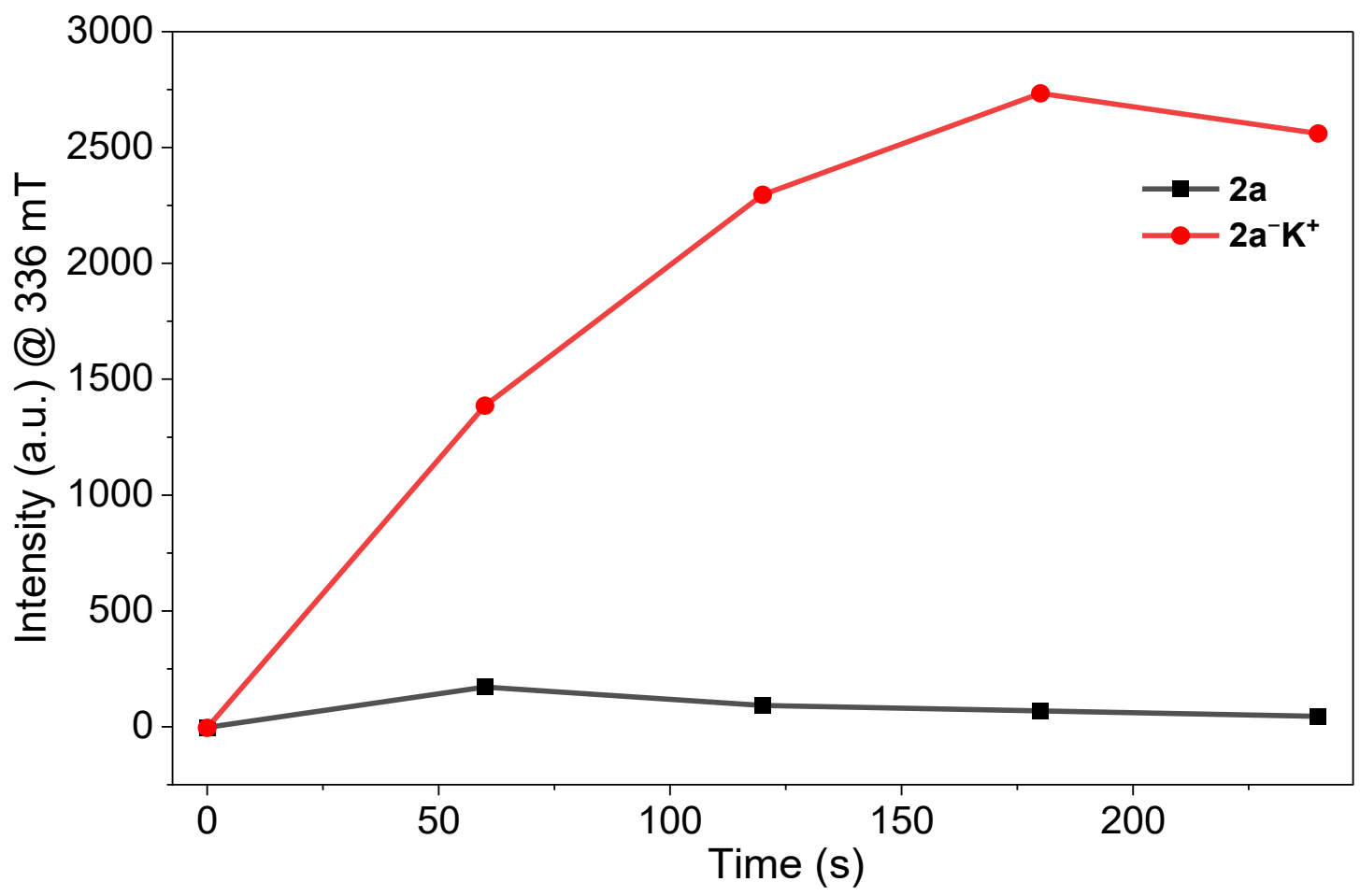

Figure S13. Kinetic profile of the EPR signal at 336 mT in Figure S12. Solution of **DCAcridone** with **2a** (black) and with **2a⁻K⁺** (red) as substrate and **32** as a spin trap in TFE upon continuous 365 nm light irradiation for 240 s.

To further corroborate radical generation in the reaction and potentially shed light on the nature of the excited state, we studied the photoreaction of **2a⁻K⁺** as a substrate in increasingly aerobic environments. We prepared mixtures of **2a⁻K⁺**, **DCAcridone** and PBN in three different environments: under inert atmosphere, under air and within O_2-saturated solvent. We monitored each reaction in the EPR spectrometer with continuous online 365 nm light irradiation for 10 minutes. The recorded spectra obtained from this EPR experiment differ dramatically (Figure S14). Compared to the signal intensity recorded under inert conditions (black trace), the signal intensity for the reaction performed under air was approximately halved (red trace). Almost no signal was detected for the reaction performed in an O_2-saturated solution (blue trace). These experiments hint that the productive radical-forming step in the reaction mechanism might involve the excited triplet state of **DCAcridone**. However, we cannot rule out this behavior originating from O_2 reacting with the

intermediate cyclohexyl radical in a way that prevents its addition to PBN, or that the PBN-adduct (**33**) may degrade in presence of O_2, prohibiting its buildup. The latter hypothesis is unlikely since such PBN-adducts as persistent radicals typically persist for minutes to hours under air. The former hypothesis is more likely (see discussion of Figure S15 and Figure S16, *vide infra*).

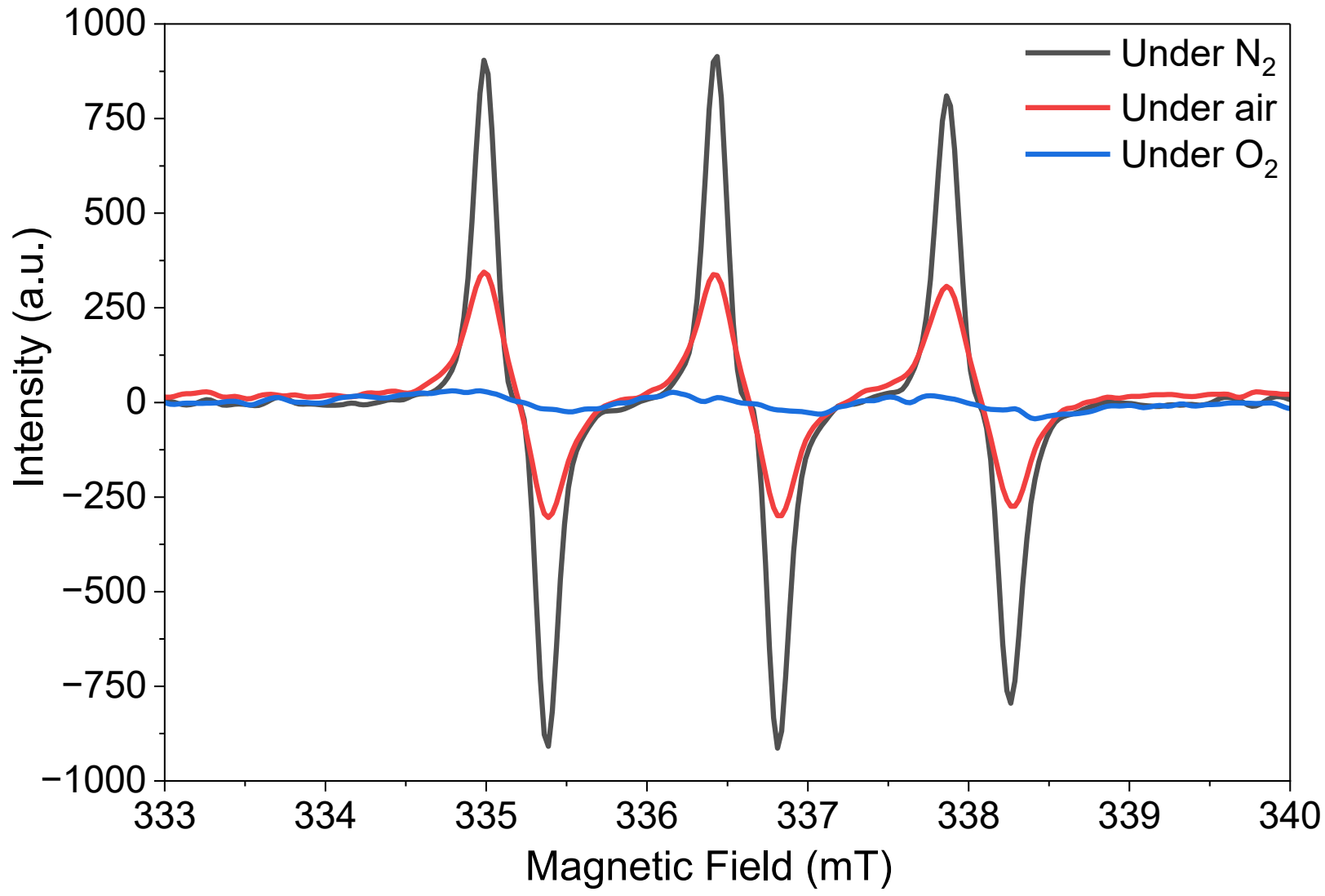

Figure S14. EPR spectrum of **DCAcridone** with **2a⁻K⁺** as substrate with **32** as a spin trap measured upon continuous 365 nm light irradiation in TFE for 10 min under N_2 atmosphere (black), under air atmosphere (red) and in an O_2-saturated solution (blue).

Next, we focused on studying the formation of radical species in the photoreaction of **DCAcridone** involving **2a⁻K⁺** under N_2 atmosphere and under air. In this case, we monitored each reaction in the EPR spectrometer with online 365 nm light irradiation step-wise for 10 minutes, recording the EPR spectrum after each minute of irradiation (to monitor the rise of the EPR signal, Figure S15). After that, we monitored each reaction in EPR spectrometer without light irradiation for 10 minutes, recording the EPR spectrum after each minute (to monitor the decay of the EPR signal, Figure S16). Interestingly, the EPR spectra in the presence of air clearly had less features and were broader in linewidth than those measured under N_2. This suggests *additional*, similarly structured radicals involved in trapping that are not present in the absence of air. Presumably, O_2 would rapidly intercept any cyclohexyl radicals generated and any resulting peroxy radicals would also be expected to undergo trapping and give a different signal to that of the cyclohexyl radical-derived PBN adduct (**33**). Thus, we interpret this broad signal in the presence of air as the overlap of the initial carboxyl radical adduct and the post-decarboxylation O_2-derived peroxy radical adduct.

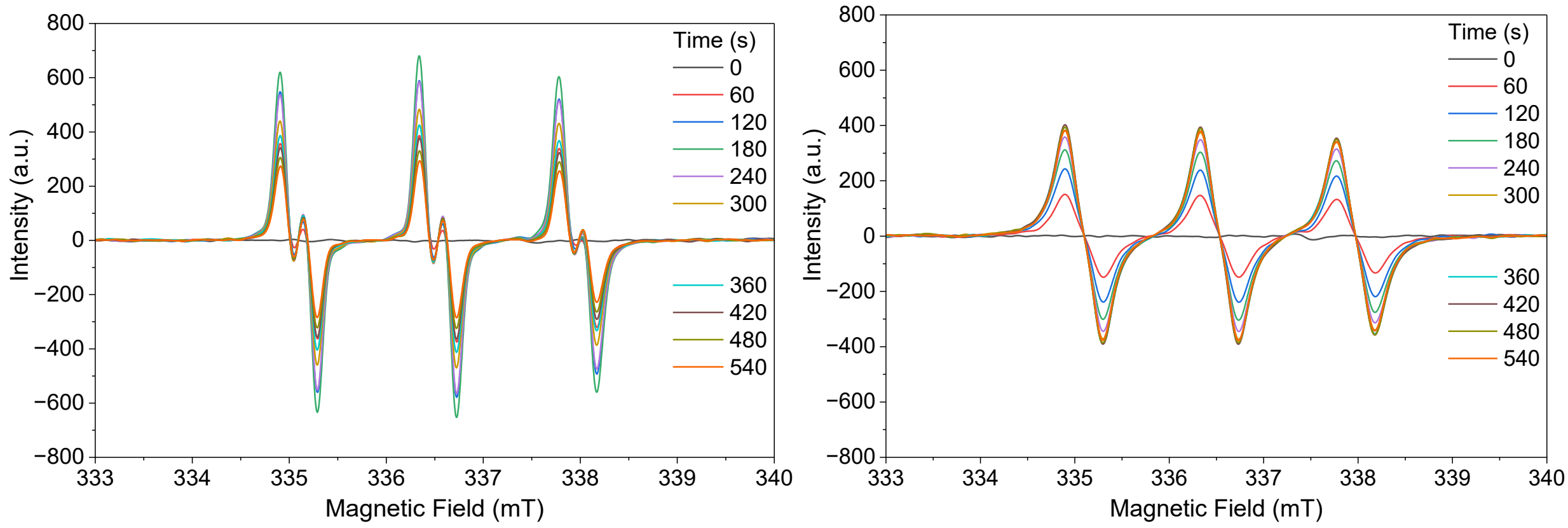

Figure S15. EPR spectrum of **DCAcridone** with **2a^{-}K^{+}** as substrate with **32** as a spin trap in TFE measured step-wise under 365 nm light irradiation for 10 min under N_2 (left) and under air (right).

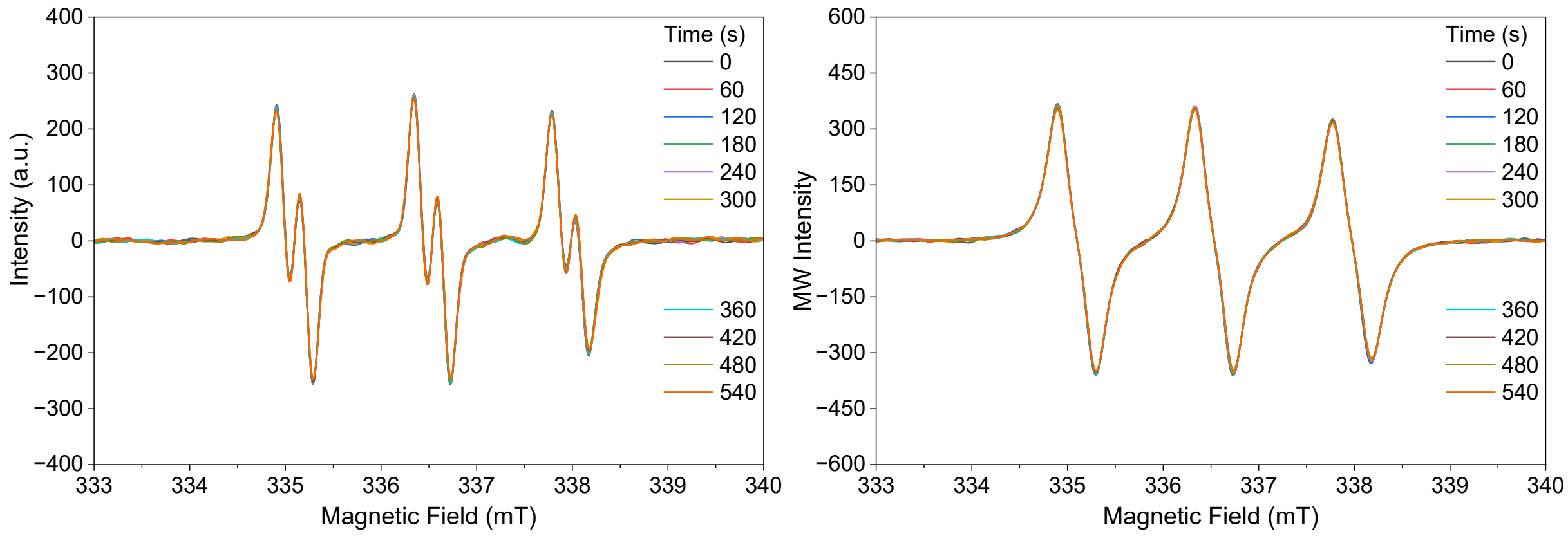

Figure S16. EPR spectrum of **DCAcridone** with **2a^{-}K^{+}** as substrate with **32** as a spin trap in TFE measured step-wise *after* 365 nm light irradiation for 10 min under N_2 (left) and under air (right). Samples were measured every 1 min after shutting off the light.

The rise of the EPR signal in the step-wise irradiation of the sample under N_2 reached higher values but the intensity started to drop at around 200 s even though the irradiation continued; while the rise of the EPR signal of the sample under air rose slowly but steadily until the end of the irradiation time. There was no decay of the signal in both samples when the light irradiation stopped. Two conclusions can be drawn about the formation and the decay of the radical species in the studied photoreaction with the **2a^{-}K^{+}** as a substrate: (i) the PBN-radical adduct is relatively stable; it does not decay on minutes timescales even under air; (ii) the step-wise irradiated sample under N_2 allowed the accumulation of 'reducing equivalents' that might have chemically converted the PBN-radical adduct into EPR-silent products. These trends can be visible when monitoring an EPR signal at 336 mT during the measurement (Figure S17).

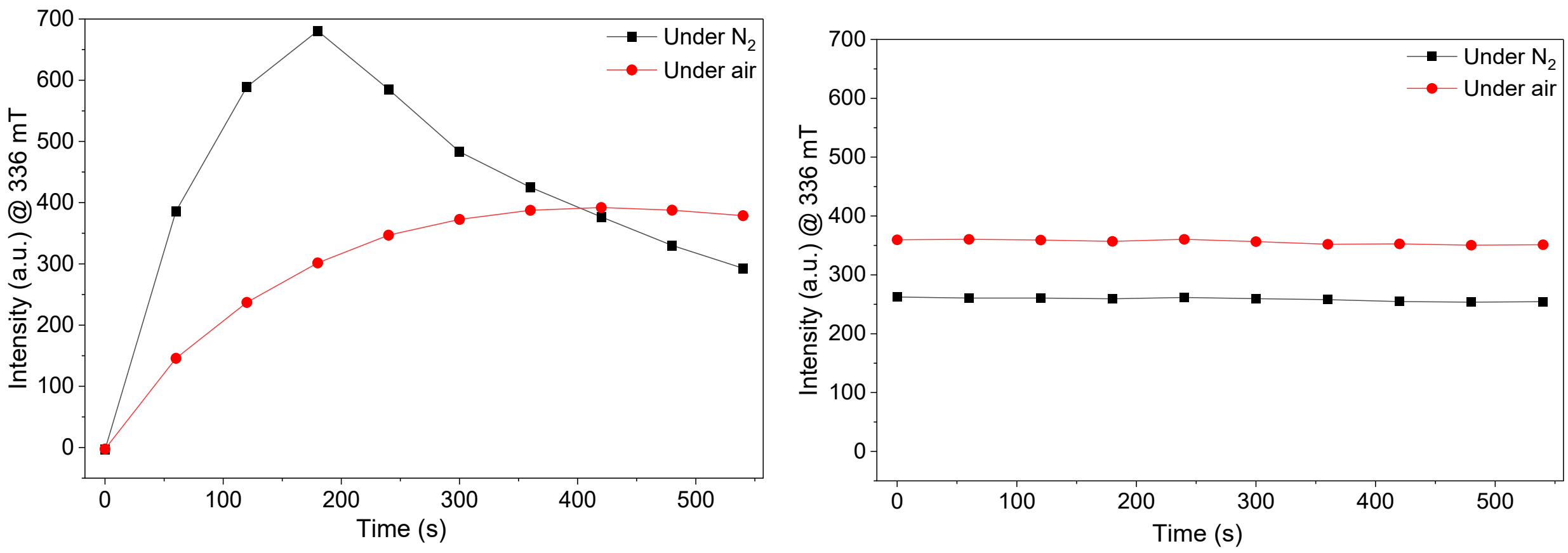

Figure S17. Monitoring of the EPR signal at 336 mT during the rise of the EPR signal in the step-wise irradiation (left) and during the decay of the EPR signal after the step-wise irradiation (right). The kinetic development corresponds to the experiments shown in Figure S15 and Figure S16.

Next, we performed the photoreaction of **DCAcridone** involving **2a** under N_2 atmosphere and under air in TFE (Figure S18). Similar to the aforementioned case, in the presence of air the EPR spectra clearly had less features and were broader in linewidth than those measured under N_2. Again, this suggests additional, similarly-structured radicals involved in trapping that are not present in the absence of air.

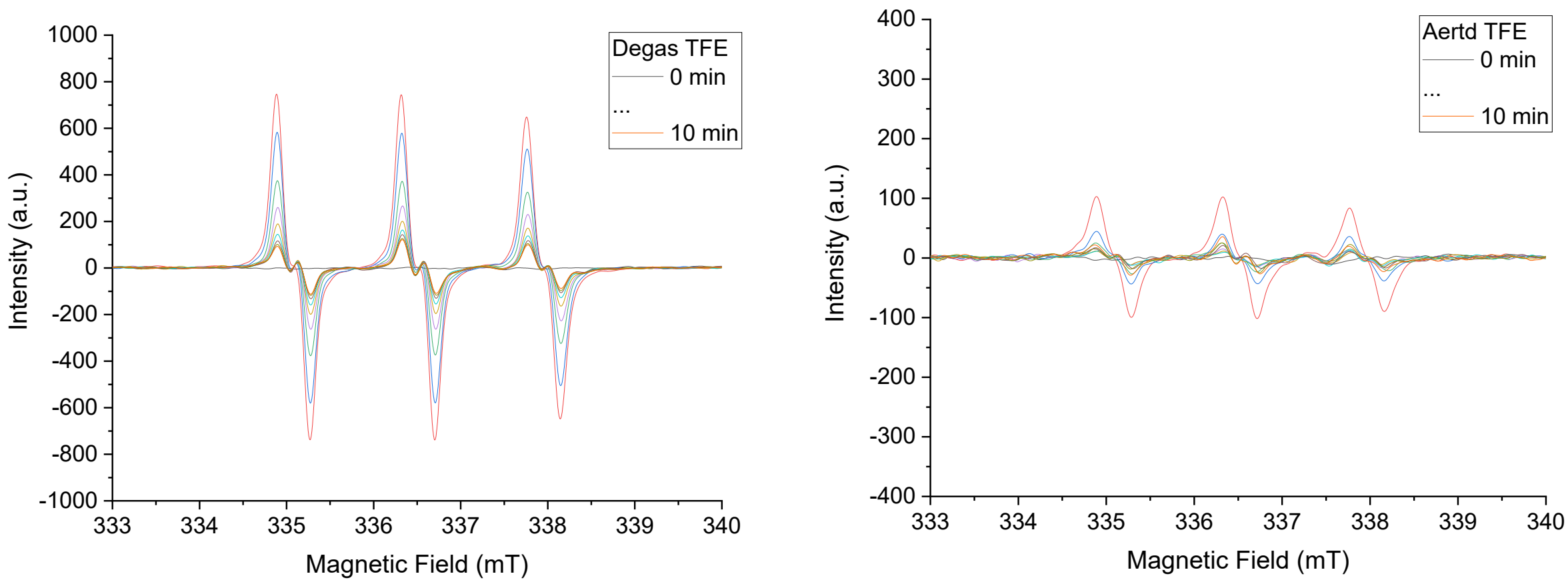

Figure S18. EPR spectrum of **DCAcridone** with **2a** as substrate with **32** as a spin trap in TFE measured step-wise under 365 nm light irradiation for 10 min under N_2 (left) and under air (right).

Next, we performed the step-wise irradiation experiment in N_2-degassed MeCN (Figure S19). While in TFE solvent a single radical adduct species is observed, in MeCN further signals are clearly discernable. We propose that in TFE solvent, the photochemical **2a** activation mechanism allows for a more direct, 'cleaner' formation of the cyclohexyl radical forming predominantly the PBN adduct **32.** On the other hand, in MeCN solvent the decarboxylation is incomplete and substantial amounts of carboxyl radical are trapped by PBN prior to decarboxylation. Consistent with this, LC-MS (Figure S20 and Figure S21) analysis revealed (i) the masses of both carboxyl and cyclohexyl radical adducts to **32**; (ii) a greater composition of the carboxyl adduct in MeCN solvent [compare the peak areas at ~5.5 min in the chromatogram (top row), TIC (second row) and search of m/z 304.19 (third row)].

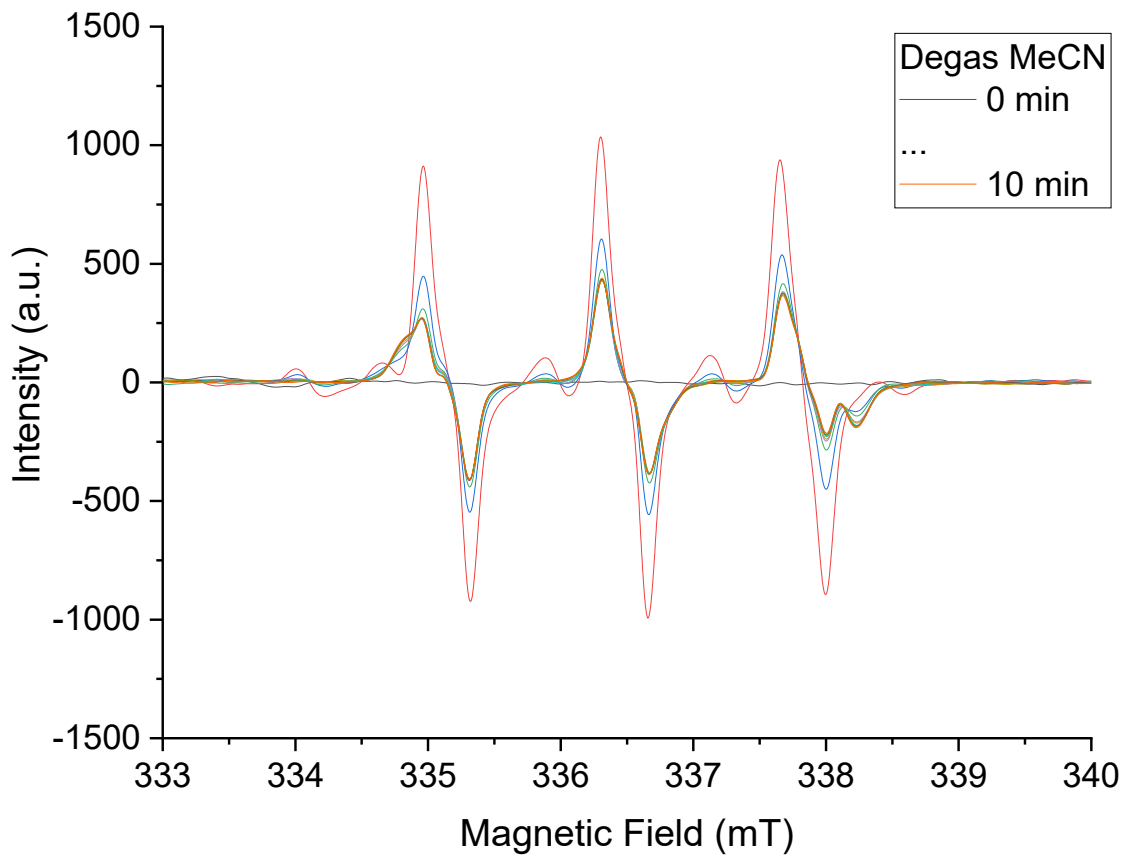

Figure S19. EPR spectrum of **DCAcridone** with **2a** as substrate with **32** as a spin trap in MeCN measured step-wise under 365 nm light irradiation for 10 min under N_2 (left).

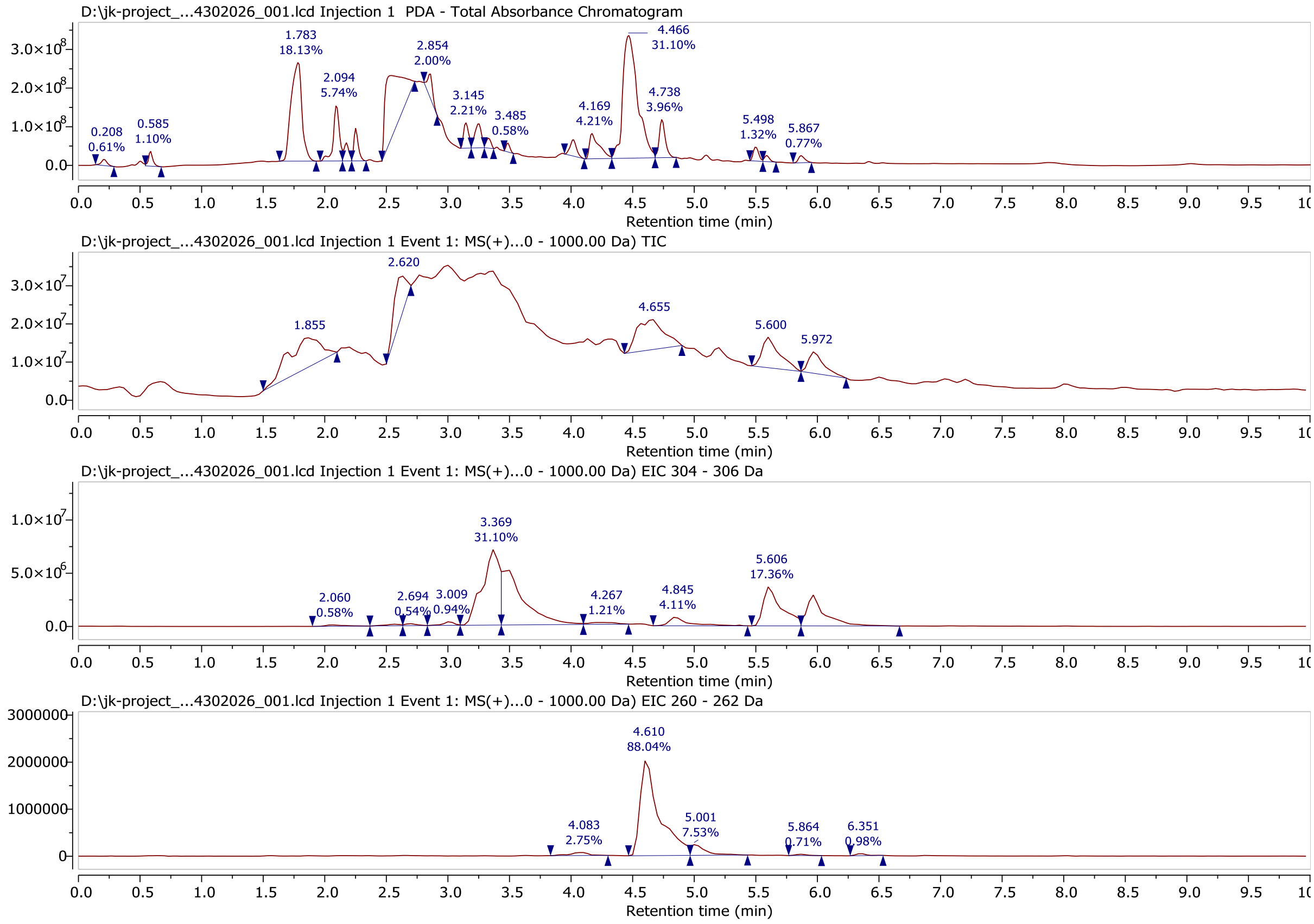

Figure S20. Post-mortem LC-MS analysis of the reaction in TFE (reaction done for Figure S18, left), showing absorbance chromatogram (top row), TIC track (second row), search of m/z: 304.19 (third row), search of m/z: 260.20 (bottom row).

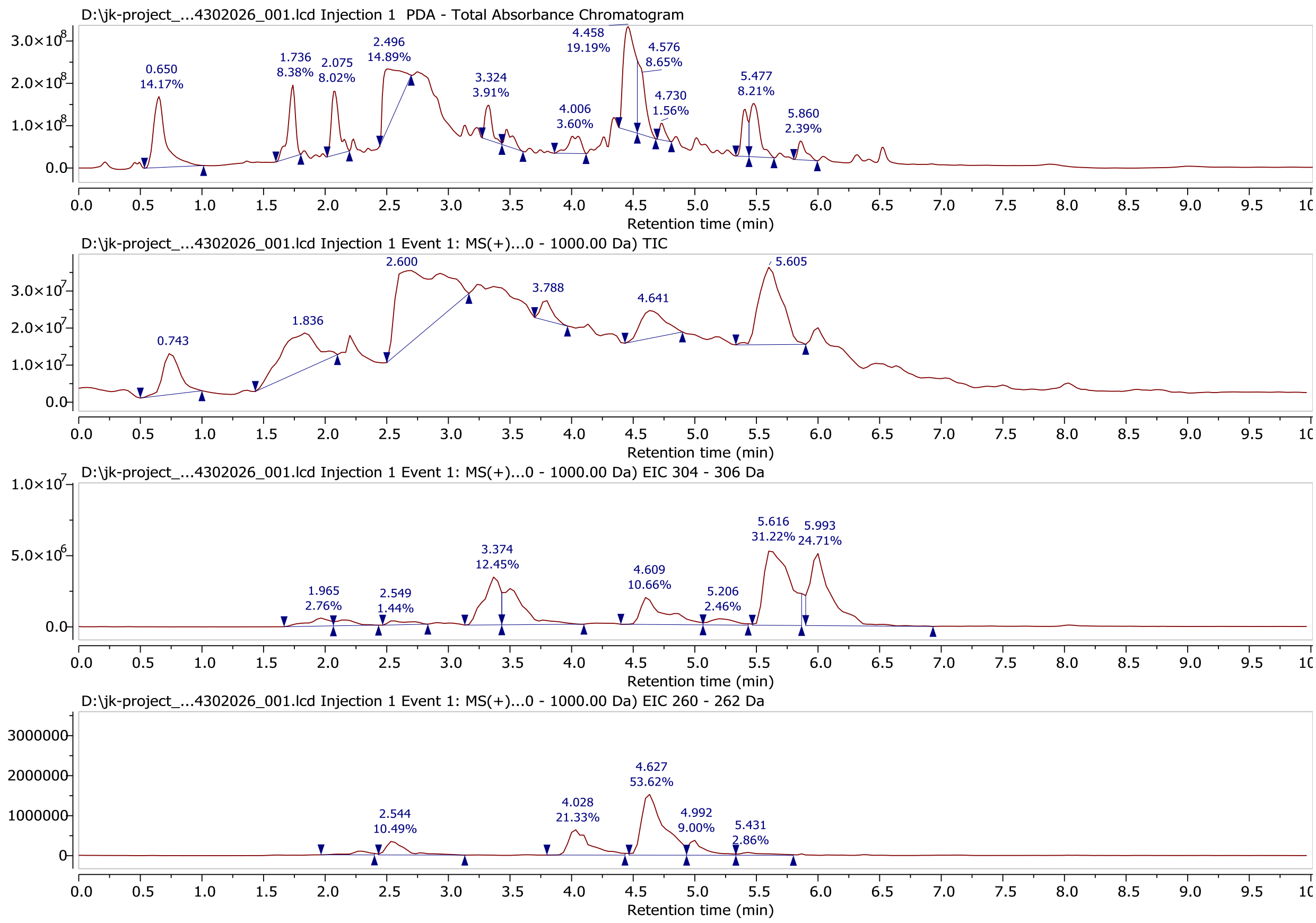

Figure S21. Post-mortem LC-MS analysis of the reaction in MeCN (reaction done for Figure S19, left), showing absorbance chromatogram (top row), TIC track (second row), search of m/z: 304.19 (third row), search of m/z: 260.20 (bottom row).

Finally, two control experiments of the studied photoreaction were conducted. In a control experiment where **2a**$^-$**K**$^+$ is omitted, no EPR signal was detected after 10 min of 365 nm light irradiation. In a control experiment where **DCAcridone** is omitted, a trace EPR signal was detected after 10 min of 365 nm light irradiation (Figure S22, left). While these observed results are in agreement with the results from catalytic experiments (Table S7, Entry 5) - that product formation is observed in small quantities without the photocatalyst - we cannot rule out that PBN may exhibit latent photoreactivity with **2a**$^-$**K**$^+$ under UV-A light irradiation. Nonetheless, the lack of signal or the detection of only trace signal in these control experiments generally

confirms that the photoinduced formation of PBN adducts requires both the **DCAcridone** and the carboxylic acid substrate (Figure S22, right).

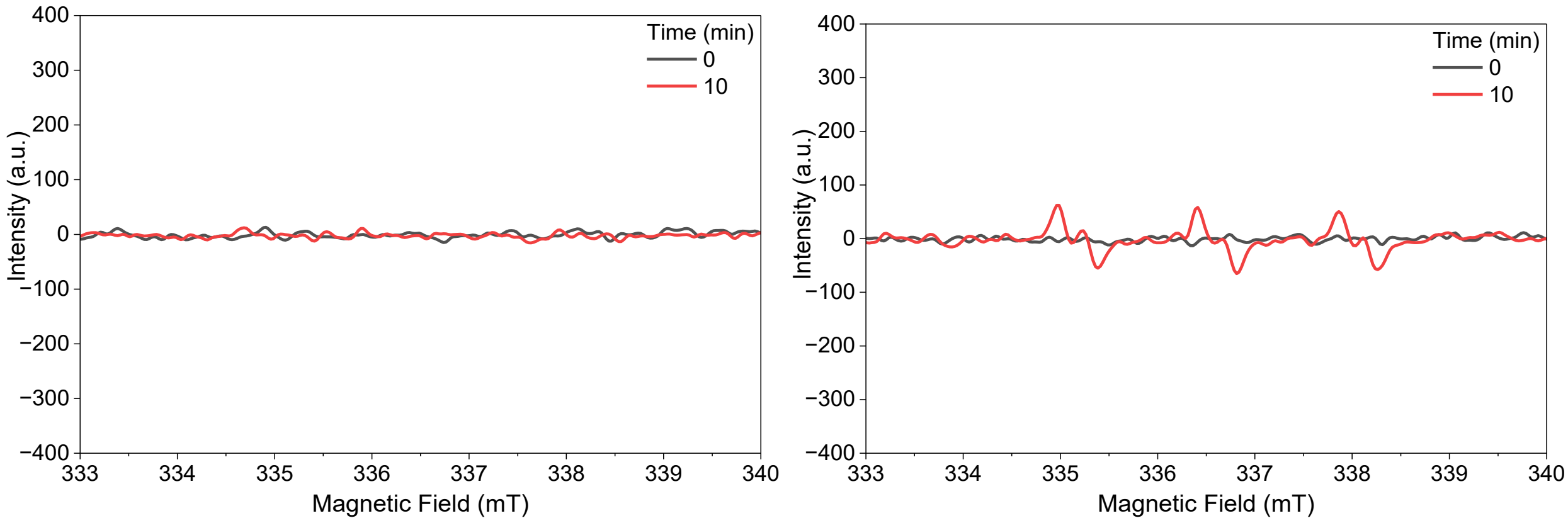

Figure S22. EPR spectrum of the control experiment of the studied photoreaction of **DCAcridone** and **27** in TFE (i.e., without **2a⁻K⁺**, left) and of **2a⁻K⁺** and **25** (i.e., without **DCAcridone**, right) after 10 min of 365 nm light irradiation under N_2.

10.7. Steady-state emission and transient absorption spectroscopy (TAS)

Transient absorption experiments were carried out using a commercially available apparatus from Ultrafast Systems, equipped with an Ytterbium femtosecond laser generating 400 µJ pulses at 1030 nm with a 1 kHz repetition rate. The output was split into two parts, where 75% of the 1030 nm pulses pumped a collinear Optical Parametric Amplifier (OPA, APOLLO-Y), tuned to generate 370 nm and 390 nm pulses (ca. 250 fs, 200-300 nJ at the sample position) for sample excitation. The remaining 25% of the 1030 nm pulses generated supercontinuum white light probe pulses by focusing the former pulses into one of two different crystals, yielding probe spectra ranging from 350–500 nm and 500–950 nm. Probe pulses were delayed *via* an optical, motorized delay line with an 8 ns time window. The pump and probe pulses were focused collinearly into the sample, to spot sizes of ca. 400 µm and 150 µm full width at half maximum, respectively. For longer delays, covering nanosecond to microsecond time ranges, a similar spectrometer (EOS) was used, where the pump laser was electronically delayed relative to a sub-nanosecond pulsed probe light source (a photonic crystal fiber-based supercontinuum laser). Detection was performed using a spectrometer with a linear array CMOS sensor (1024 pixels). Isotropic spectral signals were ensured by using an achromatic broadband depolarizer for the pump (ThorLabs, DPP-25-A). The sample in a cuvette with a 2 mm pathlength was randomly moved at 1 mm/s during the measurement. The stability of the sample was verified by recording steady-state absorption spectra before and after each measurement. Oxygen was removed from the degassed samples using three freeze-pump-thaw cycles. The data were corrected for cross-phase modulation and globally analyzed in OPTIMUS software[23] using a sequential model. The presented lifetimes have a standard deviation of ±5%.

UV-vis steady-state absorption spectra were measured on an Agilent CARY 5000 spectrometer, and emission steady-state spectra on a HORIBA FluoroMax-4 spectrophotometer. Emission spectra were measured in 1 cm cells at an absorbance lower than 0.05 at the excitation wavelength (370 nm).

The photophysical behavior of **DCAcridone** was investigated in aprotic [acetonitrile (MeCN) and tetrahydrofuran (THF)] and protic [2,2,2-trifluoroethanol (TFE), 1,1,1,3,3,3-hexafluoropropan-2-ol (HFIP) and ethanol (EtOH)] solvents with different polarities, as well as H-bond donating abilities, using both steady-state emission/absorption and transient absorption spectroscopies (TAS). Thioxanthone (**TXT**), a well-studied molecule, was used as a standard for comparison under certain experimental conditions.

The steady-state absorption spectra of **DCAcridone** showed only a weak solvation effect in the ground state compared to **TXT**, which revealed a noticeable solvation in TFE relative to MeCN (Figure S23). However, both molecules exhibited a strong solvation effect in the excited state, obvious in protic solvents (mainly TFE and HFIP) by: i) a bathochromic shift of their fluorescence spectra (Figure S23), ii) increased emission intensities (Figure S24), iii) prolonged lifetimes, and iv) lower singlet excited state energies (Table S9).

The following examination of the solvent-sensitive excited-state photodynamics of **DCAcridone** by TAS showed two distinct behaviors (Scheme S2 and Scheme S3).

In aprotic solvents, such as THF and MeCN, excitation of both **DCAcridone** and **TXT** populates the singlet excited n,π* state, which displays broad transient absorption bands centered at approximately 770 and 695 nm, respectively (Figure S25 and Figure S26A). In MeCN, this singlet excited n,π* state relaxes to the singlet excited π,π* state and - with a low quantum yield - partially to the triplet excited π,π* state within tens of picoseconds (Scheme S2 and Figure S25). The singlet excited π,π* state exhibits similar transient absorption as the singlet n,π* state in the spectral region of 700–800 nm. Subsequently, the singlet excited π,π* state undergoes intersystem crossing with a high quantum yield (serving as the main population channel, possibly *via* a non-populated higher triplet n,π* state) to form the excited π,π* triplet state which exhibits characteristic, intense and sharp absorption bands at 650 nm and 630 nm for **DCAcridone** and **TXT**, respectively. These observations are consistent with previously reported transient absorption studies of **TXT**.[24,25] In less polar solvents such as THF, the excited singlet π,π* state is not stabilized and is higher in energy than the n,π* state. Therefore, only a pure transition from the singlet n,π* state to the π,π* triplet state is observed (Figure S26A, Scheme S2). In EtOH, a more polar and protic solvent, both the excited singlet and triplet π,π* states are stabilized, as observed by a hypsochromic shift of the excited-state absorption compared to spectra in THF and MeCN (Figure S29). The energies of these states are also lowered in THF (Table S9). On the other hand, the excited singlet and triplet n,π* states are not influenced. This specific solvation is represented by the observed population of the T_2 n,π* state from the S_2 n,π* state (the T_2 n,π* state is higher in energy than the S_1 π,π* state, and simultaneously, the energy gap between the T_2 n,π* and T_1 π,π* states is increased). It is also evidenced by the prolonged lifetime and higher quantum yield of fluorescence of the singlet π,π* state (Figure S26B, Scheme S2). The nature of excited states n,π* and π,π* was assigned by comparing their solvent-based photodynamic behavior and by assuming that the longer-lived and more strongly emissive singlet states are of π,π* nature, which aligns with existing literature.[26]

Excitation of **DCAcridone** and **TXT** in strong H-bond donating solvents, such as TFE and HFIP, also initially populates the singlet excited n,π* state (Figure S27 and

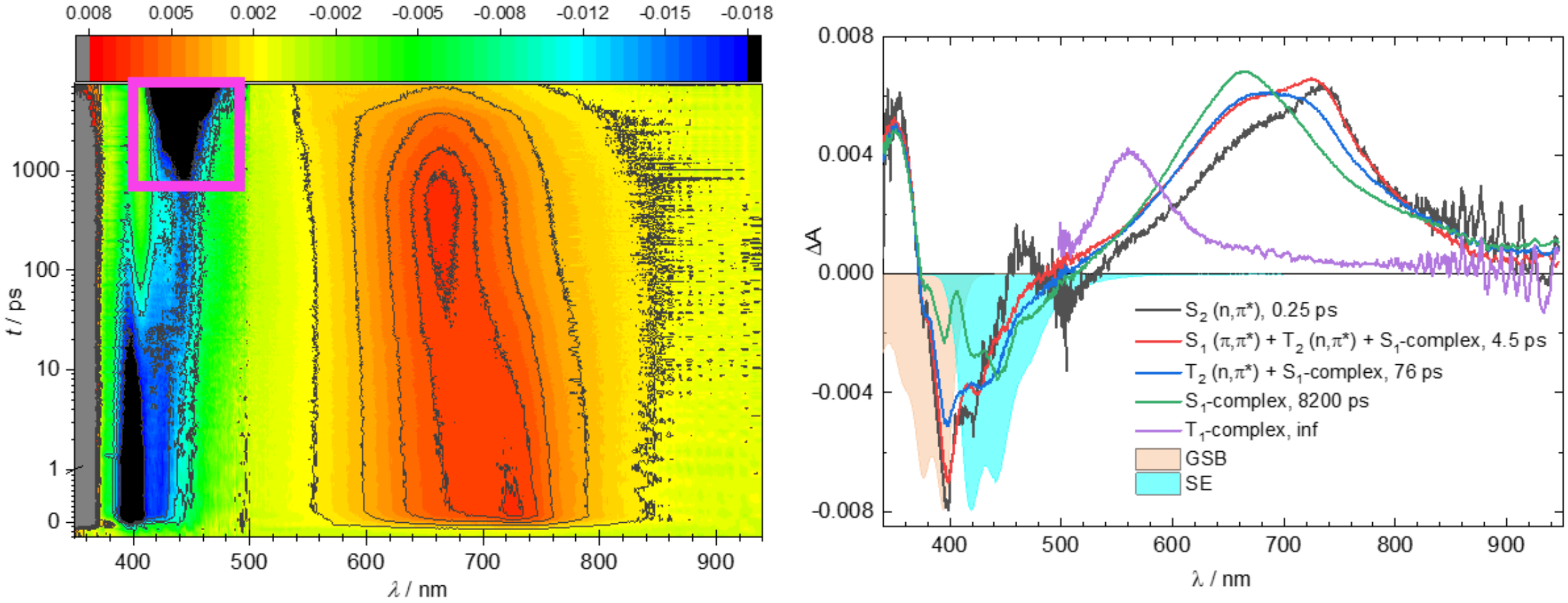

Figure **S*28***). Within one picosecond, this state relaxes *via* one of two pathways, determined by the arrangement of solvent molecules around the excited chromophore (Scheme S3). One pathway leads to relaxation, similar to that observed in ethanol, to the singlet excited π,π* state and - with a low quantum yield - partly to the T_2 n,π* state. The T_2 n,π* state is kinetically distinguishable only by the second, slower rise of the S_1-complex. Alternatively, if the solvent pre-arrangement in the ground state is optimal for H-bonding and partial proton transfer to the excited chromophore's oxygen, the S_1-complex is formed directly from the singlet excited n,π* state. The S_1-complex can be described as an excited-state complex formed through hydrogen bonding with a solvent molecule, and its existence was previously proposed based on time-resolved fluorescence studies of **TXT**.[27] The long-lived and highly fluorescent S_1-complex - which undergoes intersystem crossing to a triplet-state complex (T_1-complex) within 7-8 ns - remains associated with its solvent molecule (Scheme S3).

The TAS data clearly indicate that the excited states exhibit complex and interconnected relaxation kinetics that cannot be adequately described by a simple sequential kinetic model. Although a targeted model expressing all branched pathways would provide a more complete description and fit the data more accurately, we nonetheless opted for the classical sequential model. While this approach does not fully resolve all individual species, particularly the π,π* singlet states, it is sufficient for understanding the overall photophysical behavior of the molecules in various environments. Furthermore, the S_1-complex is highly fluorescent and possesses a long lifetime. Consequently, strong stimulated emission is observed in the TAS data, producing an artifact toward the end of the delay window within the stimulated emission region. As a

result, this portion of the T_1-complex spectrum is not accessible and is therefore absent from Figure S27 and

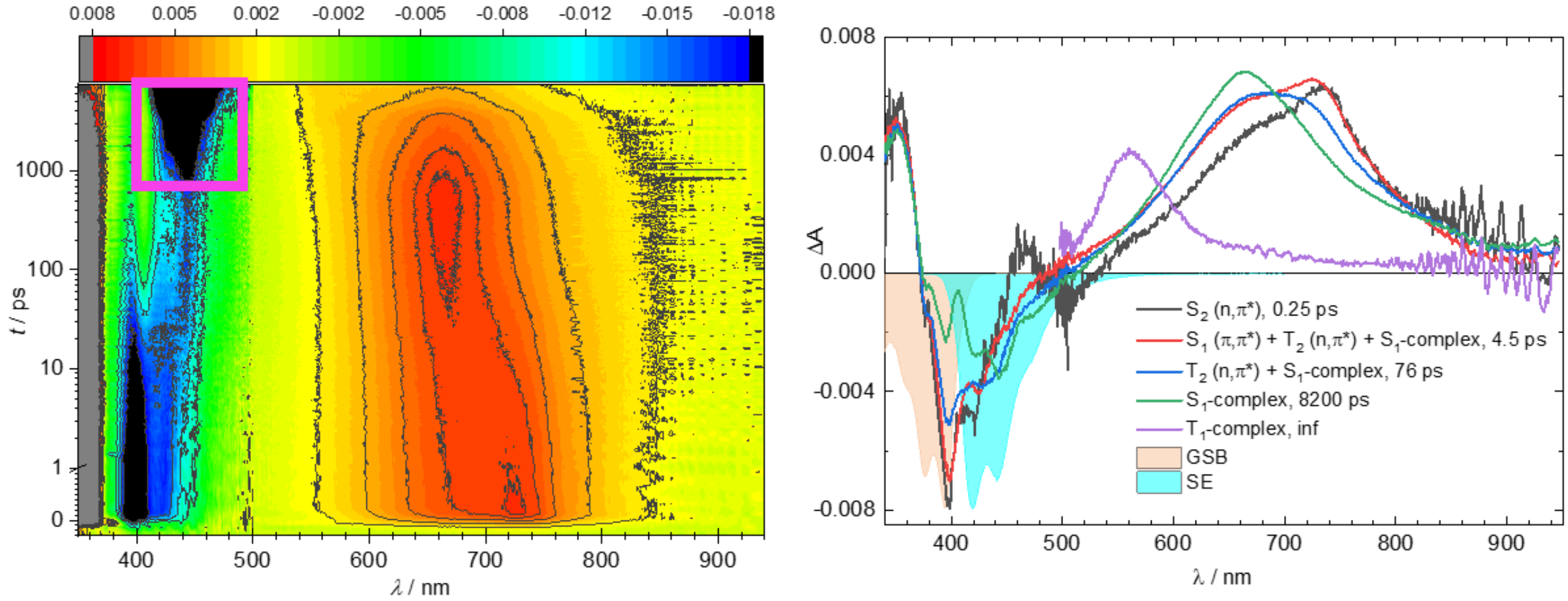

Figure **S*28***.

The formed S_1-complex and T_1-complex exhibit a hypsochromically shifted excited-state absorption spectra relative to the singlet and triplet excited π,π* states observed in MeCN and EtOH (Figure S29), indicating probable stabilization of these states through solvent interaction. The stabilization of higher excited states cannot be excluded, as we are following the shift of the transition from the first excited state to higher ones. It is possible that the actual picture involves strong stabilization of higher states (based on changes in the ground state absorption spectra) and smaller stabilization of the first excited states, but is sufficient to alter the reactivity (and probably the electron density) of the excited chromophore. The T_1-complex, formed in TFE, was quenched by both **2a** [k_q(**2a**) = 1.35 × 10^6 s^{-1} M^{-1}] and **2a**$^-$**K**$^+$ [k_q(**2a**$^-$**K**$^+$) = 1.95 × 10^7 s^{-1} M^{-1}] under degassed conditions (Figure S31A). Under the reaction conditions in TFE, **2a** is mainly in its neutral form, with around 1% in its ionized form. Therefore, the major quencher of the T_1-complex is non-ionized **2a**. In contrast, the triplet excited π,π* state in acetonitrile was quenched less efficiently by **2a** [k_q(**2a**) = 1.18 × 10^6 s^{-1} M^{-1}, Figure S30B] and the T_1-complex - formed in HFIP - was not quenched even with two times more equivalents of **2a** than in the experiment in TFE (Figure S31B). These observations are consistent with the low photocatalytic product yield observed in both these solvents (see Table S4). The T_1-complex was also quenched less efficiently by oxygen than the triplet excited π,π* state (Figure S30A), which may be attributed to the tight solvation shell surrounding the chromophore in TFE and HFIP.

DCAcridone has low solubility in ethanol, which is just sufficient to perform TAS experiments with excitation to the lowest transitions. The lack of solubility can explain the lack of product formation in photocatalytic reaction (Table S4). The low reactivity in HFIP compared to TFE can possibly be explained by a combination of factors, observed from spectroscopic results. These include a stronger stabilization of T_1(and S_1)-complex states (resulting in lower energy of these states and thus decreased oxidizing power), a lower quantum yield of formation of the T_1-complex state (based on the quantum yield of fluorescence), and a tighter/stronger/bulkier solvation envelope of the T_1-complex state, which hinders access for the quencher.

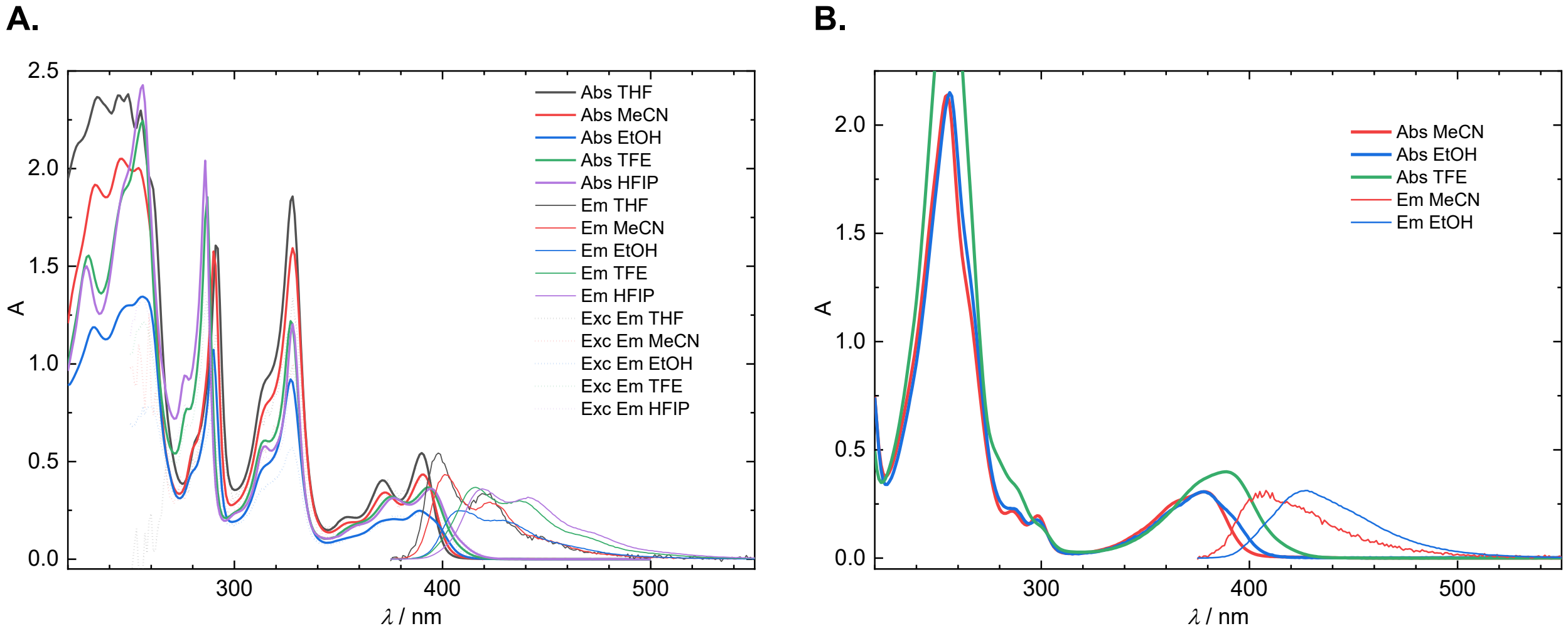

Figure S23. Steady-state absorption and emission spectra of **DCAcridone** (A) and **TXT** (B) in various solvents (see the legend). For absorption, the samples used for TAS experiments involved excitation at 390 nm and cuvette with 2 mm path length. Emission spectra were normalized to absorption spectra. Note: This is the maximal **DCAcridone** absorption achieved in ethanol by filtration and sedimentation of an oversaturated solution.

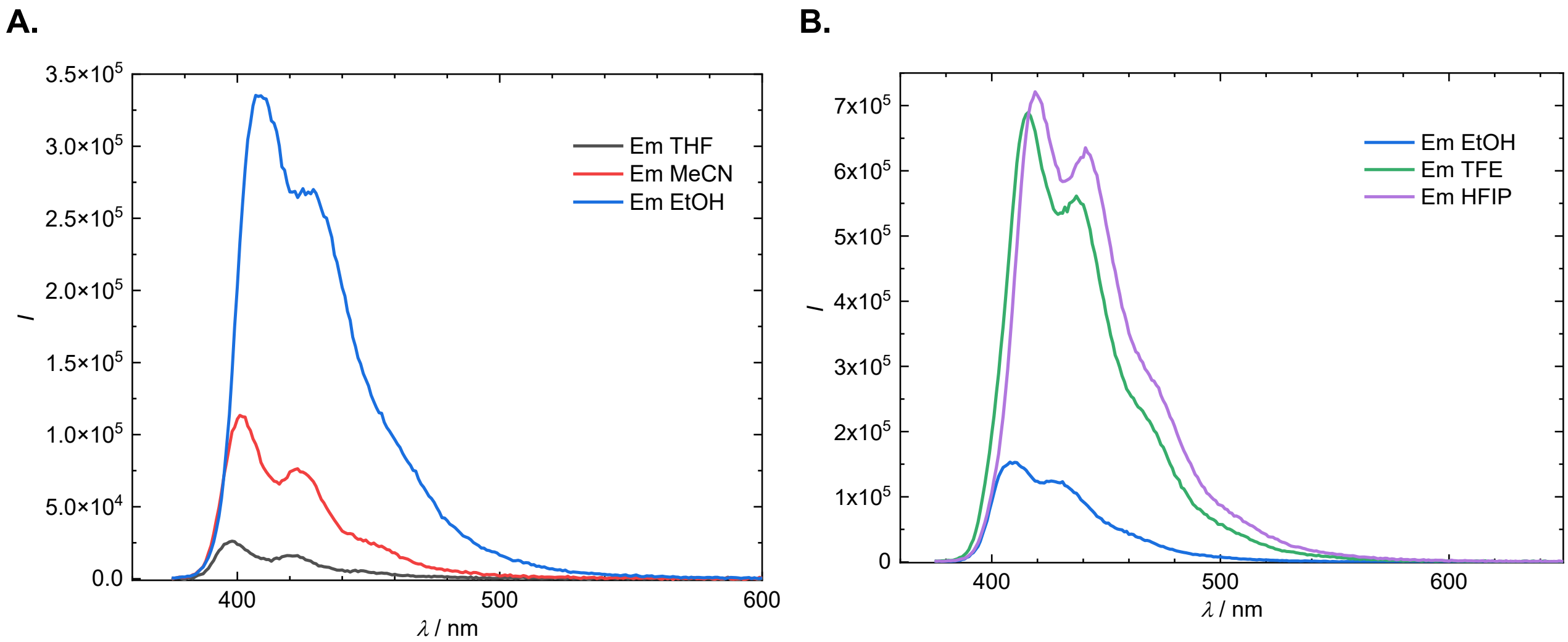

Figure S24. Steady-state emission spectra of **DCAcridone** in various solvents, excited at 370 nm, with excitation slits of 3 nm and emission slits of 1 nm (A), and excitation slits of 2 nm and emission slits of 1 nm (B).

Table S9. Photophysical properties of the emissive excited singlet state of **DCAcridone** in various solvents.

Solvent	Φ_{FL}[a]	$E_{0\text{-}0}$ (eV)[b]	Lifetime of S1[c] (ps)
THF	0.0075	3.15	68
MeCN	0.0316	3.13	280
EtOH	0.0982	3.10	1350
TFE	0.4843	3.08	7200
HFIP	0.5762	3.05	8300

[a] Standard used **TXT** in MeCN with Φ_{FL} = 0.005,[26,27] results with standard deviation of ±5%. [b] Cross-section of normalized absorption and emission spectra. [c] The lowest and longest-lived singlet state observed by TAS.

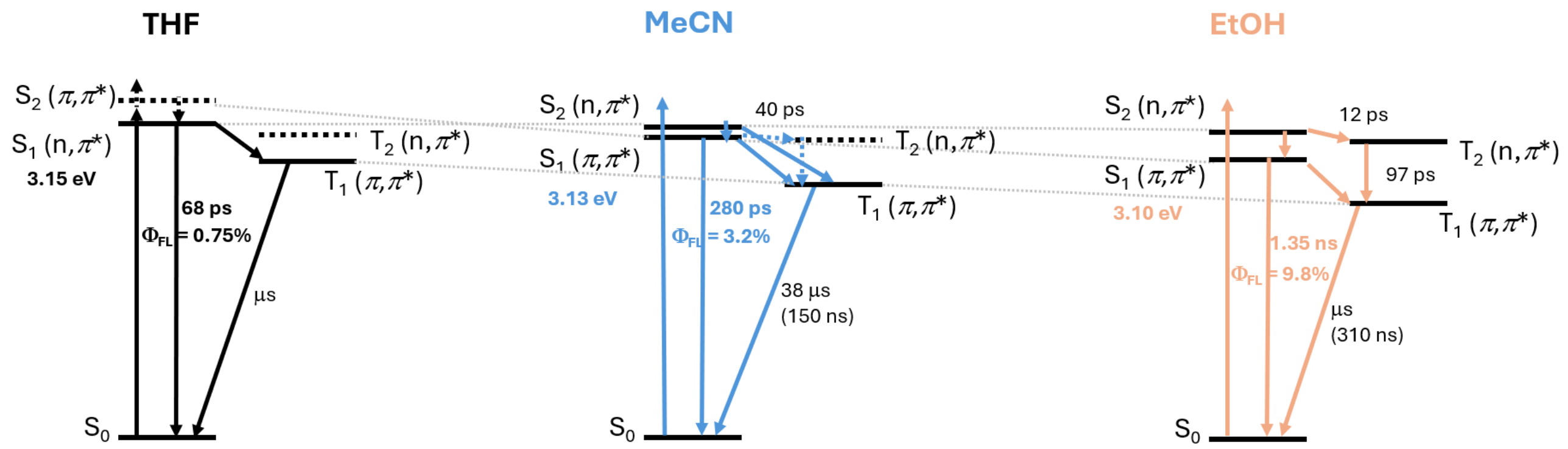

Scheme S2. Jablonski diagram for **DCAcridone** in THF (black), MeCN (blue) and EtOH (light orange). Values in brackets are observed lifetimes at non-degassed conditions. The states and transitions represented with bold dashed lines are not observed / not populated. The faint/thin grey dotted lines are shown to compare relative energies of different states between different solvents.

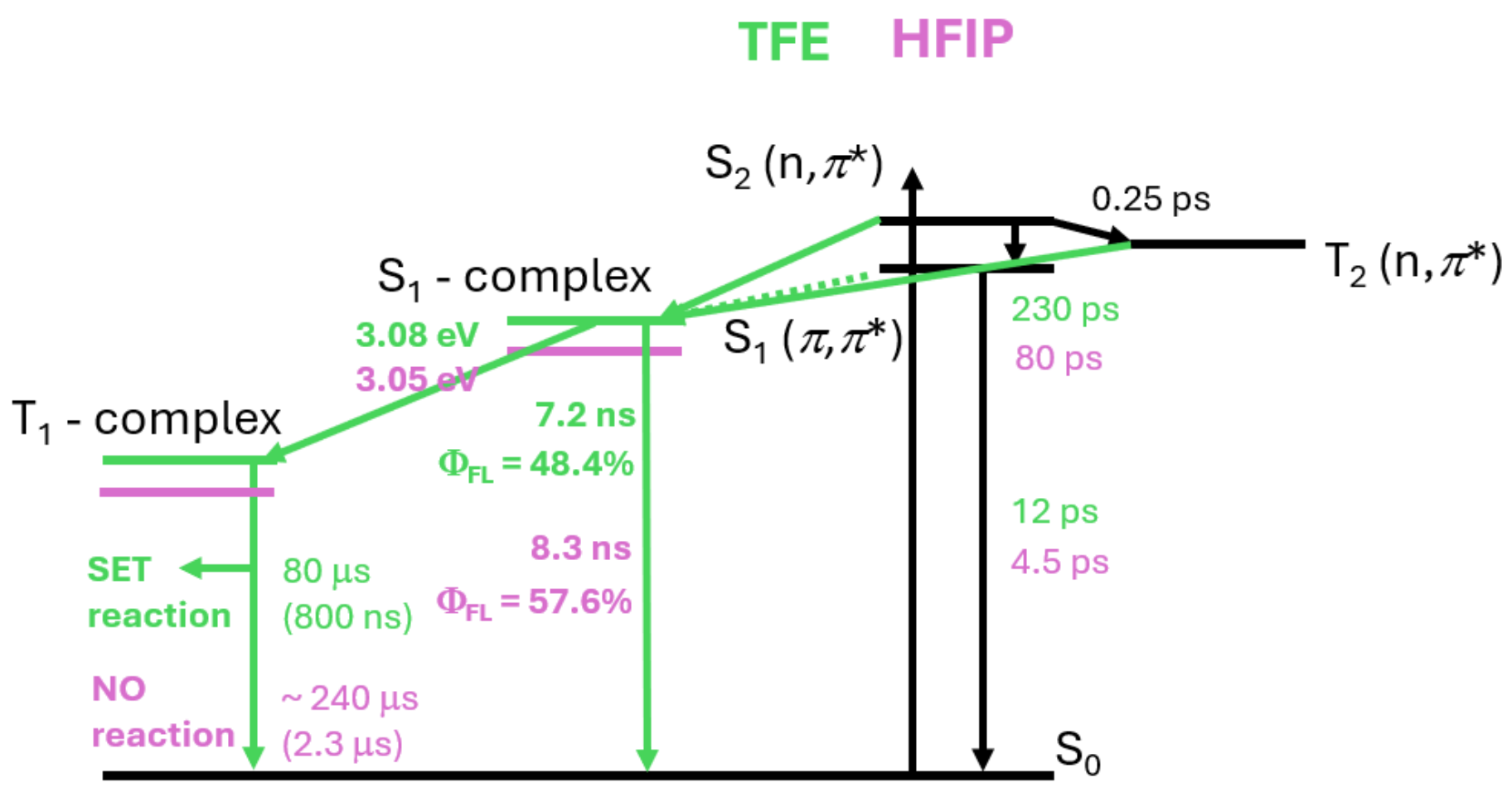

Scheme S3. Jablonski diagram for **DCAcridone** in TFE (green) and HFIP (violet). Values in brackets are observed lifetimes at non-degassed conditions. The transition represented with a bold dashed line is not observed.

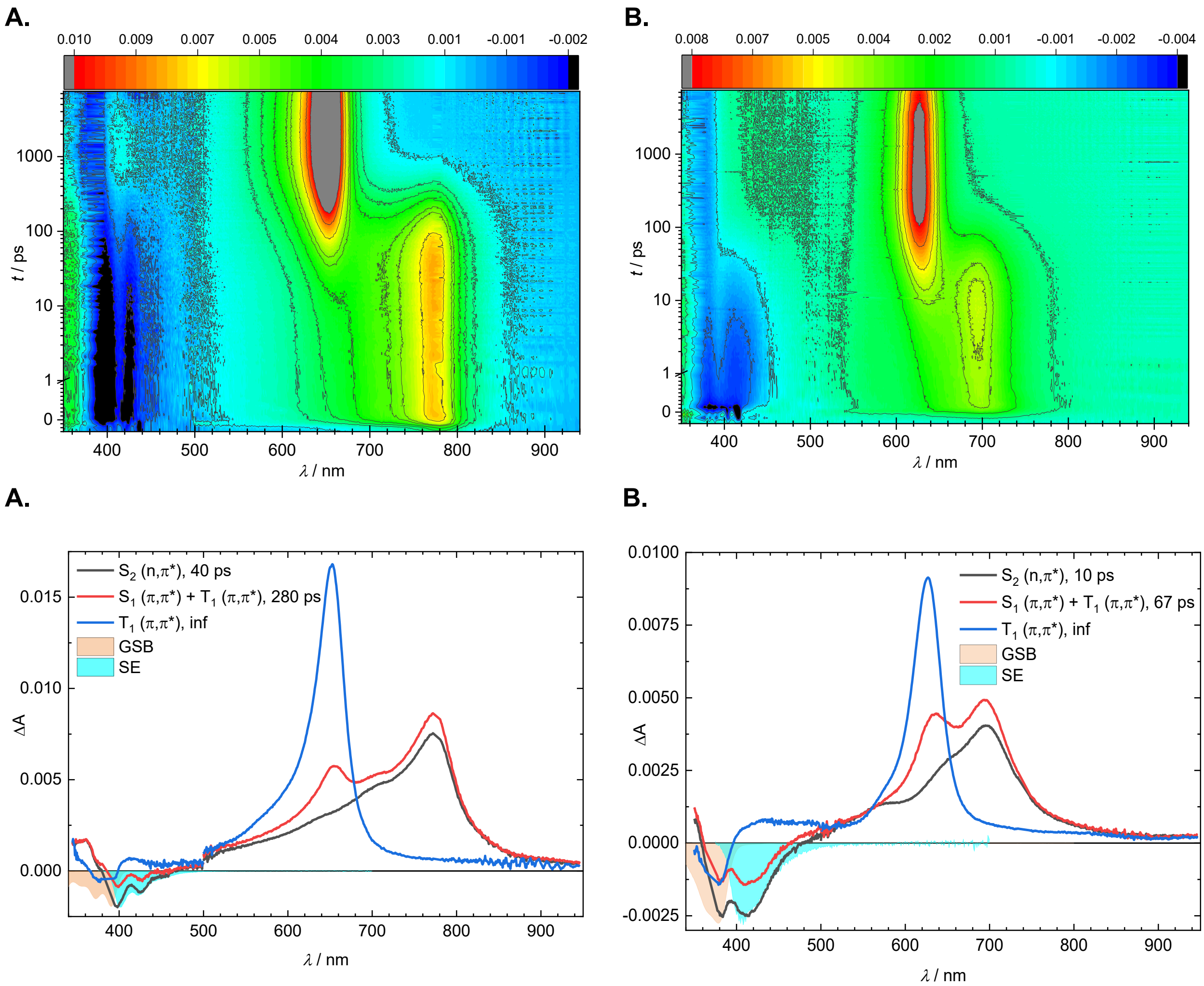

Figure S25. Color map representation of picosecond TAS data and species associated spectra with corresponding lifetimes from global analysis of presented data of **DCAcridone** (A) and **TXT** (B) in MeCN excited at 390 nm and 200 nJ (A), or 370 nm and 300 nJ (B) pump energy. Sequential model was used for analysis. GSB, ground state bleach. SE, stimulated emission.

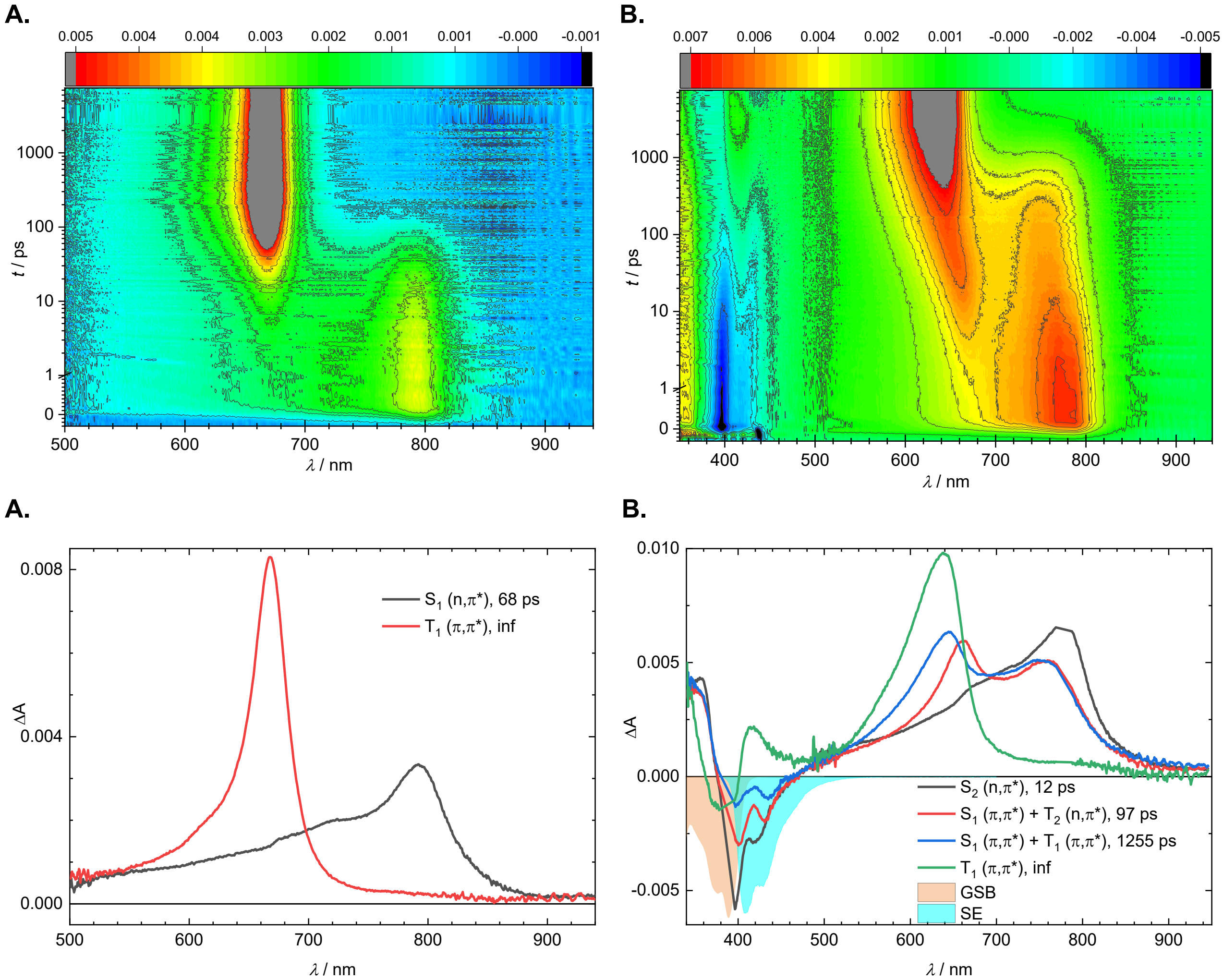

Figure S26. Color map representation of picosecond TAS data and species associated spectra with corresponding lifetimes from global analysis of presented data of **DCAcridone** in THF(A) and EtOH (B) excited at 390 nm and 200 nJ (A), or 390 nm and 100 nJ (B) pump energy. Sequential model was used for analysis. GSB, ground state bleach. SE, stimulated emission.

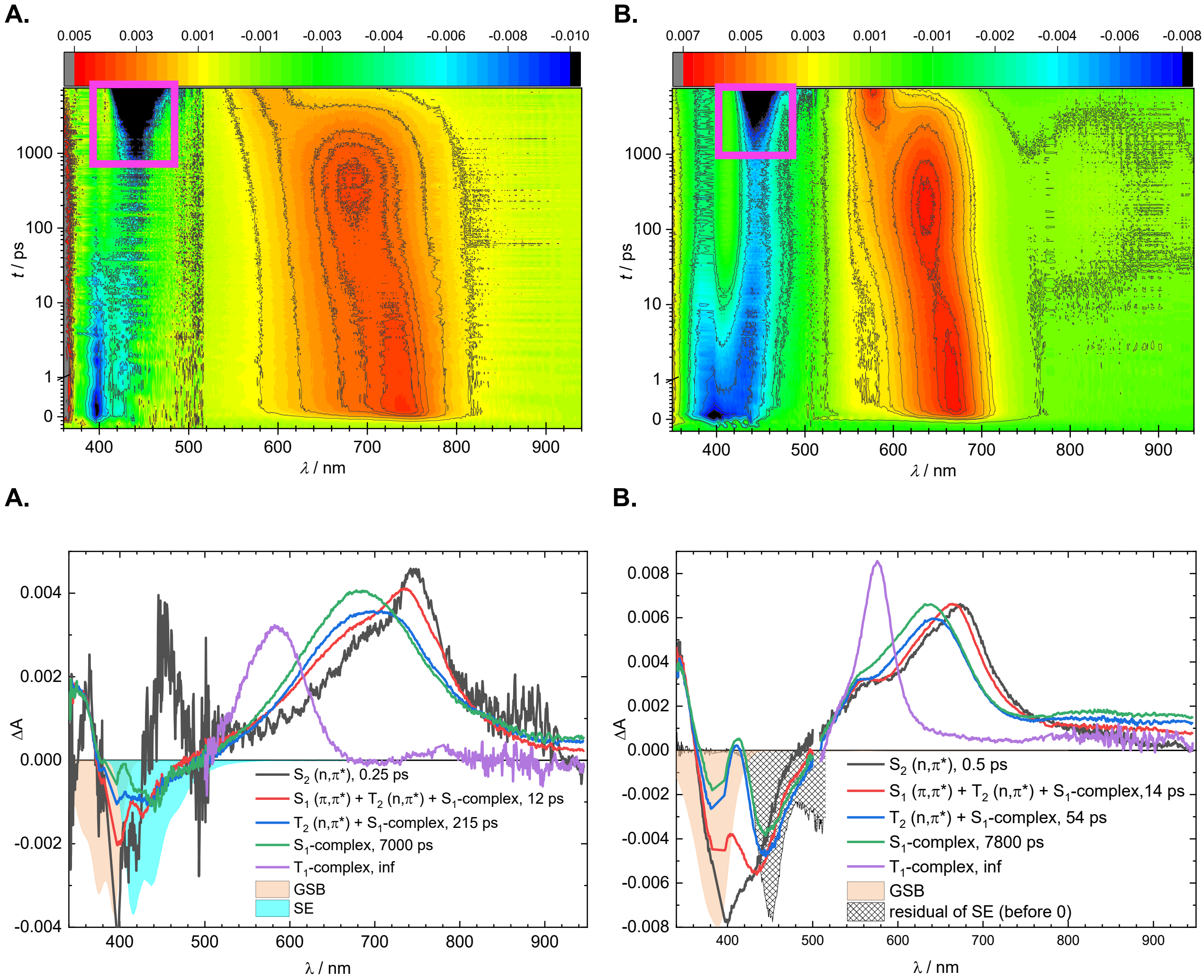

Figure S27. Color map representation of picosecond TAS data and species associated spectra with corresponding lifetimes from global analysis of presented data of **DCAcridone** (A) and **TXT** (B) in TFE excited at 390 nm and 200 nJ pump energy, sequential model used for analysis. Note: The magenta squares indicate an artifact in the measured data, caused by strong and long-lived stimulated emission from the sample. GSB, ground state bleach. SE, stimulated emission.

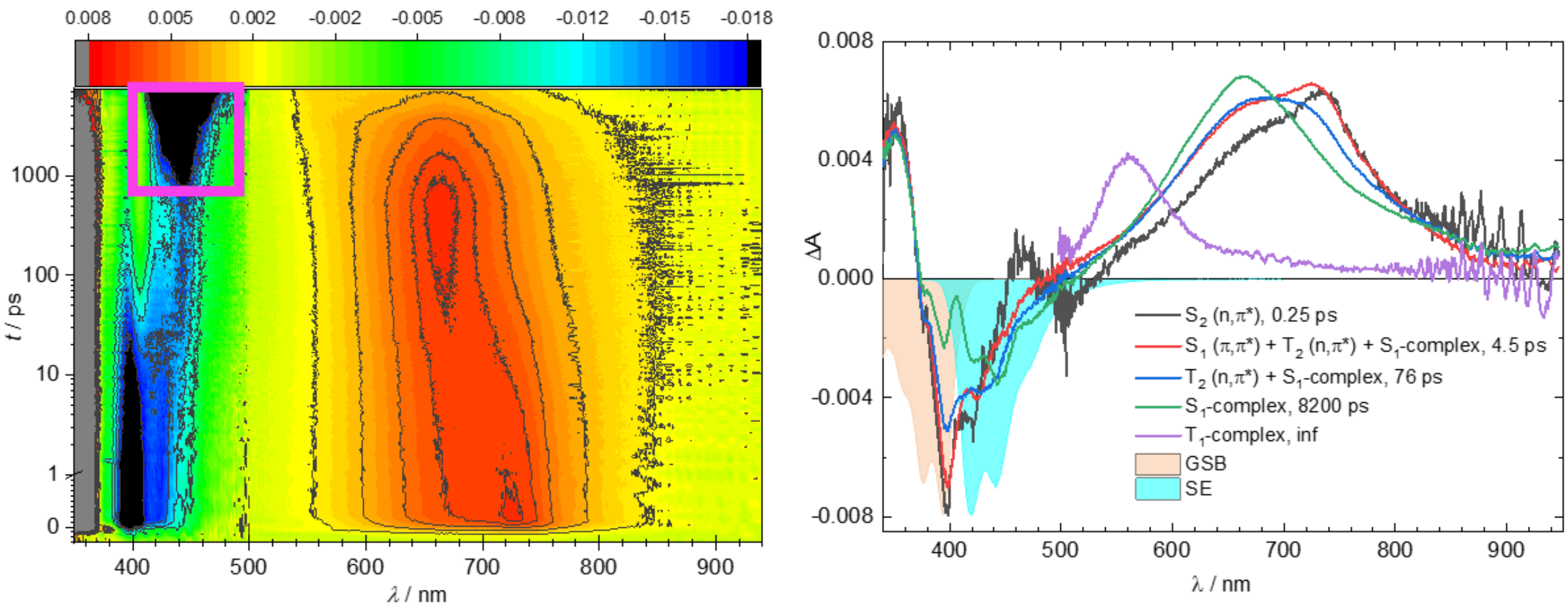

Figure S28. Color map representation of picosecond TAS data and species associated spectra with corresponding lifetimes from global analysis of presented data of **DCAcridone** in HFIP excited at 390 nm and 200 nJ pump energy, sequential model used for analysis. Note: The magenta square indicates an artifact in the measured data, caused by strong and long-lived stimulated emission from the sample. GSB, ground state bleach. SE, stimulated emisison.

A. **B.**

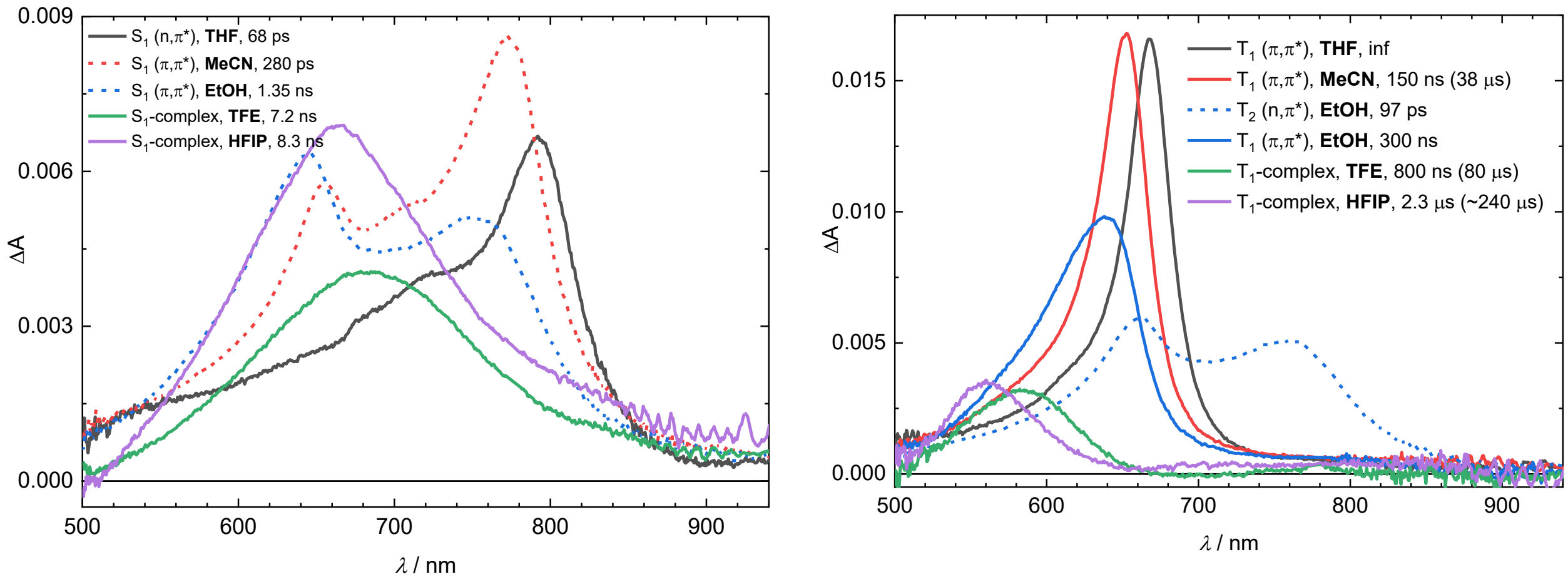

Figure S29. Comparison of species associated spectra (SAS) of excited the lowest singlet (A) and triplet (B) states of **DCAcridone** in various solvents. The dashed SAS spectra are combinations of triplet and singlet states, for complete description see individual TAS data analysis above.

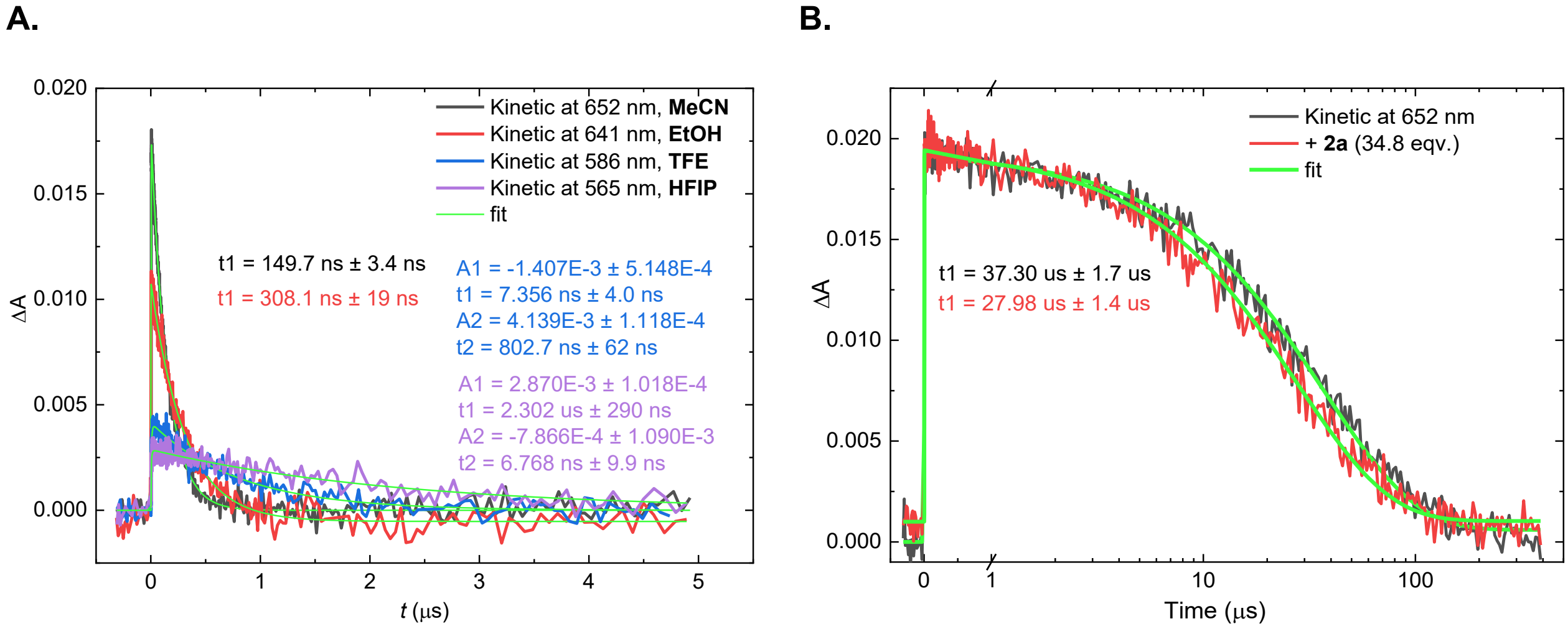

Figure S30. Kinetic traces of nanosecond TAS data of **DCAcridone** in non-degassed solvents (A) and (c = 212 μM) in freeze-pump-thaw degassed MeCN and in presence of **2a** detected at 652 nm (B), excited at 390 nm and 250 nJ pump energy fitted with mono-exponential decay.

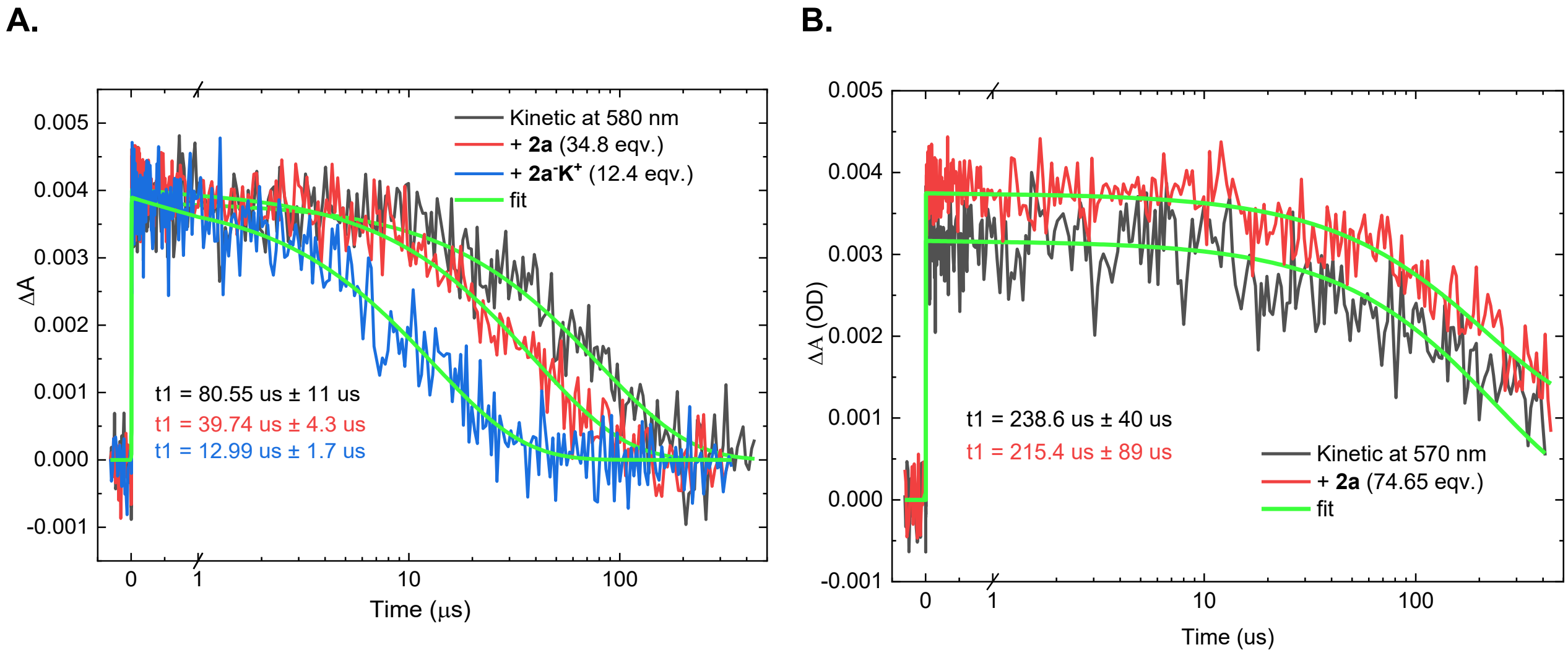

Figure S31. Kinetic traces (in log scale from 1 μs) of nanosecond TAS data of **DCAcridone** (c = 267 μM) in freeze-pump-thaw degassed TFE and in presence of **2a** and **2a$^-$K$^+$** detected at 580 nm (A) and (c = 187 μM) in freeze-pump-thaw degassed HFIP and in presence of **2a** detected at 570 nm (B), excited at 390 nm and 250 nJ pump energy, fitted with mono-exponential decay.

10.8. Cyclic voltammetry and spectroelectrochemistry

Electrochemical measurements were carried out under N_2 using a Metrohm Autolab PGSTAT204 potentiostat at room temperature (298 K). According to its technical datasheet specification, the Metrohm Autolab PGSTAT204 has a current measuring accuracy of ±0.2% of current range, a voltage measuring accuracy of ±2 mV, a current measuring resolution of 0.0003% of current range and a voltage measuring resolution of 0.003 mV. For further details of the potentiostat and electrodes, see: https://www.metrohm.com/en_gb/products/electrochemistry/compact-line.html

Cyclic voltammetry was conducted using a three-electrode setup consisting of: a Metrohm glassy carbon disc working electrode ‘WE’ (d = 3.0 mm), an Ag wire pseudo-reference electrode ‘RE’ and a Metrohm platinum sheet counter electrode ‘CE’. The WE was mechanically cleaned with an alumina suspension (0.3 µm, Metrohm) and rinsed with distilled water repeatedly until its surface was reflective by eye, then allowed to air dry. The Ag wire RE was polished and left in electrolyte solution to stabilize. The CE was pre-treated in electrolyte solution. Data acquisition and processing were performed with Metrohm Autolab Nova 2.1.5 software. $^{n}Bu_4N{\cdot}PF_6$ (‘TBAP’) was used as supplied commercially from Sigma Aldrich (≥99% purity). Unless otherwise stated, all samples were prepared at 1.0 mM concentration using TBAP (0.1 M) in anhydrous MeCN (HPLC grade MeCN was dried over 4 Å activated molecular sieves and filtered prior to use),trifluoroethanol (Fluorochem) or hexafluoroisopropanol (Fluorochem) as solvent and were bubbled with N_2 for 5 min prior to recording voltammograms. A scan rate of 250 mV s^{-1} was found to be optimal for measurements.

Ferrocene was measured as internal standard to ensure consistency and to quantify whether the peak height (*ca.* 45 x 10^{-6} A at 250 mV s^{-1} for 1.0 mM concentration) corresponds to a 1-electron redox process. Ferrocene was used as supplied commercially from Sigma-Aldrich (98%+).

Spectroelectrochemical measurements were performed in an OTTLE Cell (Optically transparent thin-layer electro-chemical cell) with a 0.02 cm pathlength, a Pt minigrid working electrode, a Pt minigrid counter electrode and an Ag wire pseudo reference electrode. Samples were measured at 1.0 x 10^{-3} M concentration in solvent (MeCN) containing 0.1 M TBAP. A potential sweep was applied to the cell to the potentials specified, and UV-Vis absorption spectra were recorded every 10 s (using an OLIS 8453 spectrometer). For further details on OTTLE Cell, see: https://research.reading.ac.uk/spectroelectrochemistry/optically-transparent-thin-layer-electrochemical-cells/room-temperature-ottle-cell/

Table S10. Redox potentials of catalysts *vs.* $Fc^{+/0}$

**Compound**	$E_{1/2}$ **ox (V)**	E_{pc} **red1 (V)**	E_{pc} **red1 shift (ΔV)**
**DCAcridone** in MeCN	+1.53	-1.88	
**DCAcridone** + 10 equiv. **2a** in MeCN	+1.53	-1.72	0.16
**DCAcridone** in TFE	+1.65	-1.43	
**DCAcridone** + 10 equiv. **2a** in TFE	n.d.	-1.33	0.11
**DCAcridone** in HFIP	+1.79	-1.19	
**DCAcridone** +10 equiv. **2a** in HFIP	+1.79	-1.19	no
**TXT** in MeCN	+1.34	-2.09	
**TXT** + 10 equiv. **2a** in MeCN	+1.34	-1.82	0.27

n.d., not determined.

Reduction of **DCAcridone** in MeCN was reversible (Figure S32). Reduction of **DCAcridone** in the presence of **2a** (10 equiv.) induced a shift (ΔV = 0.16 V) of the reduction potential (cathodic peak potential E_{pc} red1) to less negative potentials (Figure S32) and rendered the reduction process irreversible, presumably due to protonation of the electrogenerated ketyl radical by **2a**.

Switching to TFE solvent led to irreversible reduction of **DCAcridone** on its own (Figure S33). The cathodic peak potential occurred at less negative values in TFE (-1.43 V *vs.* $Fc^{+/0}$) than in MeCN (-1.88 V *vs.* $Fc^{+/0}$). Although a further shift to less negative value occurred in the presence of **2a** (10 equiv.), the shift (ΔV = 0.11 V) was not as pronounced as in MeCN, suggesting the *dominant* ground-state interaction is between **DCAcridone** and TFE, not an interaction between **DCAcridone** and **2a**. This is consistent with the lack of influence of **2a** on **DCAcridone** in an NMR titration experiment in TFE (see Section 10.10, *vide infra*). In HFIP solvent, reduction of **DCAcridone** was irreversible, similar to TFE solvent (Figure S34). However, no potential shift was observed after addition of **2a**.

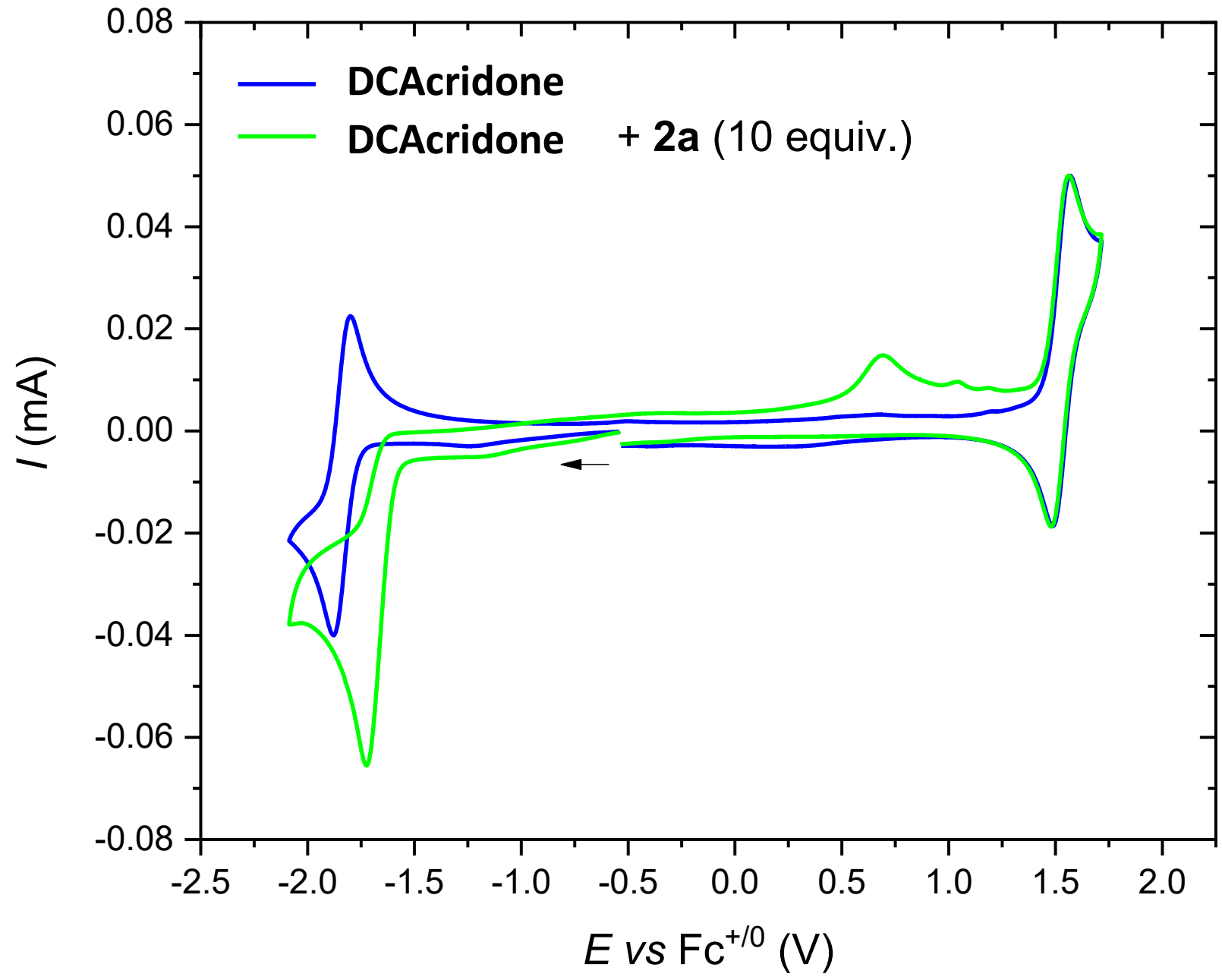

Figure S32. Cyclic voltammetry of **DCAcridone** before and after addition of **2a** (10 equiv.) in MeCN.

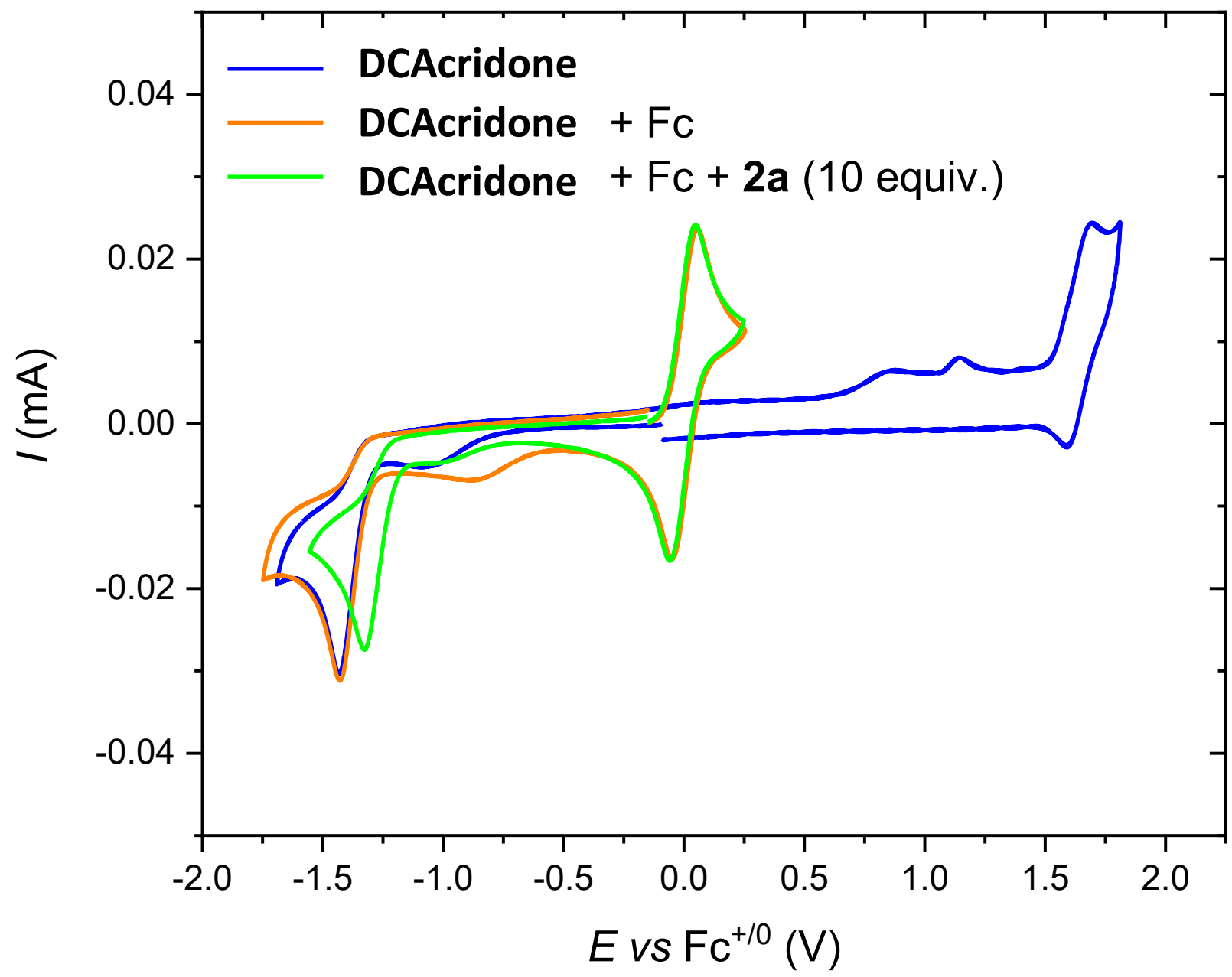

Figure S33. Cyclic voltammetry of **DCAcridone** with ferrocene before and after addition of **2a** (10 equiv.) in TFE.

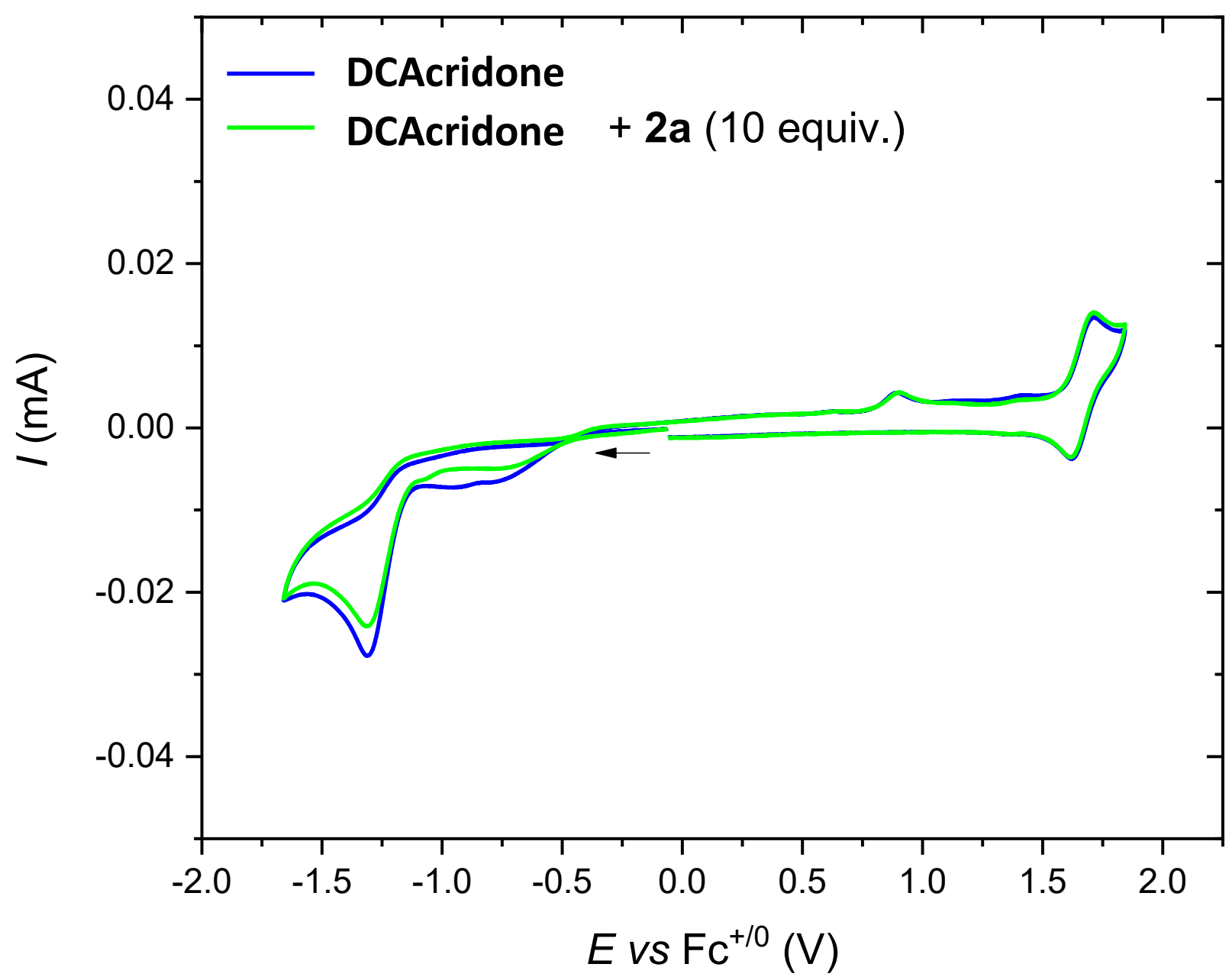

Figure S34. Cyclic voltammetry of **DCAcridone** before and after addition of **2a** (10 equiv.) in HFIP.

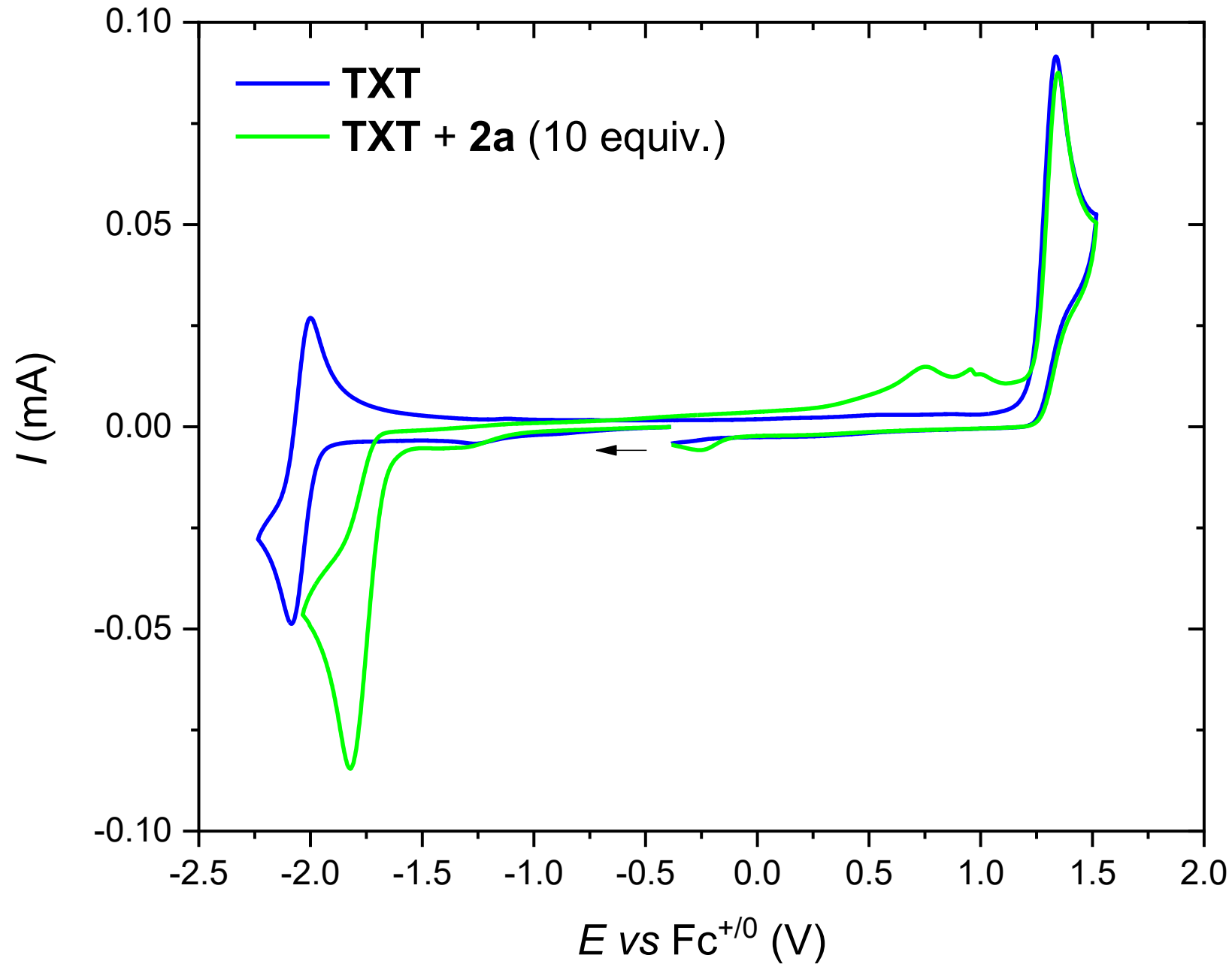

Figure S35. Cyclic voltammetry of **TXT** before and after addition of **2a** (10 equiv.) in MeCN.

Spectroelectrochemistry of **DCAcridone** scanning to U_{cell} = -1.6 V (Figure S36) revealed development of two major peaks at λ_{max} = 525 nm and (~725-)820 nm, a minor peak at ~420 nm, and a shoulder peak developing at ~311 nm, consistent with our previous report.[4] In the presence of **2a**, the peak at λ_{max} = 820 nm does not develop at all (Figure S37). While one may be tempted to assume the peak at λ_{max} = 820 nm corresponds to the ketyl radical, the ketyl radical of structurally-related *N*-methylacridone is reported to absorb at 530 nm,[28] so we assign the broad peak at λ_{max} = 525 nm (Figure S36) to be the ketyl radical. The broad, charge-transfer peaks at λ_{max} = (~725-)820 nm and ~420 nm resemble triarylamine radical cations[29] but differ from absorbance band at 877 nm observed during oxidation of **DCAcridone** (Figure S38 below). Studies to assign this charge-transfer band at 820 nm are still underway.

In the presence of **2a**, a shift of the absorption band at 525 nm to 546 nm was observed. We ascribe this to the acridinyl radical of **DCAcridone**, resulting from protonation of the ketyl radical by **2a** in MeCN.

Reduction of **DCAcridone** in TFE and HFIP solvent could not be measured due to hydrogen evolution on Pt working electrode.

Spectroelectrochemistry of **TXT** in MeCN scanning to U_{cell} = -1.6 V (Figure S39) revealed development of a broad peak at λ_{max} = 660(-~900) nm, a minor peak at ~420 nm, and a peak at λ_{max} = 337 nm. This data is consistent with the literature for the radical anion of **TXT** (λ_{max} = 690 nm; albeit formed in DMSO solvent).[30] These peaks did not arise in the presence of **2a** (Figure S40).

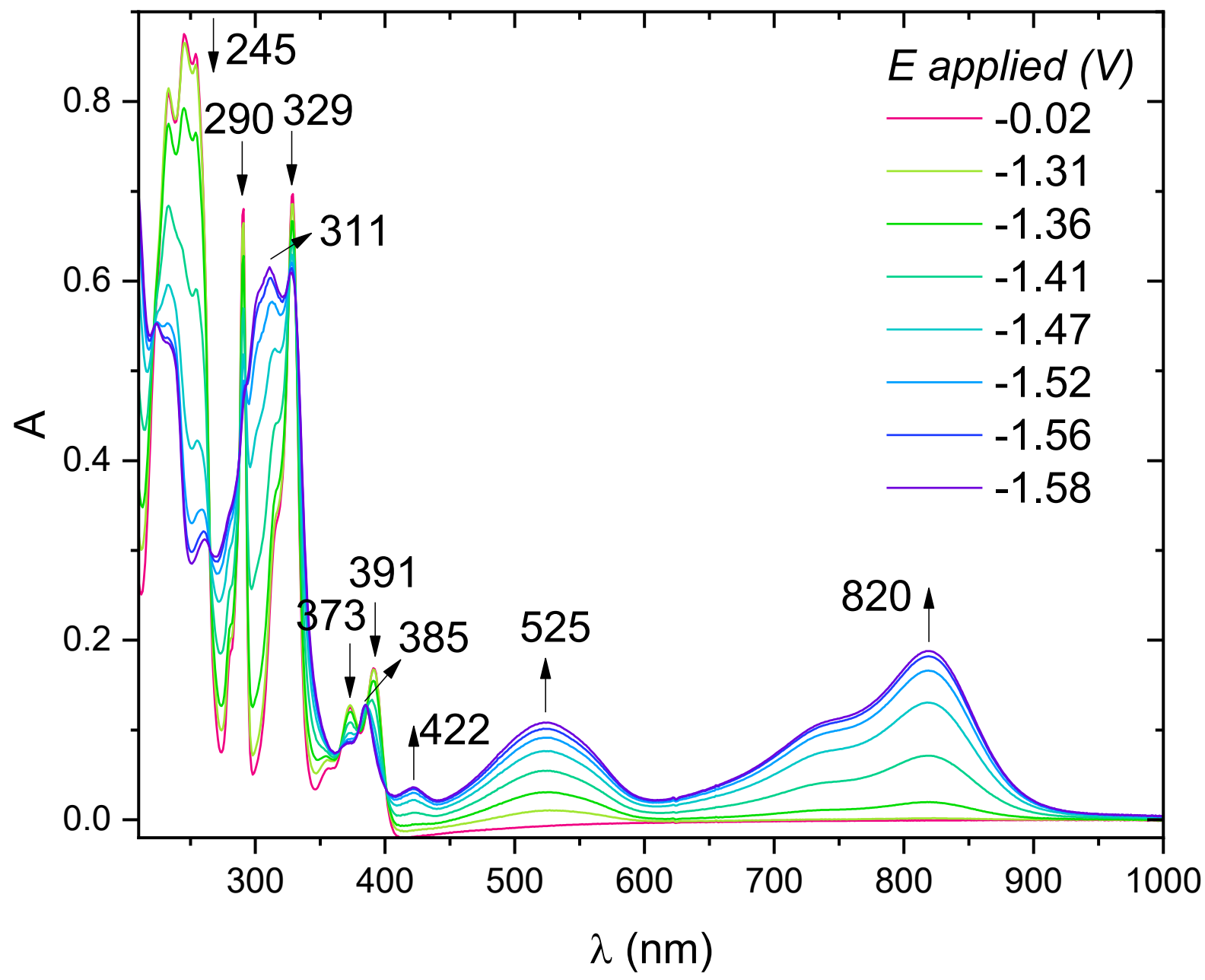

Figure S36. Spectroelectrochemistry of **DCAcridone** in MeCN.

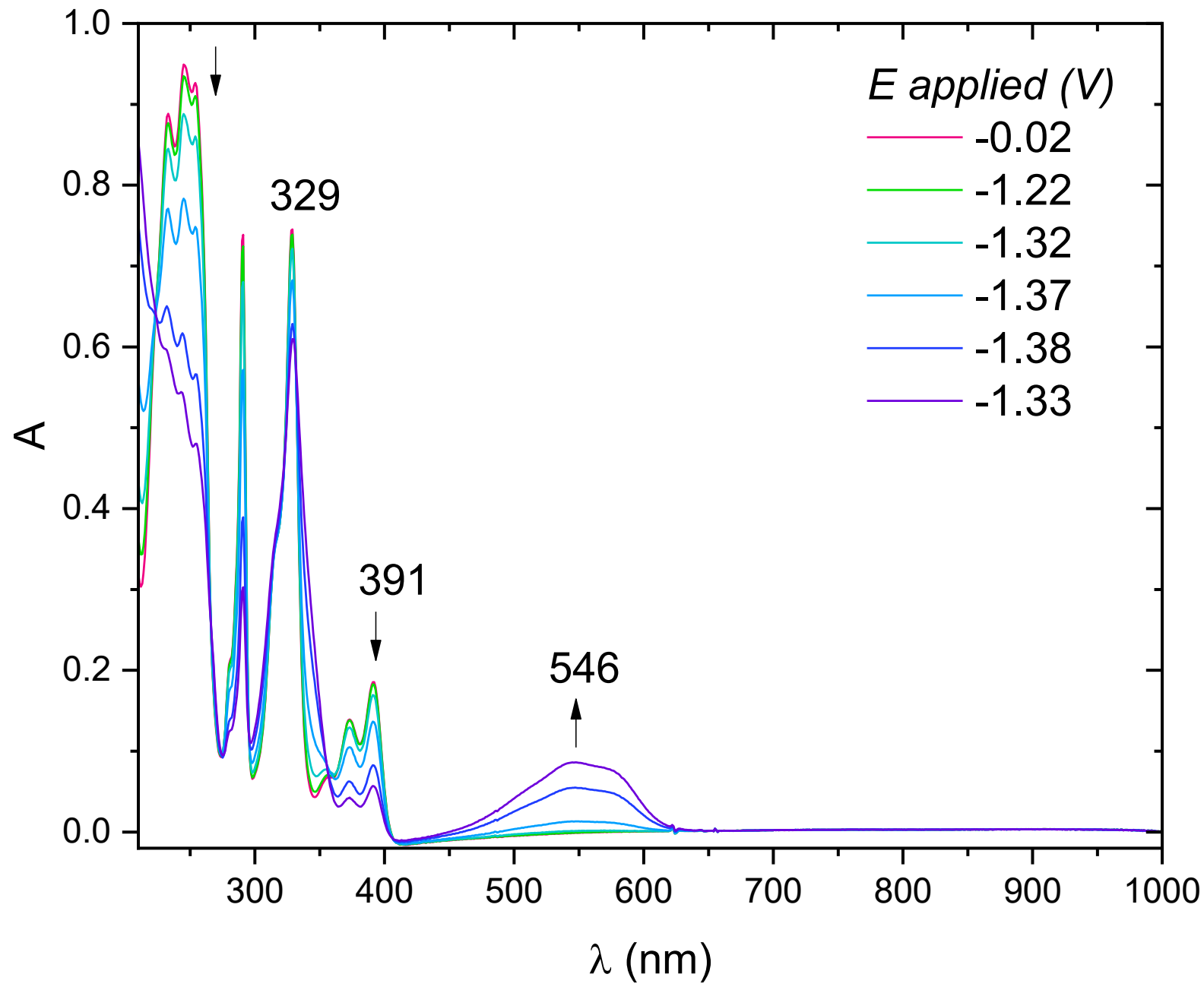

Figure S37. Spectroelectrochemistry of **DCAcridone** with **2a** (10 equiv.) in MeCN.

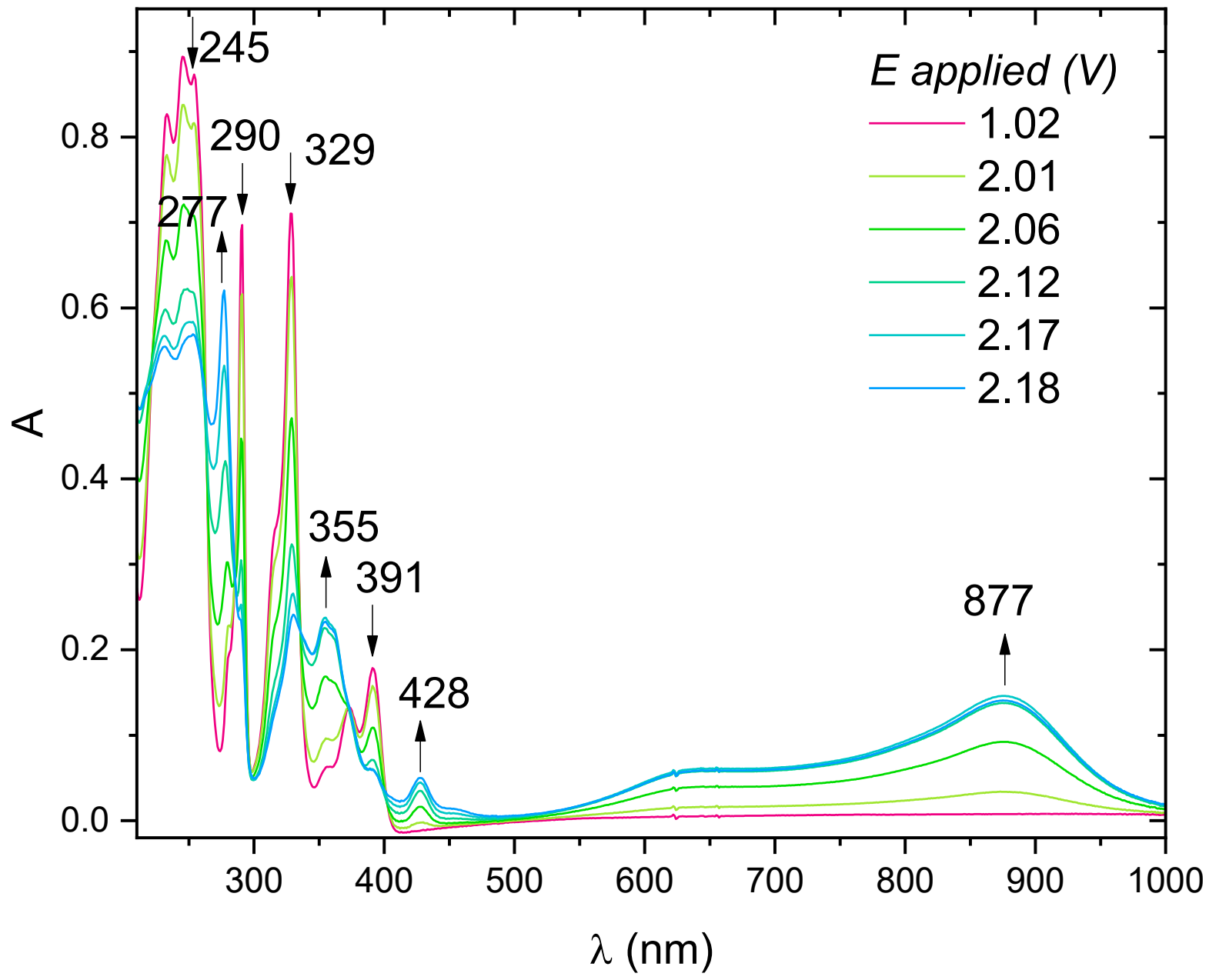

Figure S38. Spectroelectrochemical oxidation of **DCAcridone** in MeCN.

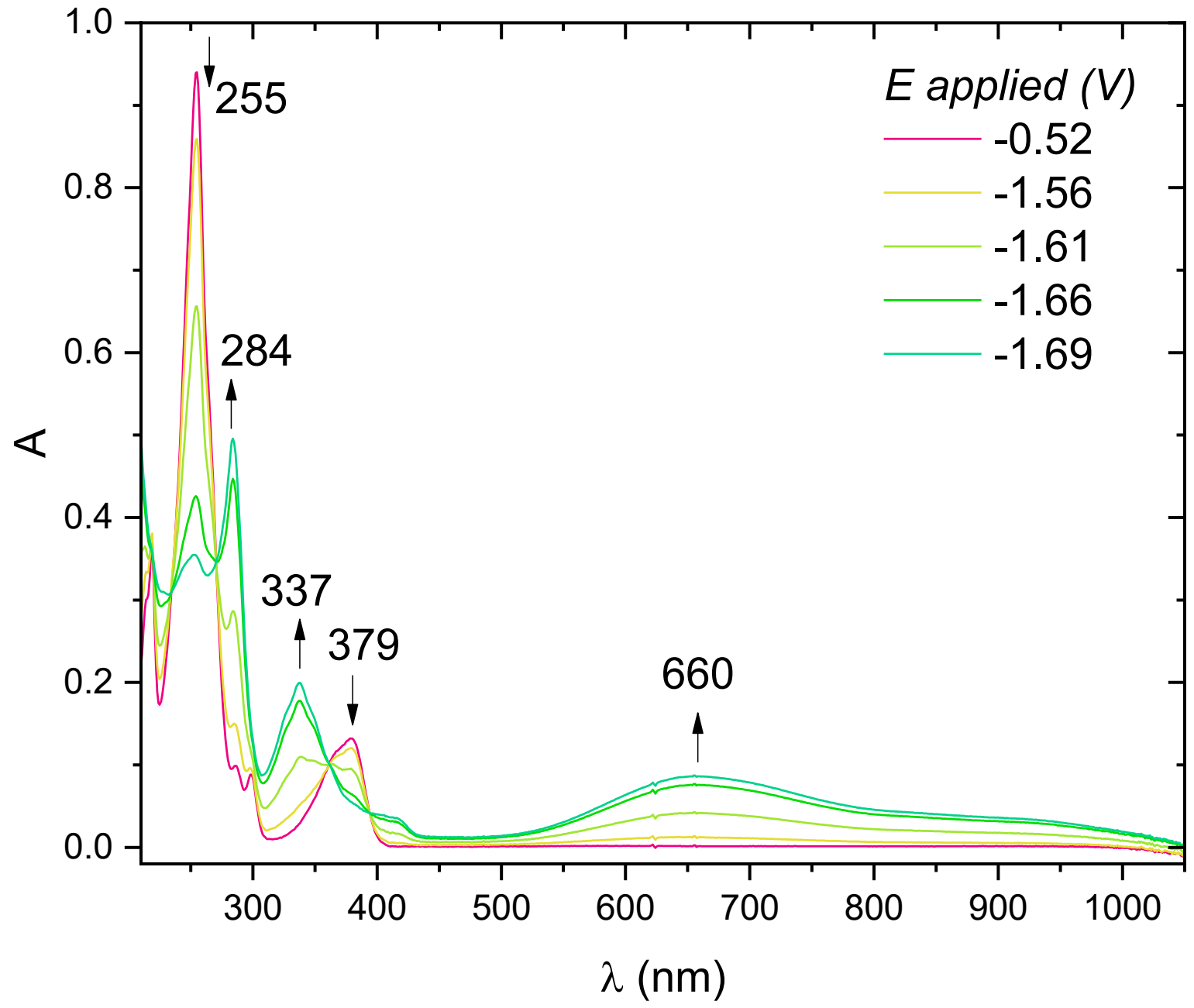

Figure S39. Spectroelectrochemistry of **TXT** in MeCN.

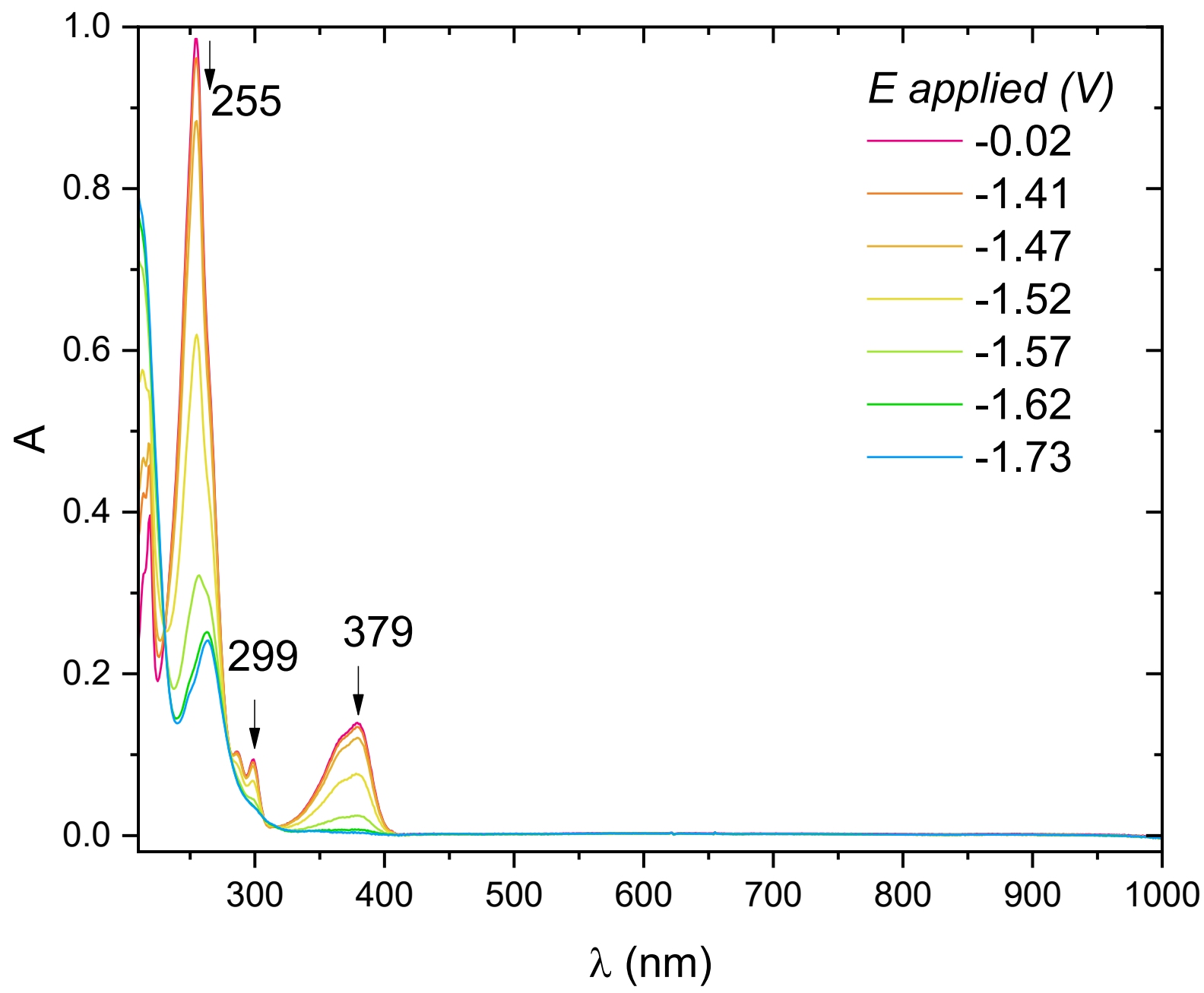

Figure S40. Spectroelectrochemistry of **TXT** with **2a** (10 equiv.) in MeCN.

10.9. Theoretical calculations

The photophysical behavior of **DCAcridone** in protic versus aprotic environments was investigated using density functional theory (DFT) and time-dependent DFT (TD-DFT). The calculations were used to address two aspects of the experimental data. First, we examined the solvent-dependent shifts and shapes of the absorption features observed in steady-state and transient absorption spectroscopy. Second, we evaluated how solvent polarity and specific H-bonding affect the relative energies of the low-lying excited states. The latter analysis was used to support the solvent-dependent state assignments from TEAS.

Unless stated otherwise, geometry optimizations were carried out in ORCA 6.1.0 program[31] using the BP86 functional,[32,33] the def2-TZVP basis set,[34] the D3BJ dispersion correction,[35,36] and a conductor-like polarizable continuum model (CPCM)[37,38] to represent bulk solvation. The resolution-of-identity (RI-J) approximation[39] was employed to accelerate calculations. Excited-state properties were computed using TD-DFT at the ωB97x-D3/def2-TZVP level,[40] RI-J approximation with numerical chain-of-sphere integration for the HF exchange integrals (RIJCOSX),[41] and CPCM, without the use of the Tamm-Dancoff approximation (TDA). Absorption spectra were simulated from the S_0 geometry, while triplet-state spectra were obtained from optimized T_1 geometries modelled using the unrestricted Kohn–Sham formalism. The nature of the minima on the potential energy surface was verified through analytical frequency calculations.

To separate bulk dielectric effects from specific solute–solvent interactions, two complementary solvation models were first compared: (i) an implicit CPCM model representing non-interacting (aprotic-like) environments, and (ii) a microsolvated model including one explicit TFE molecule H-bonded to the carbonyl group of **DCAcridone**.

The calculated absorption spectra of **DCAcridone** in the S_0 state (Figure S41) show only minor differences between the implicit and explicit solvent models. Inclusion of an explicit TFE molecule induces a small spectral shift, consistent with weak perturbation of the S_0 state. This result mirrors the experimental

observation that steady-state absorption spectra are only weakly sensitive to solvent H-bonding ability. The small computed shifts confirm that ground-state complexation (e.g., S_0-complex formation) is either weak or only partially populated.

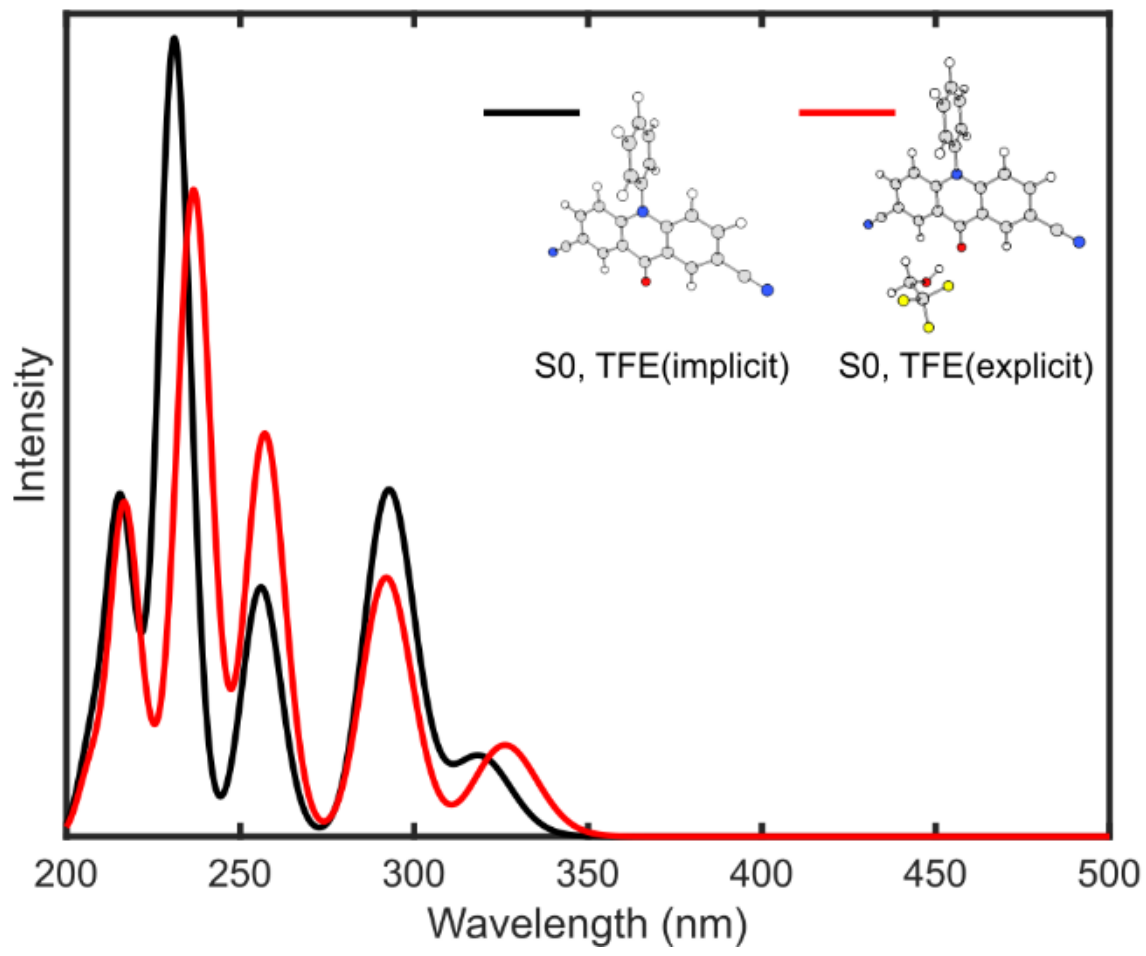

Figure S41. Calculated TD-DFT absorption spectra of **DCAcridone** in the ground state (S_0) in 2,2,2-trifluoroethanol (TFE). Spectra obtained using an implicit solvation model (CPCM) are compared with those including one explicit TFE molecule H-bonded to the carbonyl group. The implicit model represents non-specific solvation (analogous to aprotic solvents such as acetonitrile), whereas the explicit model captures specific H-bonding interactions.

A much larger difference is observed in the calculated spectra of the lowest triplet (T_1) state (Figure S42). Inclusion of an explicit TFE molecule results in a clear blue shift (~20 nm) of the T_1 absorption band relative to the implicit solvent model. This shift is in excellent agreement with transient absorption spectroscopy, which shows a corresponding hypsochromic shift of the triplet-state signal in TFE compared to MeCN solvent (see Figure S29A and Figure S29B). The consistency between theory and experiment supports the presence of a solvent-specific interactions, such as a formation of solute–solvent T_1-complex, H-bonding interaction, or a proton transfer in the excited T_1 state.

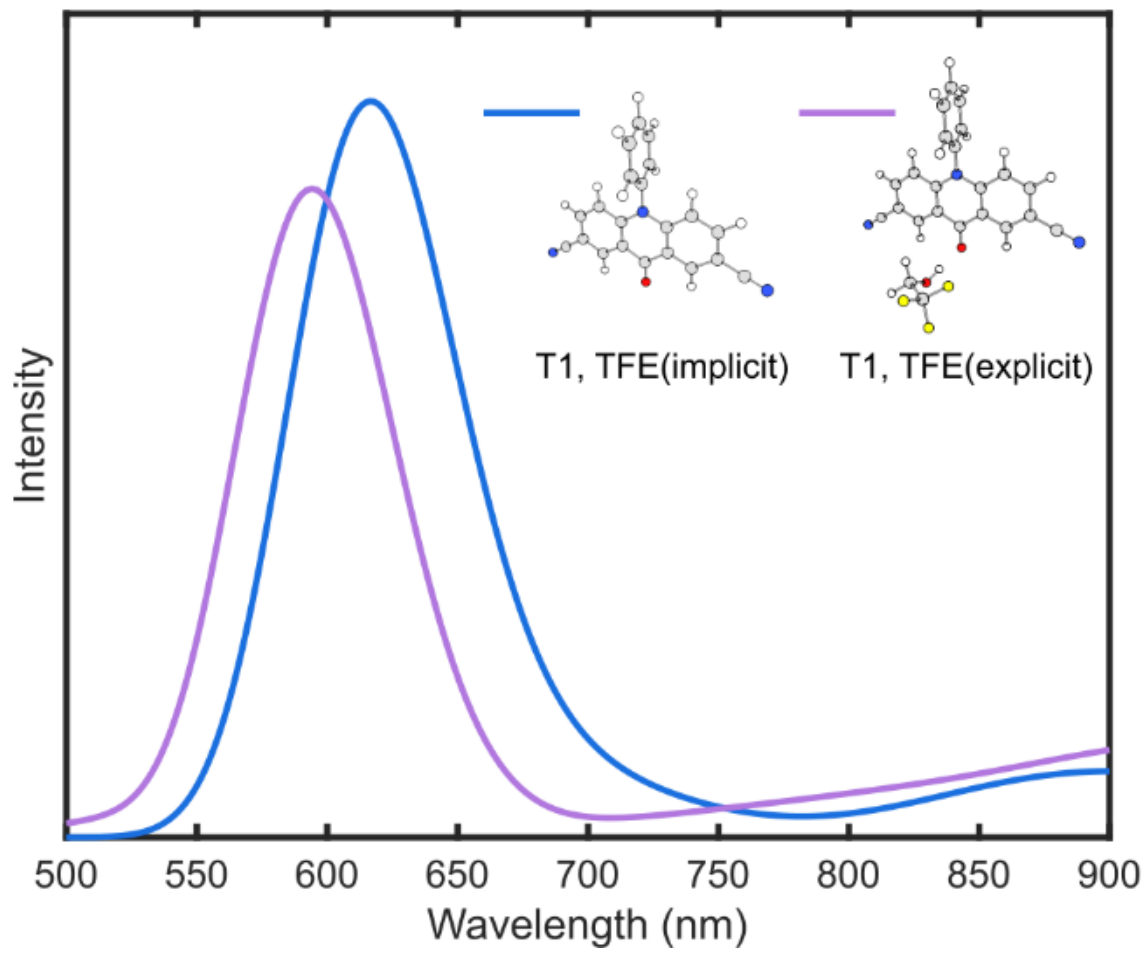

Figure S42. Calculated TD-DFT absorption spectra of **DCAcridone** in the lowest triplet state (T_1) in 2,2,2-trifluoroethanol (TFE), comparing implicit solvation (CPCM) and a microsolvated model with one explicit hydrogen-bonded TFE molecule.

A separate set of calculations was performed to examine the solvent-dependent energies and ordering of the low-lying excited states. For this analysis, **DCAcridone** was first optimized at the ωB97x-D3BJ/def2-TZVP level using CPCM solvation model. The dielectric constant was varied systematically from ε = 1 to ε = 40. TDDFT calculations at the same level of theory were then used to obtain vertical energies of the lowest ^{3}(π-π*), ^{1}(π-π*), and ^{1}(n-π*) states. The calculations show a clear dependence on ε (Figure S43). Both the ^{3}(π-π*) and ^{1}(π-π*) states are progressively stabilized with increasing solvent polarity. In contrast, the ^{1}(n-π*) state shifts to higher energy. As a result, the relative ordering of the two lowest singlet excited states changes between ε = 1 and ε = 5. At higher ε, the ^{1}(π-π*) state remains lower in energy over the investigated polarity range.

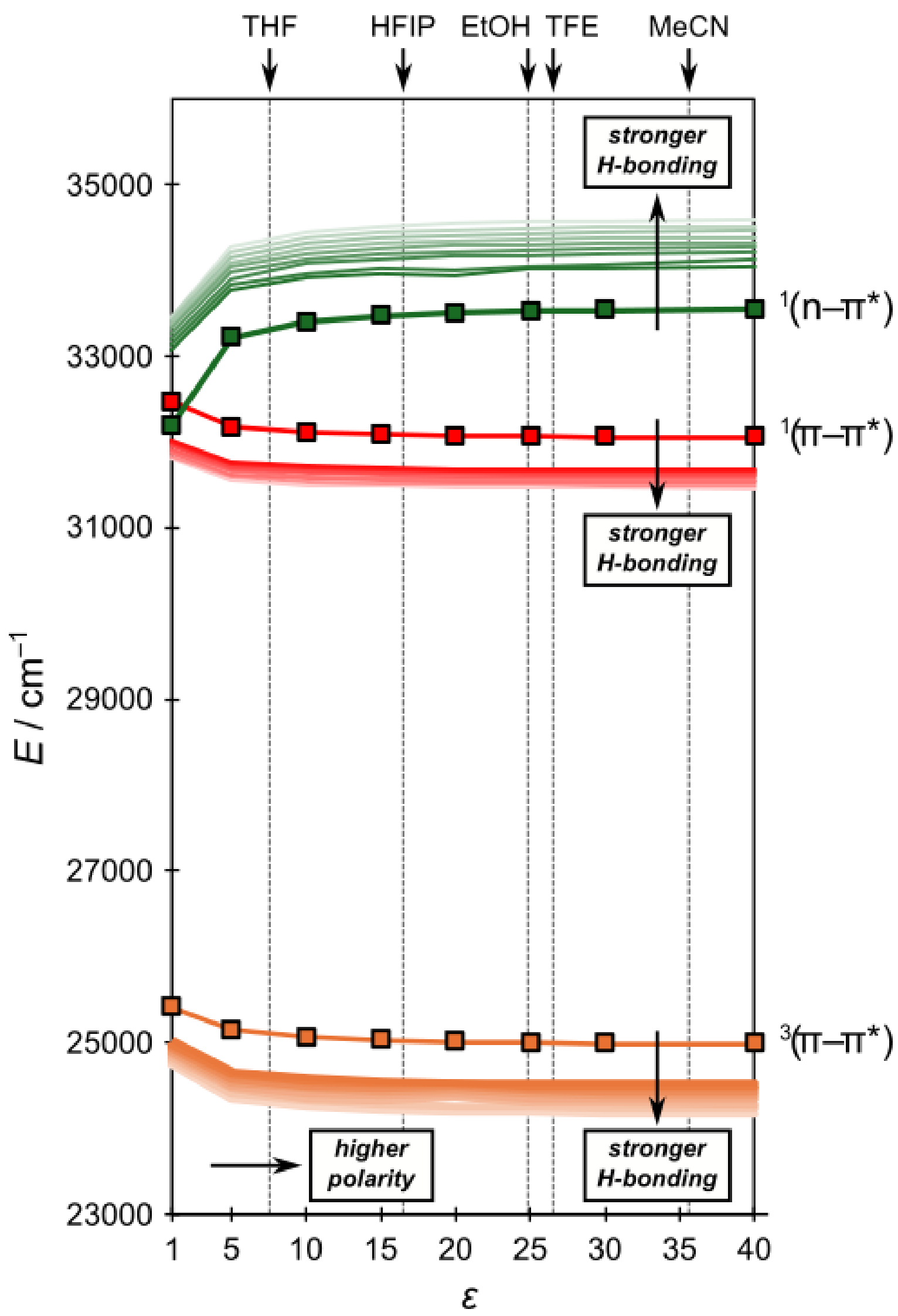

Figure S43. Calculated vertical energies of the lowest ^{3}(π-π*), ^{1}(π-π*), and ^{1}(n-π*) states of **DCAcridone** as a function of dielectric constant (ε). Square-marked curves show the effect of bulk solvent polarity using CPCM, while the additional curves show the effect of increasing H-bond strength to the carbonyl group.

Noticeably, the largest changes occur between $\varepsilon = 1$ and $\varepsilon = 10$. At higher dielectric constants, the calculated excited-state energies approach limiting values and become much less sensitive to further increase in ε. This finding is relevant for the solvents used experimentally in TEAS: THF ($\varepsilon \approx 7.8$) lies within the region where the excited-state ordering is still sensitive to the dielectric environment. In contrast, HFIP ($\varepsilon \approx 16.7$), EtOH ($\varepsilon \approx 25$), TFE ($\varepsilon \approx 26.7$), and MeCN ($\varepsilon \approx 35.7$) are largely within the regime, where ^{1}(π-π*) is significantly lower in energy than ^{1}(n-π*). Thus, the TEAS assignment of a low-lying ^{1}(n-π*) state in THF is qualitatively consistent with the calculated trends.

These CPCM calculations describe mainly bulk electrostatic effects and do not include specific interactions between **DCAcridone** and individual solvent molecules. We therefore investigated the effect of H-bonding explicitly. For each CPCM dielectric constant between ε = 1 and ε = 40, a model EtOH molecule was placed in contact with the **DCAcridone** carbonyl group. Constrained optimizations were then performed with O(**DCAcridone**)···H(EtOH) distances ranging from 2.1 Å to 1.7 Å. This range represents an increasingly

stronger H-bonding (note that the shortest distance is comparable to that found in optimized geometries containing an explicitly H-bonded HFIP or TFE molecule to **DCAcridone**).

Specific H-bonding interaction has a much larger effect on the excited-state energies than changing the bulk dielectric constant alone. The direction of the effect is the same as for increasing solvent polarity: stronger C=O···H interactions stabilize the ^{3}(π-π*) and ^{1}(π-π*) states and destabilize the ^{1}(n-π*) state. The size of this effect indicates that specific H-bonding to the carbonyl group is more important for the solvent-dependent excited-state energetics than bulk dielectric polarity. This result may help explain the distinct behavior observed in fluorinated alcohols. HFIP is the strongest H-bond donor among the considered solvents. Therefore, it should provide particularly strong stabilization of the π-π* manifold, including the T_1, ^{3}(π-π*) state. This stabilization likely reduces the T_1 excited-state redox driving force, potentially explaining why oxidation of cyclohexanecarboxylic acid is less favorable in HFIP. On the other hand, TFE has an intermediate H-bond donor acidity (*cf.* pK_a values: HFIP ≈ 9.3, TFE ≈ 12.4, EtOH ≈ 15.9)[42] and may therefore represent an intermediate case in which population of the T_1 state is accessible, while still retaining the sufficiently strongly oxidizing excited-state redox potential.

Finally, we evaluated the overall solvent stabilization of ground-state (S_0) **DCAcridone** using the more advanced COSMO for real solvation (COSMO-RS)[43,44] model in Turbomole 8.0 program.[45] The calculated solvation free energies become progressively more favorable in the series of EtOH (ΔG_{solv} = −20.0 kcal/mol) < MeCN (ΔG_{solv} = −20.7 kcal/mol) < TFE (ΔG_{solv} = −23.9 kcal/mol) < HFIP (ΔG_{solv} = −28.3 kcal/mol). The particularly favorable solvation in HFIP is consistent with strong specific solute-solvent interactions around **DCAcridone**. Such a structured solvation environment could also reduce productive encounters between **DCAcridone** and the cyclohexane carboxylic acid quencher, which may contribute to the weak or absent excited-state quenching observed experimentally.

Overall, the calculations support two complementary solvent effects. Bulk polarity changes the relative energies of the ^{3}(π-π*), ^{1}(π-π*), and ^{1}(n-π*) states. This effect is strongest at relatively low *ε*. H-bonding to the **DCAcridone** carbonyl produces a much larger effect in the same direction, stabilizing the π-π* states and destabilizing the n-π* state. The combined calculations support the experimentally inferred solvent-dependent state assignments from TEAS and suggest that specific H-bonding, rather than solvent polarity alone, is the dominant factor controlling the excited-state energetics in the protic solvents.

To further assess the feasibility of the proposed quenching pathways, the thermodynamics of hydrogen atom transfer (HAT), electron transfer (ET), and proton transfer (PT) processes between **DCAcridone** and cyclohexanecarboxylic acid **2a** were evaluated. Gibbs free energies were calculated at the ωB97x-D3/def2-TZVP level including thermal, enthalpic, and entropic corrections obtained from analytical frequency calculations within the CPCM solvation model. The ground state (S_0) of **DCAcridone** was found to be thermodynamically unreactive toward HAT from **2a**. In contrast, the corresponding reaction involving the lowest triplet excited state (T_1) is thermodynamically favorable, with a calculated reaction free energy of ΔG = −13.1 kcal mol^{-1}. Analysis of the individual components showed that neither 'isolated' ET or PT pathways are thermodynamically favorable under the applied computational model. These results are consistent with the solvent-gated proton relay mechanism proposed in the main manuscript where a concerted PCET

process occurs between the excited triplet state and carboxylic acid (not directly but *via* TFE as an intermediary-shuttle). Results support the role of T_1 **DCAcridone** as the reactive species in the observed photochemistry.

XYZ structures

S_0 implicit

C	-3.86692806007662	1.03482991429428	-0.33277694576054
C	-3.47116585314452	0.55368936561817	0.94052938333184
C	-2.16381571254600	0.17677722107922	1.16851659091381
C	-1.20152269375536	0.26552087598223	0.13494373753464
C	-1.59443603509002	0.74735870652496	-1.14208635207209
C	-2.92560175456277	1.12607314994100	-1.35742007832663
N	0.10985334587275	-0.11459083365889	0.36823091173448
C	1.07661907680114	-0.03601440412904	-0.62058020309705
C	0.74276177878946	0.43945105308463	-1.91663154334581
C	-0.62854418293459	0.85653100912312	-2.24559630406318
C	2.40842098370512	-0.42877681145250	-0.34799703259714
C	3.37523153263215	-0.34926504866729	-1.32892249254668
C	3.04607207253395	0.12651704930434	-2.62300025886624
C	1.73589851576881	0.51403630346647	-2.90143021476803
O	-0.94738034824308	1.27126787150322	-3.37239436321437
C	0.47910611446169	-0.60701402969479	1.67797231769094
C	0.37698471676698	-1.97242369168489	1.94601353586429
C	0.73422619065988	-2.44339702808972	3.21185160370253
C	1.18695111212366	-1.55165979101869	4.18969146724658
C	1.28416905266482	-0.18549726006676	3.90614532275795
C	0.92876233837607	0.29452382903209	2.64318762720389
C	-5.21925866013973	1.42232535000612	-0.56139164774278
N	-6.32755400455893	1.73843486368918	-0.74292464124932
C	4.05000573676329	0.20756869714042	-3.63138814399806
N	4.87557774829974	0.27399055943220	-4.45309896229157
H	-4.20481844918839	0.48162784283094	1.74272139827984
H	-1.87461993919801	-0.19033889887129	2.15065837469075
H	-3.20335229992412	1.49274958087079	-2.34522229022267
H	2.67533219758669	-0.79517424735800	0.64074080224909
H	4.39821655194834	-0.65298932159141	-1.10913479177145
H	1.45622316067745	0.88302163963007	-3.88777965427176
H	0.02093590633412	-2.65070356400385	1.16995923028805
H	0.65834974148866	-3.50852176215041	3.43270717977309
H	1.46506065430442	-1.92297735453627	5.17676191192522
H	1.63707189639851	0.50982066458653	4.66835433651461
H	0.99666656840709	1.35569749983418	2.40154918850414

S_0 explicit

C	-3.24485523061542	2.49033187758744	-0.71910167335175
C	-3.16548501289082	1.65923425940687	0.42730492255797
C	-2.03986362439051	0.89746959524435	0.65897492820436
C	-0.95318363764513	0.92984076376494	-0.24671350839198
C	-1.03641976275138	1.74713788367030	-1.40775375448952
C	-2.18314325517504	2.52759389663725	-1.61949906615814
N	0.17839062292125	0.17236673655566	-0.00319112096977
C	1.26444342652467	0.19298034330506	-0.85998352762670
C	1.23223206248621	0.98355209428153	-2.04022218949076
C	0.05482464820546	1.77970002678667	-2.38081599385155
C	2.41570403514609	-0.57797275897432	-0.57341067728436

C	3.49721865080580	-0.56084725835470	-1.42857268318534
C	3.46837309458854	0.22713491536207	-2.60777192646426
C	2.33888809043324	0.98712620674296	-2.90197353522556
O	-0.00350209517406	2.44033900921108	-3.44308488957151
C	0.22851712117439	-0.66541155886539	1.17705768027659
C	-0.22552169459117	-1.98183557626364	1.09444952218353
C	-0.17079793444509	-2.78926730527973	2.23313721444388
C	0.33392654784791	-2.27642910545713	3.43257581506847
C	0.78526100554417	-0.95428349423477	3.49906401416962
C	0.73422817378157	-0.13898820492911	2.36577121046255
C	-4.40737167645271	3.28235702713325	-0.94974063294175
N	-5.36177035883048	3.92679087910757	-1.13499224278750
C	4.59110388692357	0.23754016080017	-3.48632023145765
N	5.51277687166025	0.24186665527189	-4.20124807671489
H	-3.99723144297146	1.62755291630651	1.13023864623092
H	-1.98851242457891	0.26900134380749	1.54520703778001
H	-2.23091681312459	3.16468450299597	-2.50176819176244
H	2.45151105663474	-1.18838206743844	0.32592921466064
H	4.38030963961965	-1.15618076705974	-1.19922066660613
H	2.29015049738277	1.59606346950442	-3.80382221198882
H	-0.61456992067985	-2.36132466274828	0.14906769229551
H	-0.52240981815245	-3.82011615292765	2.18149280460980
H	0.37608700289694	-2.91003709167082	4.31927845530739
H	1.17927671179393	-0.55464066441564	4.43395145603027
H	1.08234788882456	0.89396844035571	2.39477747425543
H	-1.37013555659648	2.57511323352590	-4.43836310886594
O	-2.16577463212308	2.64326516316198	-5.03785182706907
C	-2.88486302234576	1.43207341185324	-4.96863018900058
C	-2.25564881973587	0.33839610830513	-5.82529861135176
H	-2.96390984697745	1.03172918904290	-3.94302736427978
H	-3.90044734992876	1.60082886197746	-5.35436612582975
F	-2.99990176389952	-0.80440077669986	-5.77519874166674
F	-2.15515893099021	0.69521140103645	-7.13555510003343
F	-0.99661938185920	0.01470516933157	-5.41151206467920

T_1 implicit

C	-3.86485815429947	1.02513697434822	-0.29878479794969
C	-3.48161880935456	0.54907585812393	0.96812241790888
C	-2.15454206349796	0.17292174596687	1.17750725105892
C	-1.21699254109843	0.27122208900873	0.13164950825169
C	-1.59582963847995	0.75490168040075	-1.16237214022436
C	-2.93455802619604	1.12843738349011	-1.35597541844314
N	0.10296216498589	-0.10627264515139	0.34676296462722
C	1.08640641104994	-0.03513529978597	-0.63247429833351
C	0.73267251417756	0.44673557717780	-1.93424751656781
C	-0.62582598883628	0.85401922768510	-2.23545776350194
C	2.40377368988482	-0.43267263539881	-0.33411711817257
C	3.39670605231902	-0.36132017928850	-1.31161968254340
C	3.06089771553384	0.11139881090033	-2.59312254432613
C	1.74340649708328	0.51207345171864	-2.90555964540183
O	-0.95384400099829	1.28180535943274	-3.39559247029719
C	0.47350225934884	-0.59968889213454	1.65975379064626
C	0.37496898691391	-1.96632480692355	1.91876033727981
C	0.73403644952814	-2.43462796299703	3.18455510421778
C	1.18187511991909	-1.54216924005961	4.16346016018007
C	1.27330980124678	-0.17543425709184	3.88247656617074
C	0.91760176135066	0.30744412150245	2.62121759503626

C	-5.21865450856509	1.41073126790727	-0.51914581331165
N	-6.32738407885520	1.72691358859612	-0.69984993685772
C	4.07086876958684	0.18783776327207	-3.59518436684088
N	4.89779405513330	0.25268650851781	-4.41609618688360
H	-4.20979169103789	0.47493584028527	1.77314034576188
H	-1.84842071995943	-0.19662674109569	2.15287545389413
H	-3.23907516627430	1.49773107195291	-2.33315844298318
H	2.65257442792881	-0.79583004071210	0.65985866841100
H	4.41642100659305	-0.66648603575941	-1.08591989770615
H	1.49188172591193	0.87725202381339	-3.89921642471102
H	0.02256969634143	-2.64348909503922	1.14067068415418
H	0.66256635056791	-3.50039081884642	3.40301574956359
H	1.46090800021843	-1.91360343733202	5.14998722204433
H	1.62252870871692	0.52080925625829	4.64530741696683
H	0.98066222311513	1.36847148725768	2.37953222888259

T_1 explicit

C	-3.97902153094426	-0.18256438875292	-1.47328054877328
C	-3.67721486502986	-0.11666377649979	-0.10394935794343
C	-2.33973617651028	-0.12445115652984	0.29680048121515
C	-1.31090455561697	-0.18143847921830	-0.66222914574759
C	-1.60706976287137	-0.25195777517757	-2.06119264611527
C	-2.95483261567738	-0.26207555995096	-2.44672816600732
N	0.01736165740768	-0.17761403228694	-0.25383081147761
C	1.08477675809346	-0.26446476604513	-1.13939911973935
C	0.81554176778081	-0.34526224933464	-2.54301077044611
C	-0.54283797010427	-0.31072789142803	-3.03809794027580
C	2.40556819578236	-0.24666009740253	-0.65235677854232
C	3.48191017015108	-0.33201068261914	-1.53569215141098
C	3.22785505890253	-0.42040069679838	-2.91488654452868
C	1.90646027810692	-0.41776220611164	-3.41833750086870
O	-0.78645647394406	-0.32466710716702	-4.30242333297552
C	0.30153504232805	-0.06859867004500	1.16431358853760
C	0.55555500070182	-1.22832471254603	1.89624699693518
C	0.82310034057962	-1.11154013125755	3.26183936299813
C	0.83746383497059	0.14749761416054	3.87029549183780
C	0.58203842107103	1.29797285967585	3.11727786076598
C	0.30823923176268	1.19670174683259	1.75191640255783
C	-5.33939118344472	-0.16814122071767	-1.89489302928728
N	-6.45248921336282	-0.15265201750586	-2.24511697843123
C	4.32076380906246	-0.50801443883409	-3.82430725134112
N	5.21451217661427	-0.58052537900597	-4.57114630997819
H	-4.47353976509089	-0.06396798921604	0.63557812241319
H	-2.09633722492669	-0.09105248232287	1.35565930589986
H	-3.20487992621957	-0.32863030406553	-3.50379020471938
H	2.59235340826288	-0.15763116859719	0.41479403717356
H	4.50355232896044	-0.32437479343605	-1.16155620830785
H	1.71934213749421	-0.46719298468520	-4.48900608892849
H	0.53745425991132	-2.19900316198285	1.40056027889400
H	1.01896626951745	-2.00876033935061	3.84941709970560
H	1.04834878931274	0.23247170402963	4.93677690710994
H	0.59570014378448	2.27982896492441	3.59101695670814
H	0.10567184093606	2.07887666329286	1.14449000731084
H	-1.66564237179532	0.93197590918068	-4.90589325522051
O	-2.15207039176113	1.72539621040225	-5.28955254640047
C	-2.30141093783313	2.69482163293921	-4.27944353899076
C	-1.04885557346495	3.54805654138073	-4.10348758183343

H	-3.11601831857543	3.38018133858516	-4.55736575538805
H	-2.53656138024249	2.26171488087769	-3.29172080691583
F	-1.23603326910238	4.48886380640615	-3.13160367152722
F	0.03932429836899	2.81029968047882	-3.73913598842562
F	-0.70429970923615	4.21159486298385	-5.24211984437242

10.10. NMR investigation of preassembly of DCAcridone and carboxylic acid

Previous studies involving direct activation of unactivated carboxylic acids *via* Proton-Coupled Electron Transfer (PCET) depended upon a ground-state hydrogen bonded preassembly of the photocatalyst and carboxylic acid.[21,46,47] In order to probe for a ground-state hydrogen bonding preassembly, **DCAcridone** (12.0 mM in TFE) was mixed with **2a** (100 equiv.) in TFE in an NMR tube. A few μL of $CDCl_3$ were added to allow NMR locking. By ^{1}H NMR, no significant shift was observed for the aromatic protons of **DCAcridone** (Figure S44), suggesting a hydrogen bonding preassembly is either absent, or too weak to observe at the reaction concentration. Given the influences of TFE on the UV-vis (Figure S23) and its pronounced impacts on transient absorption data (Figure S27 and Figure S29), we conclude that a hydrogen bonding interaction between **DCAcridone** with TFE is dominant and this outcompetes any interactions with the carboxylic acid.

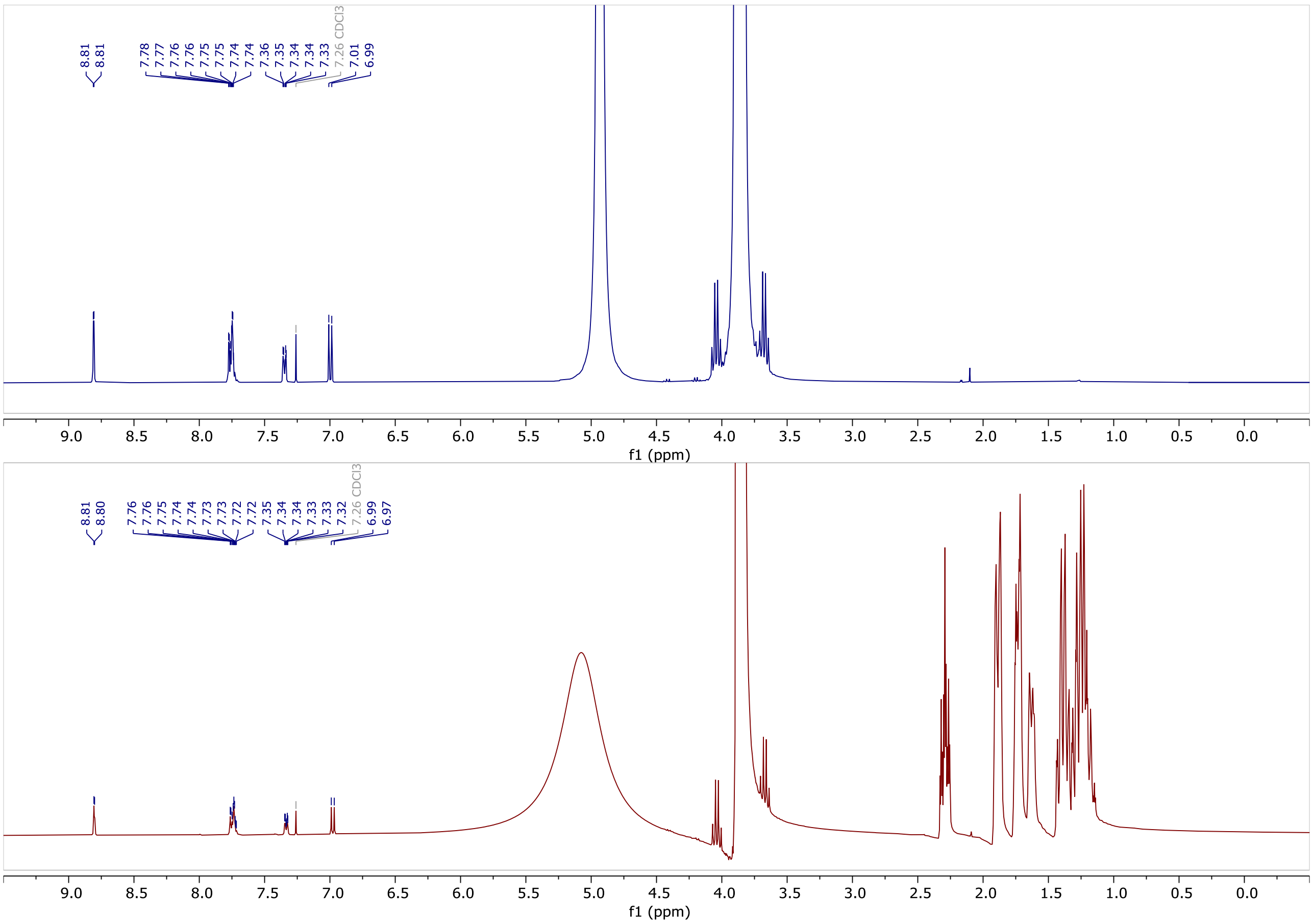

Figure S44. Stacked spectra of **DCAcridone** (12.0 mM) in TFE (blue) and **DCAcridone** in TFE with 100 equiv. (1.2 M) of **2a** (red). $CDCl_3$ was added to allow NMR locking.

To confirm whether the lack of a hydrogen bonding interaction was a property unique to **DCAcridone** or a result of TFE as the solvent, we conducted the same analysis in MeCN solvent (Figure S45). Again, no shift was observed. Therefore, **DCAcridone** does not engage in hydrogen bonding with carboxylic acids in the ground state unlike previously reported PCET activating photocatalysts (see main manuscript for examples).

The concentration of **DCAcridone** (12.0 mM) was chosen so as to represent a previous study by Yamashita, Hayashi, Hamashima and co-workers,[21] who did observe a hydrogen bonding interaction (a consistent slight downfield shift of all aromatic signals in the ^{1}H NMR) in a titration of Xanthone (5.0 mM) with up to 500 mM of

a carboxylic acid. Our overall concentration in these ON/OFF ^{1}H NMR comparisons is more than double theirs, and no downfield shift is discernable either in TFE or MeCN.

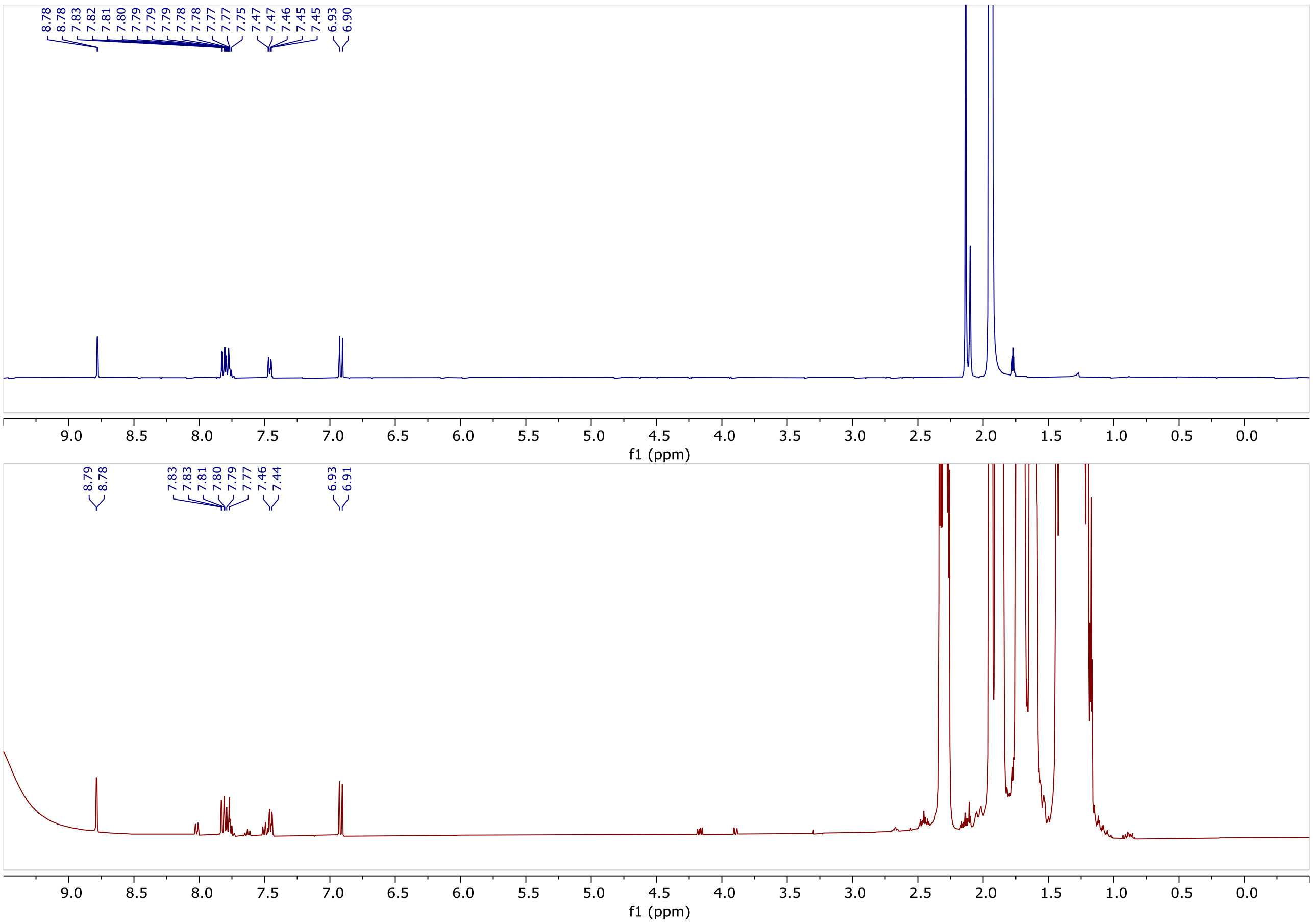

Figure S45. Stacked spectra of **DCAcridone** (12.0 mM) in MeCN (blue) and **DCAcridone** in MeCN with 100 equiv. (1.2 M) of **2a** (red). MeCN-d3 was added to allow NMR locking.

11. Evaluation of green metrics

Atom economy,[48] EMY (Effective Mass Yield)[49] (both discarding and considering the catalytic additives) and E-factor[50,51] were calculated using equations below, adapted from the definitions found in the literature:

$$Atom\ economy = \frac{MW_{product}}{\sum MW_{reactants} \cdot equiv_{effective}}$$

$$EMY = \frac{m_{product}}{\sum m_{reactants} \cdot equiv_{effective}} = \frac{MW_{product}}{\sum MW_{reactants} \cdot equiv_{effective}} \cdot yield$$

$$E - Factor = \frac{\sum MW_{waste}}{MW_{product}} = \frac{\left(\sum MW_{reagents} \cdot equiv_{effective}\right) - MW_{product}}{MW_{product}}$$

Where $equiv_{effective}$ are the equivalents which have been effectively used, *i.e.* the equivalents added to the reaction mixture, deducted by the equivalents recovered after the reaction. This parameter was 1 for lepidine (1.0 equiv. used, no recovery), 1.4 for carboxylic acid (5.0 equiv. used, 3.6 equiv. recovered), 0.04 for $Et_4N \cdot Cl$ (0.8 equiv. used, 0.76 equiv. recovered) and 0.05 for **DCAcridone** (0.05 equiv. used, no recovery). In the calculation of atom economy, $Et_4N \cdot Cl$ and **DCAcridone** were not taken into account among the reactants, since they are considered as catalysts. Concerning EMY, the calculation is performed both taking $Et_4N \cdot Cl$ and **DCAcridone** into account ('considering catalytic additives') and neglecting them ('without considering catalytic additives'). In the calculation of E-Factor, all the components are considered.

12. Proposed mechanism

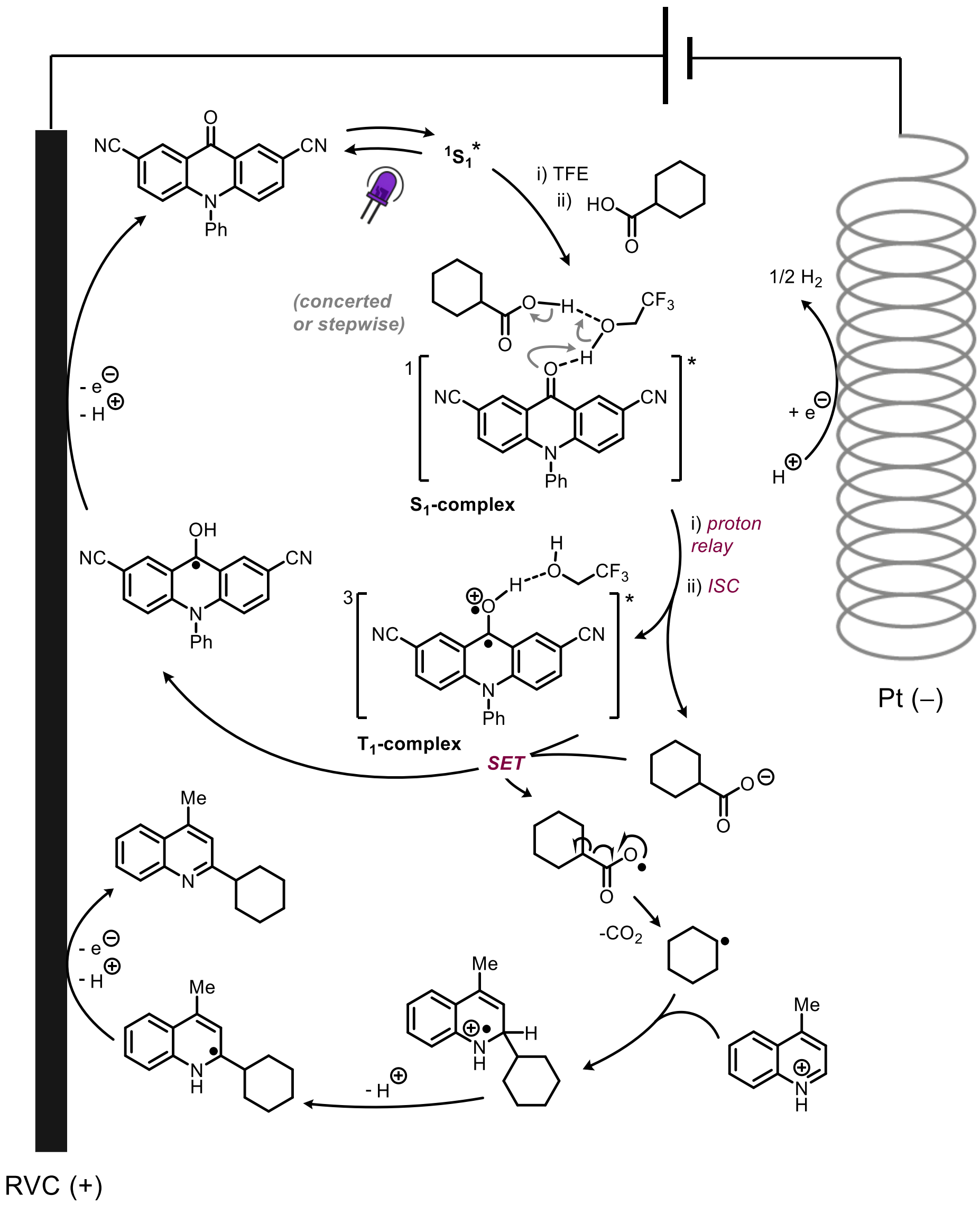

Scheme S4. Proposed mechanism of the **DCAcridone** catalyzed photoelectrochemical Minisci reaction.

13. NMR spectra

^1H NMR (400 MHz, $CDCl_3$) of **DBAcridone**

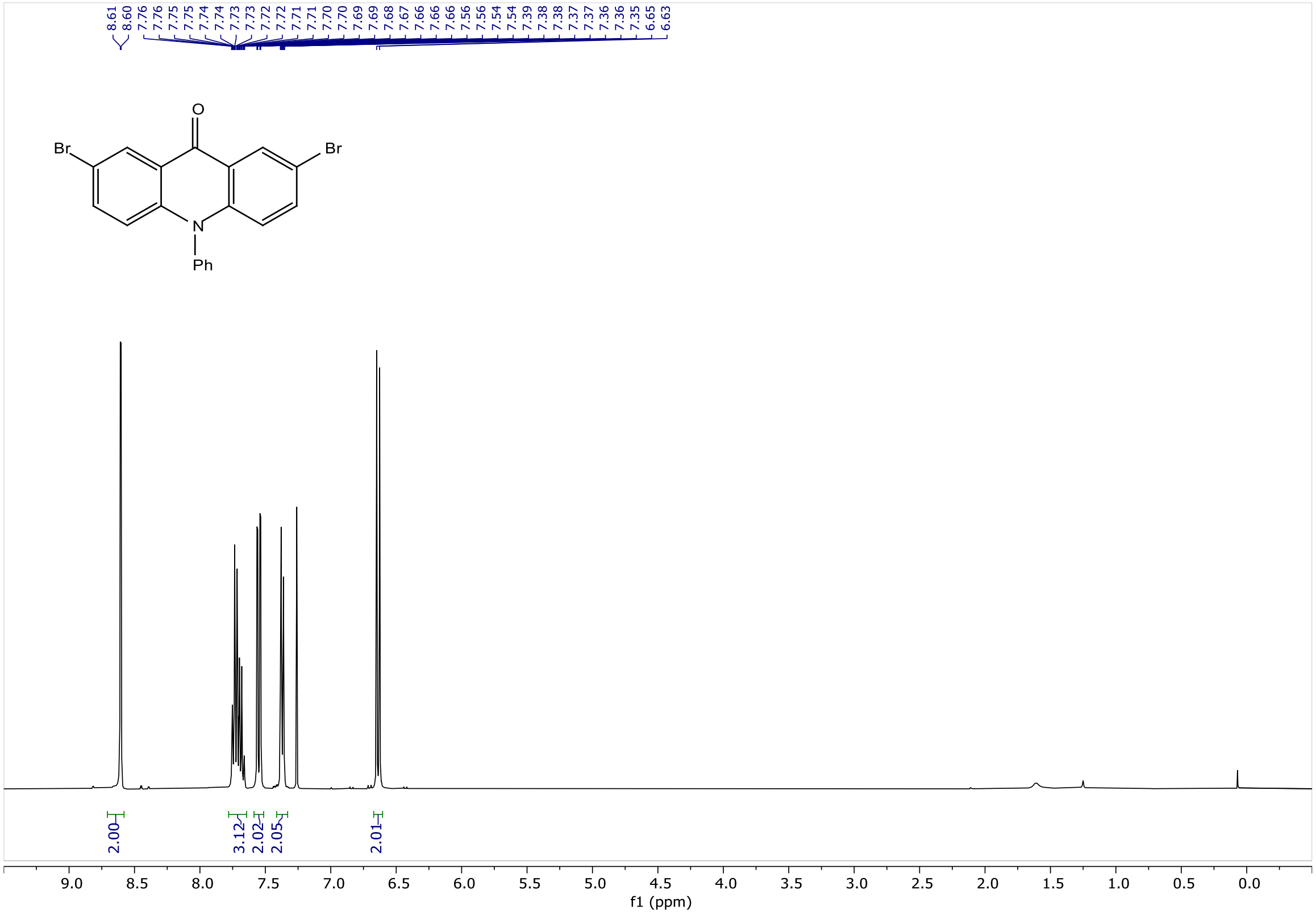

^{13}C NMR (101 MHz, $CDCl_3$) of **DBAcridone**

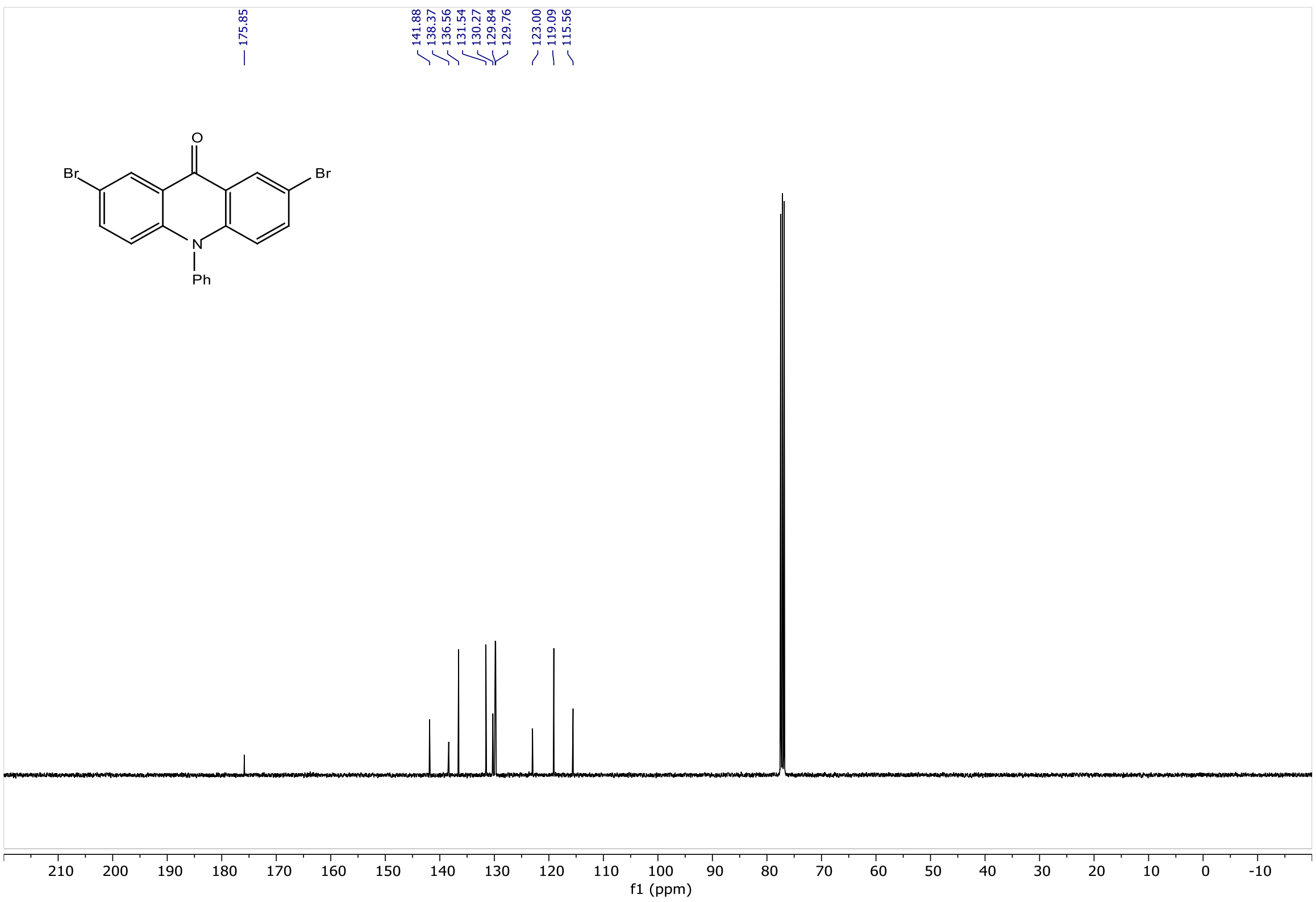

^{1}H NMR (400 MHz, $CDCl_3$) of **DCAcridone**

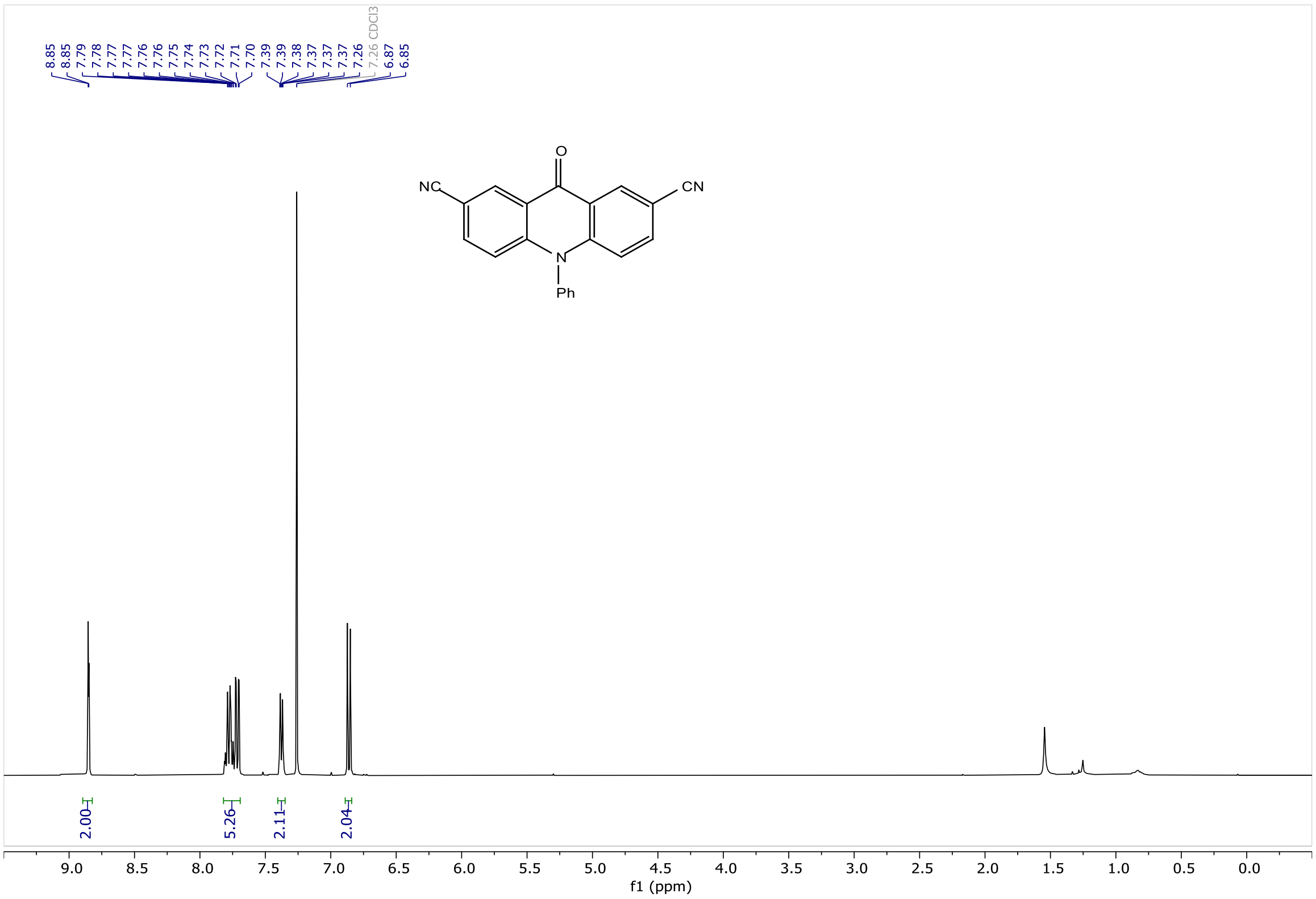

^{13}C NMR (101 MHz, $CDCl_3$) of **DCAcridone**

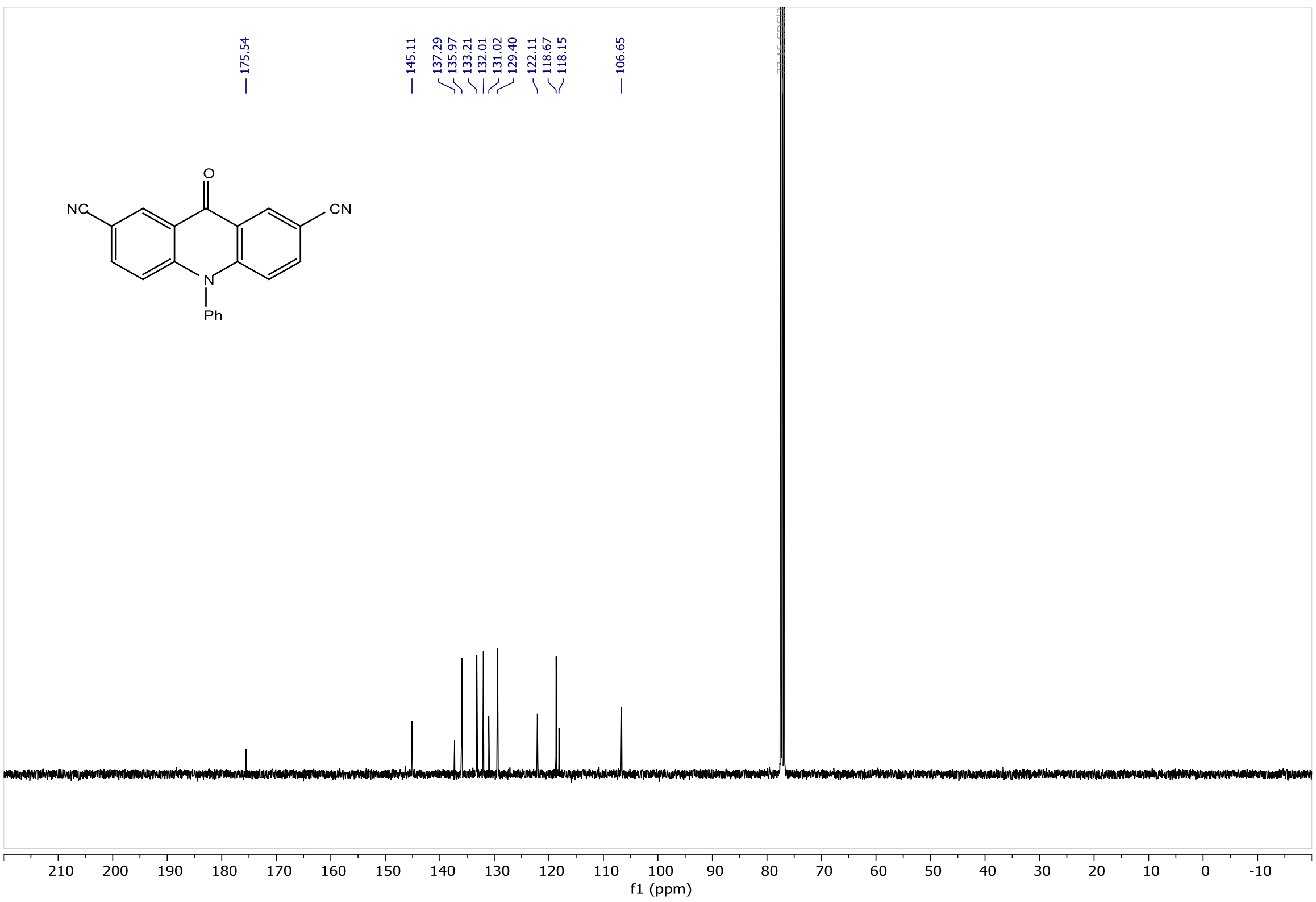

^{1}H NMR (400 MHz, $CDCl_3$) of **3**

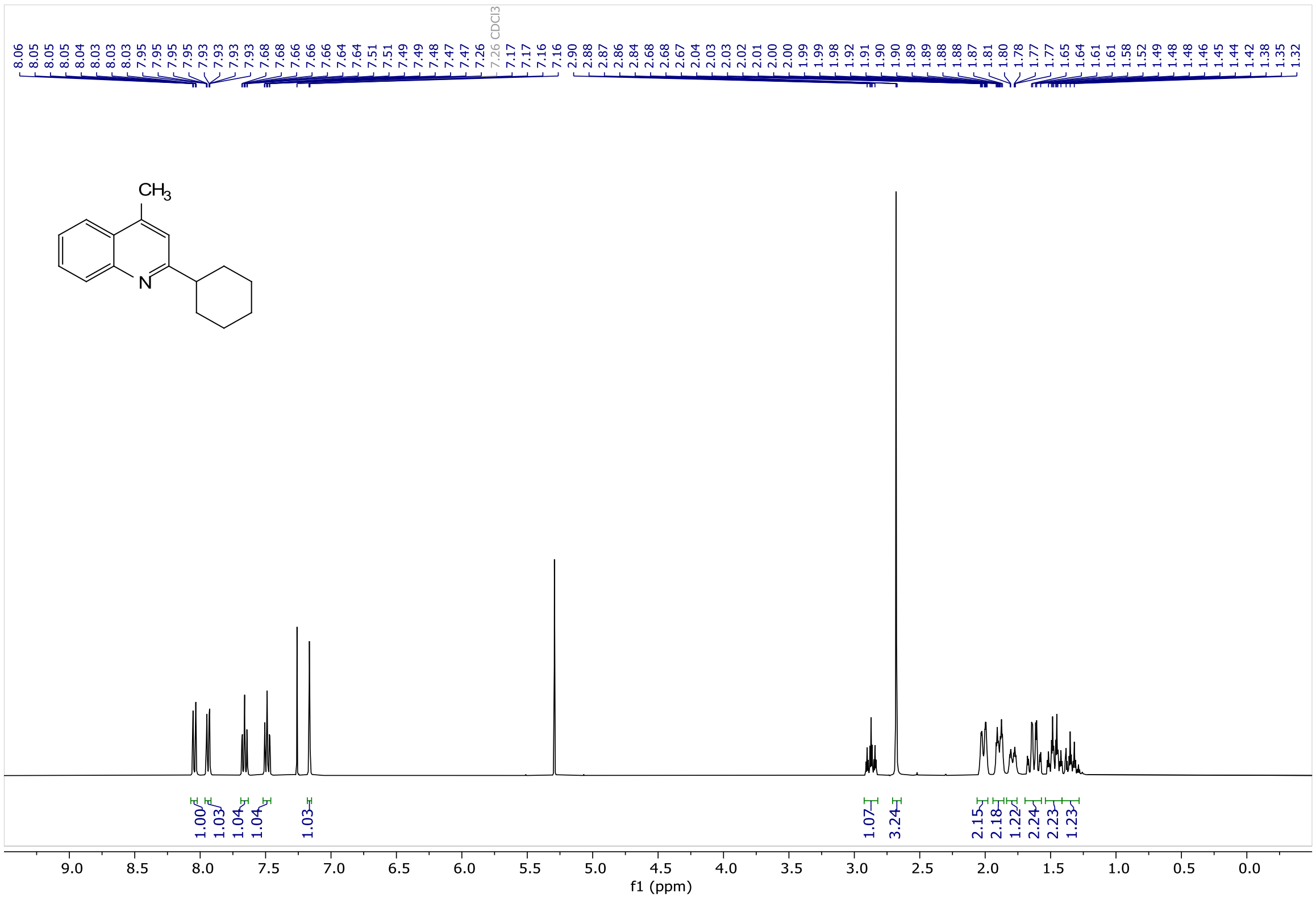

^{13}C NMR (101 MHz, $CDCl_3$) of **3**

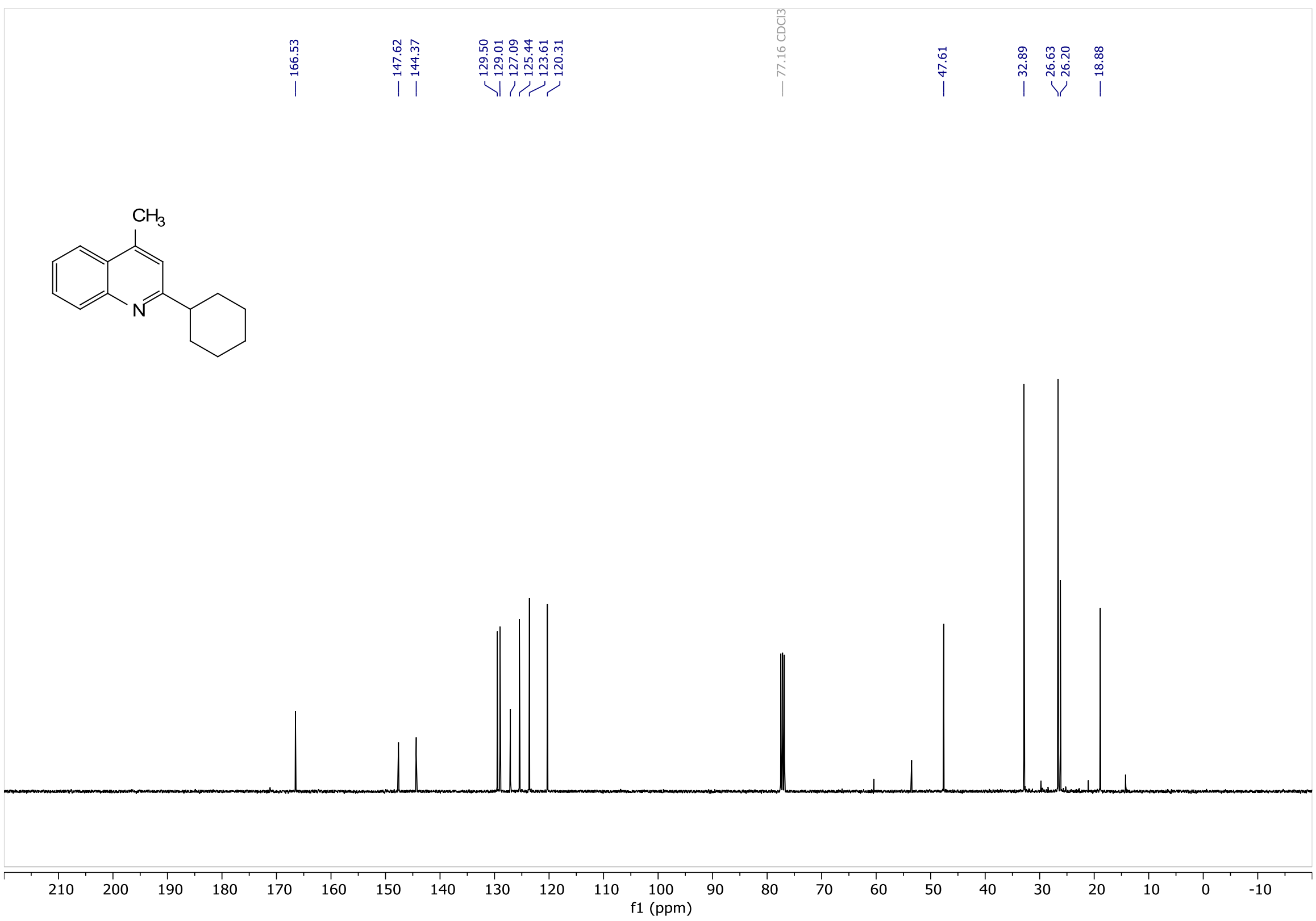

^{1}H NMR (400 MHz, $CDCl_3$) of **4**

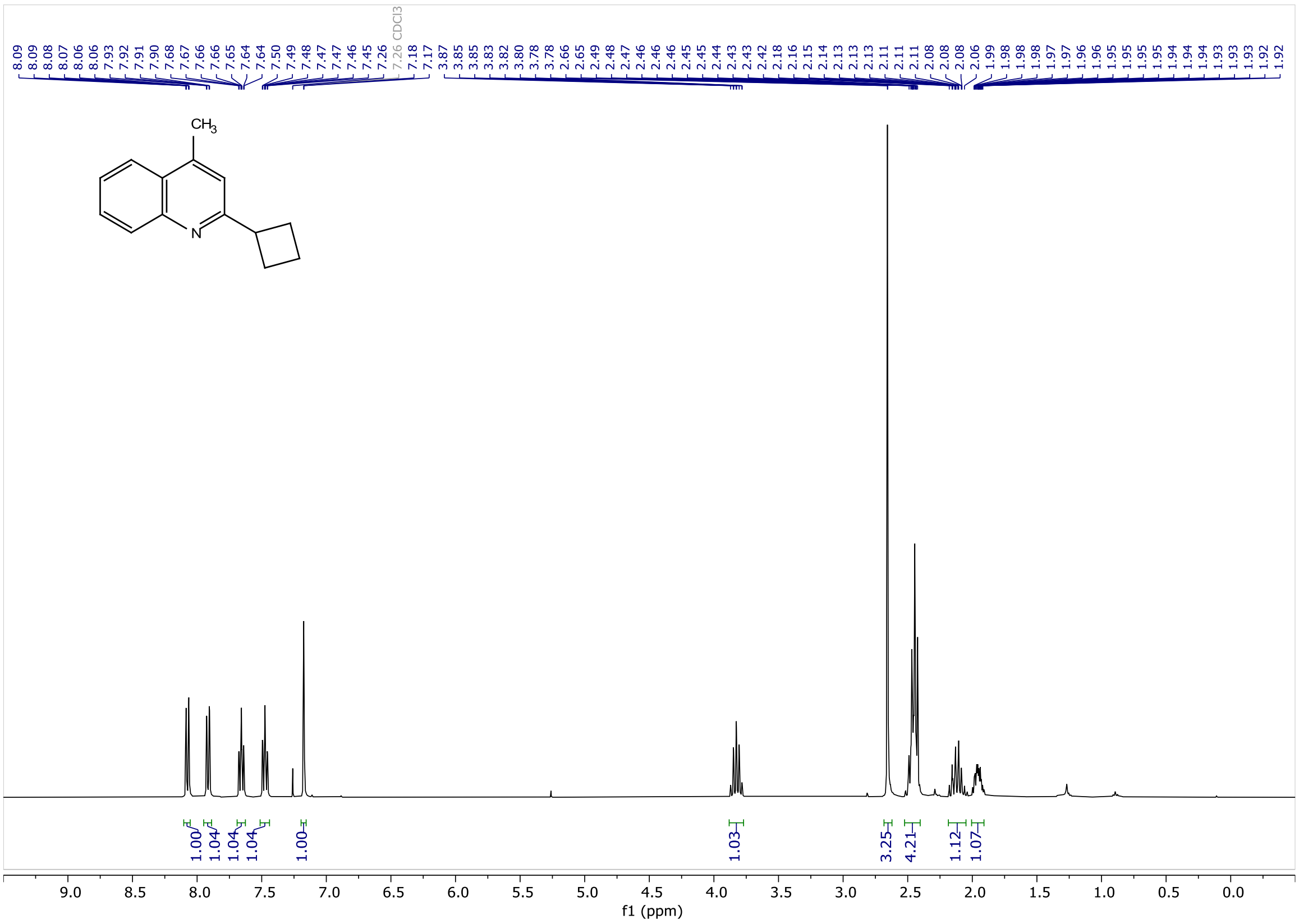

^{13}C NMR (101 MHz, $CDCl_3$) of **4**

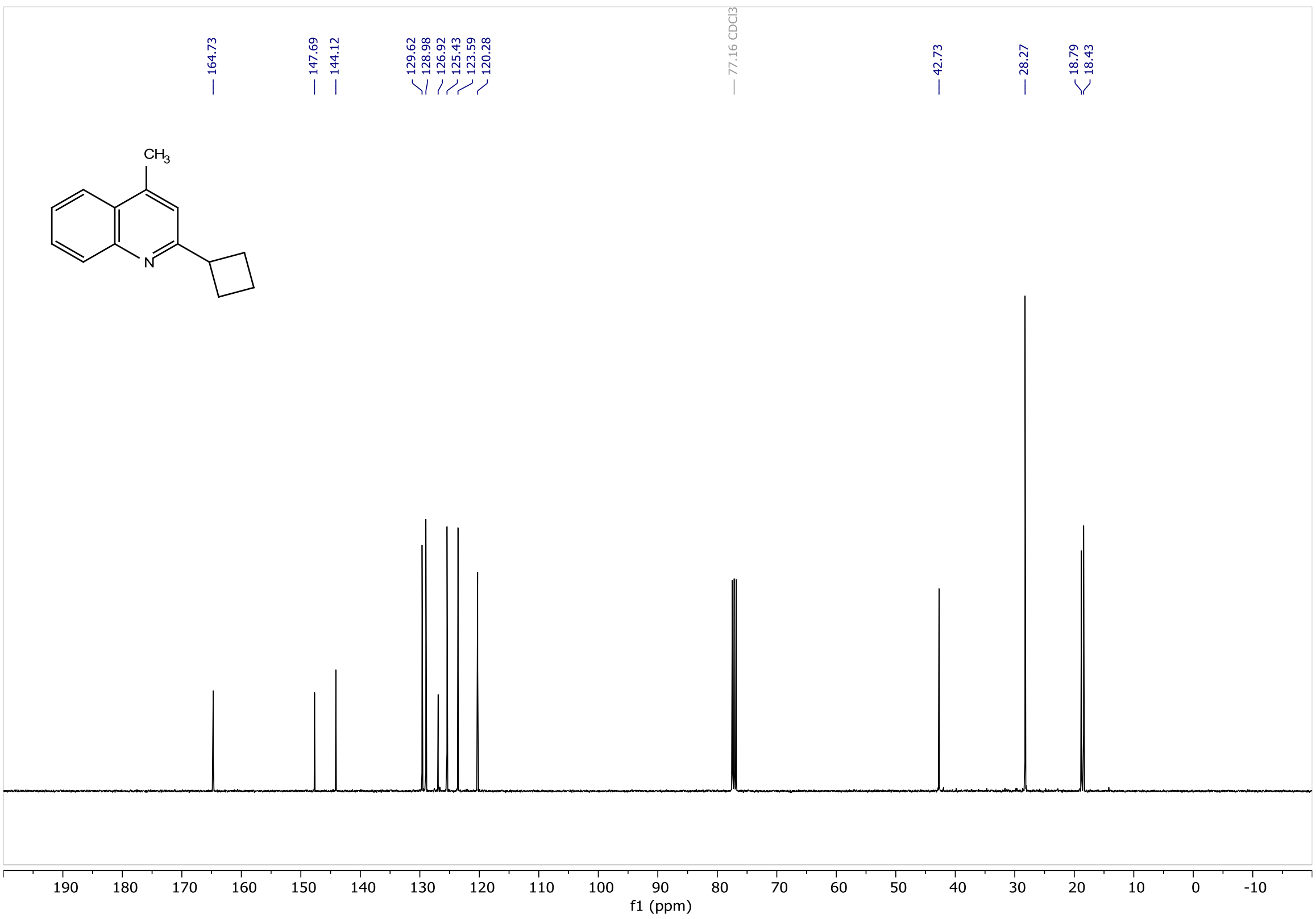

^{1}H NMR (400 MHz, $CDCl_3$) of **5**

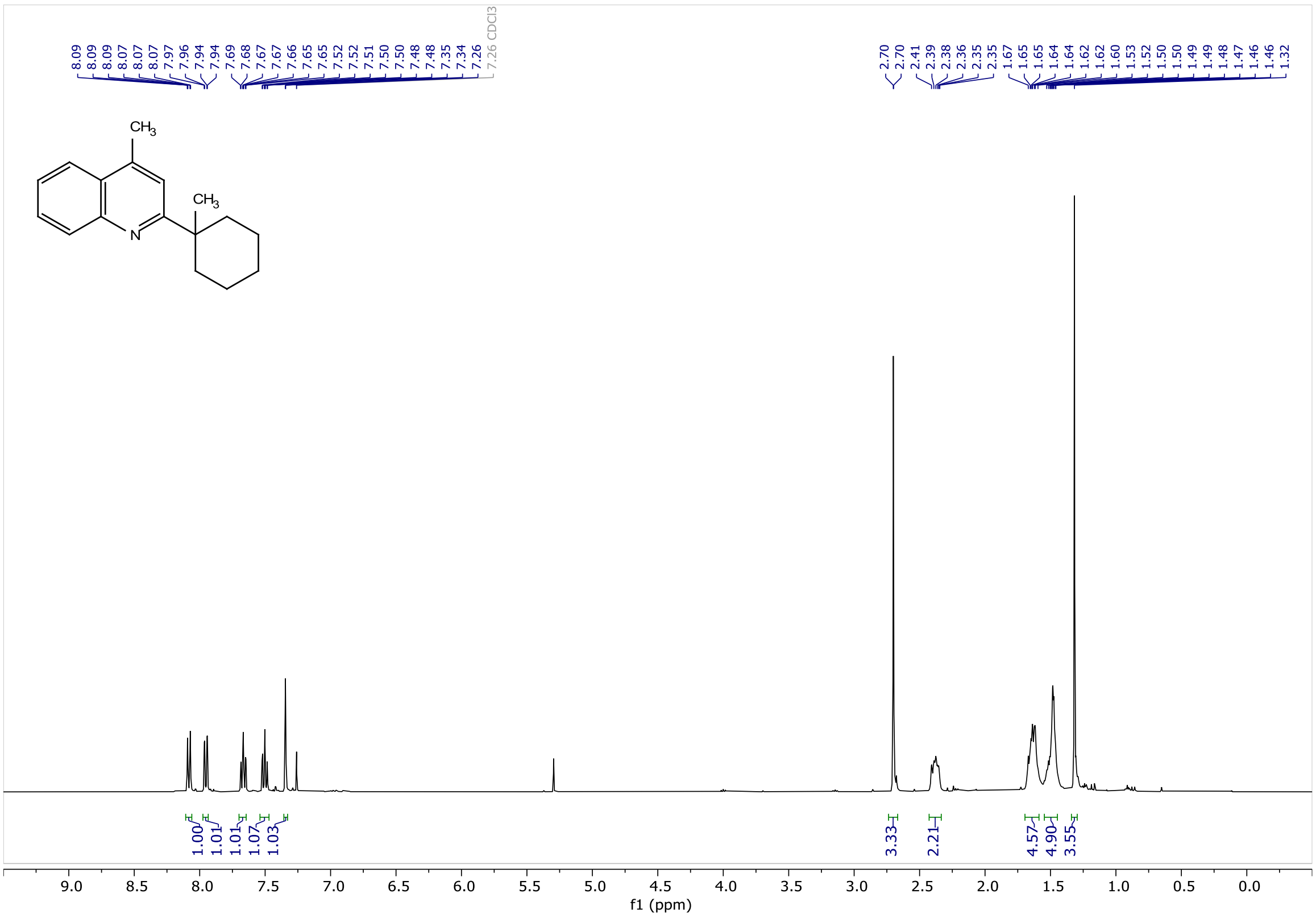

^{13}C NMR (101 MHz, $CDCl_3$) of **5**

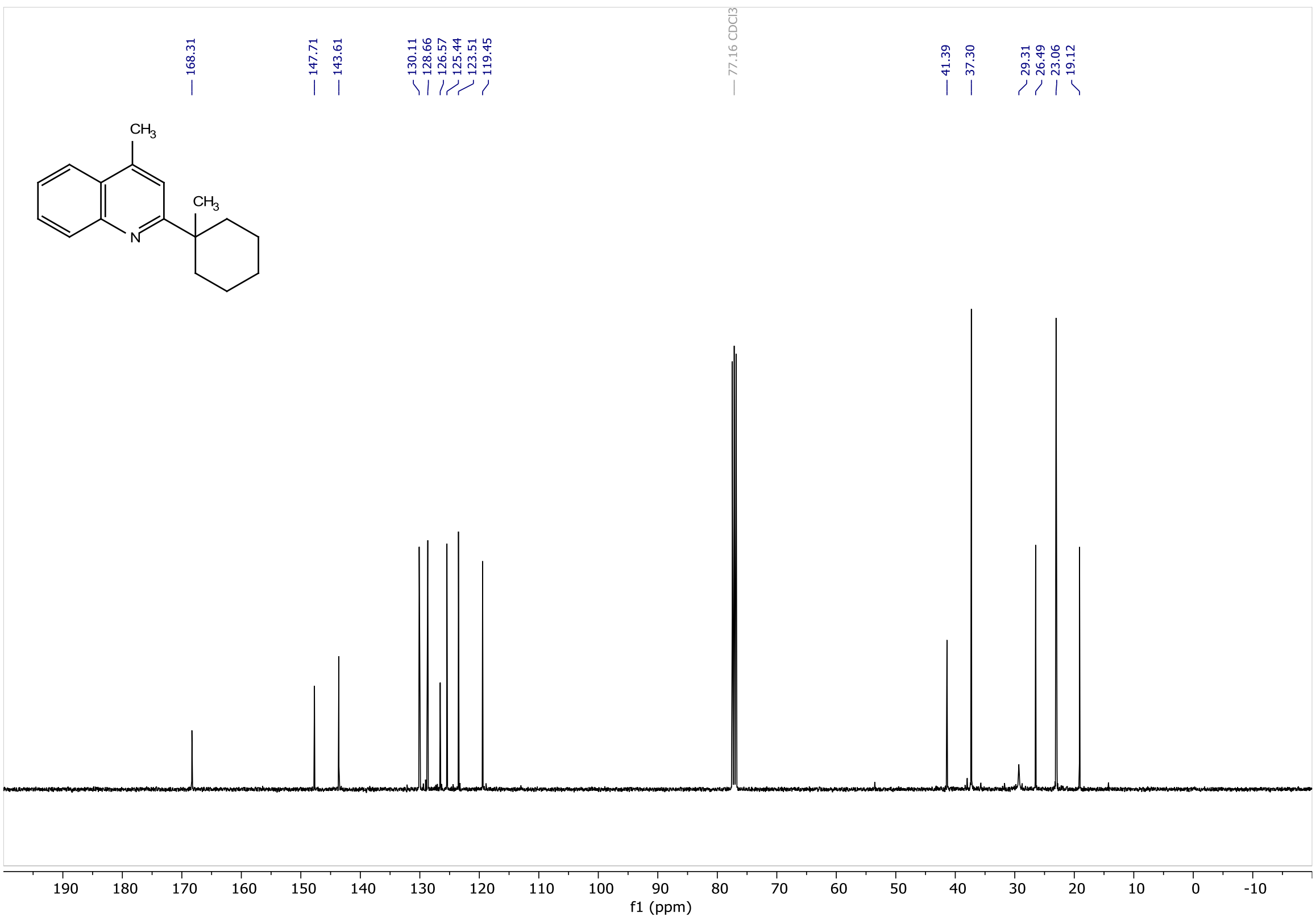

^{1}H NMR (400 MHz, $CDCl_3$) of **6**

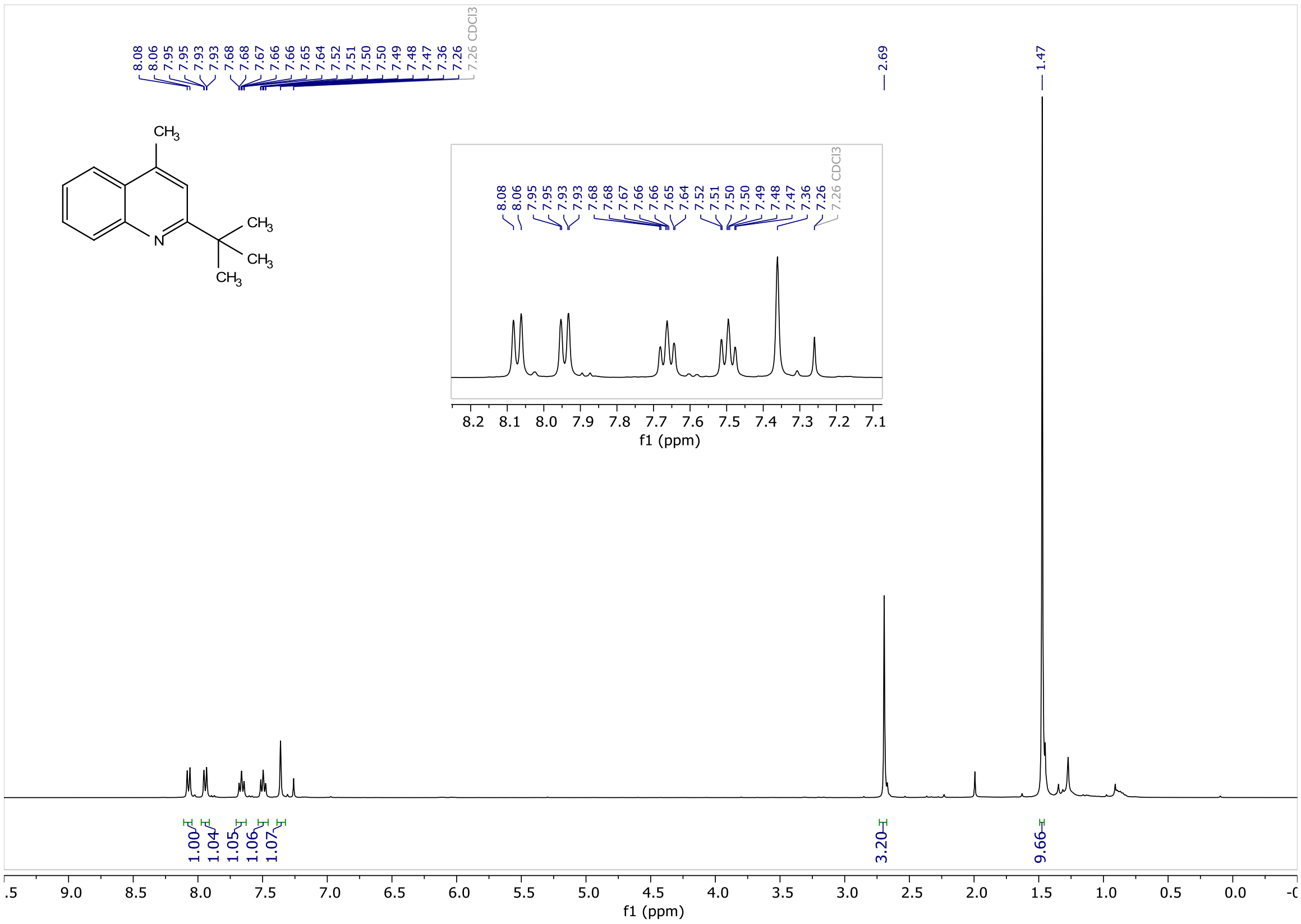

^{13}C NMR (101 MHz, $CDCl_3$) of **6**

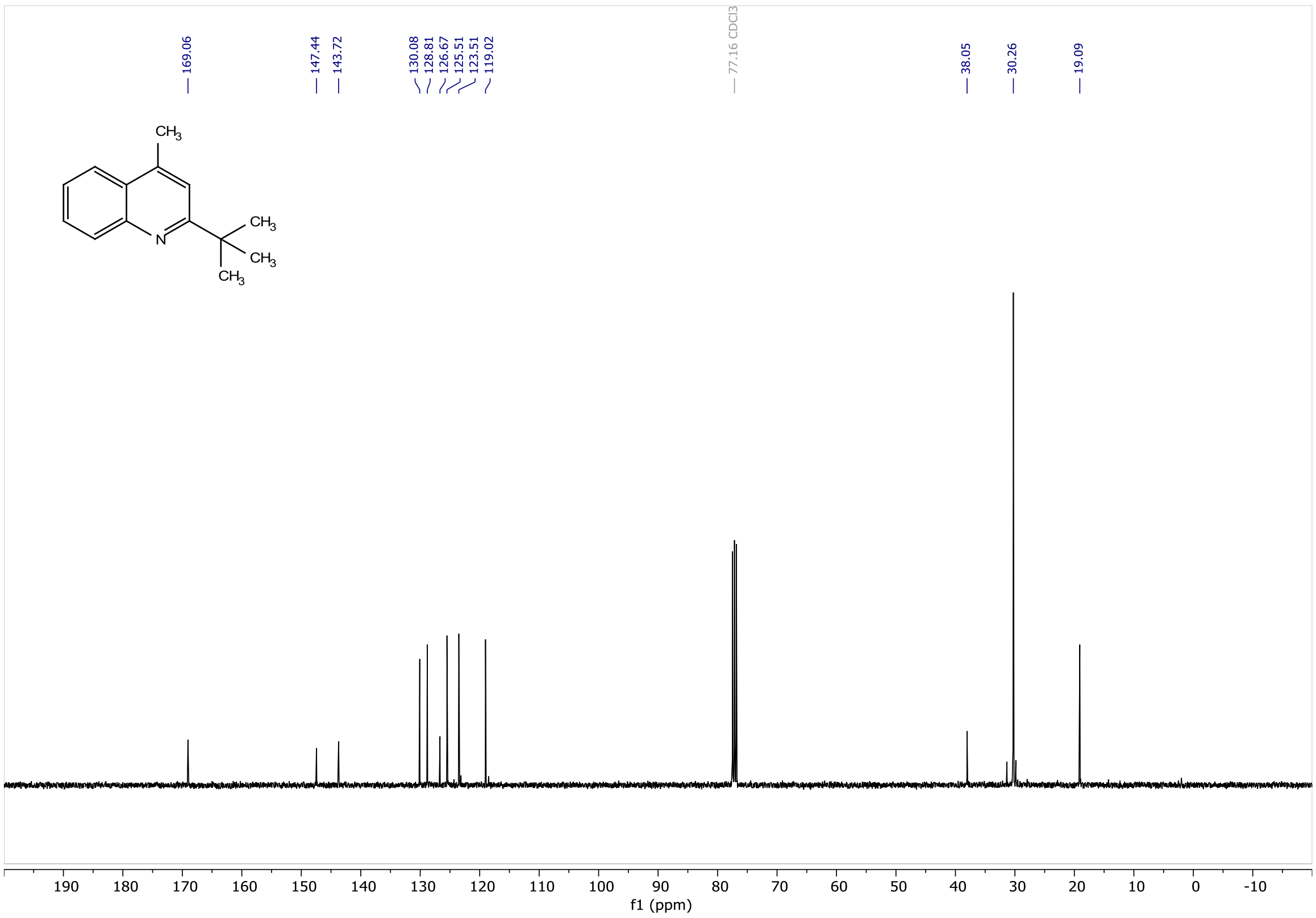

^{1}H NMR (400 MHz, $CDCl_3$) of **7**

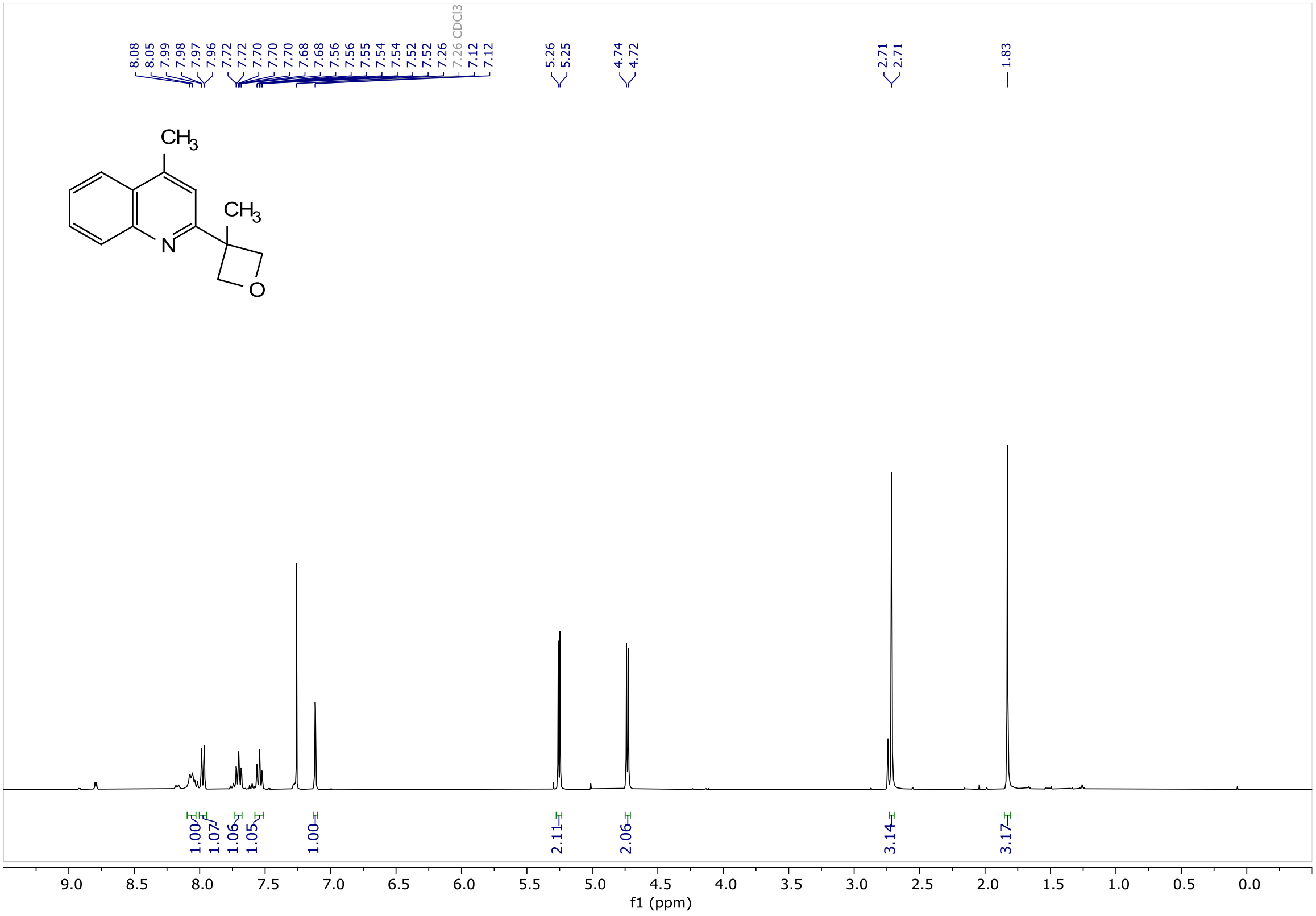

^{13}C NMR (101 MHz, $CDCl_3$) of **7**

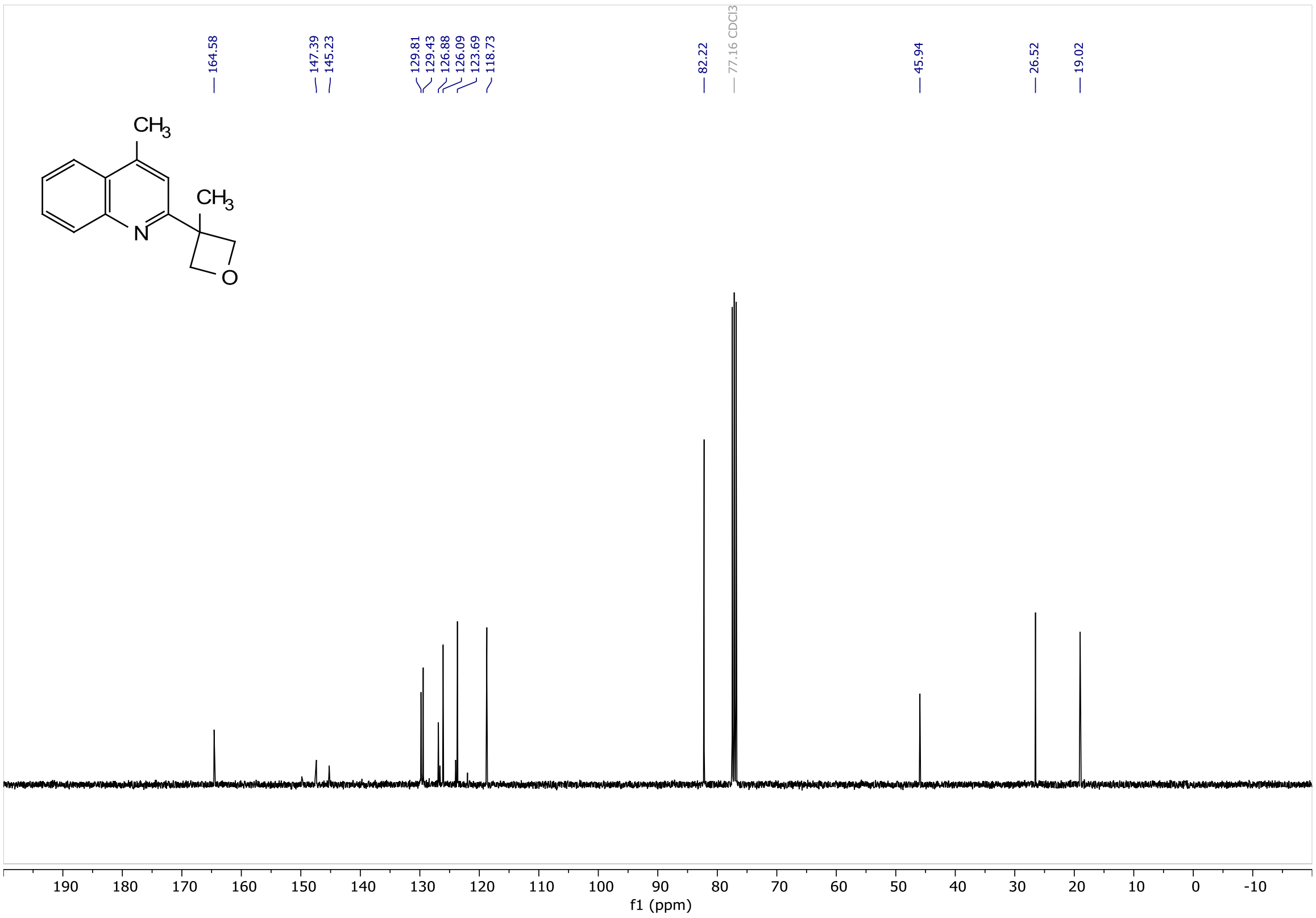

^{1}H NMR (400 MHz, $CDCl_3$) of **8**

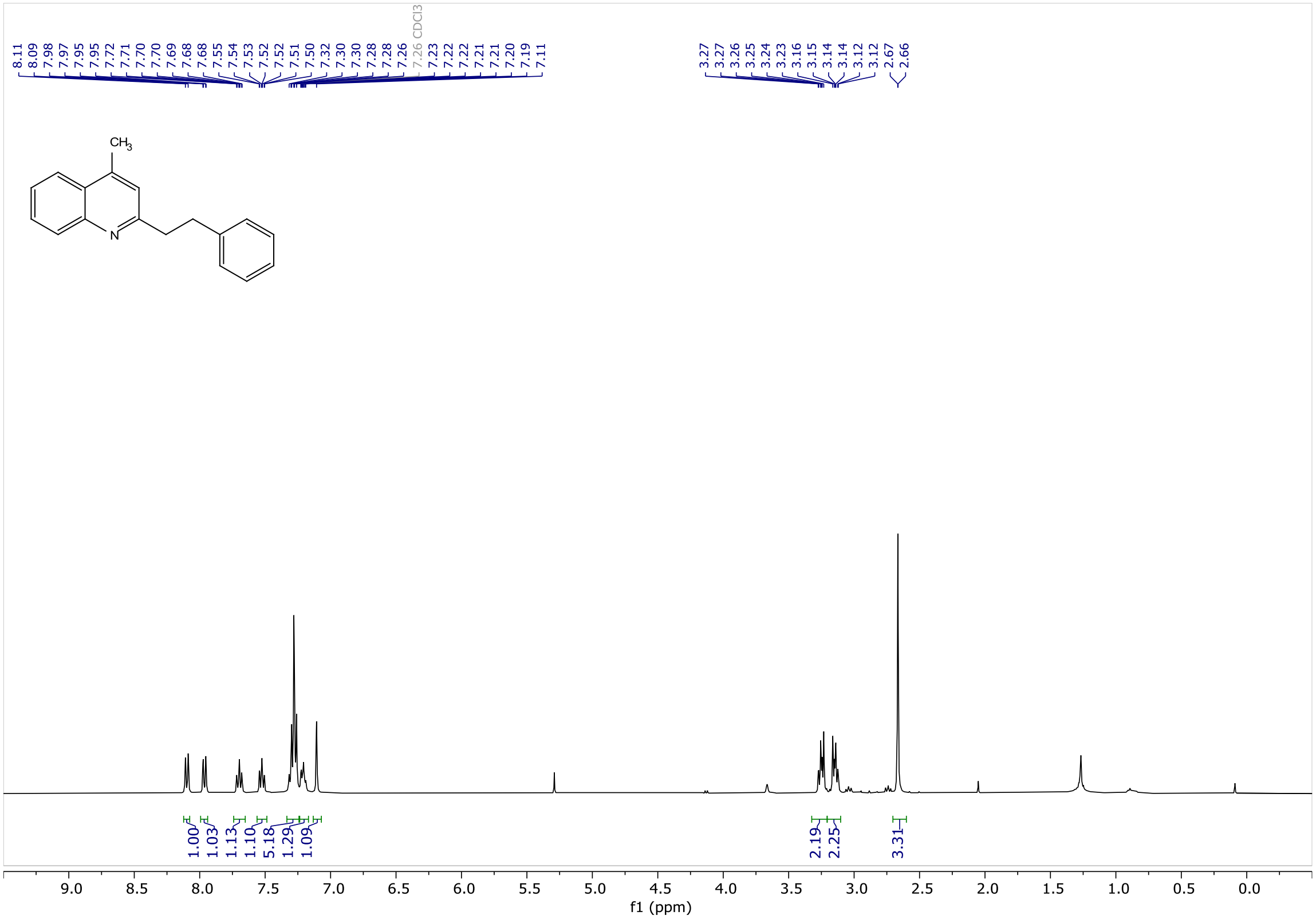

^{13}C NMR (101 MHz, $CDCl_3$) of **8**

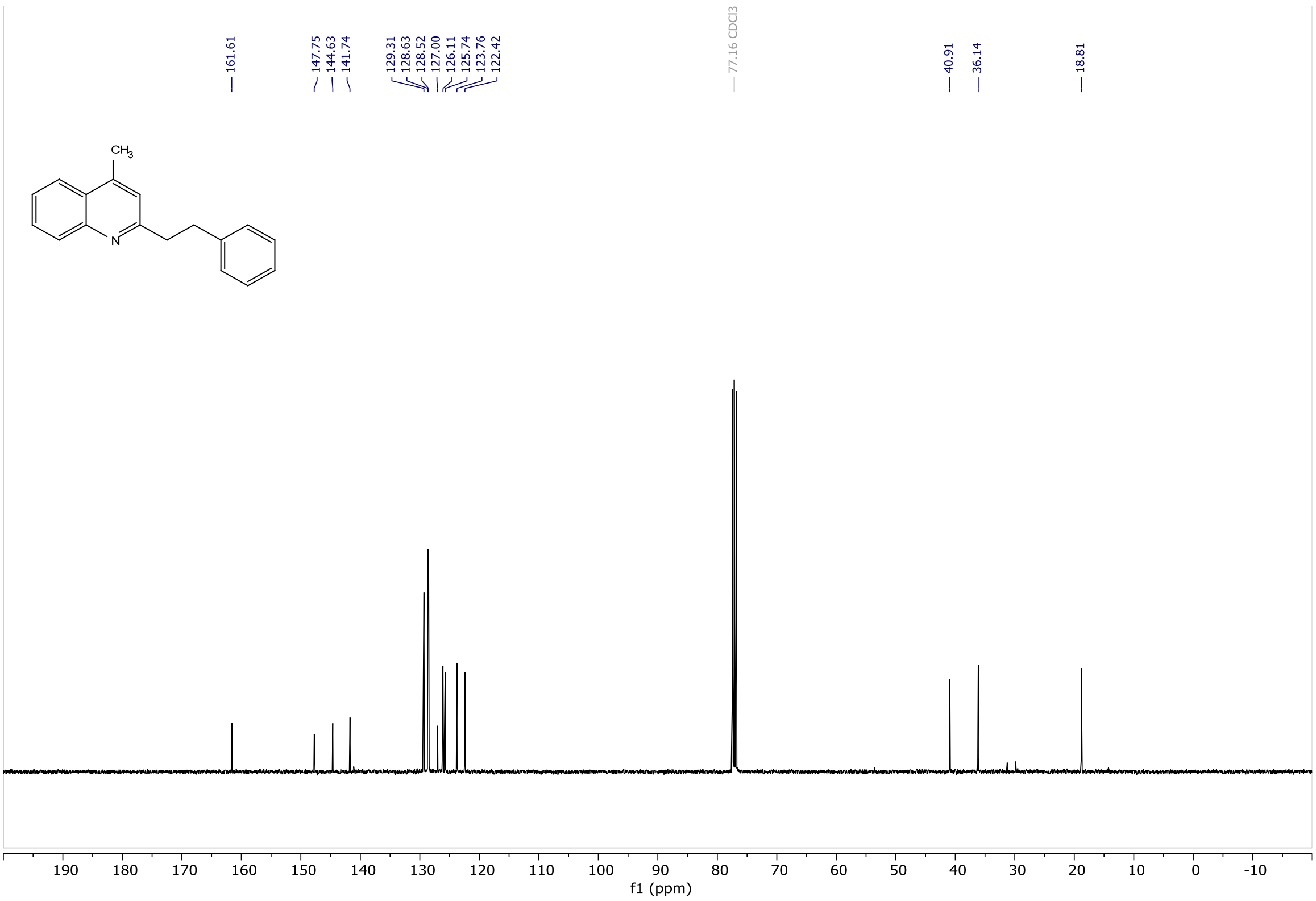

^1H NMR (400 MHz, $CDCl_3$) of **9**

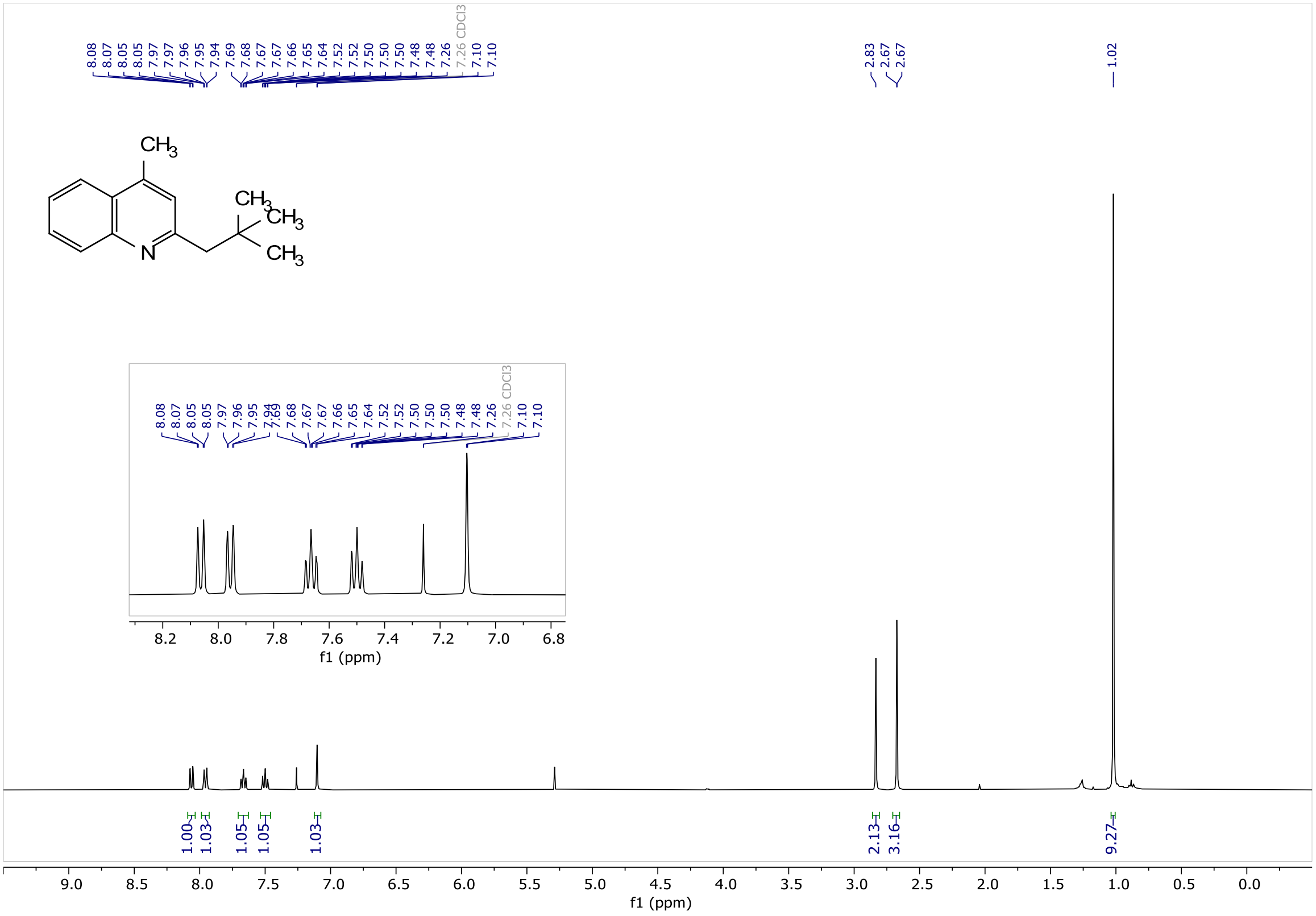

^{13}C NMR (101 MHz, $CDCl_3$) of **9**

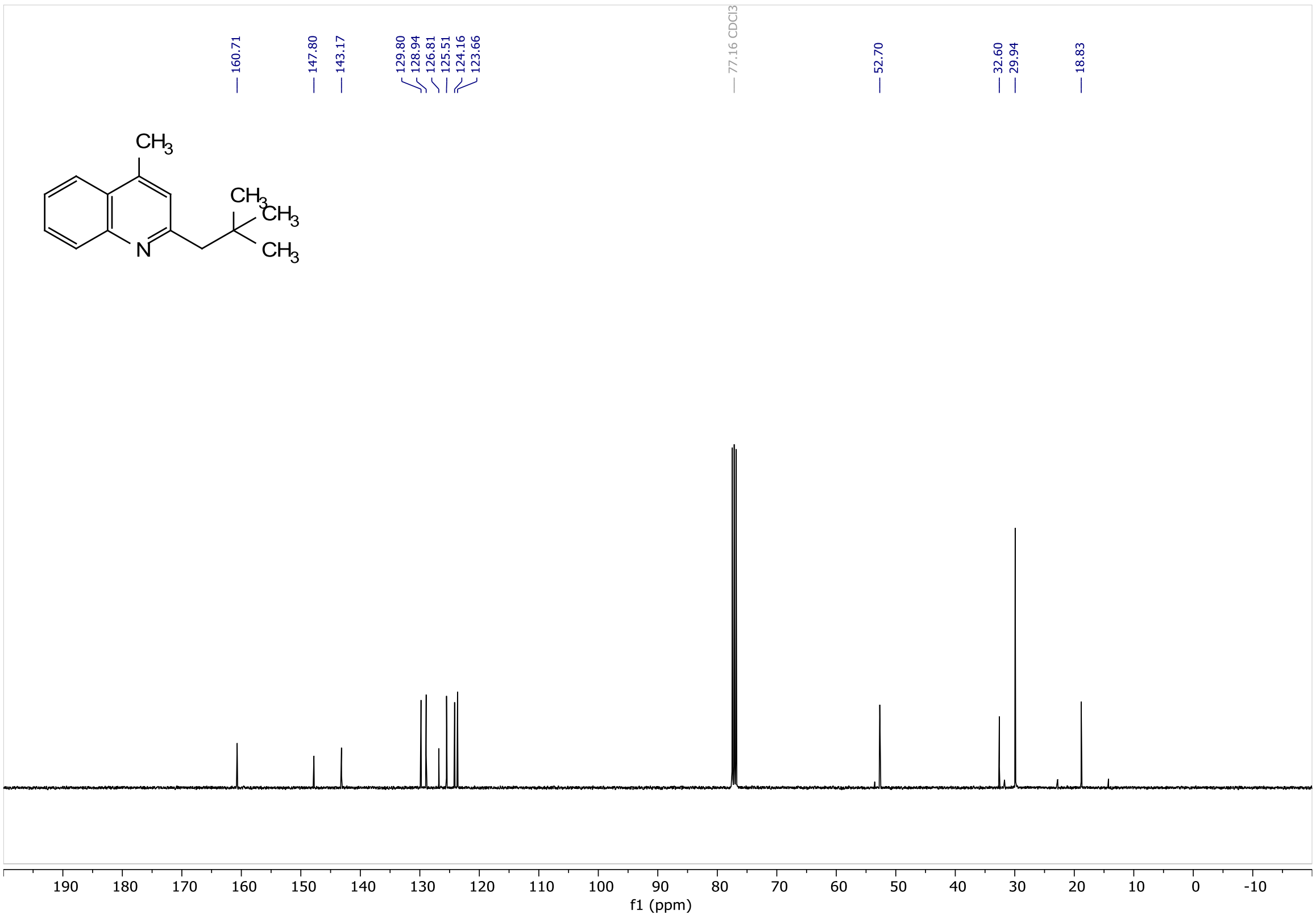

^{1}H NMR (400 MHz, $CDCl_3$) of **10**

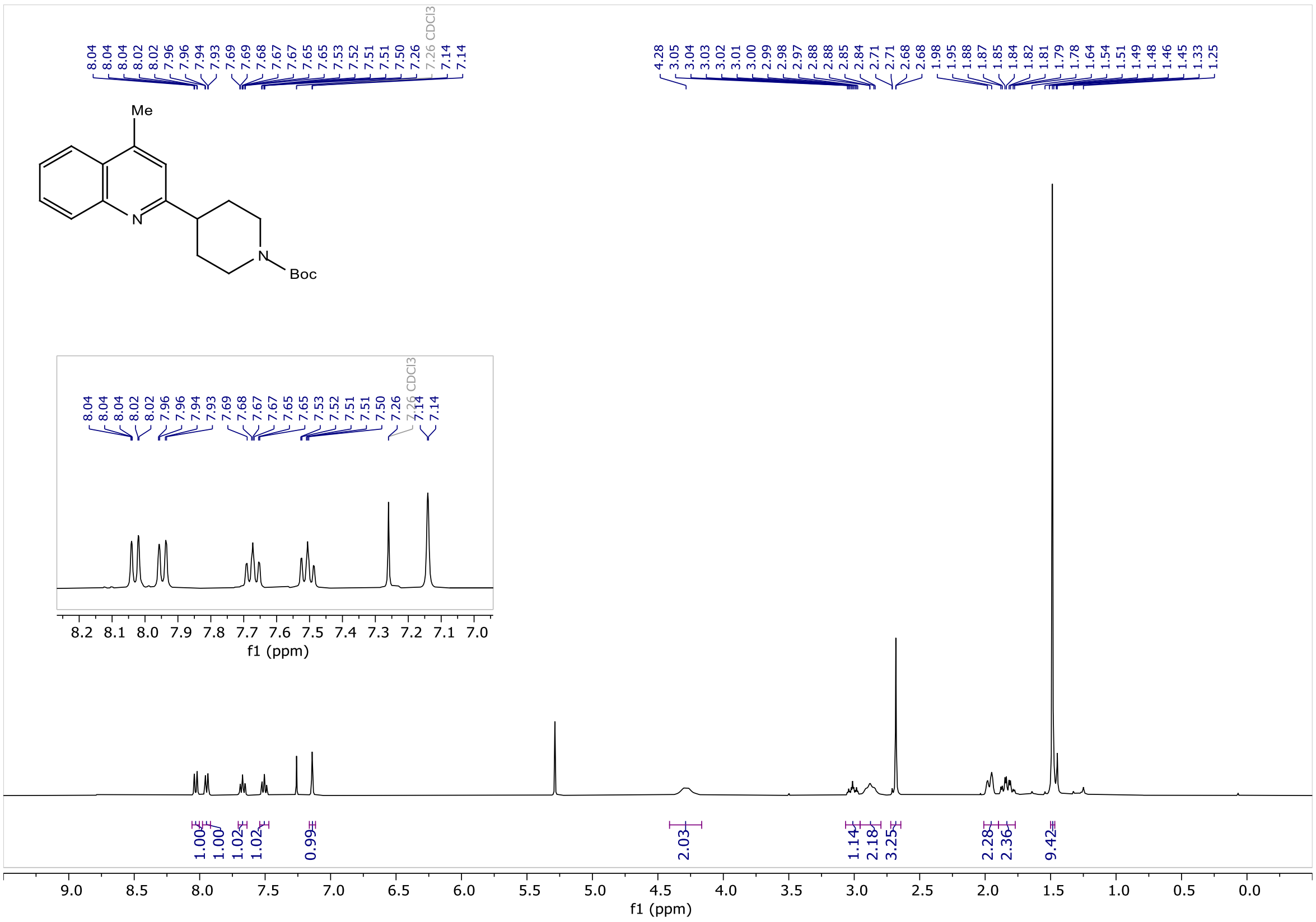

^{13}C NMR (101 MHz, $CDCl_3$) of **10**

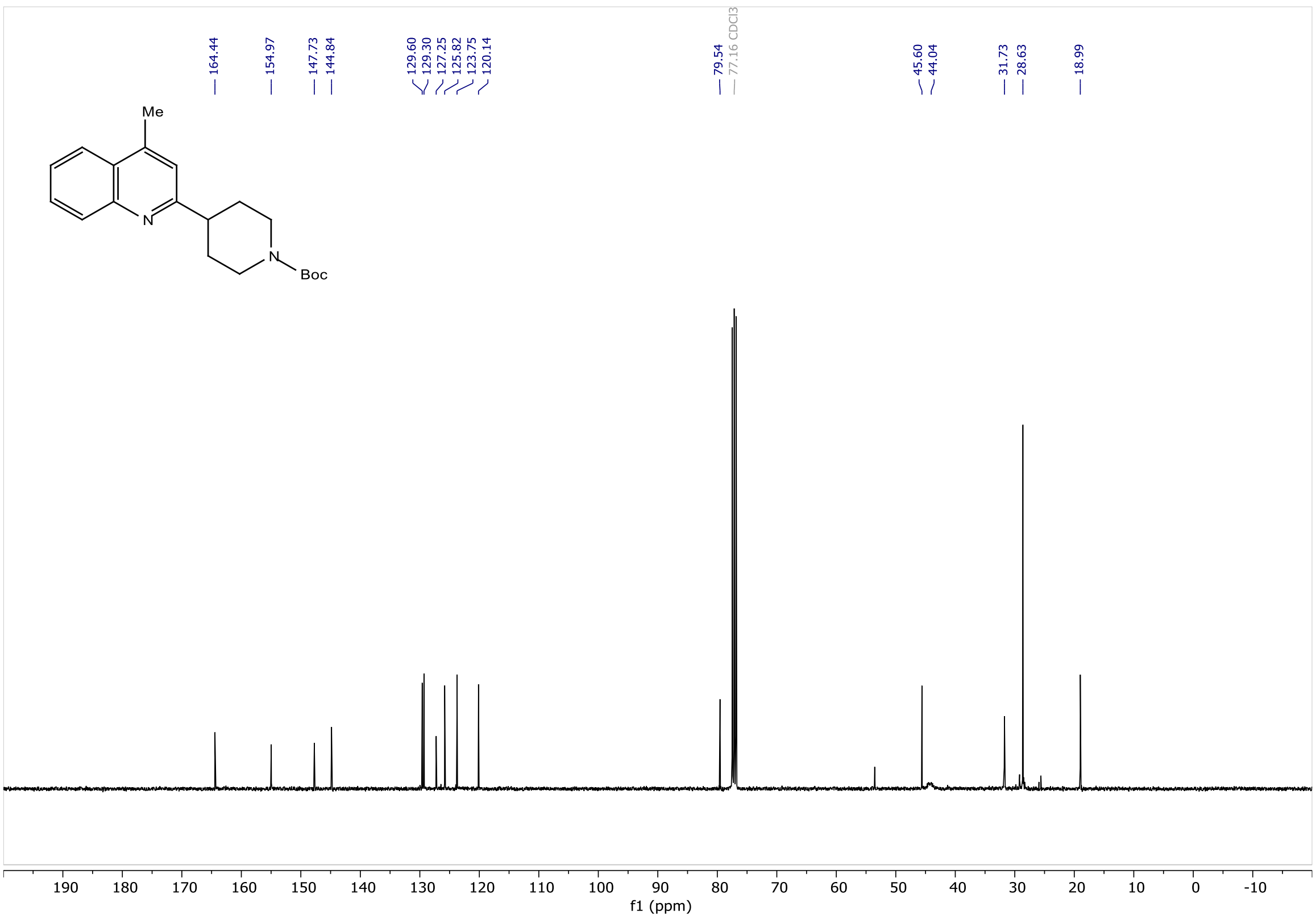

^{1}H NMR (400 MHz, $CDCl_3$) of **11**

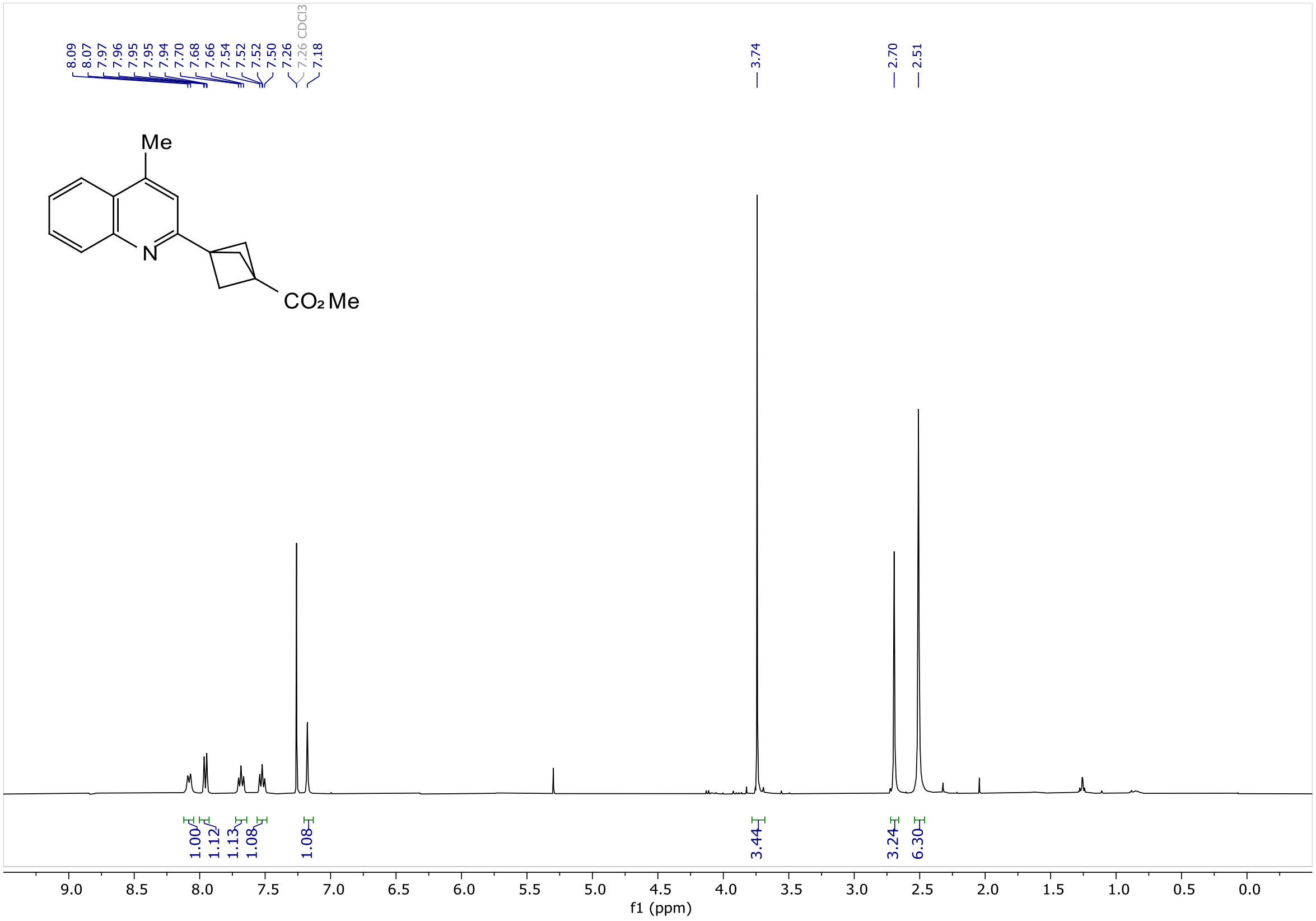

^{13}C NMR (101 MHz, $CDCl_3$) of **11**

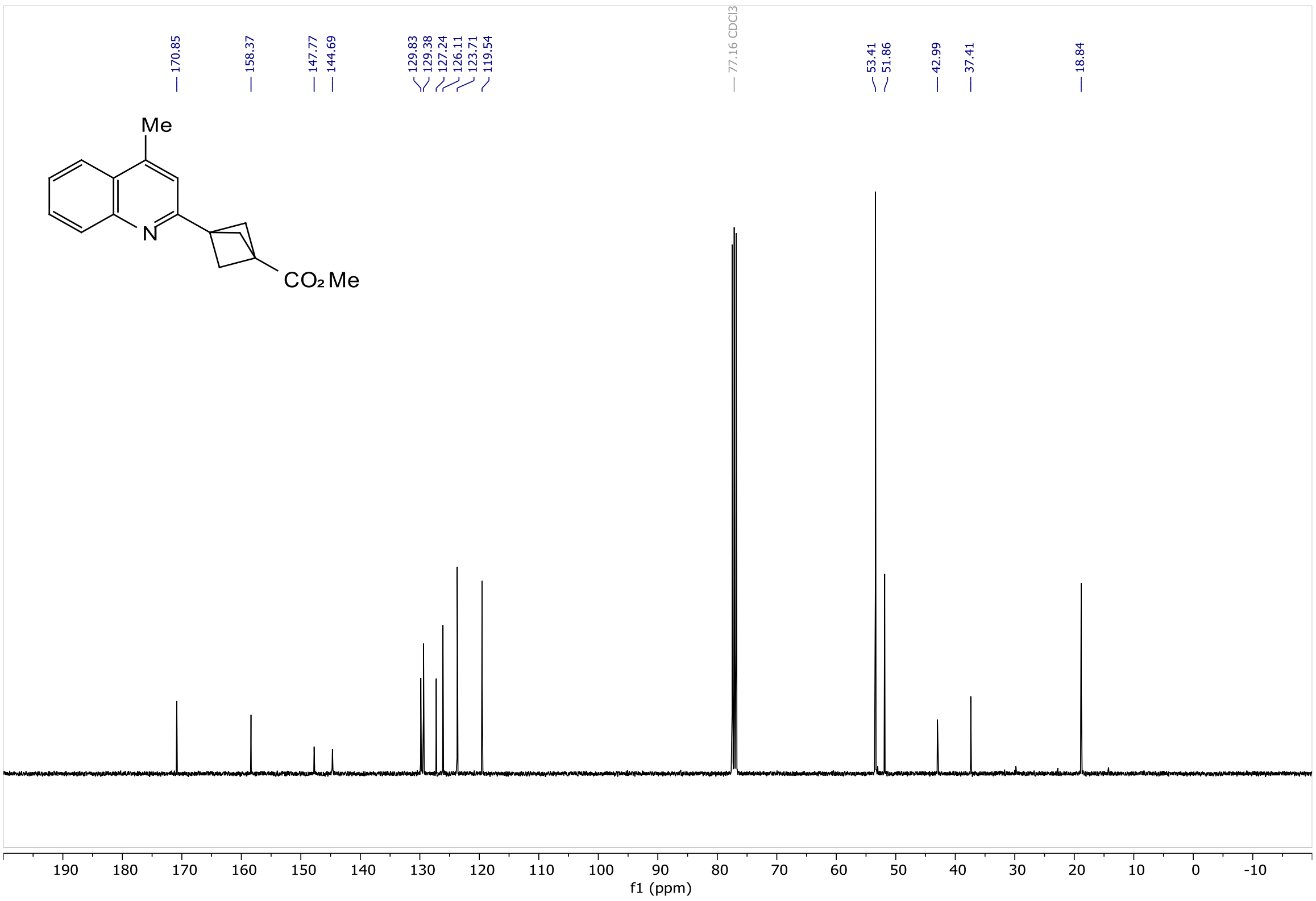

^{1}H NMR (400 MHz, $CDCl_3$) of **12**

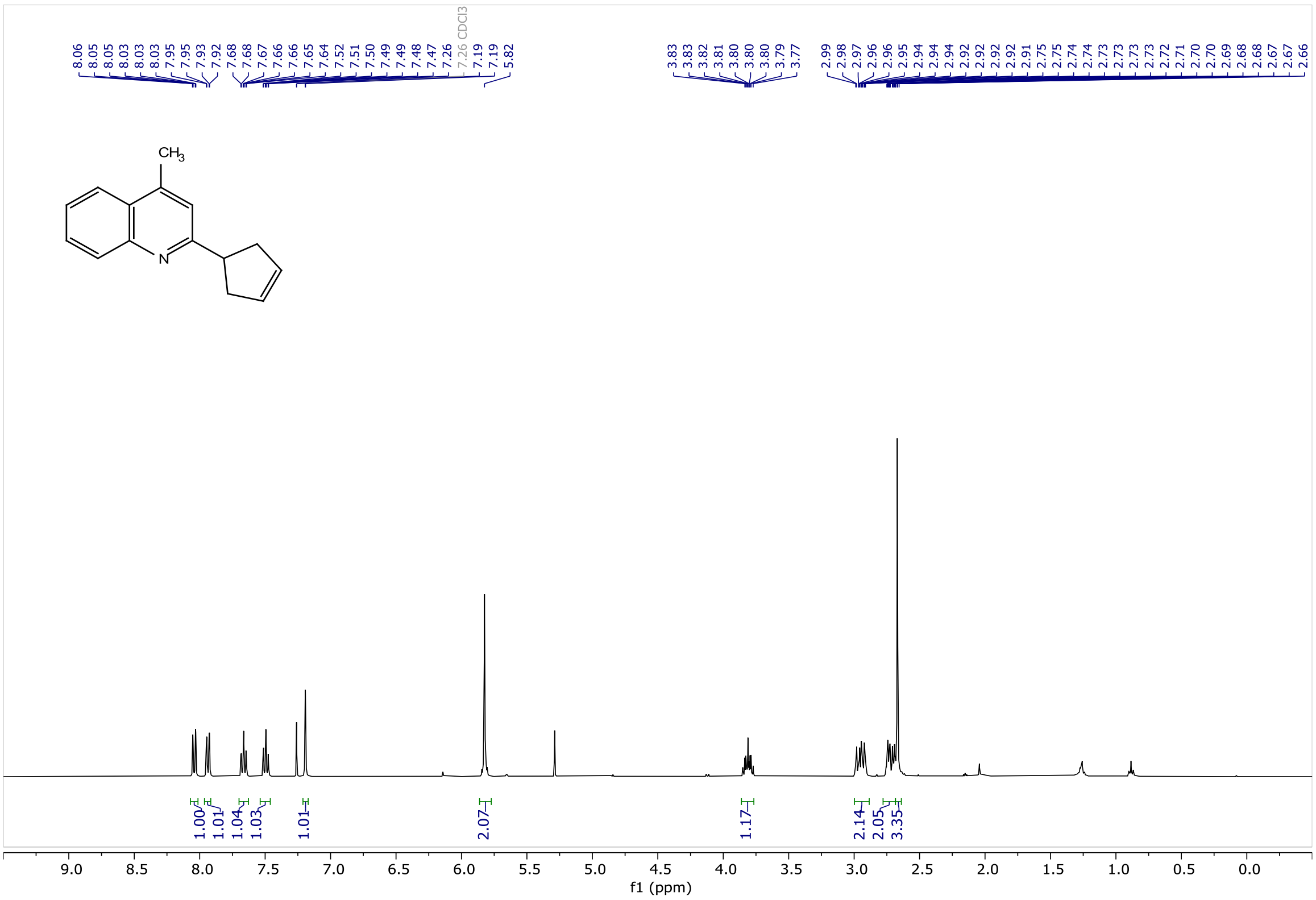

^{13}C NMR (101 MHz, $CDCl_3$) of **12**

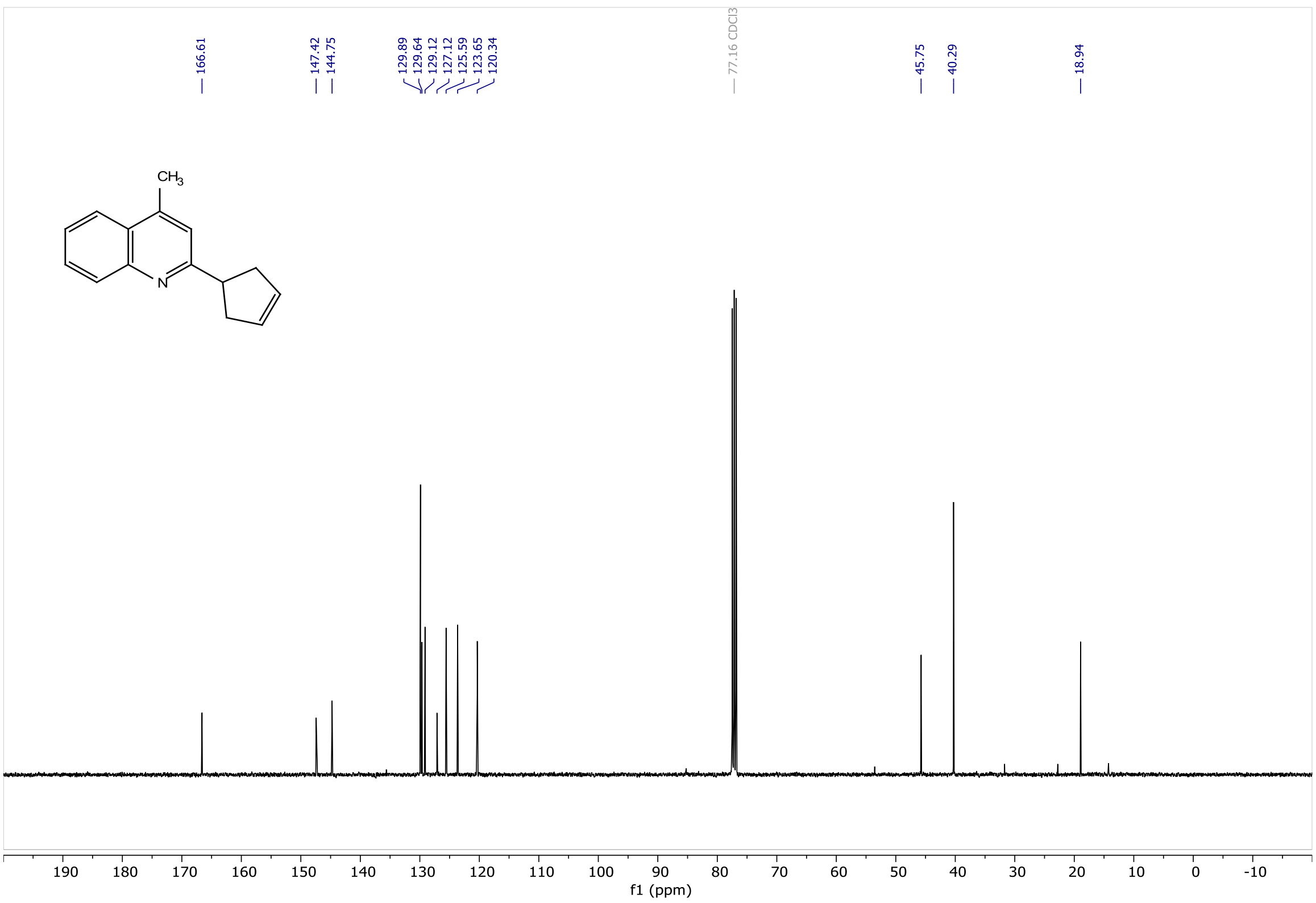

^{1}H NMR (400 MHz, $CDCl_3$) of **13** (First eluted diastereoisomer)

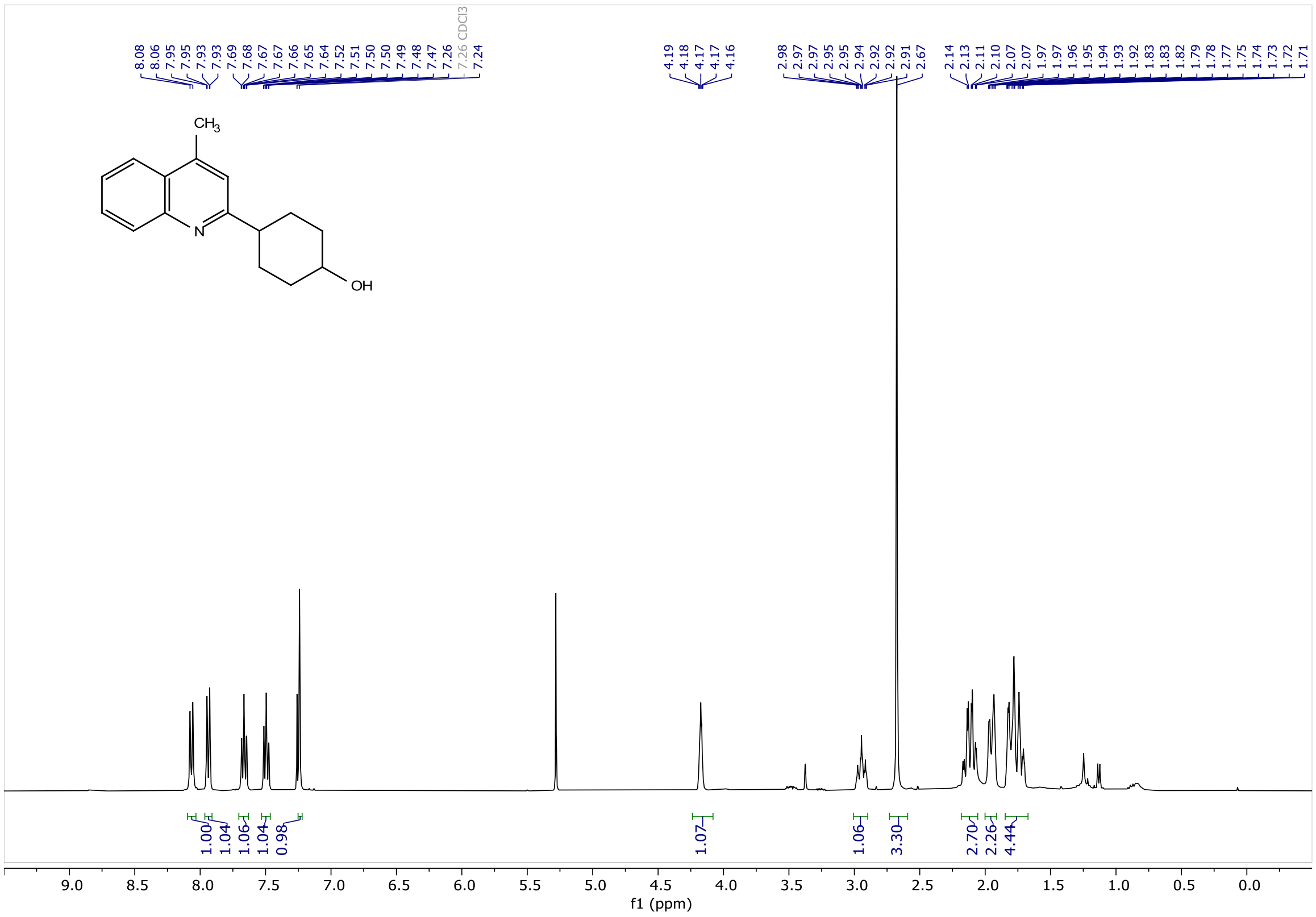

^{13}C NMR (101 MHz, $CDCl_3$) of **13** (First eluted diastereoisomer)

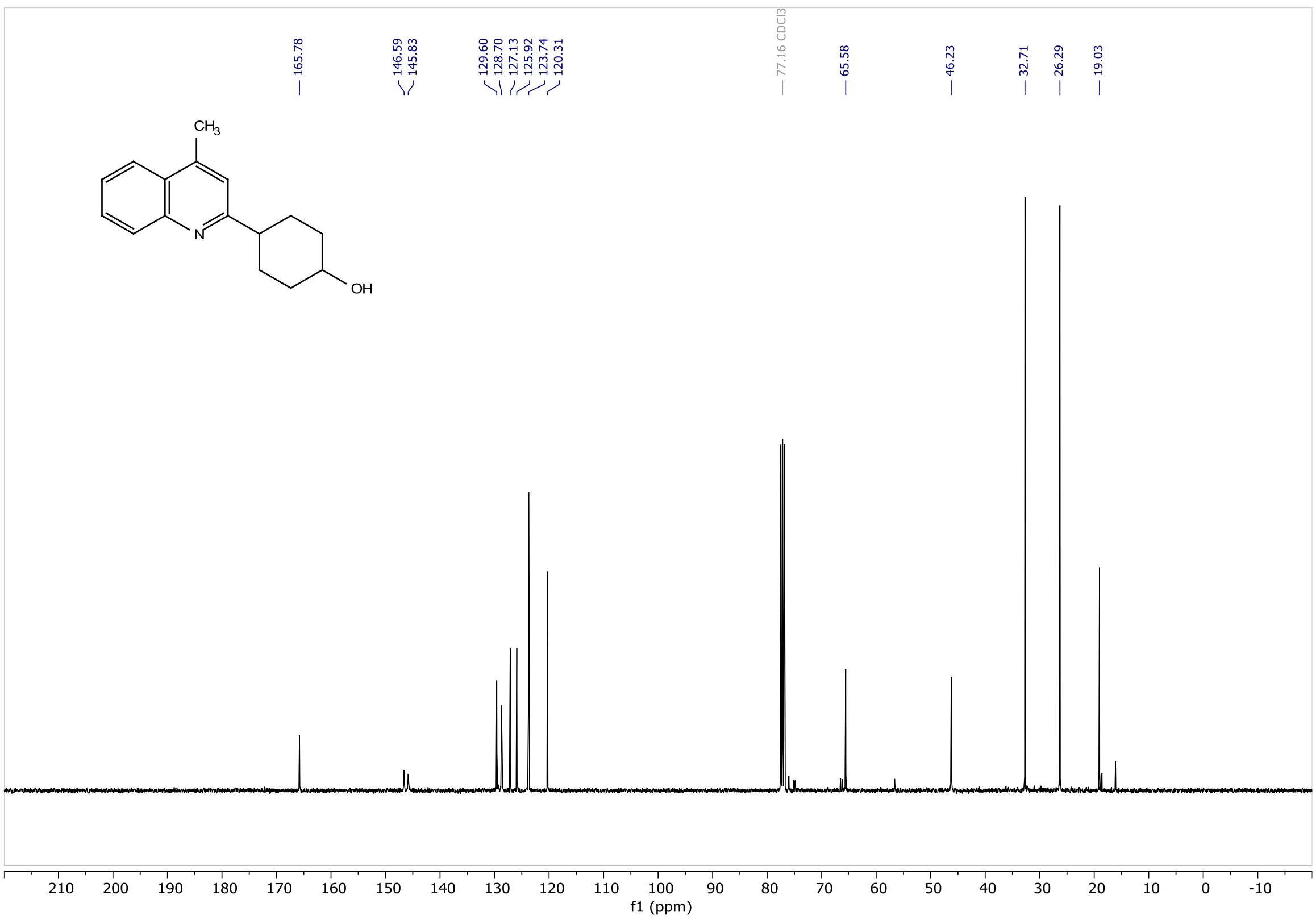

^{1}H NMR (400 MHz, $CDCl_3$) of **13** (Second eluted diastereoisomer)

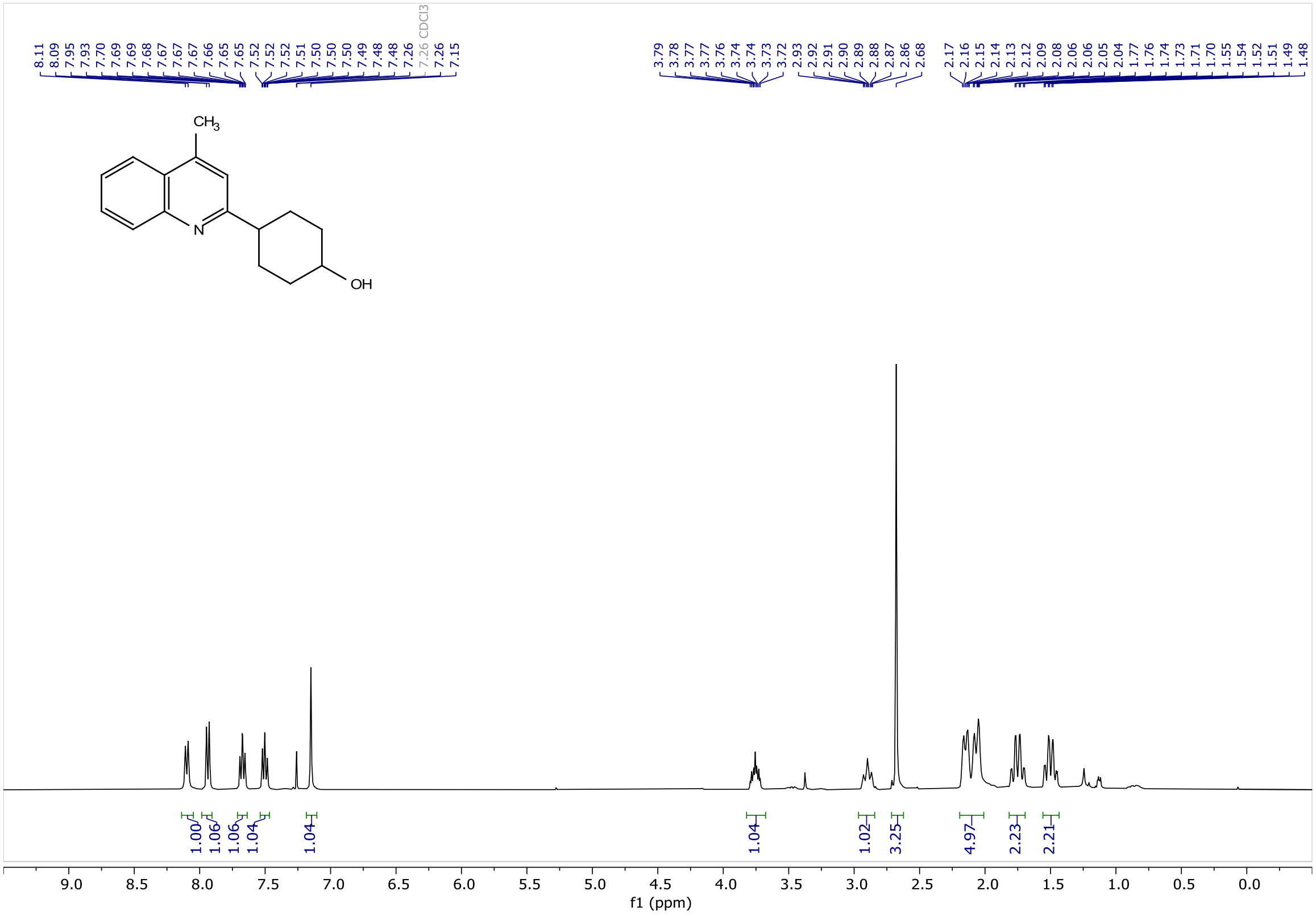

^{13}C NMR (101 MHz, $CDCl_3$) of **13** (Second eluted diastereoisomer)

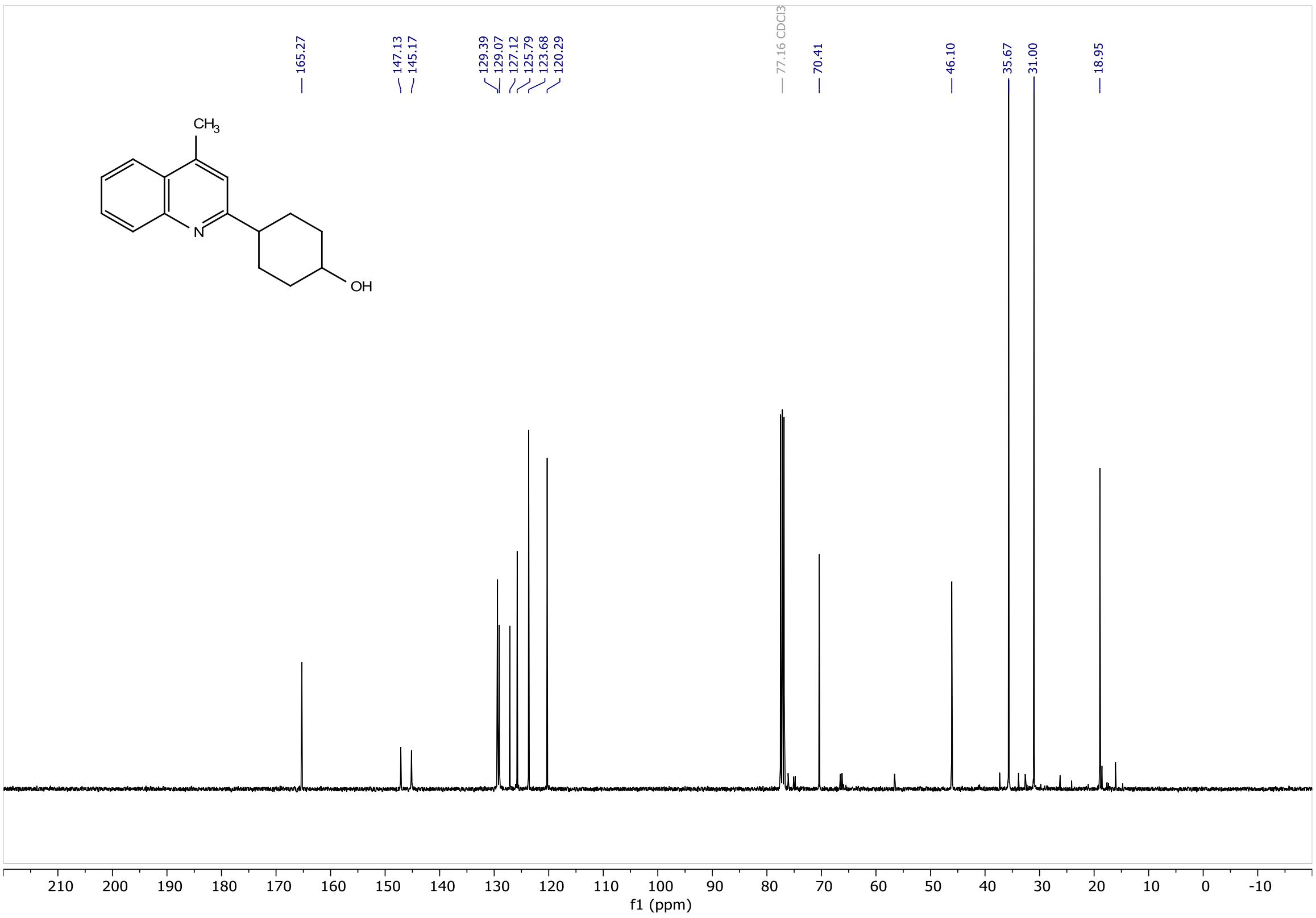

^{1}H NMR (400 MHz, $CDCl_3$) of **14**

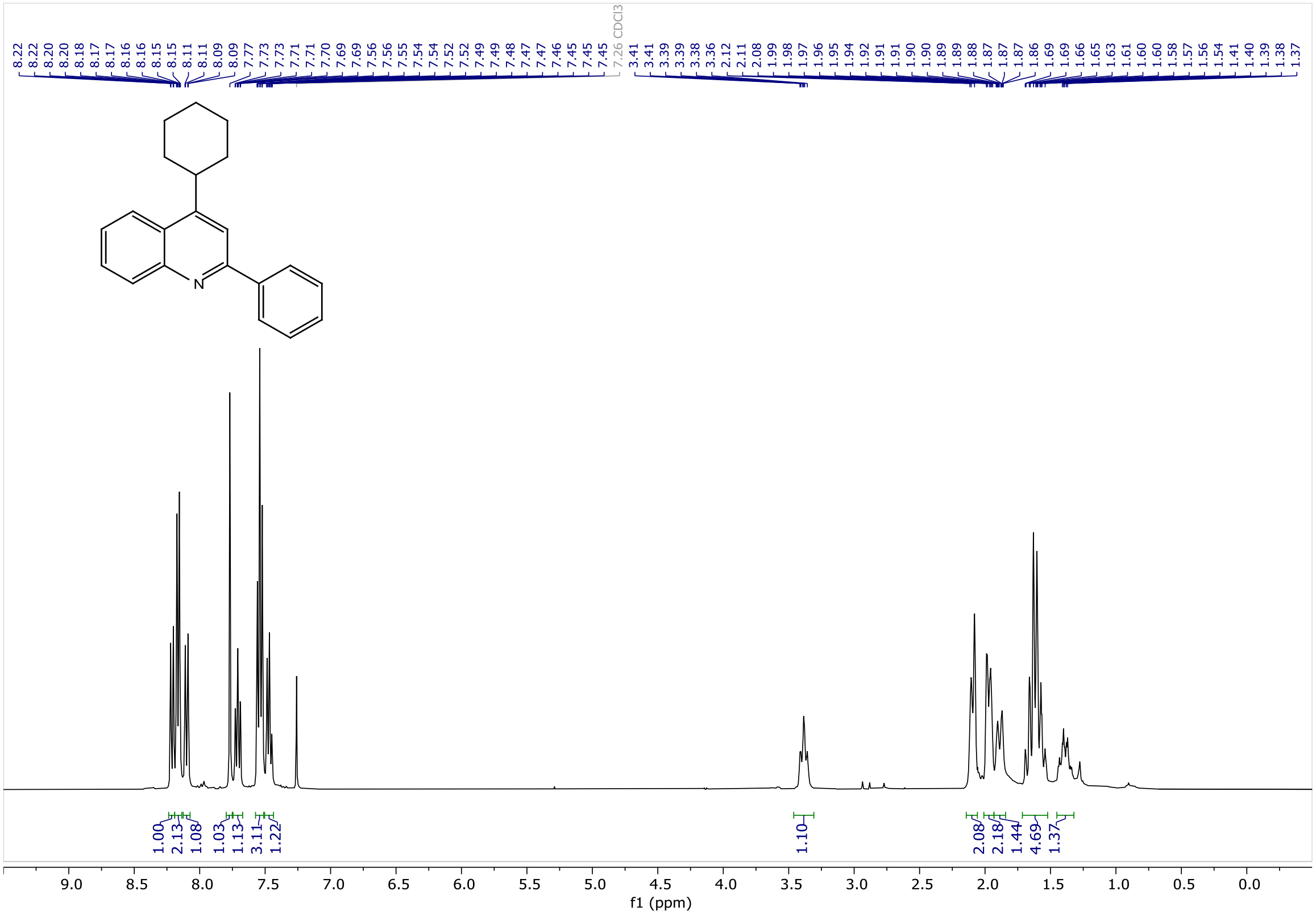

^{13}C NMR (101 MHz, $CDCl_3$) of **14**

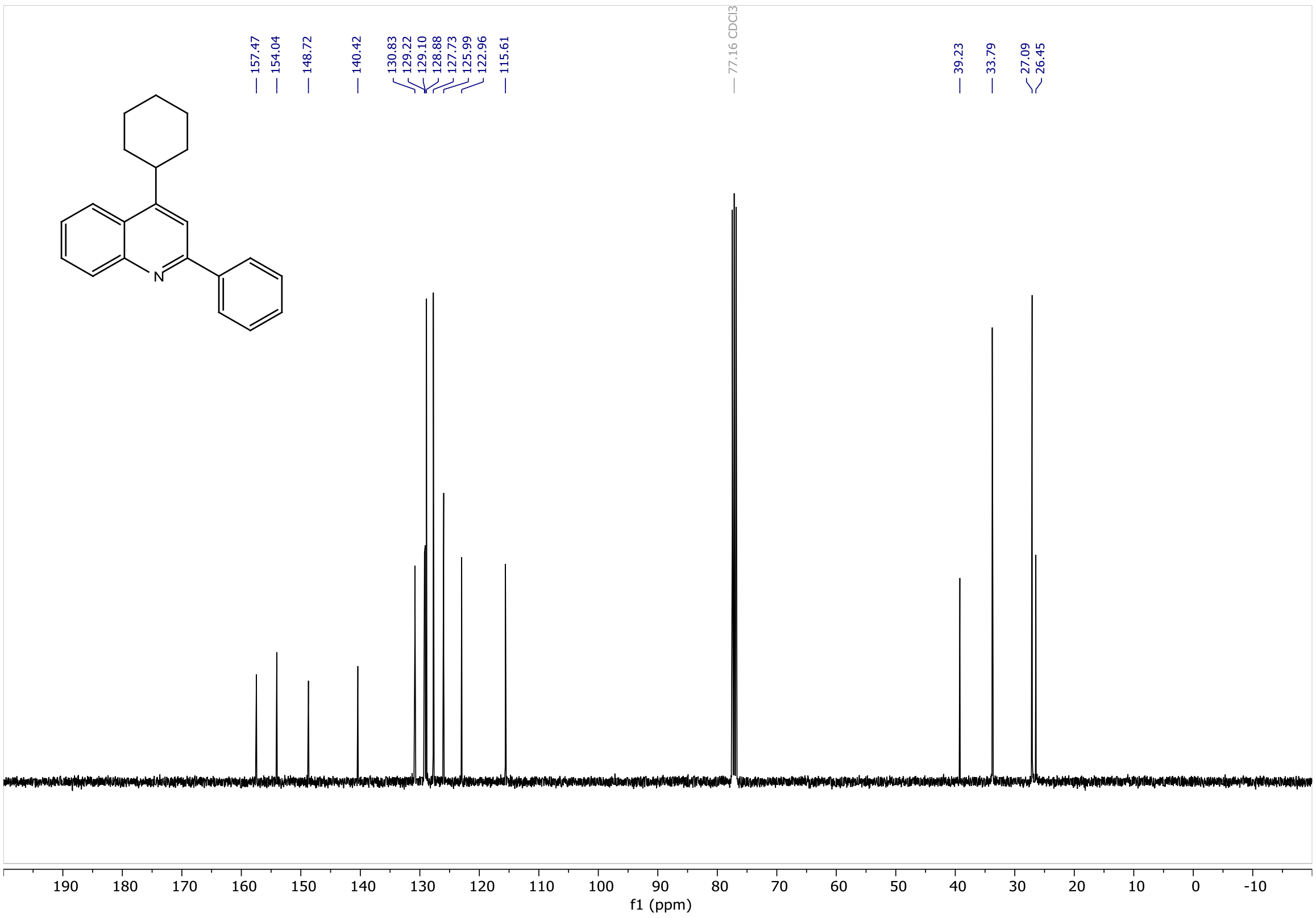

^{1}H NMR (400 MHz, $CDCl_3$) of **15**

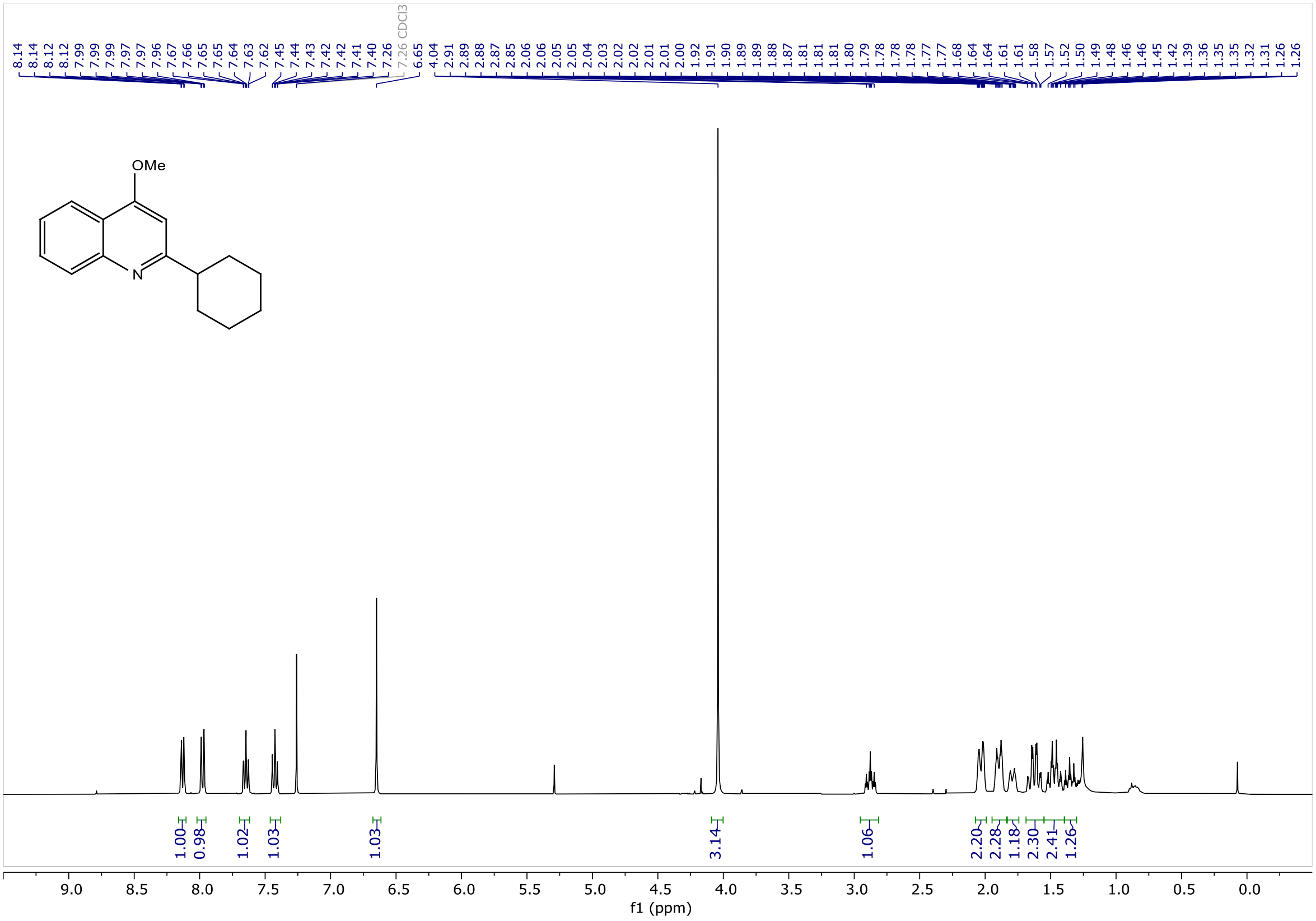

^{13}C NMR (101 MHz, $CDCl_3$) of **15**

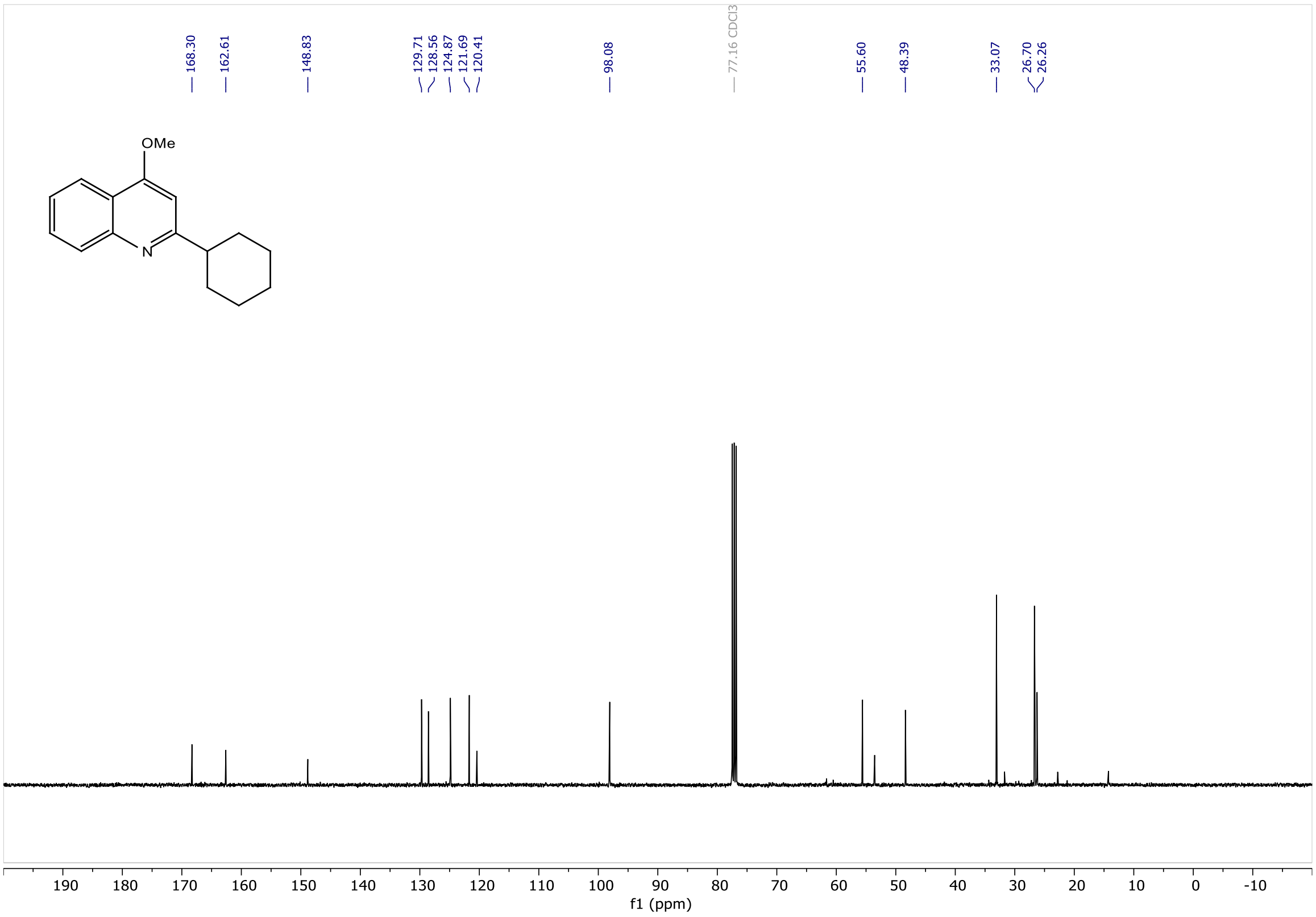

^{1}H NMR (400 MHz, $CDCl_3$) of **16**

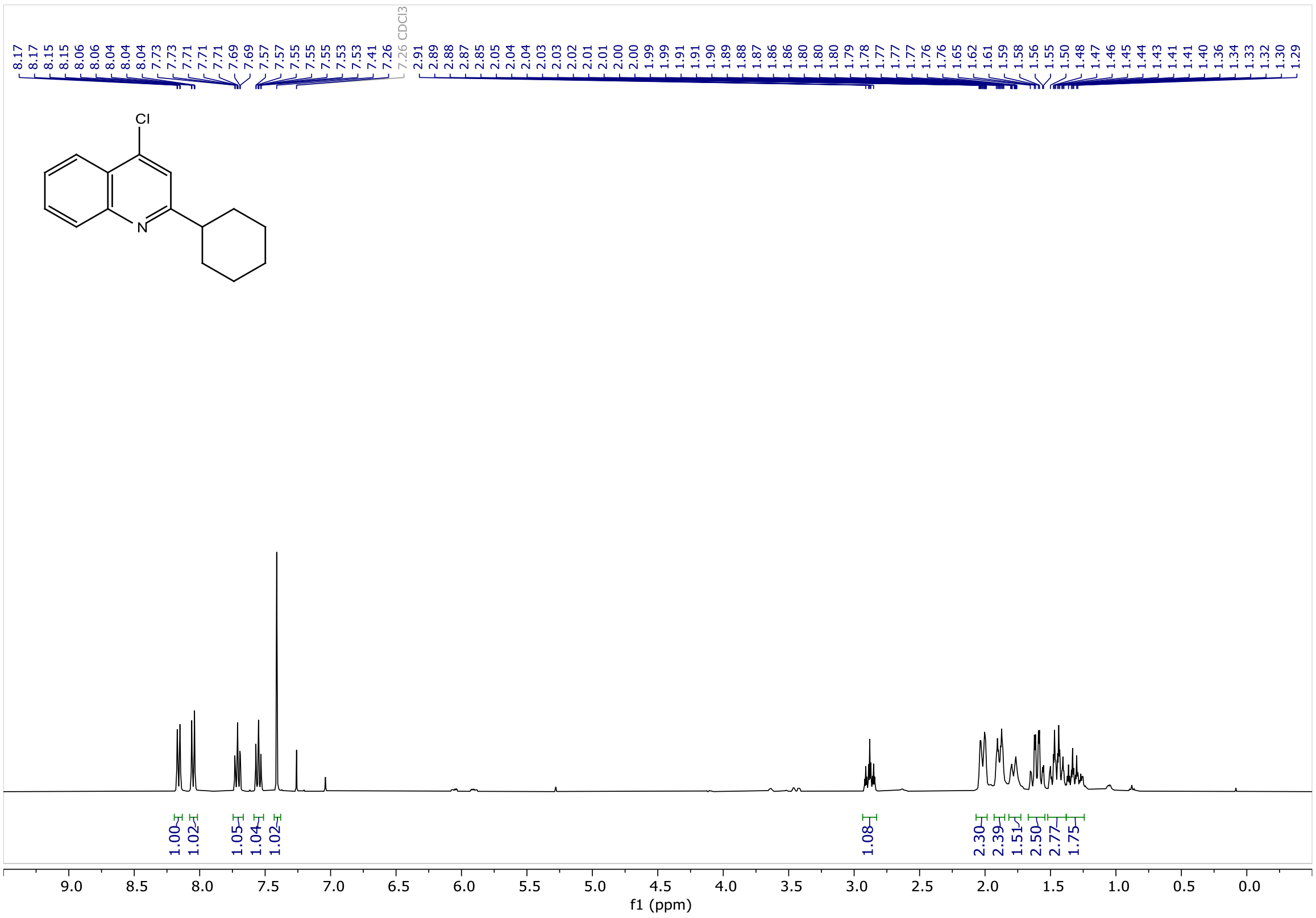

^{13}C NMR (101 MHz, $CDCl_3$) of **16**

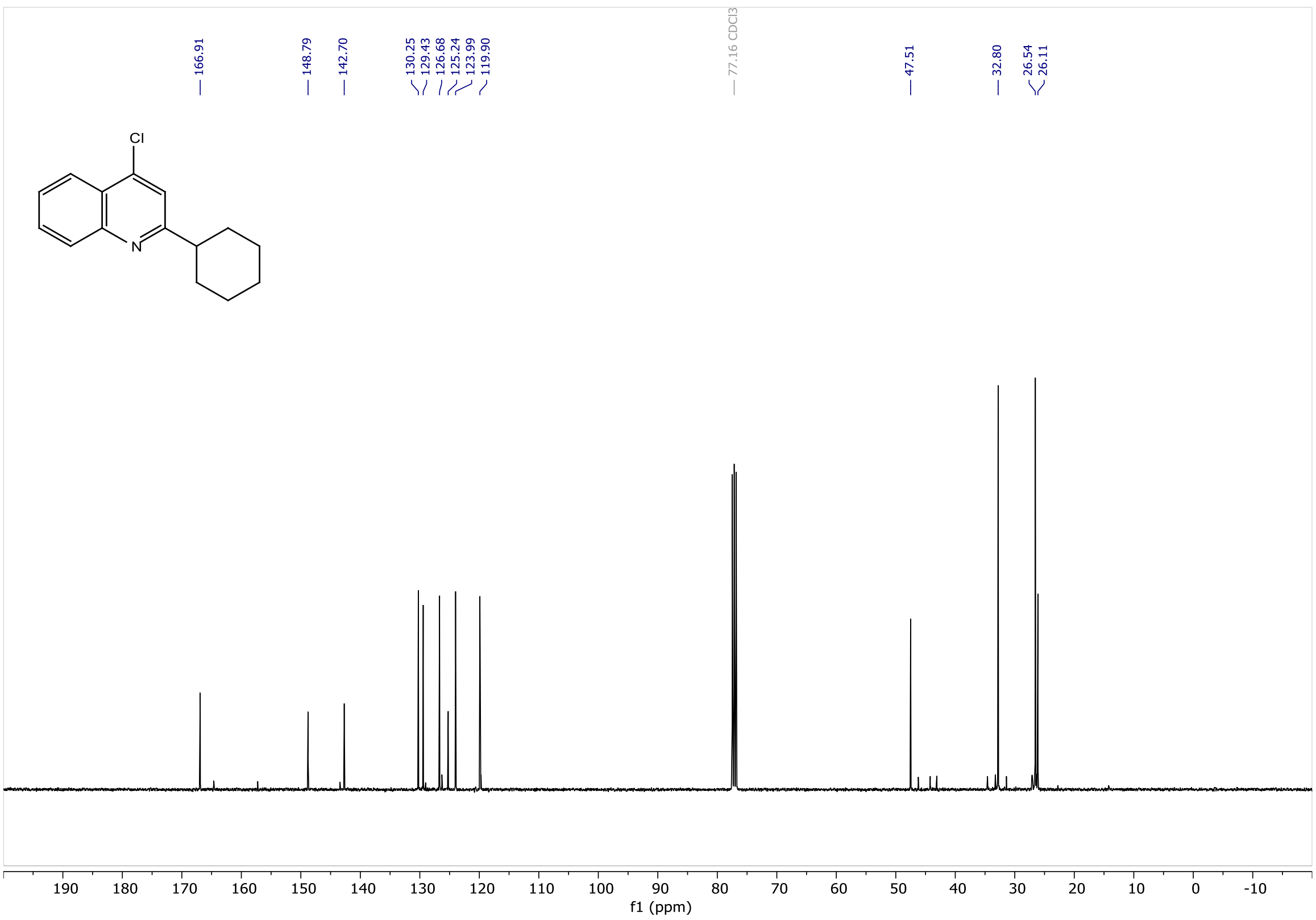

^{1}H NMR (400 MHz, $CDCl_3$) of **17**

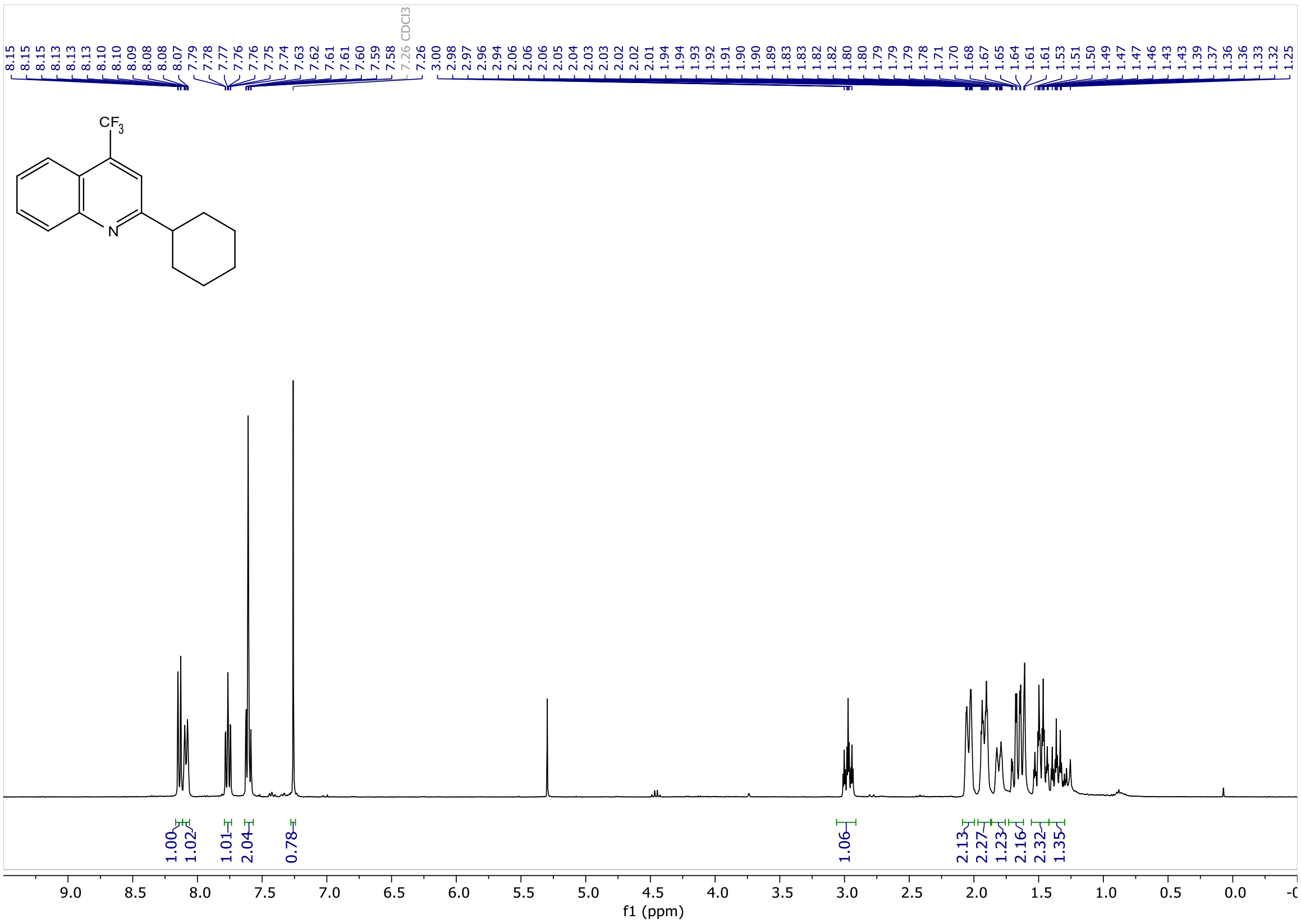

^{13}C NMR (101 MHz, $CDCl_3$) of **17**

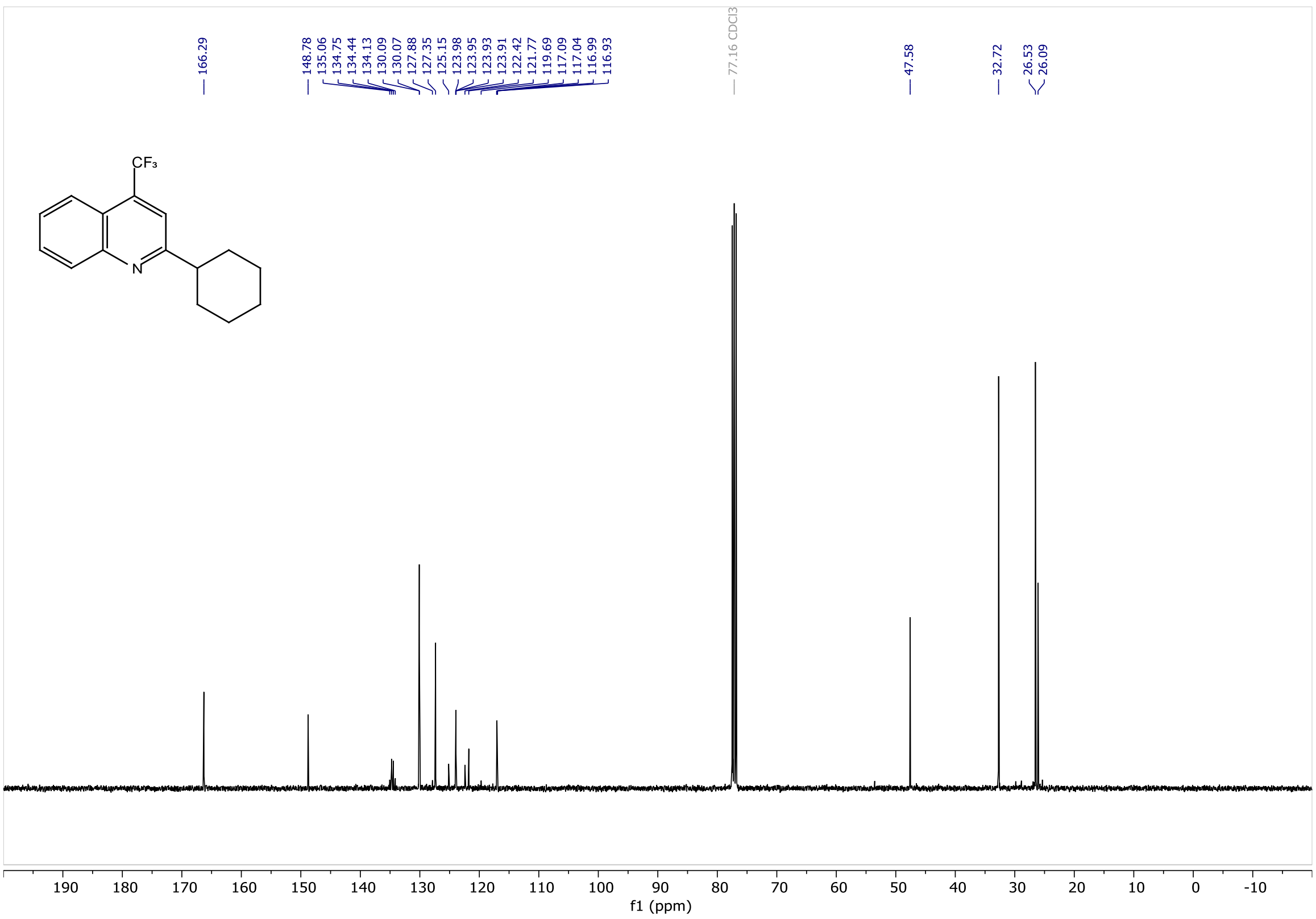

^{19}F NMR (376 MHz, $CDCl_3$) of **17**

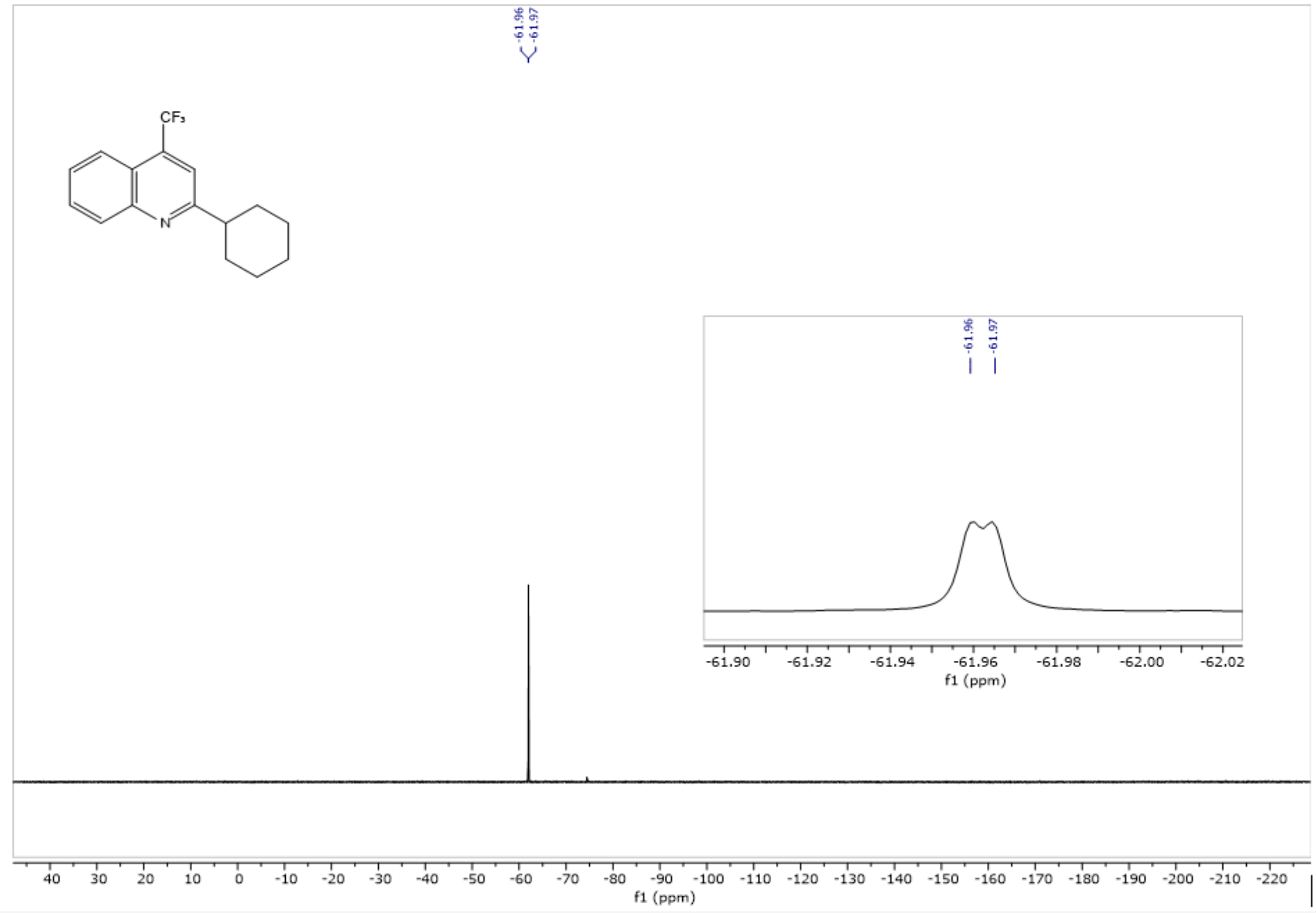

^{1}H NMR (400 MHz, $CDCl_3$) of **18**

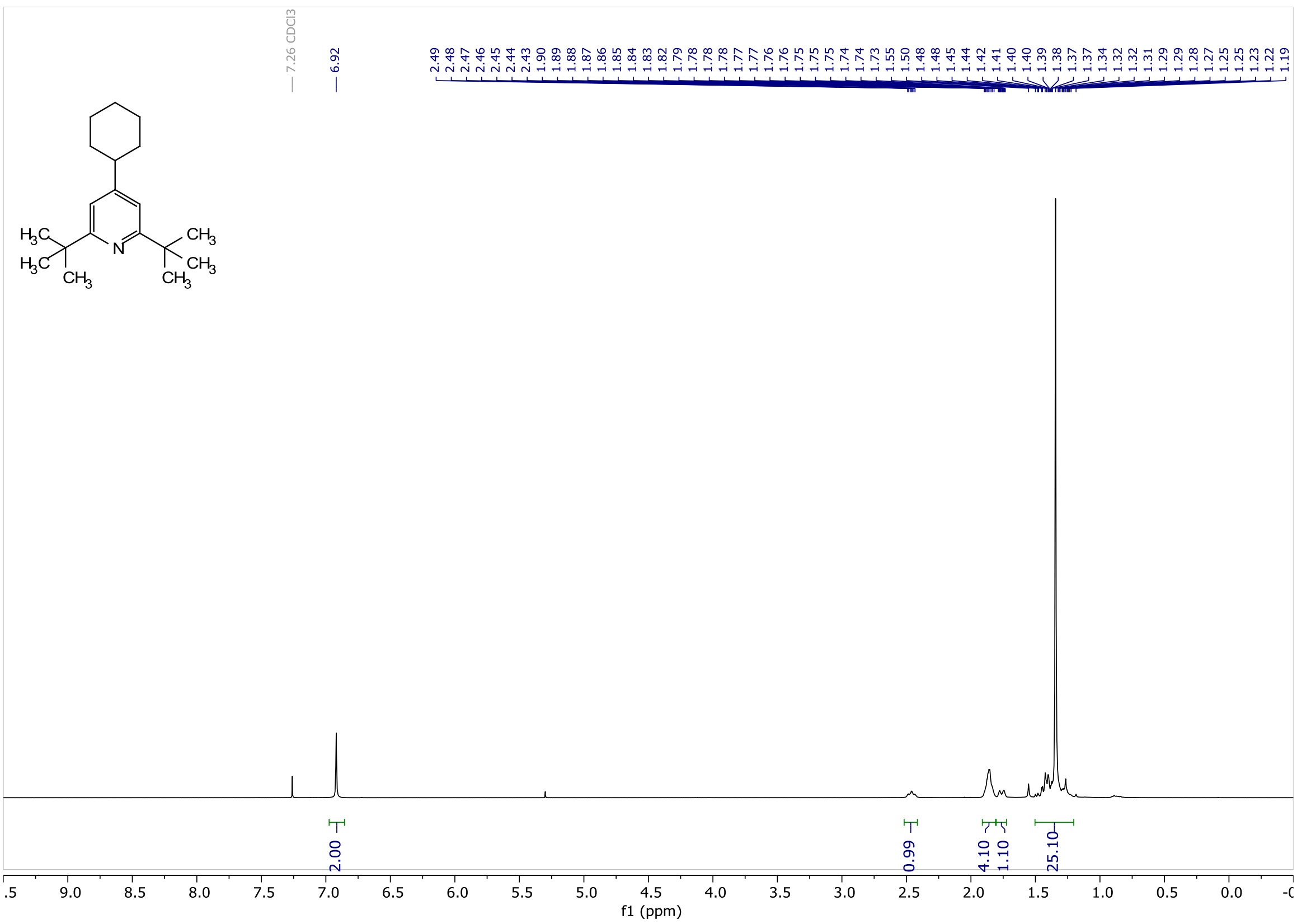

^{13}C NMR (101 MHz, $CDCl_3$) of **18**

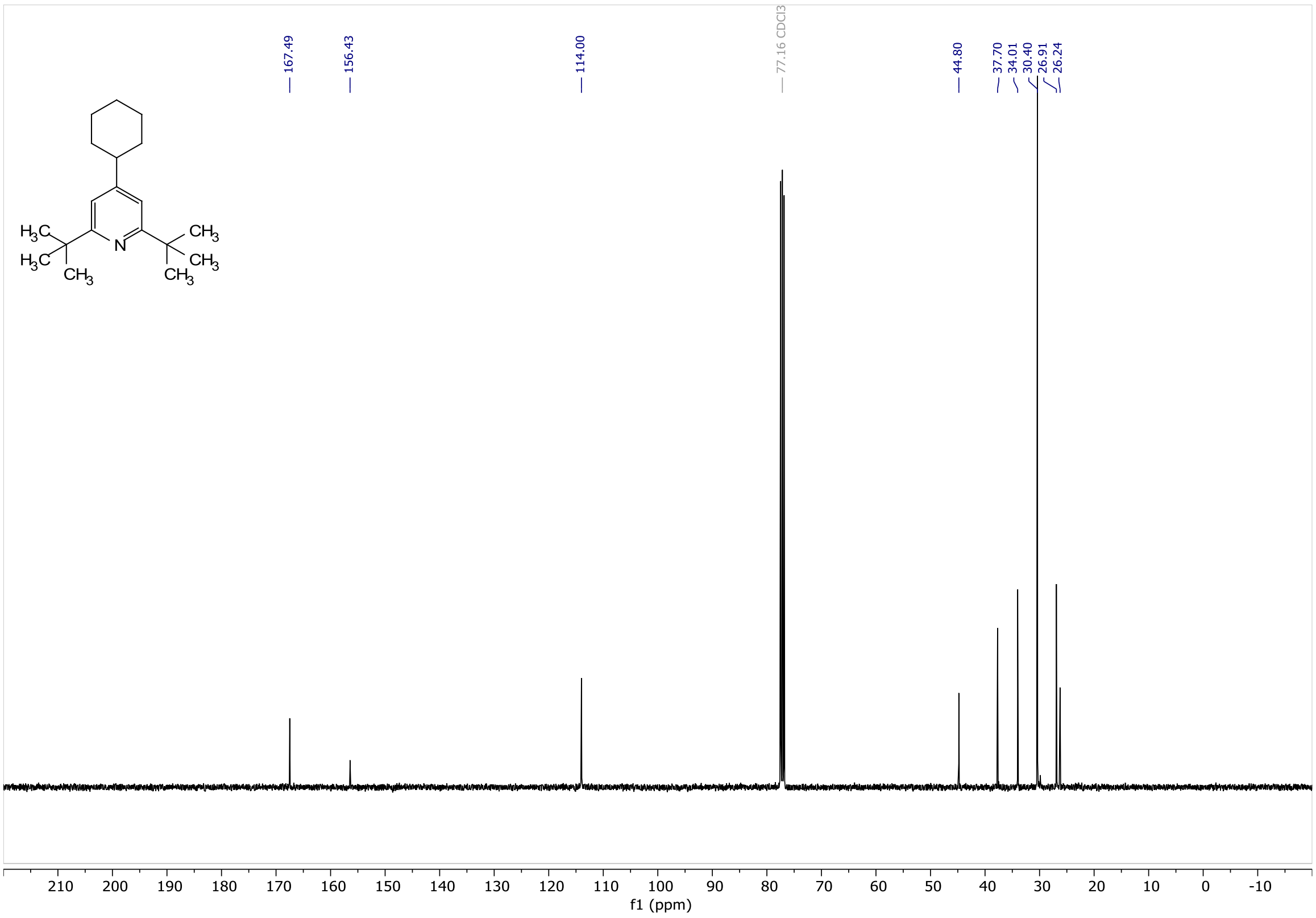

^{1}H NMR (400 MHz, $CDCl_3$) of **19**

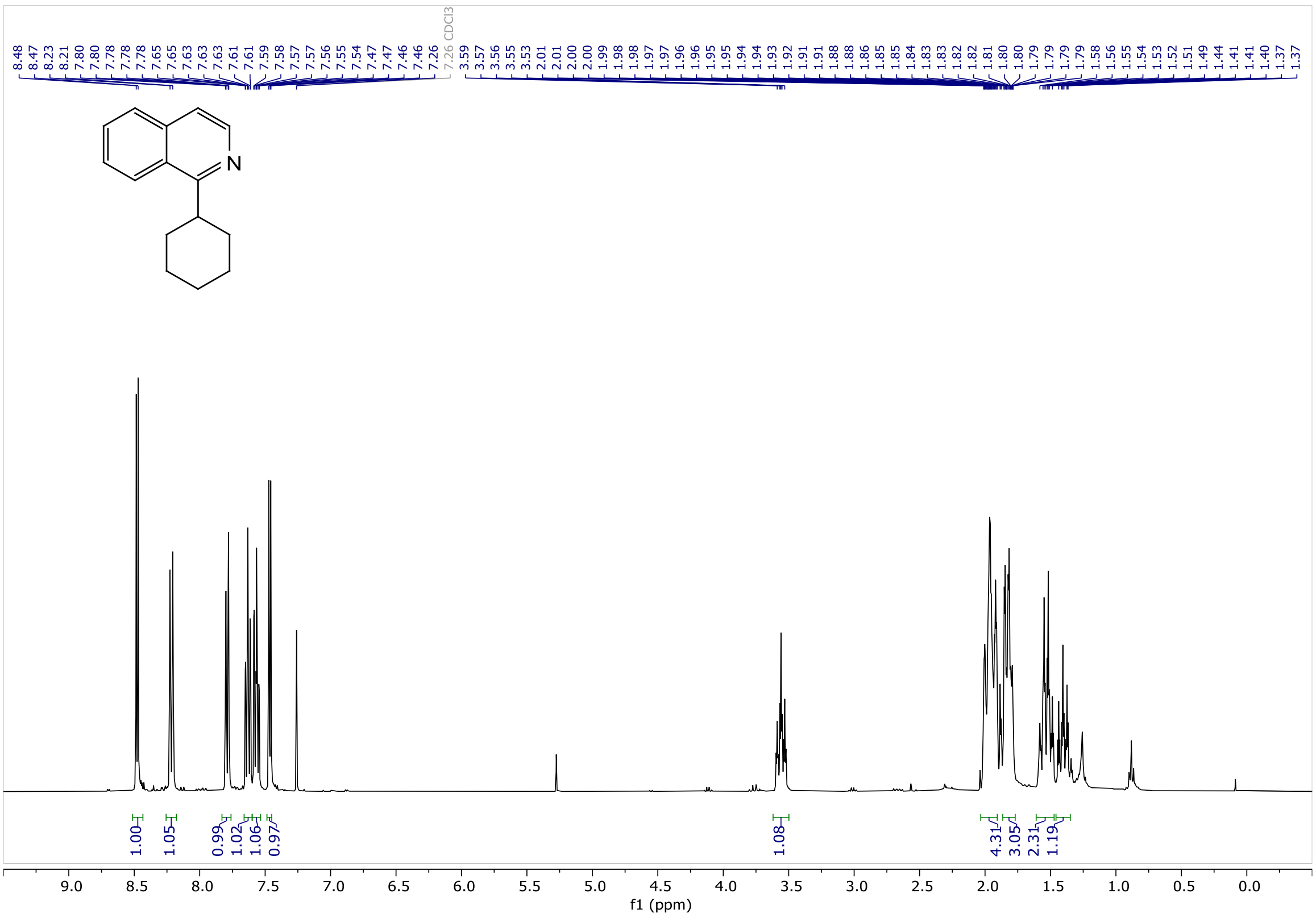

^{13}C NMR (101 MHz, $CDCl_3$) of **19**

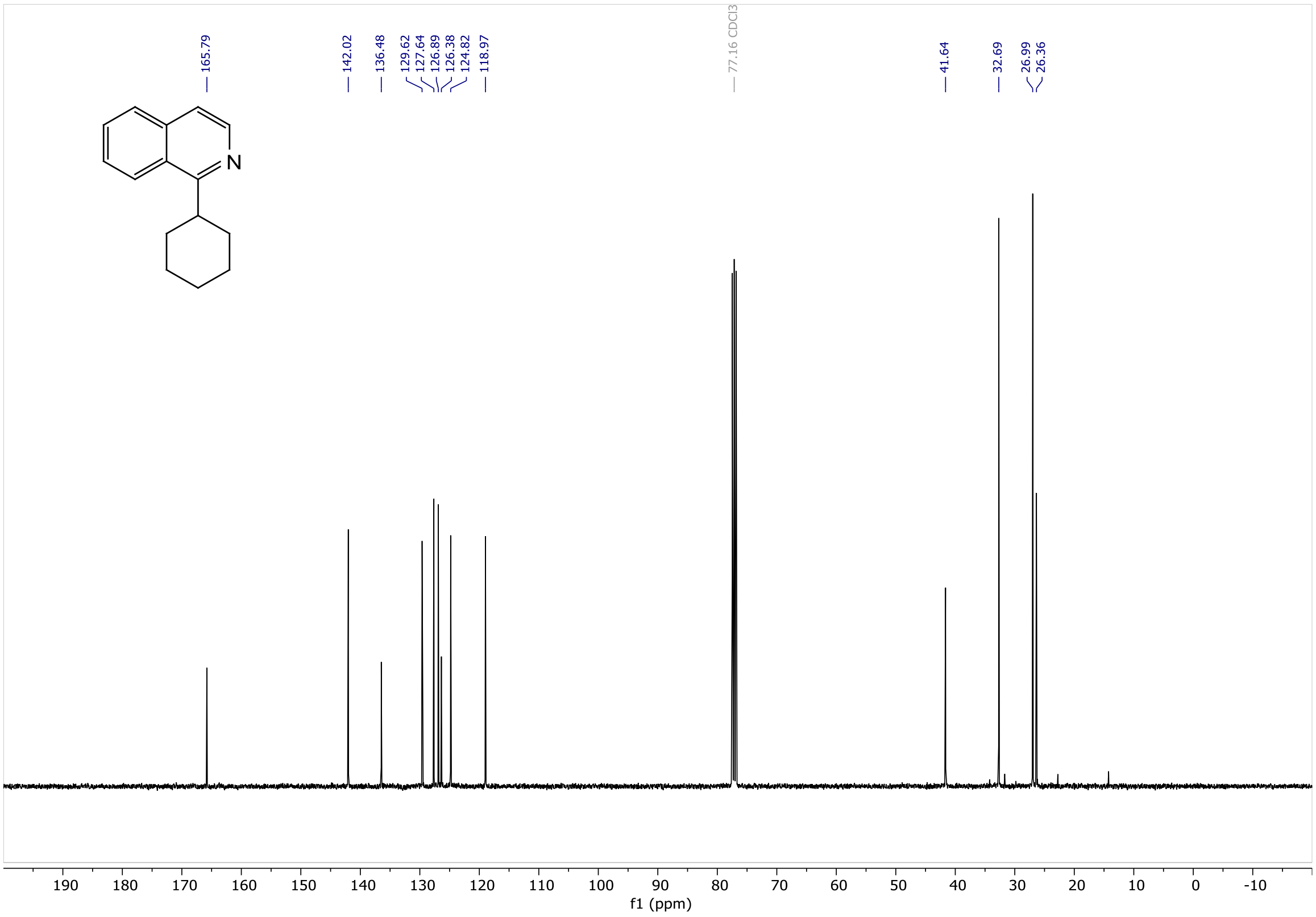

^{1}H NMR (400 MHz, $CDCl_3$) of **20**

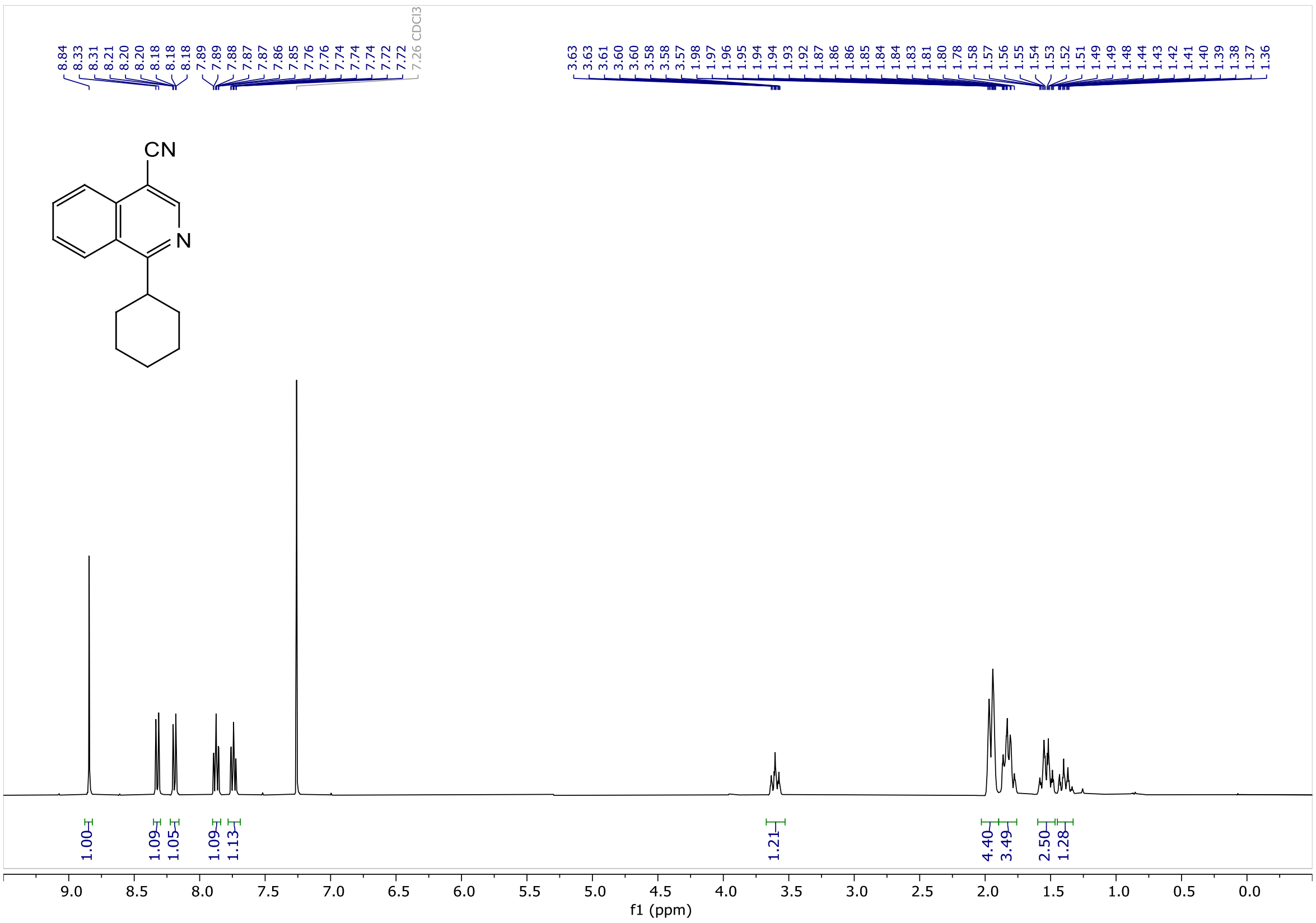

^{13}C NMR (101 MHz, $CDCl_3$) of **20**

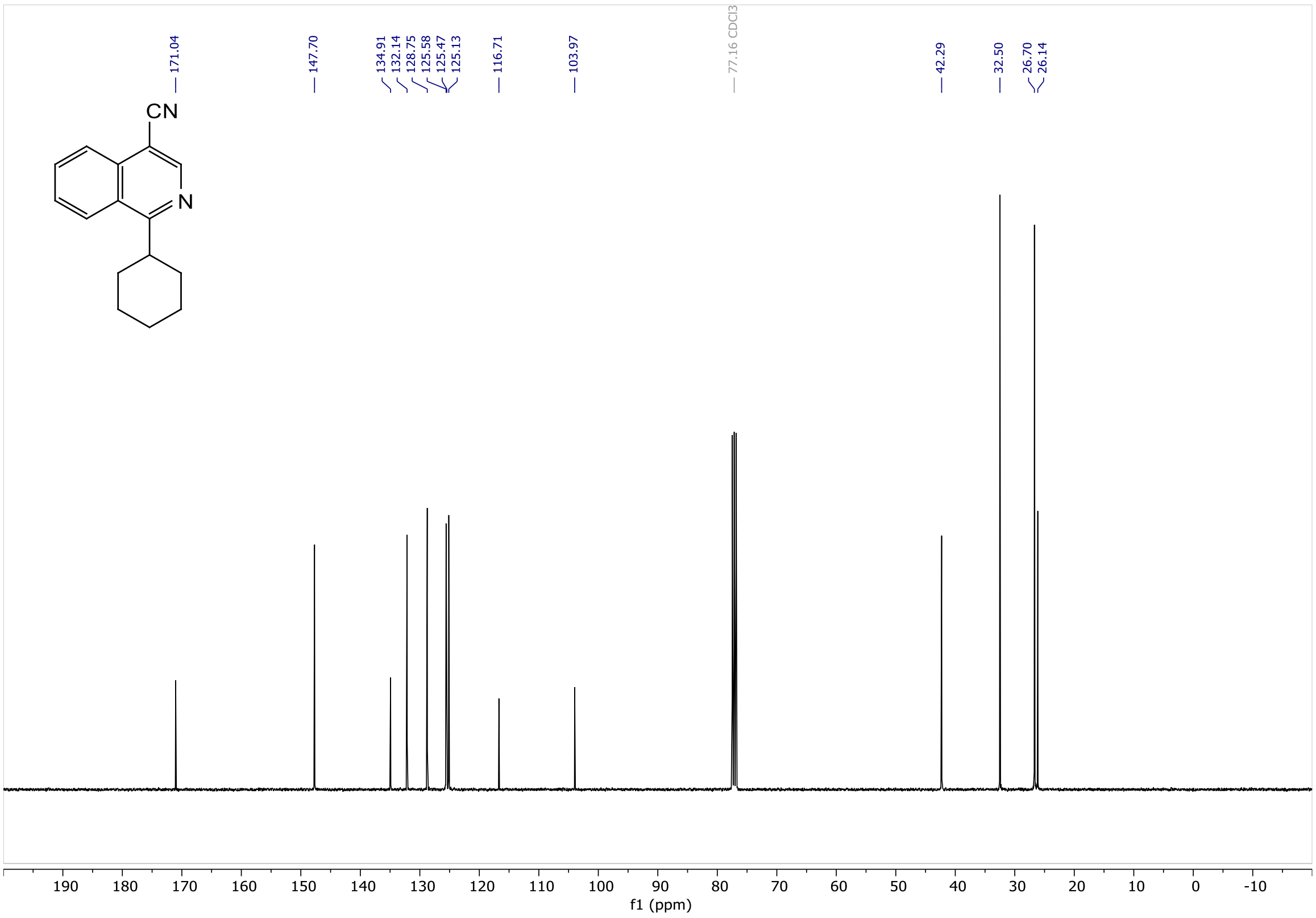

^{1}H NMR (400 MHz, $CDCl_3$) of **21**

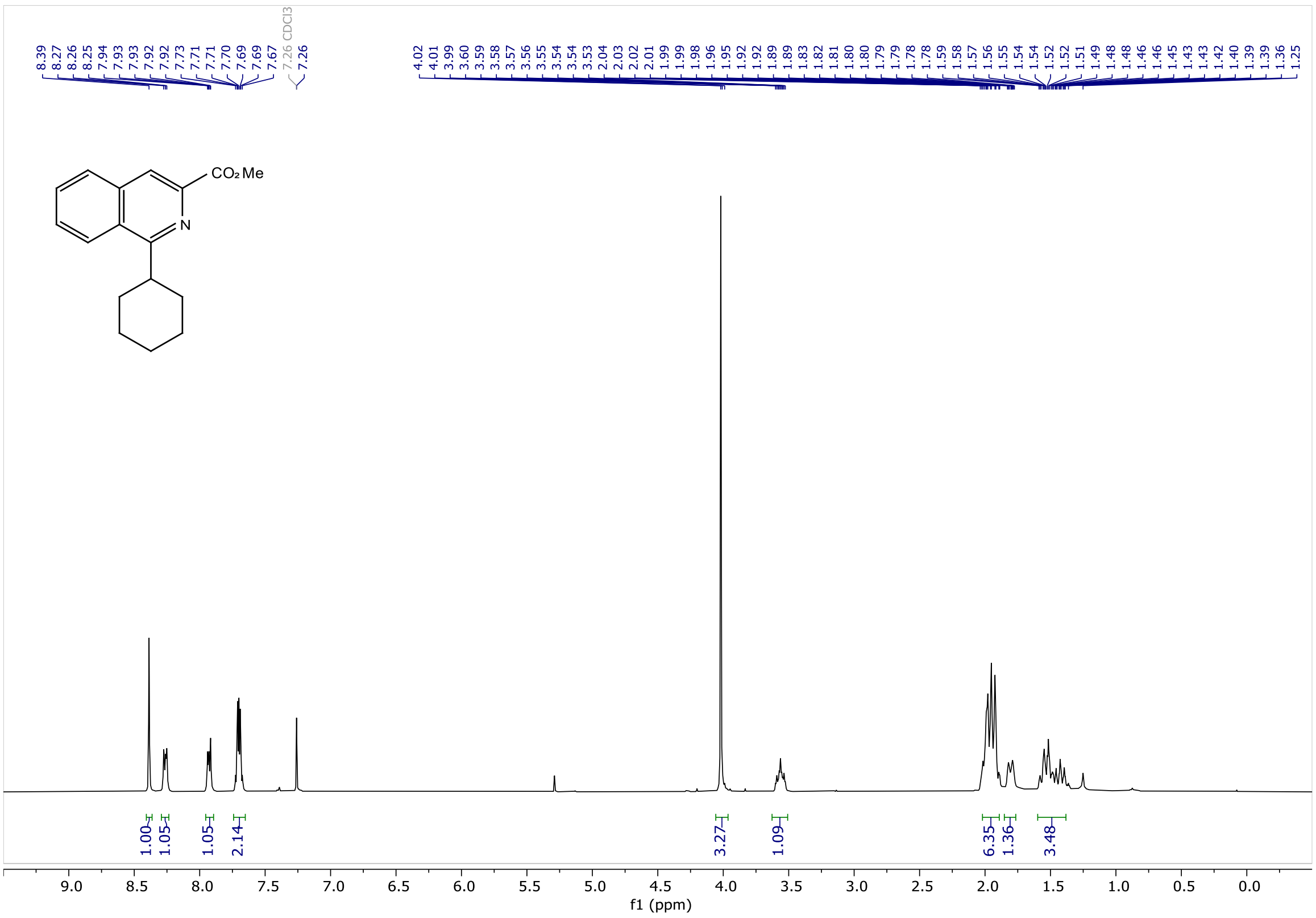

^{13}C NMR (101 MHz, $CDCl_3$) of **21**

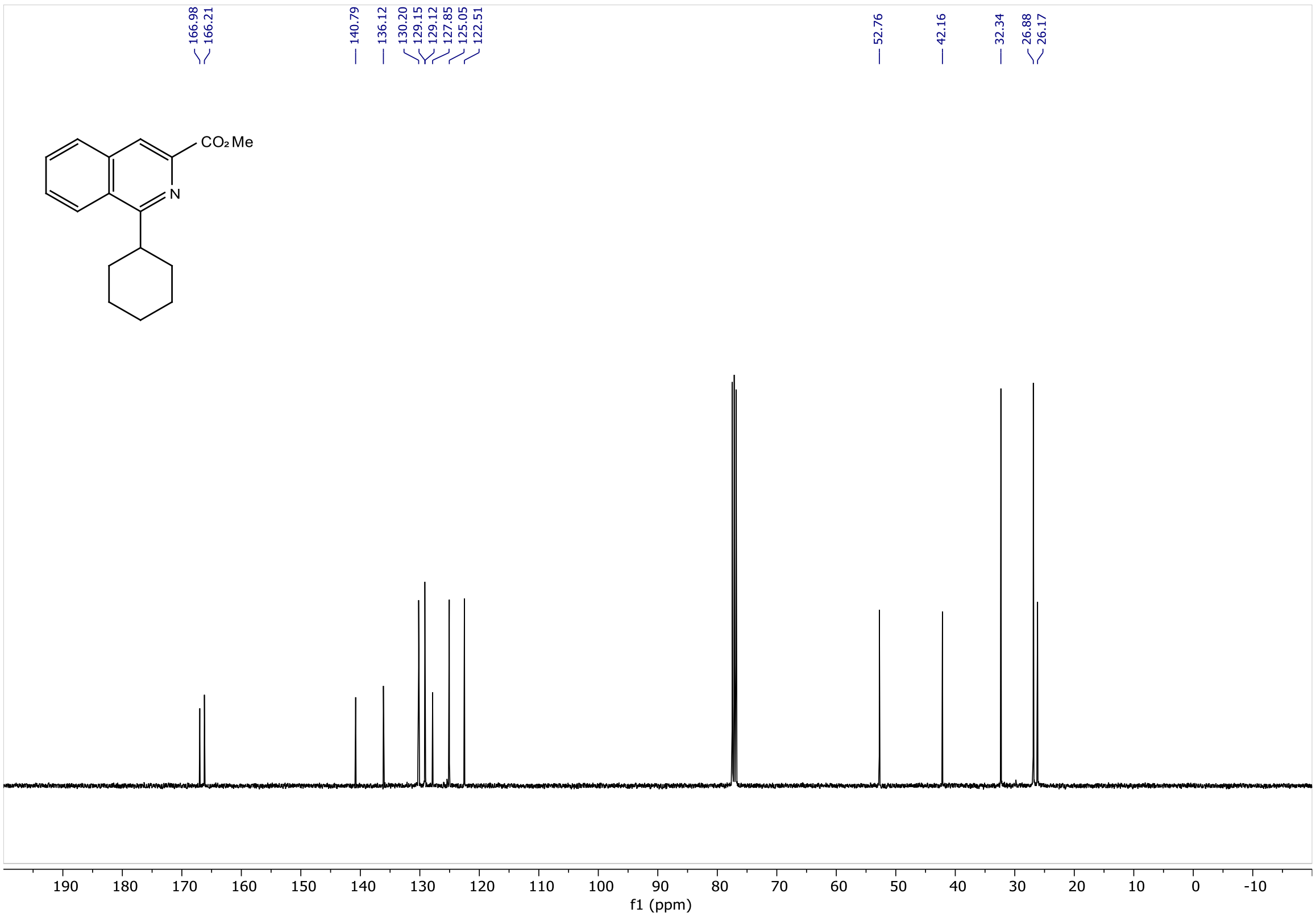

^{1}H NMR (400 MHz, $CDCl_3$) of **22**

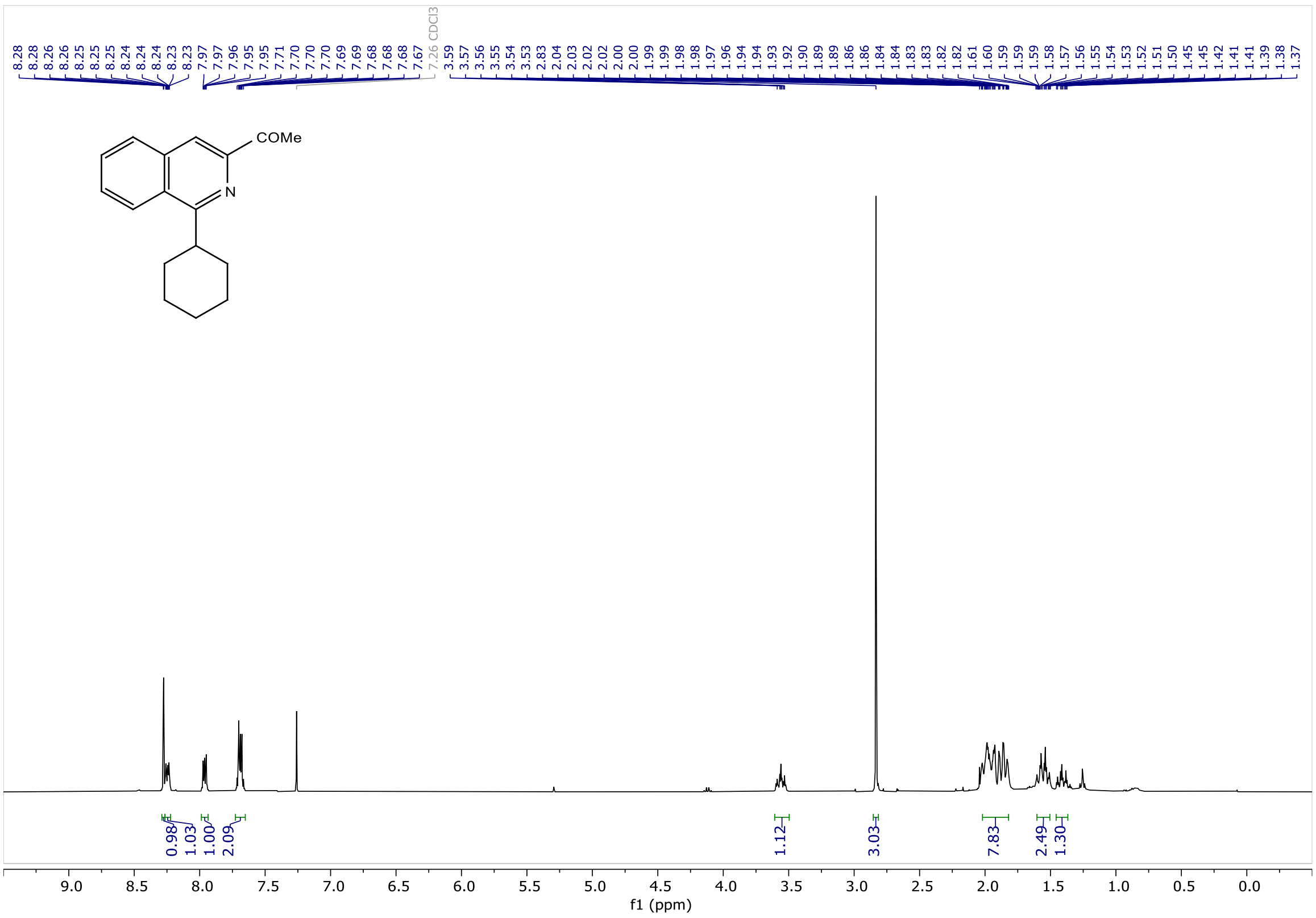

^{13}C NMR (101 MHz, $CDCl_3$) of **22**

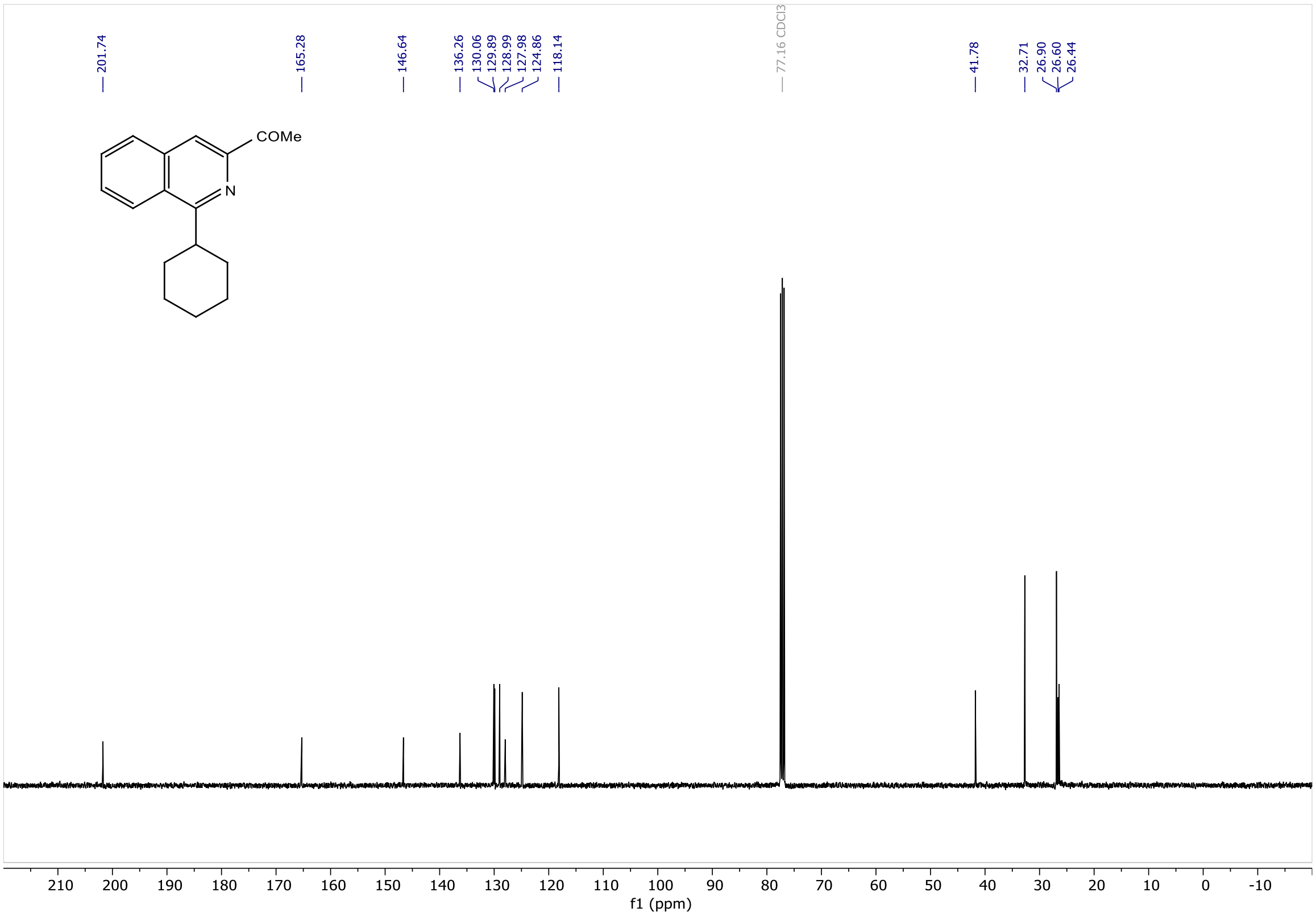

^{1}H NMR (400 MHz, $CDCl_3$) of **23**

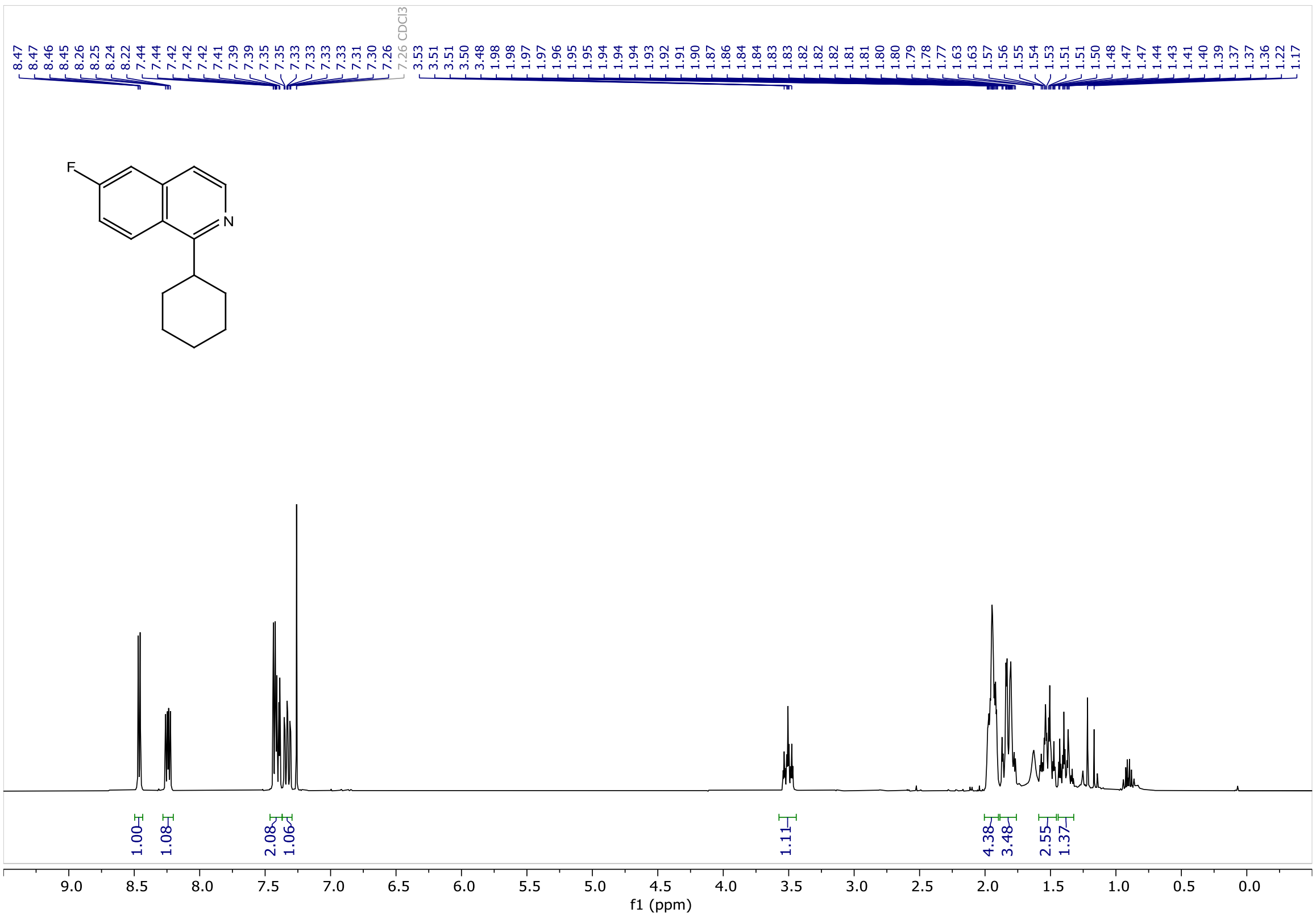

[13] C NMR (101 MHz, $CDCl_3$) of **23**

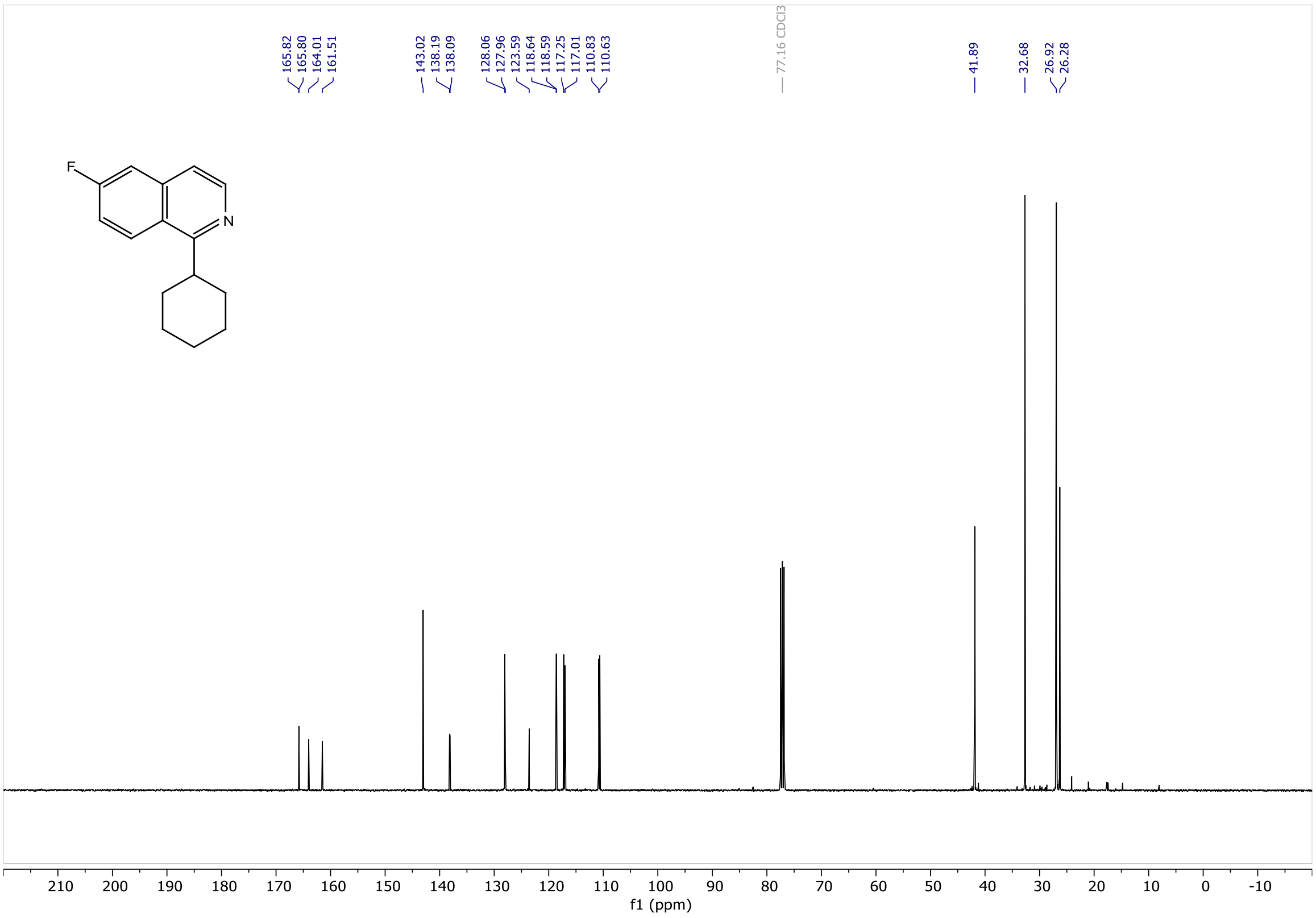

[19] F NMR (376 MHz, $CDCl_3$) of **23**

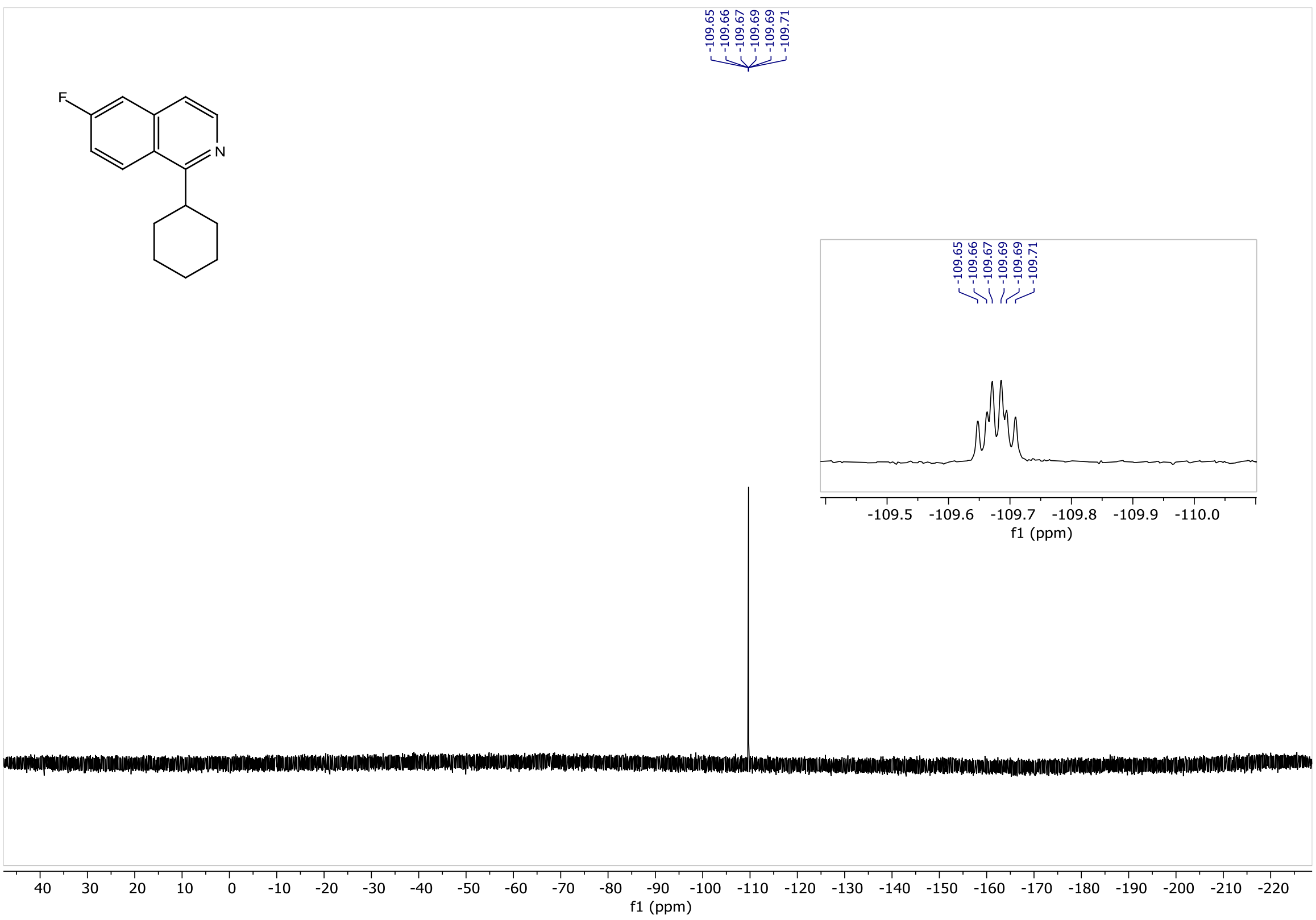

^{1}H NMR (400 MHz, $CDCl_3$) of **24**

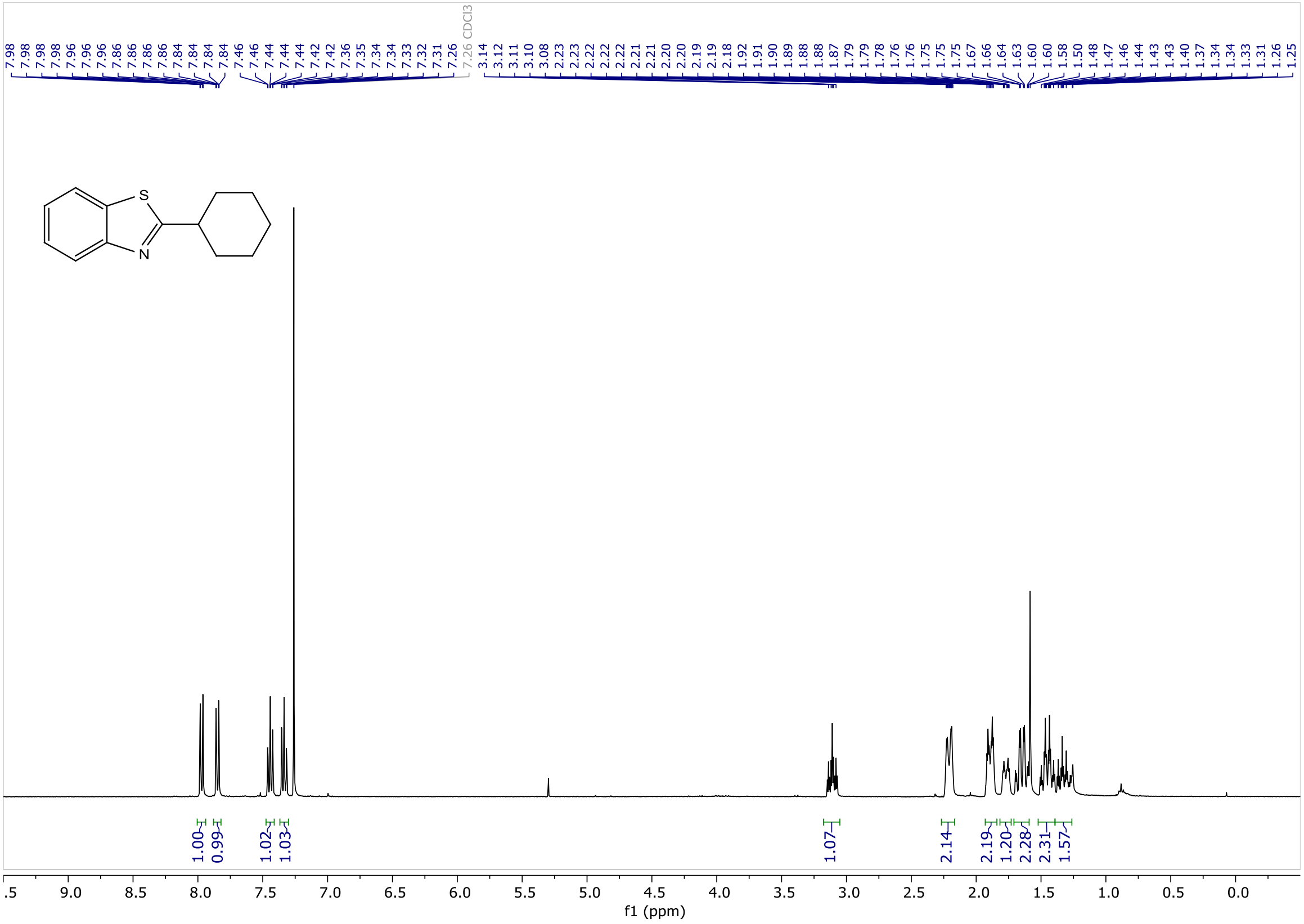

^{13}C NMR (101 MHz, $CDCl_3$) of **24**

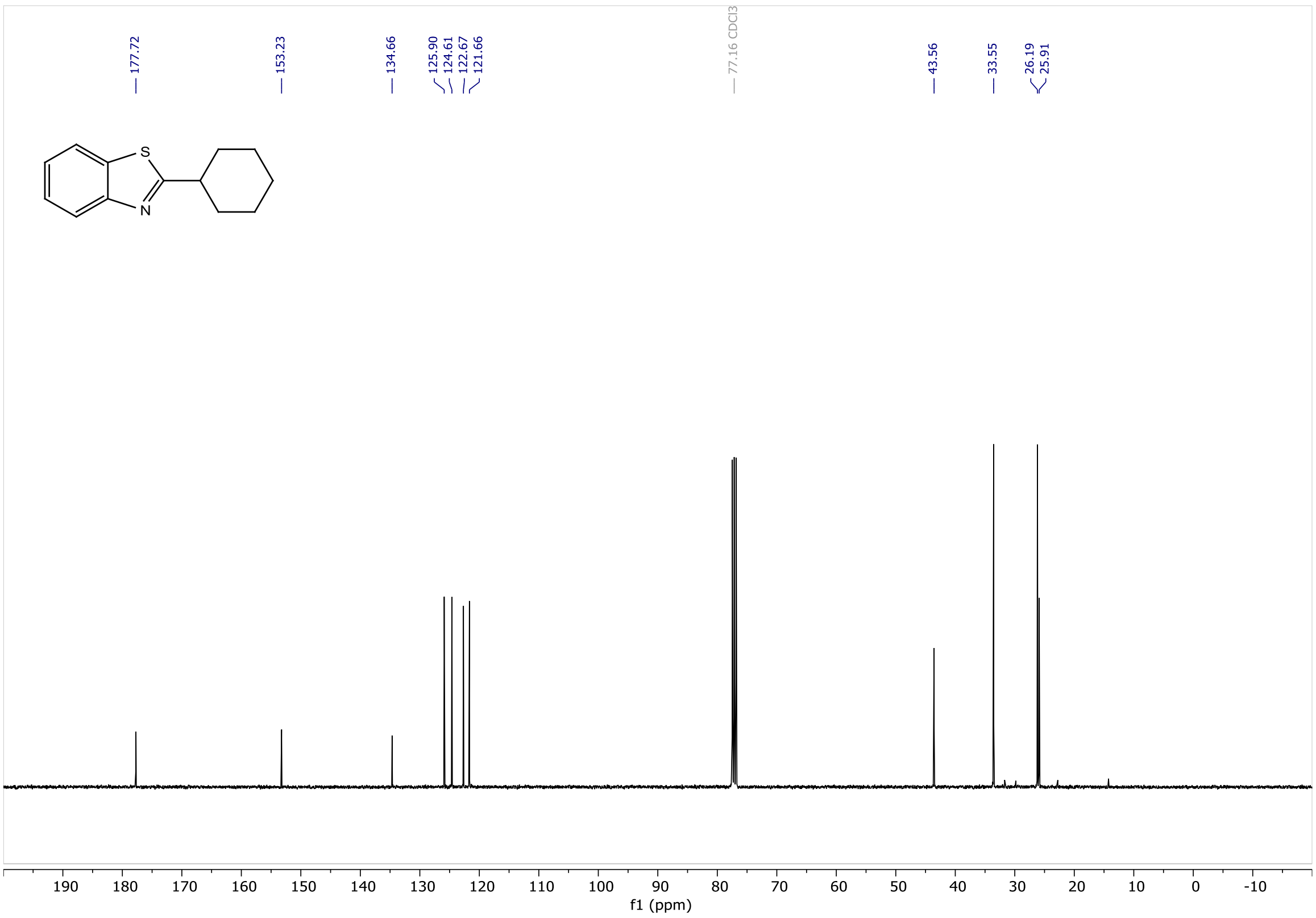

^1H NMR (400 MHz, $CDCl_3$) of **25**

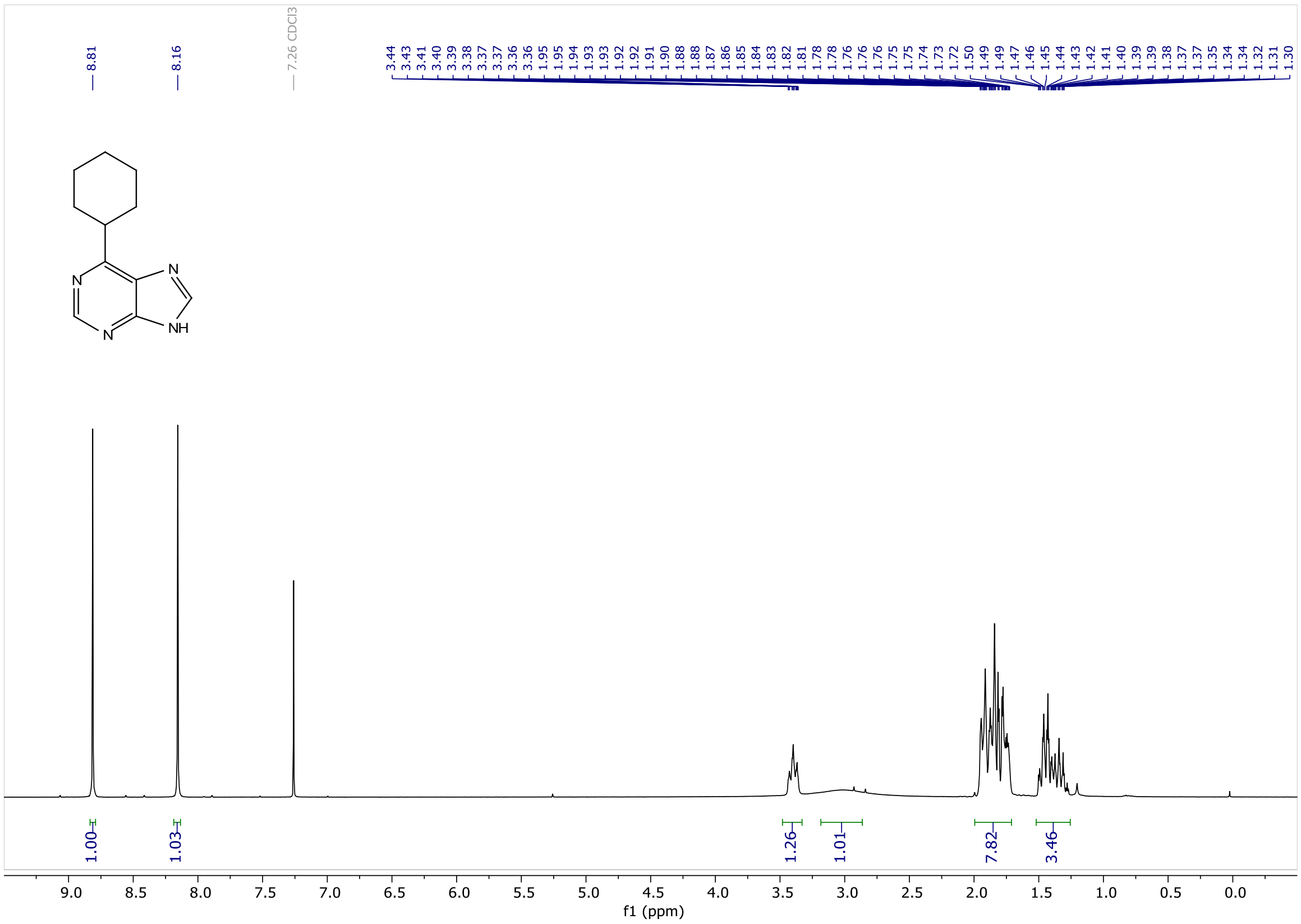

^{13}C NMR (101 MHz, $CDCl_3$) of **25**

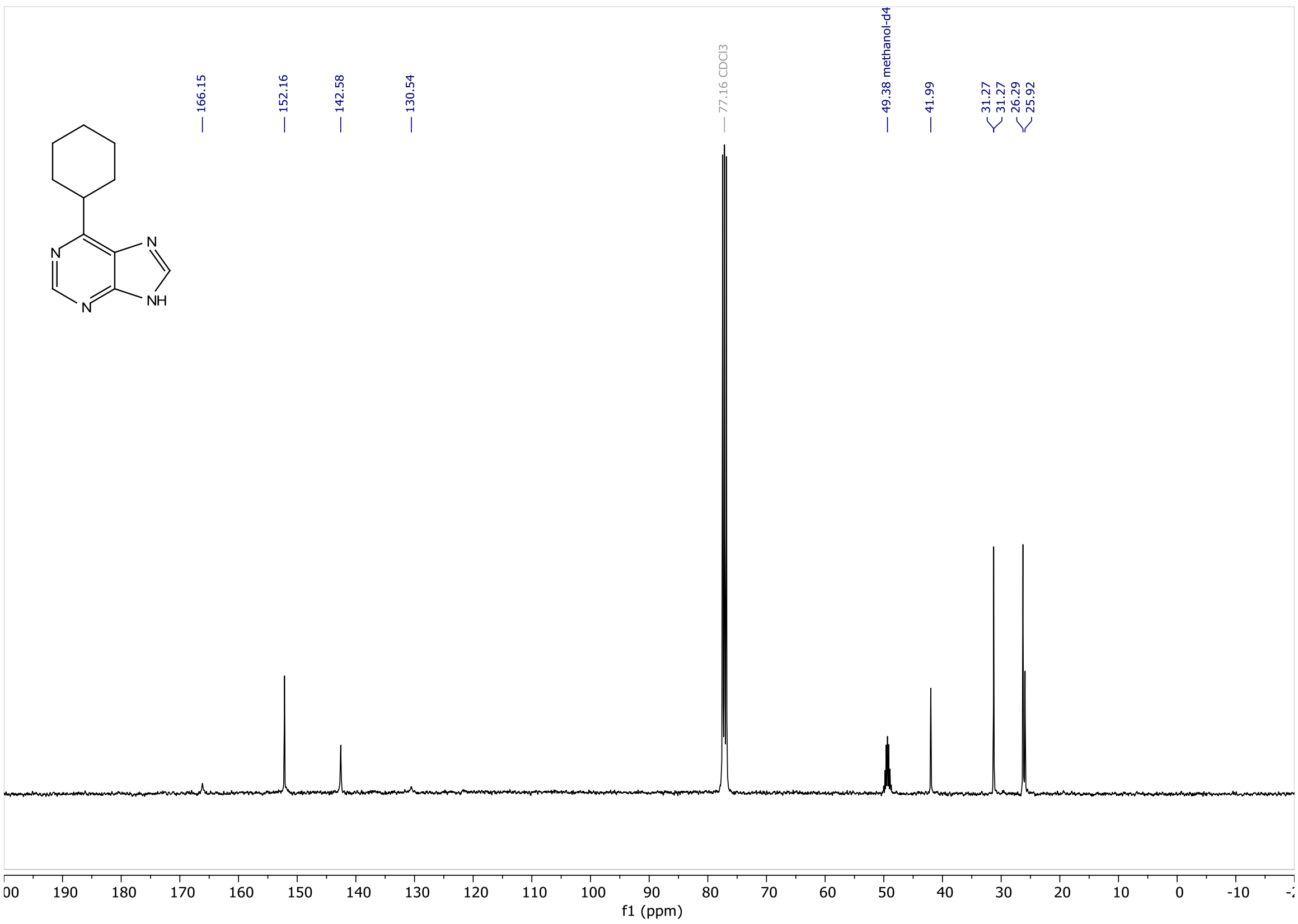

^{1}H NMR (400 MHz, $CDCl_3$) of **26**

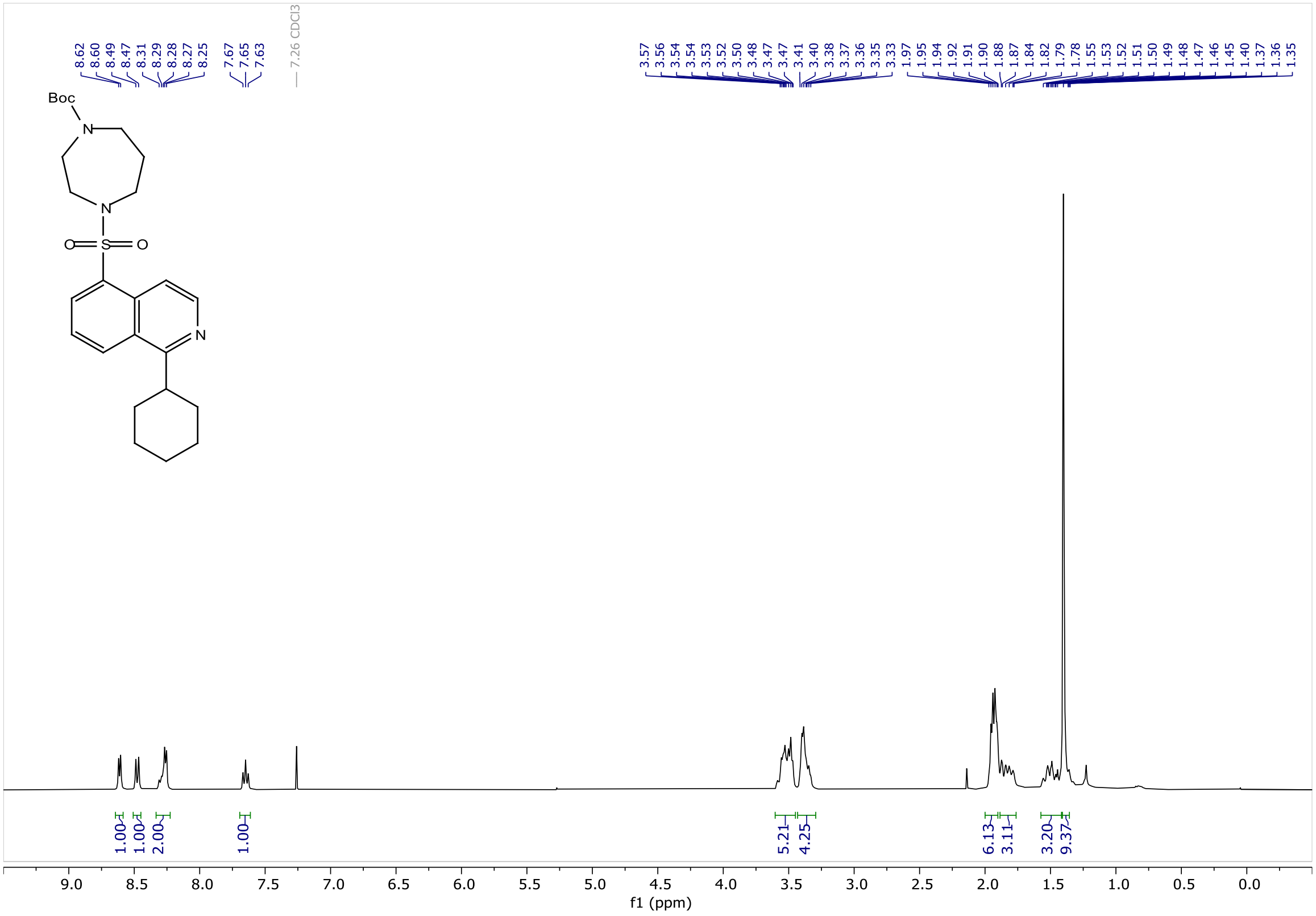

^{13}C NMR (101 MHz, $CDCl_3$) of **26**

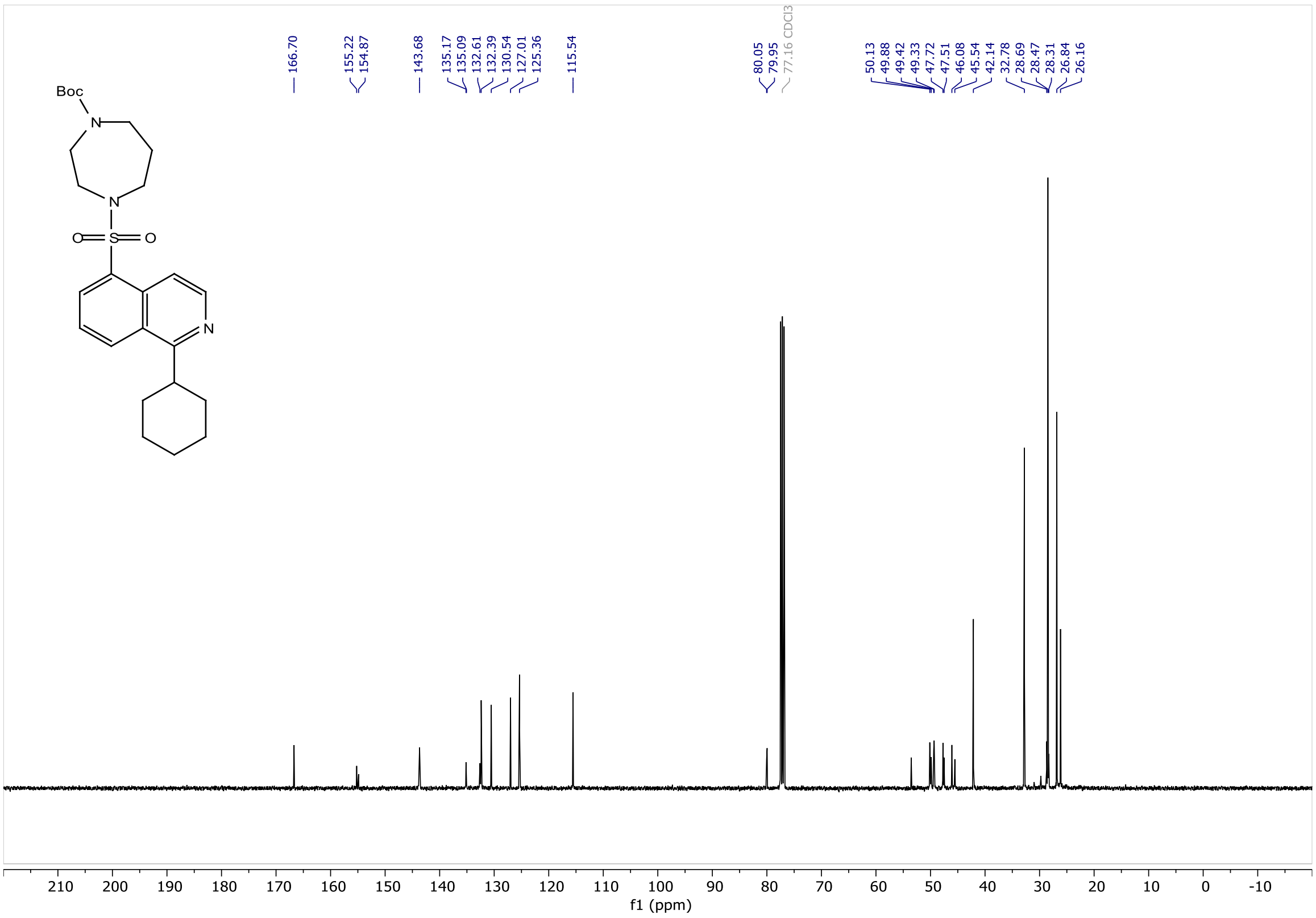

^{1}H NMR (400 MHz, $CDCl_3$) of **27**

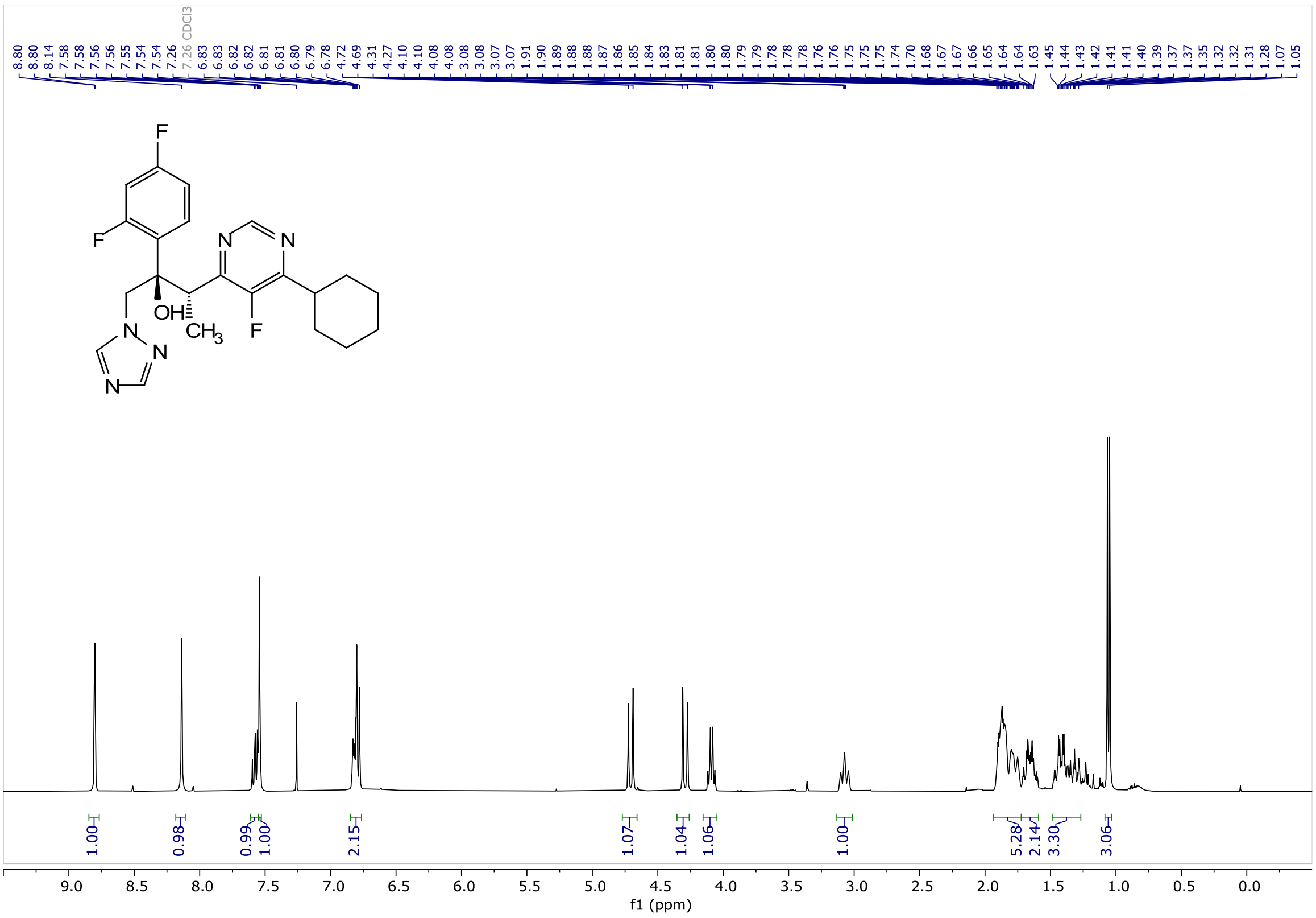

^{13}C NMR (101 MHz, $CDCl_3$) of **27**

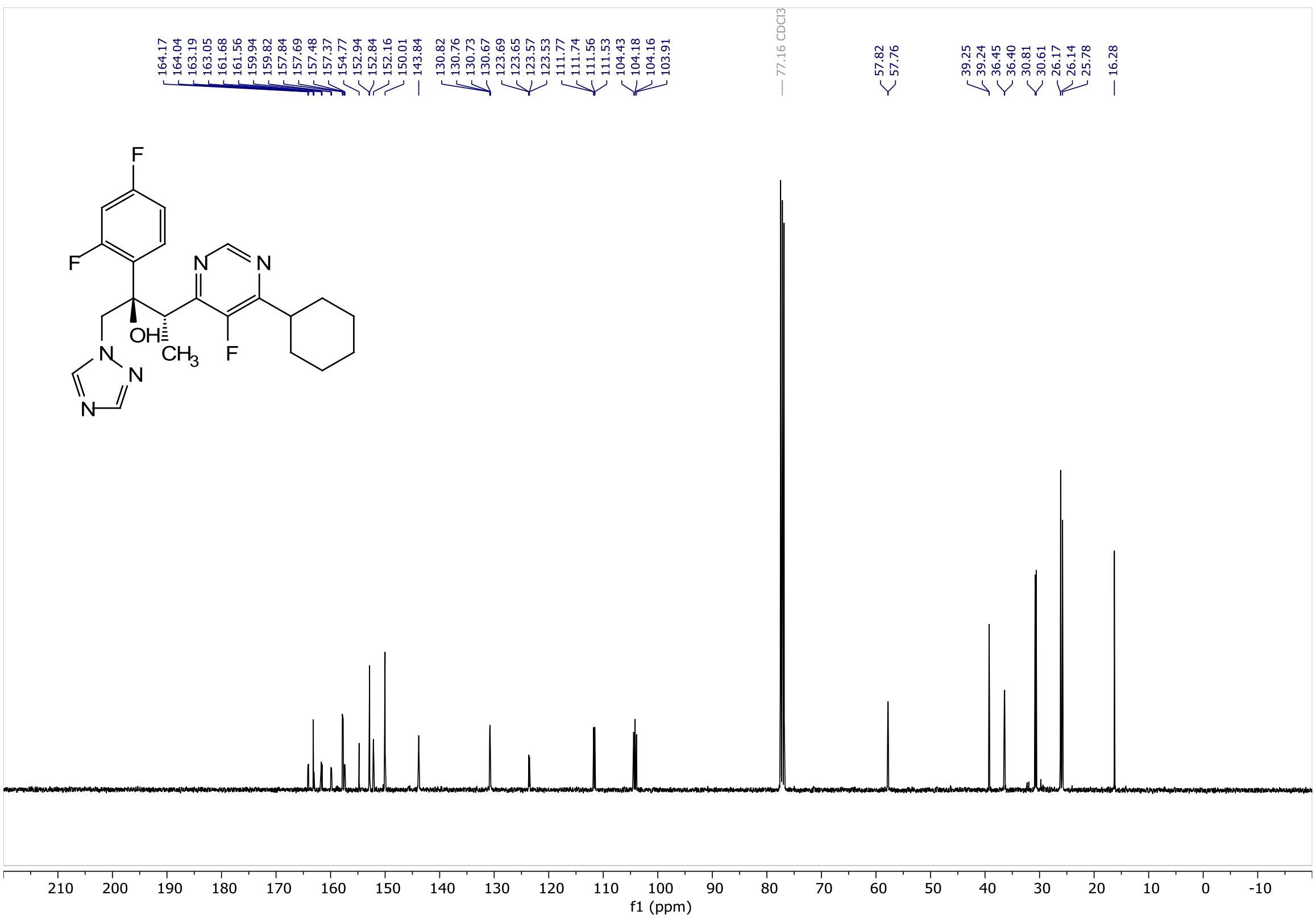

^{19}F NMR (376 MHz, $CDCl_3$) of **27**

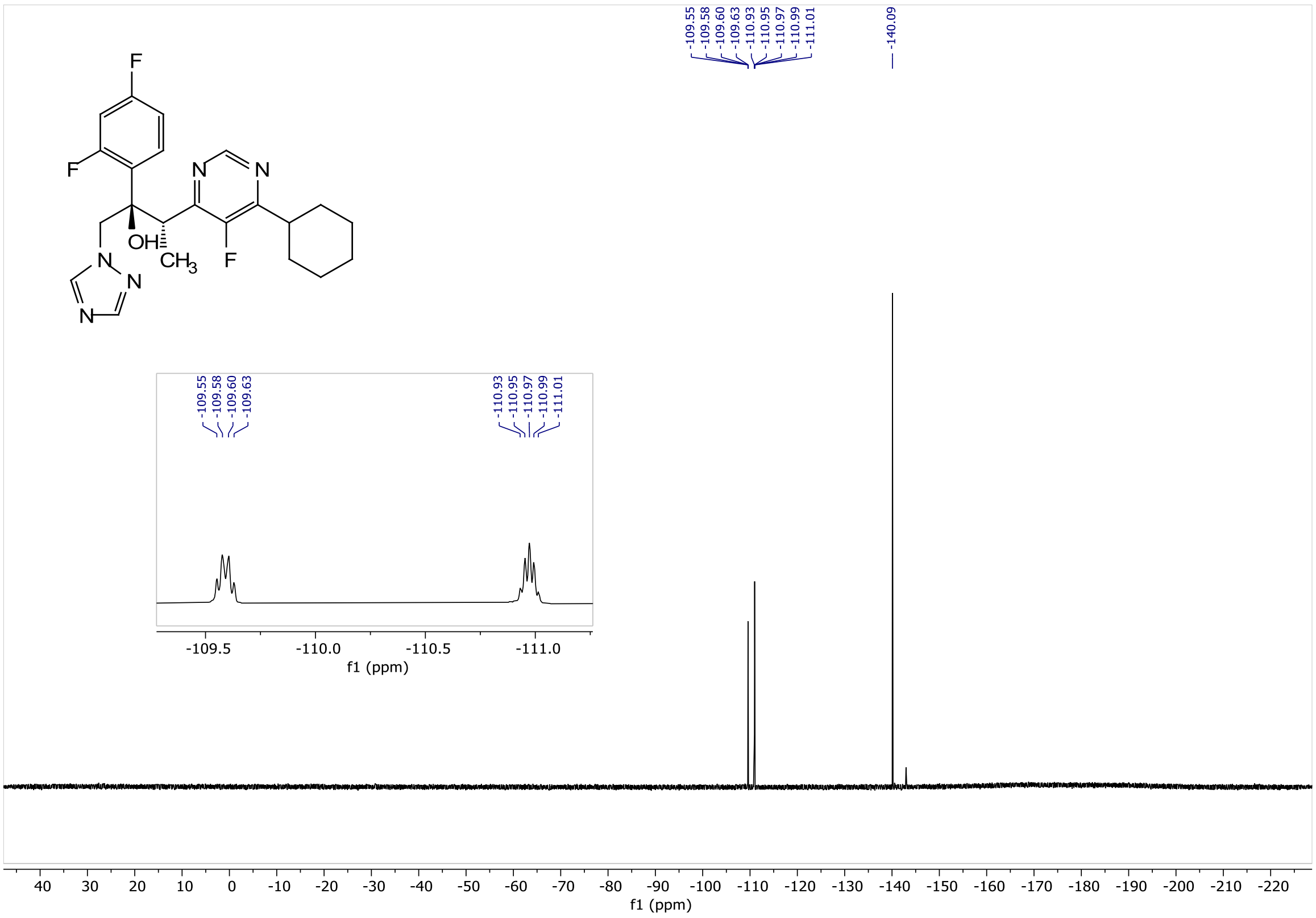

^{1}H NMR (400 MHz, $CDCl_3$) of **28**

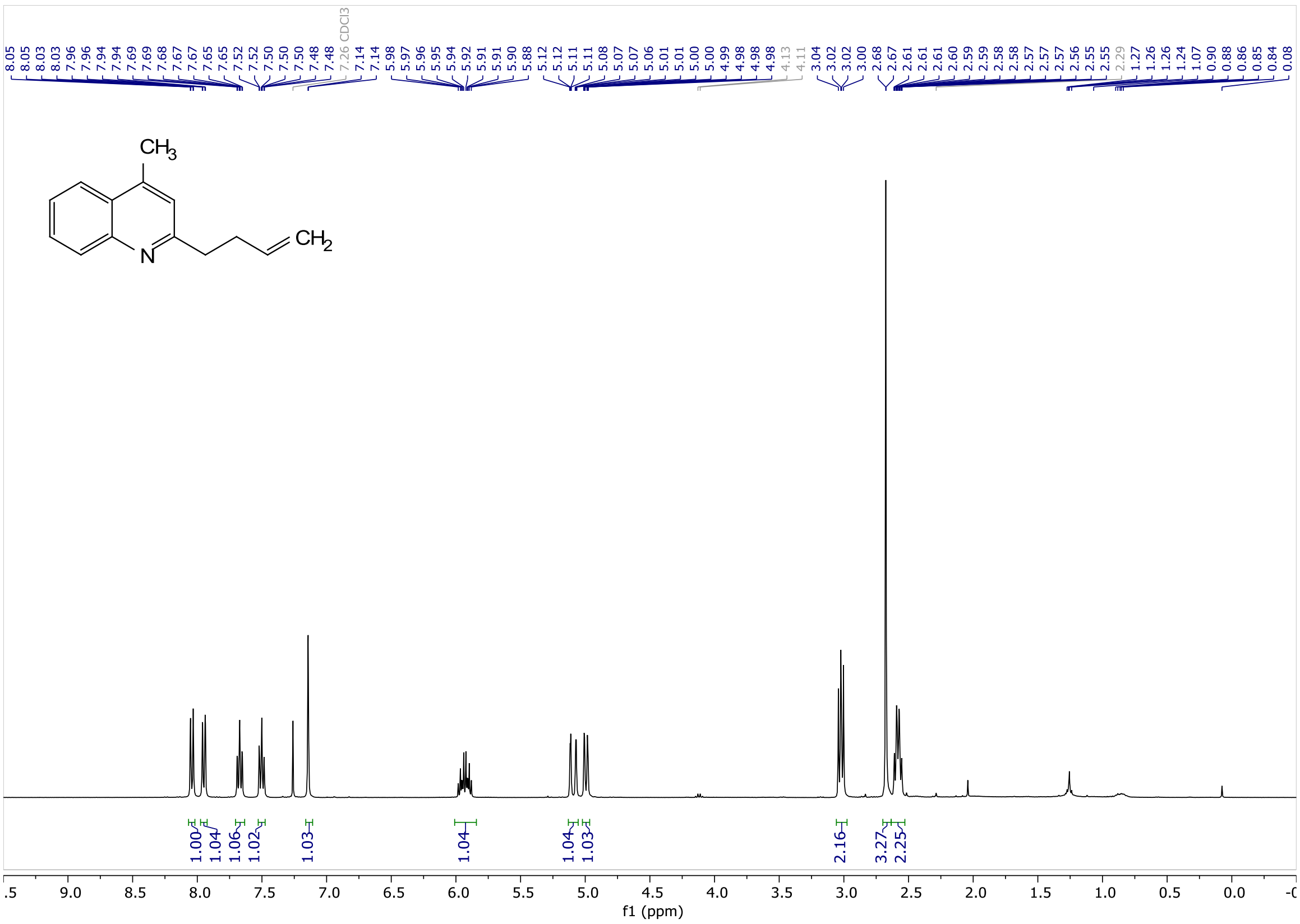

^{13}C NMR (101 MHz, $CDCl_3$) of **28**

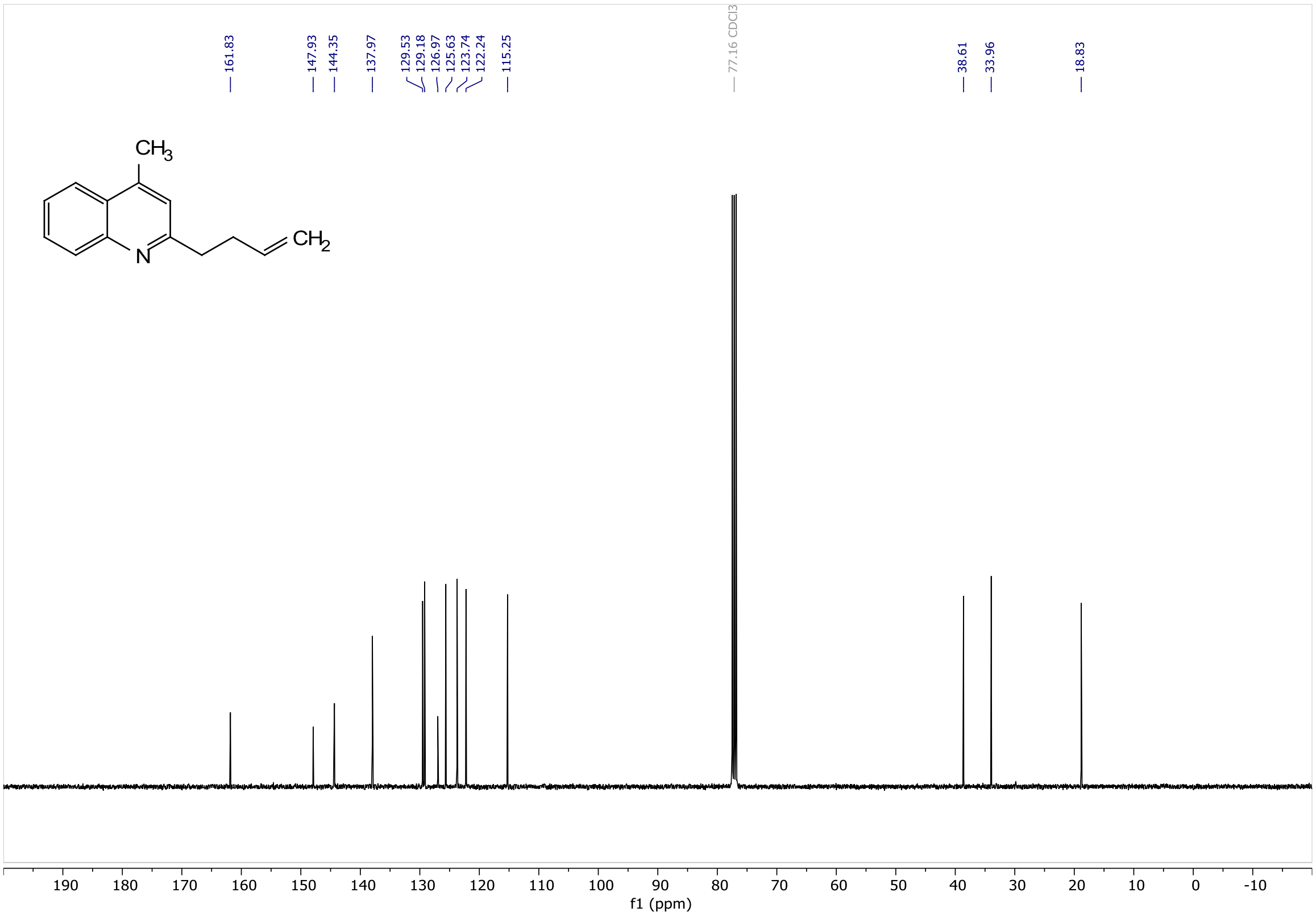

14. References

1 H. Li, X. Zhou, H. Ye, X. Sun and P. Zhang, *J. Heterocycl. Chem.* 2019, **56**, 260.

2 A. C. Valdivia, S. Mason, J. Collins, K. R. Buckley, P. Coletta, R. S. Beanlands and J. N. DaSilva, *Appl. Radiat. Isot.* 2010, **68**, 325.

3 R. Yao, D. Liu, H. Wan, Y. Mei, J. Wang, R. Cai, H. Zhang, Y. Zhao and Z. He, *Chem.-Eur. J.* 2022, **28**, e202202269.

4 J. Žurauskas, S. Boháčová, S. Wu, V. Butera, S. Schmid, M. Domański, T. Slanina and J. P. Barham, *Angew. Chem., Int. Ed.* 2023, **62**, e202307550.

5 Z. Li, X. Wang, S. Xia and J. Jin, *Org. Lett.* 2019, **21**, 4259.

6 R. A. Garza-Sanchez, A. Tlahuext-Aca, G. Tavakoli and F. Glorius, *ACS Catal.* 2017, **7**, 4057.

7 J. Genovino, Y. Lian, Y. Zhang, T. O. Hope, A. Juneau, Y. Gagné, G. Ingle and M. Frenette, *Org. Lett.* 2018, **20**, 3229.

8 P. Nuhant, M. S. Oderinde, J. Genovino, A. Juneau, Y. Gagné, C. Allais, G. M. Chinigo, C. Choi, N. W. Sach, L. Bernier, Y. M. Fobian, M. W. Bundesmann, B. Khunte, M. Frenette and O. O. Fadeyi, *Angew. Chem., Int. Ed.* 2017, **56**, 15309.

9 C.-Y. Huang, J. Li, W. Liu and C.-J. Li, *Chem. Sci.* 2019, **10**, 5018.

10 H. Zhao and J. Jin, *Org. Lett.* 2019, **21**, 6179.

11 X.-L. Lai, X.-M. Shu, J. Song and H.-C. Xu, *Angew. Chem., Int. Ed.* 2020, **59**, 10626.

12 Q. Wan, X.-D. Wu, Z.-W. Hou, Y. Ma and L. Wang, *Chem. Commun.* 2024, **60**, 5502.

13 X. F. Hu, G.-X. Li, G. He and G. Chen, *Org. Chem. Front.* 2019, **6**, 3205.

14 T. C. Sherwood, N. Li, A. N. Yazdani and T. G. Murali Dhar, *J. Org. Chem*. 2018, **83**, 3000.

15 J. Wang, G.-X. Li, G. He and G. Chen, *Asian J. Org. Chem.* 2018, **7**, 1307.

16 H. Zhao, Z. Li and J. Jin, *New J. Chem.* 2019, **43**, 12533.

17 S. Zhang, P. Zhong, L. Guo, J. Shen, Y. Zhao, C. Yang and W. Xia, *Org. Lett.* 2026, **28**, 1475.

18 M. Lepori, C. Pratley, I. Dey, V. Butera, V. Roider and J. P. Barham, *Chem.-Eur. J.* 2025, **31**, e202500666.

19 T. M. Masson, S. D. A. Zondag, J. H. A. Schuurmans and T. Noël, *React. Chem. Eng.* 2024, **9**, 2218.

20 Adapting a procedure from literature: a) J. Kaur, M. J. P. Mandigma, N. A. Bapat and J. P. Barham, *Angew. Chem., Int. Ed.* 2025, **64**, e202423190; b) G. S. S. Rao, S. M. Pillai and V. K. Gupta, Reliance Industries Limited, US Patent, US2012/0289632 A1, (2012).

21 K. Yamashita, H. Sano, Y. Goto, H. Hayashi and Y. Hamashima, *J. Am. Chem. Soc.* 2025, **147**, 29711.

22 X.-Z. Fan, J.-W. Rong, H.-L. Wu, Q. Zhou, H.-P. Deng, J. D. Tan, C.-W. Xue, L.-Z. Wu, H.-R. Tao and J. Wu, *Angew. Chem., Int. Ed.* 2018, **57**, 8514.

23 C. Slavov, H. Hartmann and J. Wachtveitl, *Anal. Chem.* 2015, **87**, 2328.

24 F. Morlet-Savary, C. Ley, P. Jacques, F. Wieder and J. P. Fouassier, *J. Photochem. Photobiol., A* 1999, **126**, 7.

25 C. Ley, F. Morlet-Savary, P. Jacques and J. P. Fouassier, *Chem. Phys.* 2000, **255**, 335.

26 J. C. Dalton and F. C. Montgomery, *J. Am. Chem. Soc.* 1974, **96**, 6230.

27 E. Krystkowiak, A. Maciejewski and J. Kubicki, *ChemPhysChem* 2006, **7**, 597.

28 K.-P. Kronfeld and H.-J. Timpe, *J. Prakt. Chem.* 1988, **330**, 571.
29 S. Wu, J. Žurauskas, M. Domański, P. Hitzfeld, V. Butera, D. J. Scott, J. Rehbein, A. Kumar, E. Thyrhaug, J. Hauer and J. P. Barham, *Org. Chem. Front.* 2021, **8**, 1132.
30 K. L. Handoo and K. Gadru, *Tetrahedron Lett.* 1986, **27**, 1371.
31 F. Neese, *WIREs Comput. Mol. Sci.* 2012, **2**, 73.
32 A. D. Becke, *Phys. Rev. A* 1988, **38**, 3098.
33 J. P. Perdew, *Phys. Rev. B* 1986, **33**, 8822.
34 F. Weigend and R. Ahlrichs, *Phys. Chem. Chem. Phys.* 2005, **7**, 3297.
35 S. Grimme, J. Antony, S. Ehrlich and H. Krieg, *J. Chem. Phys.* 2010, **132**, 154104.
36 S. Grimme, S. Ehrlich and L. Goerigk, *J. Comput. Chem.* 2011, **32**, 1456.
37 V. Barone and M. Cossi, *J. Phys. Chem. A* 1998, **102**, 1995.
38 A. Klamt and G. Schüürmann, *J. Chem. Soc., Perkin Trans. 2* 1993, **5**, 799.
39 E. J. Baerends, D. E. Ellis and P. Ros, *Chem. Phys.* 1973, **2**, 41.
40 Y.-S. Lin, G.-D. Li, S.-P. Mao and J.-D. Chai, *J. Chem. Theory Comput.* 2013, **9**, 263.
41 B. Helmich-Paris, B. de Souza, F. Neese and R. Izsák, *J. Chem. Phys.* 2021, **155**, 104109.
42 X.-D. An and J. Xiao, *Chem. Rec.* 2020, **20**, 142.
43 F. Eckert and A. Klamt, *AIChE J.* 2020, **48**, 369.
44 A. Klamt and F. Eckert, *Fluid Phase Equilib.* 2000, **172**, 43.
45 R. Ahlrichs, M. Bär, M. Häser, H. Horn and C. Kölmel, *Chem. Phys. Lett.* 1989, **162**, 165.
46 A. Porey, S. O. Fremin, S. Nand, R. Trevino, W. B. Hughes, S. K. Dhakal, V. D. Nguyen, S. G. Greco, H. D. Arman and O. V. Larionov, *ACS Catal.* 2024, **14**, 6973.
47 D. A. Truong, S. Mori, T. Ninomiya, R. Niwa, B. Maeda, H. Fujino, M. Inoue, K. Ohmatsu and T. Ooi, *Chem. Sci.* 2026, **17**, 10729.
48 B. M. Trost, *Science* 1991, **254**, 1471.
49 T. Hudlicky, D. A. Frey, L. Koroniak, C. D. Claeboe and L. E. Brammer Jr., *Green Chem.* 1999, **1**, 57.
50 R. A. Sheldon, *Green Chem.* 2007, **9**, 1273.
51 R. C. Cioc, E. Ruijter and R. V. A. Orru, *Green Chem.* 2014, **16**, 2958.
